# The Role of Zinc and Iron-Folic Acid Supplementation on Early Child Temperament and Eating Behaviors in Rural Nepal: A Randomized Controlled Trial

**DOI:** 10.1371/journal.pone.0114266

**Published:** 2015-03-30

**Authors:** Pamela J. Surkan, Mary Katherine Charles, Joanne Katz, Emily H. Siegel, Subarna K. Khatry, Steven C. LeClerq, Rebecca J. Stoltzfus, James M. Tielsch

**Affiliations:** 1 Department of International Health, Johns Hopkins Bloomberg School of Public Health, Baltimore, Maryland, United States of America; 2 Vanderbilt University School of Medicine, Nashville, Tennessee, United States of America; 3 Nepal Nutrition Intervention Project—Sarlahi (NNIP-S), Hariaun, Nepal; 4 Division of Nutritional Sciences, Program in International Nutrition, Cornell University, Ithaca, New York, United States of America; 5 George Washington University, School of Public Health, Washington, District of Columbia, United States of America; University of Washington, UNITED STATES

## Abstract

Child eating behaviors play an important role in nutrient intake, ultimately affecting child growth and later outcomes in adulthood. The study assessed the effects of iron-folic acid and zinc supplementation on child temperament and child eating behaviors in rural Nepal. Children (N = 569) aged 4–17 months in Sarlahi district, southern Nepal were randomized to receive daily supplements of placebo, iron-folic acid, zinc, or zinc plus iron-folic acid and followed for approximately 1 year. At baseline and four follow-up visits mothers completed questionnaires including information on demographic characteristics and child temperament and eating behaviors. The main effects of zinc and iron-folic acid supplementation on temperament and eating behaviors were assessed through crude and adjusted differences in mean cumulative score changes between visits 1 and 5. The adjusted rate-of-change for these outcomes was modeled using generalized estimating equations. Mean changes in temperament scores and in eating behavior scores between visits 1 and 5 were not significant in either the zinc or non-zinc group. Children in the iron-folic acid group increased temperament scores by 0.37 points over 5 visits (95% CI 0.02, 0.7), which was not significant after adjustment. Neither the adjusted rate-of-change in temperament scores between zinc and non-zinc (β = −0.03, 95% CI −0.3, 0.2) or iron-folic acid and non-iron-folic acid (β = 0.08, 95% CI −0.2, 0.3) were significantly different. Adjusted rate of change analysis showed no significant difference between zinc and non-zinc (β = −0.14, 95% CI −0.3, 0.04) or between iron and non-iron eating behavior scores (β = −0.11, 95% CI −0.3, 0.1). Only among children with iron-deficiency anemia at baseline was there a significant decrease in eating behavior score, indicating better eating behaviors, when supplemented with zinc (β = −0.3, 95% CI −0.6, −0.01), Ultimately, this effect of zinc on eating behaviors was the only effect we observed after approximately one year of micronutrient supplementation.

**Trial Registration:**

ClinicalTrials.gov NCT00109551

## Introduction

Malnutrition remains a pervasive global problem, predominantly for children living in low- and middle-income countries. In South-central Asia, 33% of children under age five are estimated to be underweight, with micronutrient deficiencies affecting both physical and cognitive developmental processes.[1] As in many low-income countries, iron and zinc deficiencies are a significant problem in rural Nepal,[2] where an estimated 43% of children below the age of two suffer from iron-deficiency anemia[3] and 16% are estimated to have serum zinc levels under 9.2 μmols/L.[4] Both child temperament and child eating behaviors are related to various aspects of child nutrition.[5,6]

Child temperament has been characterized by individual differences in behavior, self-regulation and reactivity based on any given individual’s unique physiological status that tend to persist over time.[7,8] Temperament has been associated with both slow and fast weight gain in infants,[9,10] as well as child feeding practices[11] and eating behaviors.[12] Children with easy temperaments are thought to be more resilient e.g., with fewer behavioral problems, more social competence, and higher levels of adaptive behaviors.[13] Eating behaviors play an important role in nutrient intake,[14] ultimately affecting child growth[11] and long-term major health outcomes into adulthood.[15]

Iron and zinc play critical roles in developing children. Iron deficiency has been widely associated with tiredness, lowered mood, and poor concentration and cognition,[16] and there is evidence that iron deficiency affects psychological function due to decreased activity of iron-containing enzymes in the brain and reduced hemoglobin synthesis and delivery.[17,18] The temperament of undernourished rural Bangladeshi children appears to be different from that of better-nourished children; they are less sociable, less attentive, more fearful and exhibit more emotionality.[19] Temperament has also been associated with iron status in neonates, where lower levels of iron are linked to poorer alertness and more negative emotionality.[6] A report of preschool-aged children found child activity levels to be positively associated with hair zinc status.[20] In addition, zinc deficiency among infants has been associated with poor appetite and reduced dietary intake.[21]

While many studies have focused on iron and zinc in relation to developmental milestones, very little is known about the specific relationship between these micronutrients and temperament and eating behaviors. Therefore we examined the effects of zinc and iron supplementation on these behaviors in Nepali infants and toddlers.

## Materials and Methods

### Subjects

The analysis of child temperament and child eating behaviors was a sub-study of the Nepal Nutrition Intervention Project, Sarlahi (NNIPS-4), a micronutrient supplementation trial to assess the effect of iron-folic acid and zinc on mortality. The protocol for this trial and CONSORT checklist are available as supporting information; see S1 CONSORT Checklist and S1 Protocol. The NNIPS-4 study was conducted in 426 randomized geographic clusters within 30 Village Development Committees (VDCs) using a cluster randomized 2×2 factorial design. This study was conducted in the rural southern district of Sarlahi, Nepal within the Ishwarpur VDC. This was one of the larger VDCs, containing 23 geographic clusters.

Participants were eligible for participation if they lived in Ishwarpur between January 7 and April 6, 2002 and were between 4 and 17 months of age. The complete date range for participant recruitment and follow-up was January 7^th^ 2001 through May 7 2003. A total of 613 eligible infants were identified through a population-based census conducted between December 2000 and March 2001. Only 44 (7%) of the eligible infants did not participate, either because they were not located or their caregivers did not provide consent. The remaining 569 infants were randomized to the 4 study arms: placebo (N = 152), iron-folic acid (N = 129), zinc (N = 126), and zinc plus iron-folic acid (N = 162). Because most participants were illiterate, verbal informed consent was obtained from all caregivers. This was indicated on a box on the enrollment form, which was then signed by the field worker obtaining consent. The study and consent procedure was approved by the Johns Hopkins University Committee on Human Subjects Research and the Nepal Health Research Council. The authors confirm that this substudy as well as all ongoing and related trials, the main trial, are registered at clinicaltrials.gov (NCT00109551).

### Data Collection

Data were collected through questionnaires administered at baseline and during four follow-up visits at approximately three-month intervals. The duration of participation was approximately one year. During baseline and follow-up visits, demographic and developmental data were collected in addition to temperament and child eating behavior outcomes. Baseline demographic data included the following: infant age in months (4–5, 6–8, 9–11, 12–14, or 15–17), gender (male or female), caste (higher: Brahmin and Chetri; lower: Vaiysha, Shudra, or Muslim), ethnic group (Pahadi or Madeshi), maternal literacy (not literate, literate), paternal literacy (not literate, literate), and paternal occupation (farmer, including also unskilled worker, laborer, or unemployed; business, including also government, or private sector worker). Time of birth in relation to local festivals and the lunar calendar was used to calculate birth dates when necessary. Field workers identified the presence or ownership of 12 items to determine the socio-economic status (SES) of participants: a latrine at the house, a servant, cattle, bicycle, radio, farmable land, home garden plot, second floor in the house, type of roofing material, TV, electricity at home, and bullock cart. The ownership of these items was used to create a three-category SES scale: low (0–1 items), medium (2–5 items) and high (6–12 items). Baseline health was also measured through anthropometric measurements (weight-for-age, length-for-age, mid-upper-arm circumference, and triceps skinfold thickness), baseline diarrheal disease (yes/no for symptoms in last 5 days) and acute respiratory infection (yes/no in last 5 days), micronutrient status (hemoglobin < 10.0g/dL and zinc protoprophryin > 90 μmol), and iron deficiency anemia (zinc protoporphryin>90 μmol/no/heme and hemoglobin<10.0 g/dL). We also collected information on baseline feeding behaviors, including exclusive breastfeeding (yes/no) and meat eating at baseline (yes/no).

### Exposure

Iron-folic acid and zinc supplementation were the exposures of interest for this study. Children were randomized to receive a daily supplement of either 1) 10 mg zinc; 2) 12.5 mg iron and 50 μg folic acid; 3) a combination of 10 mg zinc, 12.5 mg iron and 50 μg folic acid; or 4) a sugar placebo. In order to randomize, all combinations of the 4 treatment groups were written on pieces of paper. Senior field personnel blindly and randomly drew pieces of paper from a container that indicated the codes for the first 4 sectors on the list. The paper was replaced and random drawing continued until all sectors were assigned to one of the four supplementation groups. Children under one year of age received half a dose. Vanilla flavored supplements were provided by Nutriset (Malaunay, France) in collaboration with the Department of Child and Adolescent Health and Development (WHO, Geneva, Switzerland). Independent laboratories performed chemical analyses to monitor supplement potency and composition. The children were given the appropriate micronutrient supplements twice a week directly from fieldworkers. Young children were given tablets dissolved in breast milk or purified water when necessary. When possible, study staff administered supplements, but caregivers administered doses when study staff were unavailable or on days without home visits. Field staff monitored compliance by counting the number of tablets consumed since the prior visit. Tablets looked identical and codes and contents were not revealed to any members of the field staff or data analysts until completion of the study, resulting in complete masking of each participating family and all fieldworkers to the randomization of the intervention. Although a treatment code was printed on the micronutrient packaging, all tablets looked identical.

### Development Outcomes

Child temperament was assessed by the caregiver using a scale adapted from the temperament component of the Infant Characteristics Questionnaire.[22] The adapted scale is a twelve-item instrument that includes eleven yes or no questions and a summary question asking about the relative ease or difficulty in caring for the child (easier than average, average or more difficult than average). Temperament questions focused on topics such as intensity of crying, mood, irritability, need for attention, fussiness, and independence. For each question, one point was associated with a poor temperament while zero points were awarded for good temperament. The summary three-category question was dichotomized such that children characterized as “more difficult than average” received one point, while children characterized as “average” or “easier than average” received zero points. The temperament scale was constructed by summing the 12 questions. Temperament scores could range from 0 to 12, with lower scores indicating easier temperament.

The eating behavior questionnaire consisted of nine commonly used questions about behaviors while eating and appetite. Some examples include the relative amount of eating for age, happiness during eating, and frequency with which the infant requested food. The first eight questions had yes or no response options. One point was awarded to indicate poor eating behaviors, while no points indicated better eating behaviors. The ninth question, assessing the relative quality of the child’s appetite, had three response categories (good, average and poor). No points were awarded for the categories “good” and “average,” while “poor” appetite was awarded one point. The scores from each question were summed to create the composite eating behavior scale. Eating behavior scores could range from 0 to 9, with lower scores indicating better eating behaviors.

### Statistical Analysis

Data were analyzed using STATA 11.0 (Statacorp, College Station, Texas). Baseline comparability between treatment groups was assessed for temperament and eating behaviors using Generalized Estimating Equations (GEE) to account for the cluster randomization. Compliance with the assigned treatment group was compared to placebo by calculating the mean number of supplements taken.

In this 2x2 factorial study, one arm received a combination dose of zinc plus iron-folic acid. We tested for an interaction between the effects of zinc-only and iron-folic acid-only to see if the effect of joint administration differed from what would be expected from summing each treatment under the assumption of independence. No interaction effect was found, so the main effects of zinc and iron-folic acid were estimated separately: children who received any zinc were compared to those who did not receive zinc, and children who received any iron-folic acid were compared to those who did not receive iron-folic acid.

Children were included in the temperament analysis if they had a baseline score and at least one follow-up visit. For child eating behaviors, children whose mothers reported their child consuming only breast milk or water at baseline or any subsequent visits did not complete the module for that visit. If a child began consuming complementary foods, then the eating behavior module could be completed in follow-up visits. The GEE eating behavior analysis included all children with eating behavior data, including those who were not eligible for baseline data collection. Temperament and eating behavior data were counted as missing if more than one third of questions were incomplete for the visit. When less than one third of questions were missing but the module was not complete, the remaining questions were averaged and multiplied by the total number of questions on the respective scales (12 for temperament and 9 for eating behaviors).

For temperament and eating behaviors separately, we calculated the mean cumulative change in scores between the first and fifth visit. The crude and adjusted difference in mean cumulative change between first and fifth visit scores was also calculated to compare treatment interventions (zinc and iron folic-acid). Adjusted temperament and eating behavior models accounted for baseline differences in SES, ethnicity, caste, and paternal occupation. We also adjusted for the difference in temperament score between treatment groups at baseline, but no adjustment was made for baseline eating behavior scores because they were comparable at baseline. Because of the age-dependent nature of temperament and eating behaviors, all adjusted analyses accounted for age at baseline. The adjusted rate-of-change for temperament and eating behaviors was subsequently modeled using GEE. This GEE analysis accounted for all follow-up visits with complete data. Given baseline differences in levels of iron-deficiency anemia, we performed an unstratified analysis and an analysis stratified by iron-deficiency anemia status. After examining correlation matrices and time series graphs for temperament and eating behavior outcomes, an auto-regressive correlation structure was selected.

## Results

### Child Temperament

**Fig 1:**
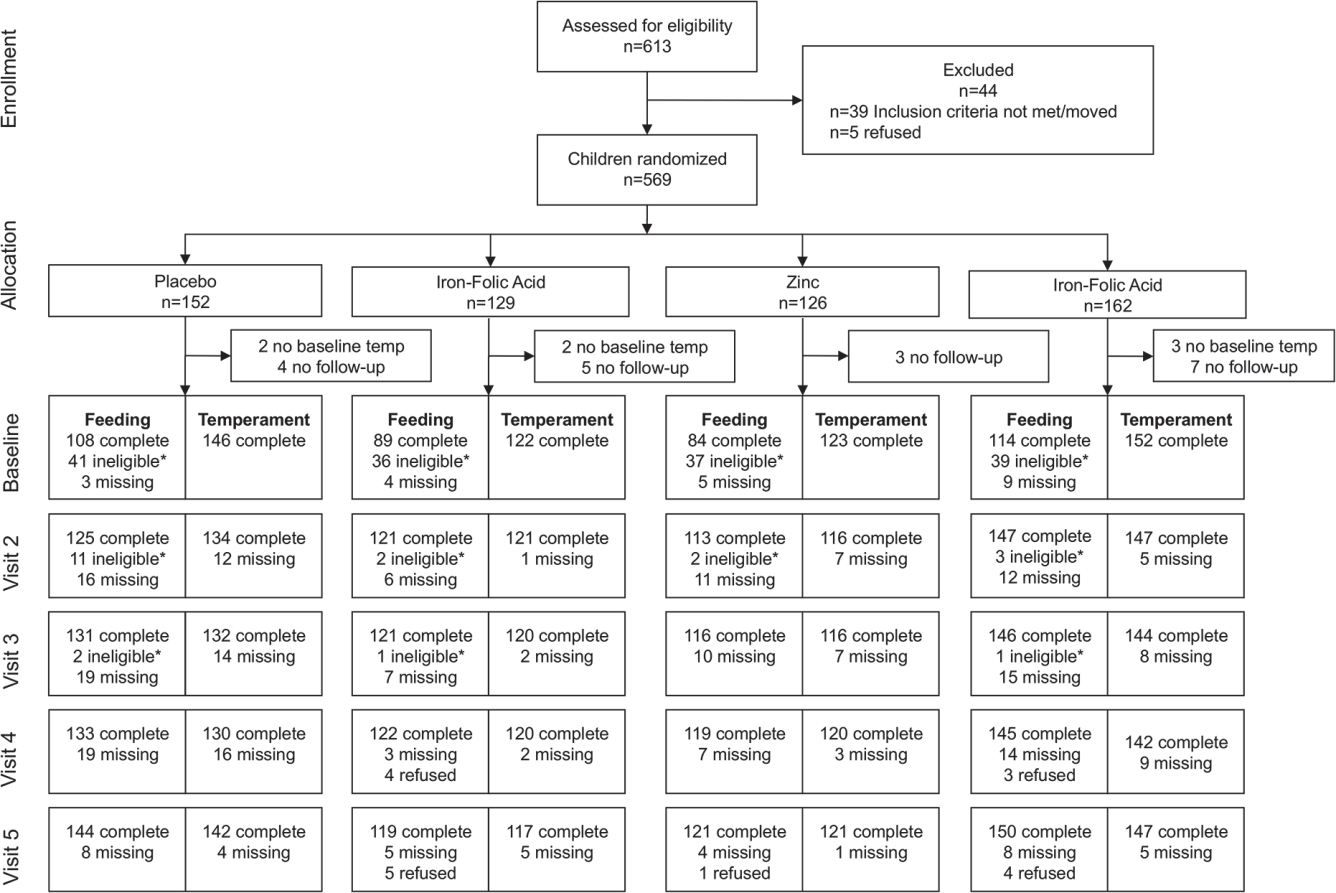
CONSORT Diagram of Participant Flow. *Infants were ineligible for eating behavior measurements during a visit if they consumed only breast milk and/or water.

Of the total 569 infants randomized to the four study arms, the temperament analysis excluded 1.2% (N = 7) of infants due to missing baseline scores and 3.3% (N = 19) without any follow-up visits. The final analysis accounted for 543 infants remaining in each treatment group: iron-folic acid (N = 122), zinc (N = 123), iron-folic acid plus zinc (N = 152), or placebo (N = 146) (Fig. 1). Infants with complete outcomes for visits one through five were available for 94.3% of infants receiving iron-folic acid, 86.2% of zinc, 87.5% of iron-folic acid plus zinc, and 80.1% of placebo. Infants were comparable across treatment groups in age, sex, and maternal and paternal literacy. They were not comparable however in caste, ethnicity, SES, paternal occupation and baseline temperament score (Table 1). For infants included in the temperament analysis, compliance with treatment was lowest among the group taking iron-folic acid only and highest among the placebo group. Infants who received any iron took significantly fewer supplements than those not receiving iron, while there was not a significant difference in compliance between those in the zinc and non-zinc groups. An intention to treat analysis was conducted.

**Table 1:** Background Characteristics^1^.

	Placebo n (%)	Iron-Folic Acid n (%)	Zinc n (%)	Zinc + Iron-Folic Acid n (%)	p-value^2^
Total	152	129	126	162	
**Baseline age (months)**					
4–5	24 (15.8)	17 (13.2)	23 (18.3)	26 (16.2)	0.35
6–8	23 (15.1)	27 (20.9)	33 (26.2)	26 (16.2)	
9–11	30 (19.7)	33 (25.6)	18 (14.3)	35 (21.7)	
12–14	39 (25.7)	31 (25.0)	28 (22.2)	42 (26.1)	
15–17	36 (23.7)	21 (16.3)	24 (19.1)	32 (19.9)	
**Sex**					
Female	82 (54.0)	58 (45.0)	73 (57.9)	72 (44.4)	0.06
Male	70 (46.1)	71 (55.0)	53 (42.1)	90 (55.6)	
**Caste** ^**3**^					
Higher	9 (5.9)	7 (5.4)	20 (15.9)	15 (9.3)	0.01
Lower	143 (94.1)	122 (94.6)	106 (84.1)	146 (90.7)	
**Socioeconomic status** ^**4**^					
0–1 possessions	27 (18.0)	40 (31.5)	11 (9.2)	41 (25.8)	<0.01
2–5 possessions	81 (54.0)	57 (44.9)	72 (60.0)	77 (48.4)	
6–12 possessions	42 (28.0)	30 (23.6)	37 (30.8)	41 (25.8)	
**Ethnicity**					
Madheshi	131 (86.8)	108 (84.4)	92 (73.6)	126 (77.8)	0.02
Pahadi	20 (13.3)	20 (15.6)	33 (26.4)	36 (22.2)	
**Literacy**					
Maternal literacy	24 (15.8)	19 (14.8)	21 (16.7)	26 (16.2)	0.98
Paternal literacy	77 (50.7)	64 (50.0)	73 (57.9)	80 (49.7)	0.49
**Paternal occupation**					
Farmer	112 (73.7)	106 (82.8)	99 (78.6)	109 (67.3)	0.02
Businessman	40 (26.3)	22 (17.2)	27 (21.4)	53 (32.7)	
**Anthropometric measurements (sd)**					
Weight-for-age (z-score)	−1.6 (1.3)	−1.6 (1.2)	−1.7 (1.2)	−1.5 (1.2)	0.89^5^
Length-for-age (z-score)	−1.6 (1.0)	−1.3 (1.2)	−1.0 (1.0)	−1.3 (1.1)	0.10^5^
Mid-upper arm circumference (cm)	12.9 (1.0)	12.9 (0.9)	12.7 (1.0)	13.1 (1.0)	0.51^5^
Triceps circumference (cm)	6.8 (1.3)	7.0 (1.3)	6.8 (1.1)	7.2 (1.4)	0.07^5^
**Baseline health**					
Diarrheal illness^6^	35 (74)	40 (34)	21 (28)	44 (30)	0.57
Respiratory disease^7^	4 (3)	3 (3)	0 (0)	1 (1)	0.25
Exclusive breastfeeding	26 (17)	19 (15)	22 (17)	27 (17)	0.93
**Micronutrient status at baseline (sd)**					
Hemoglobin<10.0	68 (45)	50 (39)	40 (32)	68 (42)	0.20
Zinc protoporphyrin>90	97 (64)	88 (68)	56 (44)	103 (64)	0.001
Iron-deficiency anemia^8^	52 (34)	43 (33)	22 (17)	54 (33)	0.01
**Baseline scores** ^9^					
Mean temperament score (sd)	4.0 (2.2)	4.1 (2.5)	4.2 (2.4)	3.4 (2.3)	0.02^5^
Mean eating behavior score (sd)	3.2 (1.8)	3.1 (1.5)	3.1 (1.8)	2.7 (1.7)	0.14^5^

^1^One child was missing age group, paternal occupation and caste data; 36 were missing zinc protoporphyrin at baseline, 30 were missing hemoglobin at baseline, 19 were missing mid-upper arm circumference, 13 children were missing socioeconomic status data, 6 were missing breastfeeding data, 3 were missing ethnicity data, and 2 were missing maternal and paternal literacy. 80 were missing for each of iron-deficiency anemia, diarrheal disease, respiratory illness, and triceps circumference; 67 were missing for length- and weight-for-age z-scores.

^2^Chi-square p-values for categorical variables

^3^Lower caste refers to Vaiyshas, Shudras and Muslims. Higher caste refers to Brahmins and Chetris.

^4^Possessions: Presence of a latrine at the house, a servant, cattle, bicycle, radio, farmable land, home garden plot, second floor in the house, roof, TV, electricity at home, and bullock cart

^5^ANOVA-based p-values for continuous variables

^6^Defined by diarrhea present in previous 5 days

^7^Defined by acute respiratory infection in previous 5 days

^8^Defined by zinc protoporphryin>90 μmol/no/heme and hemoglobin<10.0 g/dL

^9^Seven children were missing baseline temperament scores and 174 children were ineligible for baseline eating behavior measurements.

**Fig 2:**
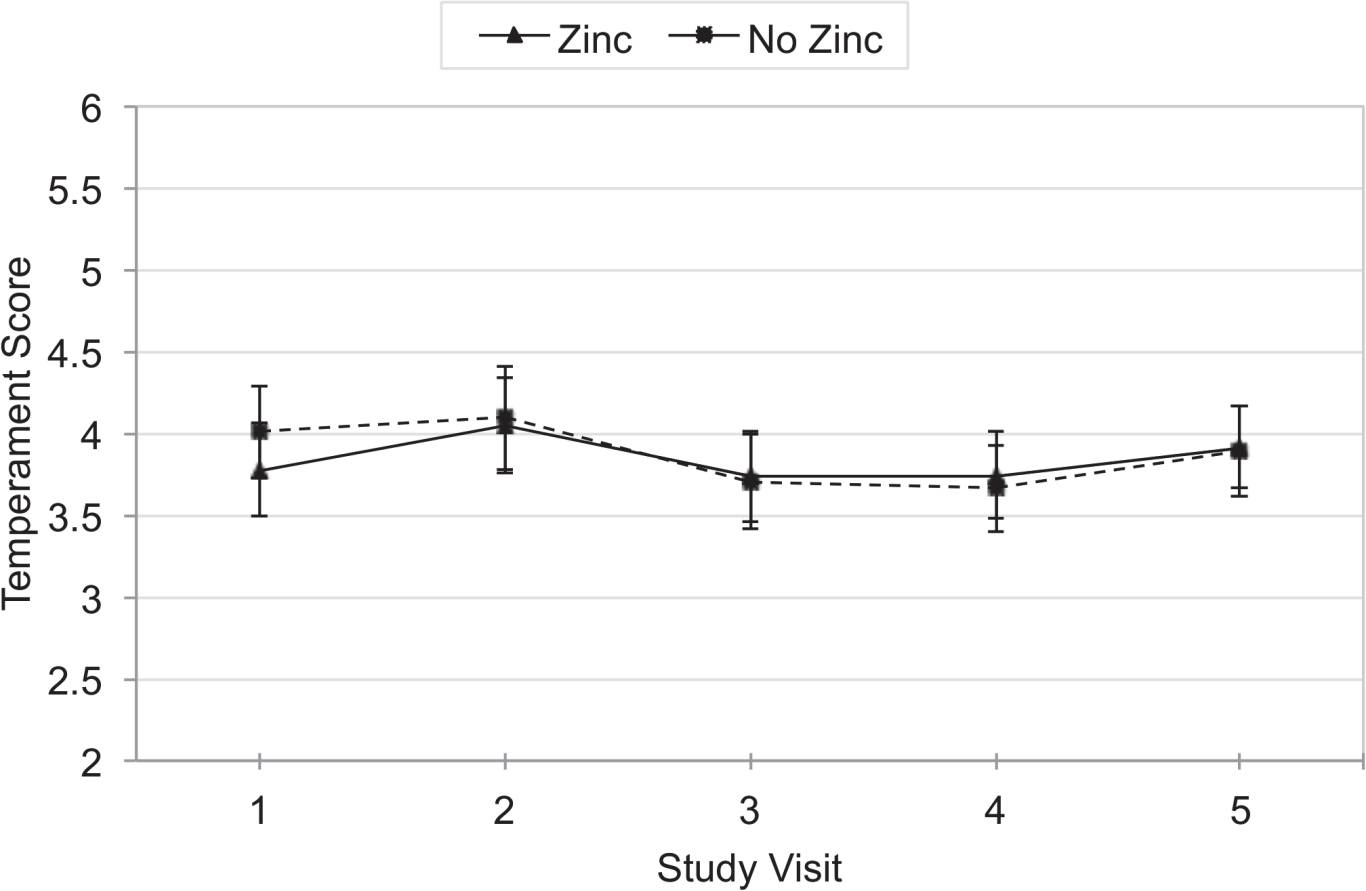
Mean Adjusted Temperament Score by Zinc over Time.

The mean change in temperament score between visit 1 and visit 5 was not significant in either the zinc or non-zinc group. The zinc group increased 0.16 points (95% CI −0.2, 0.5) and the no zinc group decreased 0.08 points (95% CI −0.4, 0.3) (Fig. 2). Comparing zinc and non-zinc supplementation groups using data from all visits in the adjusted rate-of-change GEE analysis also showed no difference in temperament scores (β = −0.03, 95% CI −0.3, 0.2)(Table 2), and there were no differences detected when stratified by iron-deficiency (Table 3).

**Table 2:** Main effects of zinc and iron supplementation on temperament and eating behavior score.

	Adjusted^1^ difference in rate-of-change for *temperament* scores between visits 1–5^3^	Adjusted^2^ difference in rate-of-change for *eating behavior* scores between visits 1–5^4^
	β (95% CI) (GEE)	β (95% CI) (GEE)
Zinc (n = 268)	−0.03 (−0.3, 0.2)	−0.14 (−0.3, 0.04)
No Zinc (n = 259)	ref	ref
Iron (n = 264)	0.08 (−0.2, 0.3)	−0.11 (−0.3, 0.1)
No Iron (n = 263)	ref	ref

^1^Adjusted for age category at baseline, ethnicity, SES category, caste, paternal occupation, and baseline temperament score.

^2^Adjusted for age category at baseline, ethnicity, SES category, caste, paternal occupation; baseline eating behavior scores were not controlled for because they did not differ at baseline.

^3^Analysis among n = 532 who had any outcome data for the post-intervention period (i.e., visits 2 through 5) and complete data on relevant baseline covariates.

^4^Analysis among n = 381 who had any outcome data for the post-intervention period (i.e., visits 2 through 5) and complete data on relevant baseline covariates.

**Table 3:** Main effects of zinc and iron supplementation on temperament and eating behavior score stratified by baseline iron deficiency.

	Adjusted^1^ difference in rate-of-change for *temperament* scores between visits 1–5^3^		Adjusted^2^ difference in rate-of-change for *eating behavior* scores between visits 1–5^4^	
	β (95% CI) (GEE)		β (95% CI) (GEE)	
	Iron deficient	Non-iron deficient	Iron deficient	Non-iron deficient
Zinc (n = 268)	−0.3 (−0.7, 0.1)	0.05 (−0.2, 0.3)	−0.3 (−0.6, −0.01)^5^	−0.02 (−0.2, 0.2)
No Zinc (n = 259)	ref	ref	ref	Ref
Iron (n = 264)	0.1 (−0.3, 0.6)	0.09 (−0.2, 0.4)	−0.1 (−0.5, 0.2)	−0.1 (−0.4, 0.1)
No Iron (n = 263)	ref	ref	ref	ref

^1^Adjusted for age category at baseline, ethnicity, SES category, caste, paternal occupation, and baseline temperament score.

^2^Adjusted for age category at baseline, ethnicity, SES category, caste, paternal occupation; baseline eating behavior scores were not controlled for because they did not differ at baseline.

^3^Analysis among n = 532 who had any outcome data for the post-intervention period (i.e., visits 2 through 5) and complete data on relevant baseline covariates.

^4^Analysis among n = 381 who had any outcome data for the post-intervention period (i.e., visits 2 through 5) and complete data on relevant baseline covariates.

^5^Significant at the p<0.05 level

**Fig 3:**
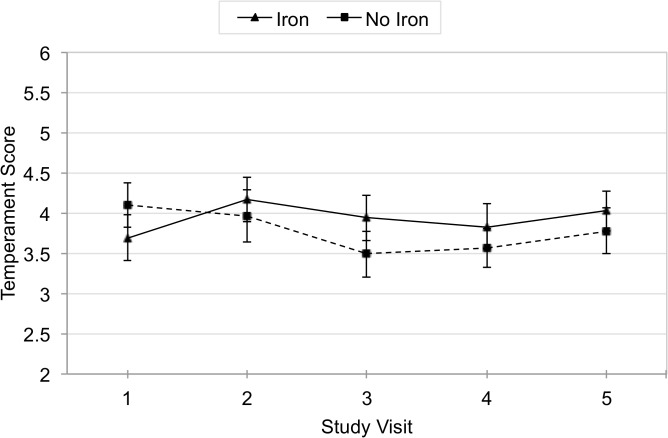
Mean Adjusted Temperament Score by Iron over Time.

For those receiving any iron, the mean temperament score from visit 1 to visit 5 increased by 0.37 points (95% CI 0.02, 0.7), while infants in the non-iron group had a non-significant decrease of 0.29 points (95% CI −0.6, 0.1) (Fig. 3). After adjusting for baseline temperament score and socio-demographic differences, there was no significant difference between iron and non-iron groups in the mean score change between visits 1 and 5. The adjusted GEE analysis using non-missing data was also not significant between iron and non-iron groups (β = 0.08, 95% CI −0.2, 0.3) (Table 2). Stratified GEE demonstrated no significant change in temperament score between baseline iron-deficient and non-iron-deficient children (Table 3).

In the analysis of unadjusted and adjusted difference in mean temperament scores between visit 1 and 5, there were no significant difference between zinc and non-zinc groups or between iron and non-iron groups.

### Child Eating Behaviors

Of the 569 infants that consented to participate, 174 infants (30.6%) were not eligible to complete the baseline child eating behavior module. An additional 13 (2.3%) infants had no follow-up visits. This left 382 infants for an analysis comparing visits 1 through 5 among iron-folic acid (N = 85), zinc (N = 82), zinc and iron-folic acid (N = 109), and placebo (N = 106) (Fig. 1). Complete visit data for visits one through five were available for 94.1% of infants receiving iron-folic acid, 89.0% of zinc, 83.5% of iron-folic acid plus zinc, and 78.0% of placebo. GEE analysis was used to incorporate all infant data, regardless of baseline completion.

Sex, age, SES, maternal and paternal literacy, and eating behavior scores were comparable between supplement groups at baseline for the eating behavior analysis. The caste and ethnicity were significantly different at baseline and were adjusted for in subsequent analyses. Similar to the temperament subset, compliance with the intervention was highest in the placebo group and lowest in the iron-folic acid group. Infants in the iron group took significantly fewer supplements than the infants in the non-iron group. However, infants in the zinc group were significantly more compliant with treatment than infants in the non-zinc group. At baseline, exclusive breastfeeding occurred in 17.1% of placebo, 14.7% of iron-folic acid, 17.5% of zinc, and 16.7% of iron-folic acid plus zinc children (p = 0.93). Meat was included in the diet of 32.8% of placebo, 29.5% of iron-folic acid, 29.4% of zinc, and 27.1% of iron-folic acid plus zinc children (p = 0.09).

**Fig 4:**
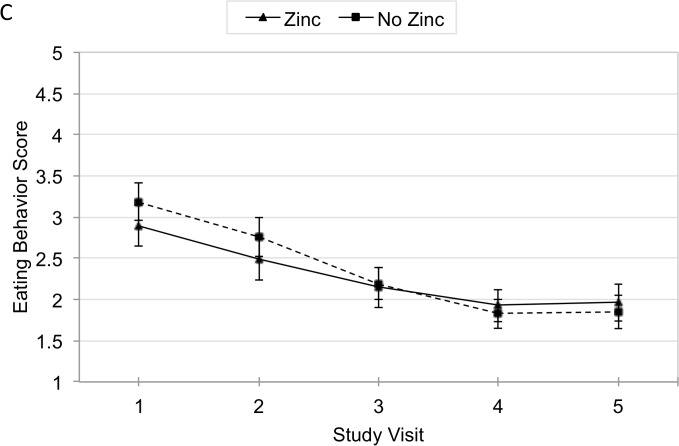
Mean Adjusted Eating Behavior Score by Zinc over Time.

The main effects of zinc and iron supplementation on eating behavior score were first analyzed for infants with complete baseline data between visits 1 and 5. The mean decrease in eating behavior score between visit 1 and 5 for those receiving zinc was 0.92 points (95% CI −1.2, 0.6), and 1.29 points (95% CI −1.6, 1.0) for those not receiving zinc (Fig. 4). Using all non-missing data between visits 1 and 5 in the GEE analysis showed no significant difference between zinc and non-zinc eating behavior scores (β = −0.14, 95% CI −0.3, 0.04) (Table 2). However, baseline iron-deficient anemic children had a significant decrease in eating behavior score when supplemented with zinc (β = −0.3, 95% CI −0.6, −0.01), while those without iron-deficiency anemia at baseline had no significant change in eating behavior score with zinc supplementation (Table 3).

**Fig 5:**
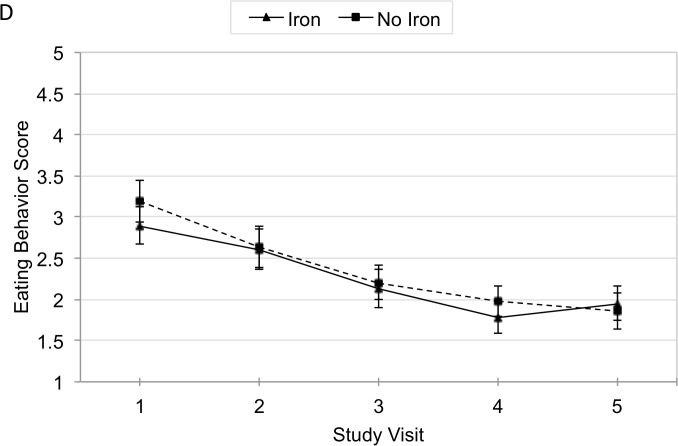
Mean Adjusted Eating Behavior Score by Iron over Time.

The mean decrease in eating behavior score between visits 1 and 5 was 0.88 points (95% CI −1.2, −0.7) in the iron group and 1.33 points (95% CI −1.6, −1.1) in the non-iron group (Fig. 5). The GEE modeled rate-of-change of eating behavior score was also not significant between iron and non-iron groups (β = −0.11, 95% CI −0.3, 0.1) (Table 2). A stratified analysis based on iron-deficiency status demonstrated no significant change in eating behavior scores (Table 3).

Performing an analysis of unadjusted and adjusted difference in mean eating behavior scores between visit 1 and 5 showed no significant difference between zinc and non-zinc groups or between iron and non-iron groups.

## Discussion

Results of this community trial indicated no effect of zinc or of iron-folic acid supplementation on either early child temperament or child eating behaviors, using a longitudinal design to follow children over the course of approximately one year. As illustrated in the graphs, temperament scores were relatively constant over the five visits. Overall, child eating behaviors improved with age, reflected by lower scores over time. The scores dropped more in the non-zinc versus the zinc supplementation group and in the non-iron-folic acid versus the iron-folic acid supplementation group, although the difference in the rate of change between groups was not statistically significant. However, we did detect an association suggesting better eating behaviors (indicated by lower scores) associated with zinc supplementation among those baseline iron-deficiency anemia at baseline.

We did not find an association between iron-folic acid supplementation and child temperament scores, despite previous studies suggesting that iron deficiency anemia is associated with behavioral development in infants and preschool children.[23–25] In a study of Indian preschoolers in New Delhi, children with iron-deficiency anemia displayed less social looking at their mothers and were slower to display positive affect.[26] Iron deficiency anemia in infants has also been linked to poorer maternal-child interactions, including reduced maternal responsivity and less sensitivity to infant cues,[27,28] which could affect eating behaviors through the reciprocal nature of caregiver-child feeding interactions.[29] The qualitative component of a study from India, in which weekly iron-folic acid supplements were given to older girls, found that they reported an increase in appetite as a result of the supplementation.[30] One of the few research reports that specifically measured child temperament in relation to iron supplementation was an Australian randomized controlled trial in which pregnant women were provided iron supplements during approximately the last 20 weeks of pregnancy. No differences were found in scores on the Temperament Scale for Children in the supplement or non-supplement group at follow up when children were between 6 to 8 year old.[31] In a study of childhood anemia among low-income urban American children and their mothers, low childhood hemoglobin status was significantly correlated with maternal perception of feeding difficulty.[32]

In our study, zinc supplementation was not related to child temperament. However, we did detect a modest association suggesting zinc supplementation to be related to better eating behaviors in children with baseline iron-deficiency anemia. We are unaware of other previous studies assessing these associations. Nonetheless, zinc supplementation has been associated with numerous other behavioral outcomes, though results are mixed.[33–37] For example, Guatemalan children receiving six months of zinc supplementation showed fewer internalizing symptoms than control children, however no associations were found between other behavioral symptoms such as hyperactivity, aggression, or conduct.[37] In a Brazilian study of one year olds, child responsiveness as well as a composite measure of responsiveness, emotional tone, activity level, cooperation, and vocalization were significantly rated higher in a group of low birth weight term infants receiving 5 mg zinc for eight weeks, compared to similar infants in a placebo group.[33] Attention was shown to be better in Egyptian infants consuming foods high in zinc.[36] Two studies of infants in India and in Guatemala showed increases in activity in zinc supplementation groups compared to a group given a multivitamin supplement without additional zinc or compared to a non-supplementation group, respectively.[34,35]

Though the reasons for our finding limited effects are unclear, it is possible that supplementation may only have an effect if the duration is long enough and timing optimal and/or the degree of deficiency in the children is sufficiently severe. Given our study was a year in duration and timing of supplementation overlapped during the peak ages of iron-deficiency in childhood,[38] it is possible that there is in fact no effect of these micronutrient supplements on our outcomes of interest. Further research should be carried out for confirmation of these findings.

One strength of this study was the randomized controlled design in a setting with a high burden of malnutrition.[3,4] Both fieldworkers and families were masked to group assignment. We also had very good retention of participants (86.1% with data for all visits) and adherence to supplementation (91.1% who had at least a baseline and last visit), limiting the possibility of bias due to participant selection or drop-out. One limitation was the use of maternal self-report rather than direct observations of our main outcomes, however videotaping and coding was not possible due to budgetary constraints. Given that mothers reported child behaviors, social desirability could have come into play, since mothers might be reluctant to report their children having difficult temperaments or poor eating behaviors. Nonetheless, maternal reports are considered to be valid measures of child temperament.[7] There were also some demographic differences resulting from the randomization procedure at baseline including child temperament scores, but we accounted for these differences in our statistical analysis.

Ideally, future research could use direct observations rather than reported measures of these concepts. At present there is little information about the pathways by which zinc or iron-folic acid supplementation could affect child temperament or eating behaviors. Further, data are inconclusive regarding the ages that zinc or iron supplementation may have the greatest impact. A better understanding of these processes and what aspects of temperament or eating behaviors might be affected may provide insight into identifying the most sensitive measures for future studies.

### Conclusions

Given that our study showed almost no evidence for effects of zinc or iron supplementation on child temperament or on child eating behaviors, except for an effect of zinc supplementation on better eating behaviors in children with iron-deficiency anemia, more research is needed to confirm these results in other settings, among children of different ages, and with different micronutrient profiles.

## Supporting Information

S1 CONSORT Checklist CONSORT Checklist. (DOC)

S1 Protocol Trial Protocol. (DOC)

S1 Data Study Data. (DTA)
