# Supplementary material for: The Role of Zinc and Iron-Folic Acid Supplementation on Early Child Temperament and Eating Behaviors in Rural Nepal: A Randomized Controlled Trial
Source: PLoS One. 2015 Mar 30;10(3):e0114266. doi: 10.1371/journal.pone.0114266 (PMC4379028; doi:10.1371/journal.pone.0114266)
Supplement: S1 Protocol — (DOC) [file pone.0114266.s002.doc]

# DIMAG Manual of Operations

**1. INTRODUCTION TO DIMAG**

DIMAG, Development of Infant Motor skills, Articulations, and Growth, will take place between December 2001 and May 2003 in Ishwarpur VDC in Sarlahi, Nepal. The office will be located in Bailbas near the bazaar, approximately 24 kilometers east of Hariaun. Excluding Emily Siegel, the student in charge of running the sub-study, 23 people will be needed to carry out the demands of the study. These people include one Nepali project manager, 15 observers, 4 clinic staff members, 2 peons/night watchmen, and 1 sweeper. After a 3-month period of enrollment, 568 infants enrolled in NNIPS-4 and between the ages of 4-and 16-months will be followed for one year. During this time they will be observed in their homes and brought to the office for the clinic staff to take blood samples, anthropometric measurements, and perform cognitive testing.

**1.1 Research question**

This sub-study is designed to examine the impact of zinc and iron/folate supplementation on the cognitive development of young children living in Sarlahi District, Nepal.

### 1.2 Rationale

In recent years, the micronutrients iron and zinc have received increased attention due to their role in facilitating growth and development in children. Children consume the nutrients in sufficient quantities in breast milk when they are exclusively breast fed for the first six months of life (Brown et al., 1998). However, beginning at six months children require amounts of iron and zinc that cannot be met by breast milk alone (Brown et al., 1998). They must receive complementary foods that are rich in both nutrients. Those who are born to families who eat a predominately plant-based diet, often have difficulty obtaining iron and zinc in a sufficient quantity because the nutrients found in non-animal products, such as green leafy vegetables (iron) and grains (zinc), are not easily absorbed. In the East-Central Terai region of Nepal, where economic constraints limit the access to animal products, a large percentage of the population subsists on plant-based diets. Due to cultural norms in this area, children often are forced to compete with older family members for food, placing them at risk for eating less food and a more limited diet than their family members (Shankar et al., 1998). Nepalese children are at risk for iron and zinc deficiency and, consequently, developmental delays.

Both iron and zinc are needed for brain function. Data in both the animal and human literature suggest that deficiencies in iron and zinc cause decreased motor development, activity, and cognitive functioning. Few experimental trials have been performed to test the effects of iron supplementation on infant development (Pollitt, 2000); however, evidence suggests that iron supplementation improves motor, language, and cognitive development (Stoltzfus, 2001; Logan 2001). Experimental trials found associations between zinc supplementation and improved indices of mental and motor development and activity in vulnerable infants. These differences were detectable with general tests of sensor motor development, which captured behavior as a means of describing mental and motor development. Iron and zinc supplementation studies examining attention, recognition memory, and recall, all specific measures of cognitive functioning, have not been conducted using a randomized placebo-controlled design with this age group. More research is needed to delineate how differences in iron and zinc nutriture influence specific cognitive functions.

**1.3 Goal and specific aims**

The goal of this project is to improve the development of Nepali infants living in Sarlahi District, Nepal through daily supplementation with 10-mg zinc (5-mg for infants less than 12 months), 12.5-mg iron plus 50-μg folate, both iron/folate and zinc, or neither. The primary and secondary aims of the study follow.

Primary Aim: To measure whether zinc or iron-folate supplementation provided over a 12-month period to Nepali infants 4-16 months of age improves motor, social, and cognitive development, activity, temperament, and sleep duration.

Secondary Aim: To describe the development of rural Nepali infants from 4 -16 months of age.

**1.4 References**

Brown K.H., Dewey K.G., and Allen L.H. (1998). Complementary Feeding of Young Children in Developing Countries: A Review of Current Scientific Knowledge. Geneva: World Health Organization.

Brown K.H., Peerson J.M., Allen L.H. (1998). Effect of zinc supplementation on children’s growth: a meta-analysis of intervention trials. In: B. Sandstrom and P.Walter (Eds.): Role of Trace Elements for Health Promotion and Disease Prevention. Bibl Nutri Dieta 54: 76-83.

Logan 2001

Pollitt E. (2000). The developmental and probabilistic nature of the functional consequences of iron deficiency anemia among children. The Journal of Nutrition 131 (2SII): 669S-675S.

Shankar A.V., Gittelsohn J., West K.P., Stallings R., Gnywali T., and Faruque F. (1998). Eating from a shared plate affects food consumption in vitamin A-deficient Nepali children. Journal of Nutrition 129: 1127-1133.

Stoltzfus R.J., Kvalsvig J.D., Chwaya H.M., Montresor A., Albonico M., Tielsch J.M., Savioli L., and Pollitt E. (2001). Effects of iron supplementation and anthelminthic treatment on motor and language development of preschool children in Zanzibar: double blind, placebo controlled study. British Medical Journal 323: 1389-1393.

**2. METHODS**

**2.1 Overview**

Infants 4- to 16-months of age enrolled in the larger NNIPS 4 mortality trial and living in Ishwarpur at baseline will be eligible to participate in DIMAG. The 568 infants in this age group will be randomly assigned to one of the four treatment groups before being followed for 12-months and assessed both in the home and the clinic. A two-story concrete house located near the bazaar in Bailbas (ward 2), Ishwarpur has been designated as the clinic setting and will be used for developmental testing, anthropometry, and blood collection. The subjects will be transported to the office in one of the NNIPS-4 vehicles when they are scheduled for the clinic visits. The observers will travel to their homes on bicycles when it is time for the home visits.

Table 1 summarizes the data that will be collected during the course of the study. It breaks the data into outcome variable, measure, and time in months when each of the measures will be used. With the exception of a few select measures (one motor development (MMWD), the cognitive development, the micronutrient status, and four of the five growth measures), the data will be collected at three-month intervals. DIMAG staff will collect all of the data except the MMWD, which will be the NNIPS-4 Ishwarpur WDs’ responsibility. The observers will collect the motor and social development, activity, temperament, sleep duration, dietary pattern, and head circumference measures; whereas, the clinic staff will collect the cognitive development, micronutrient status, and remaining growth measures. Both the observers and clinic staff will assess morbidity prior to the field and clinic visits.

| **Table 1. Summary of data to be collected** | |  |  |  |  |  |
| --- | --- | --- | --- | --- | --- | --- |
| **Outcome Variables** | **Measures** | **Time (months)** | | | | |
|  |  | **0** | **3** | **6** | **9** | **12** |
| Motor Development |  |  |  |  |  |  |
|  | Motor Milestones Worksheet DIMAG (MMWD)^1^ | XXXXXXXXXXXXXXXXXXXXXXXXXX | | | | |
|  | Parental report motor scale | X | X | X | X | X |
|  | Observation | X | X | X | X | X |
| Social Development |  |  |  |  |  |  |
|  | Parental report language scale | X | X | X | X | X |
|  | Observation | X | X | X | X | X |
| Cognitive Development^2^ |  |  |  |  |  |  |
|  | Fagan Test of Infant Intelligence | XXXXXXXXXXXXXXXXXXXXX | | | |  |
|  | A-not-B Test | XXXXXXXXXXXXXXXXXXXXX | | | |  |
| Activity |  |  |  |  |  |  |
|  | Actiwatch | X | X | X | X | X |
|  | Observation | X | X | X | X | X |
| Temperament |  |  |  |  |  |  |
|  | Revised Bates Scale | X | X | X | X | X |
| Sleep Duration |  |  |  |  |  |  |
|  | Questions from TAPP Scale | X | X | X | X | X |
| Micronutrient Status |  |  |  |  |  |  |
|  | Hemoglobin | X |  |  |  | X |
|  | Zinc protoporphyrin | X |  |  |  | X |
| Dietary Pattern |  |  |  |  |  |  |
|  | Food frequency | X | X | X | X | X |
|  | Appetite | X | X | X | X | X |
| Growth |  |  |  |  |  |  |
|  | Recumbent length | X |  |  |  | X |
|  | Weight | X |  |  |  | X |
|  | Head circumference | X | X | X | X | X |
|  | Mid-upper arm circumference | X |  |  |  | X |
|  | Upper-arm skin fold thickness | X |  |  |  | X |
| Morbidity |  |  |  |  |  |  |
|  | Scale | X | X | X | X | X |
| ^1^The MMWD will be administered to NNIPS-4 children in Ishwarpur, including those enrolled in DIMAG. Data will be collected weekly. | | | | | | |
| ^2^Both of the cognitive assessment data will be collected when the DIMAG children are 39- and 52-weeks of age. | | | | | | |

**2.2 Motor development**

Motor development will be investigated using three different methods: the Motor Milestones Worksheet, the Parental Report, and observation. Although data on a range of motor activities will be collected, the investigators are primarily interested in the development of bipedal locomotion.

The Motor Milestones Worksheet-DIMAG (MMWD) is an instrument that Dr. Ernesto Pollitt revised to pinpoint the onset of developmental milestones. The MMWD is designed for the mother of the target child to keep the form in the house and to fill in the date after a particular milestone, such as crawling or standing without support, is achieved. Because the rates of literacy in our study area are low, our Ward Distributors (WDs) will administer this form to the mothers of all the NNIPS-4 subjects. The WDs will visit each of the study infants’ houses weekly and will inquire whether any of the fourteen milestones has been completed in the past 7 days. When a milestone has been completed, the WDs will record the NNIPS week that it occurred on the MMWD, the number of the milestone on the NNIPS-4 CDR-D Form, and will ask to see the child perform the milestone. Only prospective data will be collected using this method. Every month data from the NNIPS-4 CDR-D Forms, on which the data will be recorded, will be taken to Kathmandu for data entry and analysis.

The Parental Report was designed for TAPP (Trial of Anemia Prevention in Preschoolers) that took place in Tanzania from 1996 to 1997. The scale that will be used for this project was reduced from a larger group of items using factor analysis and is designed to mark the onset of bipedal locomotion. It consists of a list of 29 items, 6 of which also must be demonstrated by the child. The DIMAG observers will administer this scale to mothers as a part of the DIMAG Home Interview Form (DHIF) every three months for the duration of the study.

The observation will take place in the target children’s home every three months. Because Nepali children are most active in the morning and demonstrate the greatest range of activities during this time (Gittelsohn, personal communication, 2001), the 15 observers will observe motor activity and social-behavioral interaction for a period of three hours beginning at 9:30 a.m. Both time- and event-sampling methods will be employed. Data on motor development will be gathered from the motor activities that are checked. These activities are delineated in the Activity Section.

**2.3 Social development**

Social Development will be assessed using two methods: the Parental Report, and observation. These measures will be utilized at the same time-points as the motor development measures: every 3-months. The Parental Report has been revised from the TAPP form to include additional items from the Minnesota Infant Development Index. The revised scale is designed to measure language acquisition in a greater range of ages (4- to 28-months) than the original TAPP form.

The observation will focus on social behavior. In addition to recording the target children’s motor activities, the fieldworkers will observe direct and non-direct social behaviors. Every 15 seconds the fieldworkers will be cued by a beep to mark boxes on the Visor screen that correspond to the key social behaviors that they witnessed in the previous 15 seconds: object manipulation, eating and drinking, social play, social gesture, affective display, negative affective display, social smile, breastfeeding, carrying in arms, fussing, vocalization initiation or response to caretaker and/or peer, and non-social vocalization with or without object.

## **2.4 Cognitive development**

Cognitive development will be assessed primarily through two developmental instruments that will be administered when the infants are 39- and 52-weeks of age: the Fagan Test of Infant Intelligence and the A-not-B Test. Both of these tests are measures of information processing, designed to measure the infants’ ability to make sense of their surrounding environment and to target different areas of the central nervous system, revealing different cognitive skills. The Fagan Test of Infant Intelligence records percent time spent looking at novel stimuli (human faces) in a paired-comparison paradigm. The A-not-B Delayed Response Task focuses on the infant’s ability to search. During this test, the child is asked to find the toy that has been hidden in one of two wells that has been selected according to a predetermined sequence based on the child’s performance. We will measure all infants who are 9- and 12-months of age. The clinic staff will begin testing at baseline and will finish 9-months after the last child has been enrolled, when the youngest infants turn 12-months. These two cognitive tests will be performed as a part of Clinic B.

**2.5 Activity**

Activity will be measured every three months using a sturdy, watch-like device called an Actiwatch that will be strapped on to the left ankle of each child enrolled in the project. The Actiwatch will be left on the child for a period of 22-hours. One of the observers will place it on the child the day before the observation is scheduled to occur (4 p.m. the day before) and will remove it after the observation (from around 12 p.m. to 2 p.m. the following day). Actiwatch data will be collected in 15-second intervals and will inform the investigators about the child’s activity throughout the period of measurement. The time interval will be synchronized with the Visor to reinforce the observational data.

Activity will also be measured with the observation. This part of the observation is event-sampled. Once fieldworkers witness a key motor activity they will check the corresponding box on a Palm Pilot screen. Key motor activities include: being carried, lying, sitting with and without support, balancing on all four’s, squatting, creeping, crawling, scooting, kneeling, standing with and without support, walking with and without support, climbing, jumping, and running.

**2.6 Temperament**

Temperament will be measured using a section of the Infant Characteristics Questionnaire (Bates, 1979), which assesses a child’s level of fussiness and irritability. The observers will administer 12 questions to the primary caretaker every three months as a part of the DIMAG Home Interview Form (DHIF).

**2.7 Sleep duration**

Infant sleep patterns will be measured using questions that were developed for the TAPP study. These questions have been included in the DIMAG Home Interview Form and will be asked every three months when the observers visit the study infants’ houses.

**2.8 Micronutrient status**

Because of cultural barriers to drawing blood from Nepali infants and the difficulties we have encountered finding veins in healthy infants for venous blood draws, we have limited our micronutrient status assessments to those that can be measured using a heel-prick. These include two measures of iron status: hemoglobin (Hb) and zinc protoporphyrin (ZPP). One of three medical assistants experienced in infant phlebotomy will collect 2 drops of whole blood from the infant through heel-prick at baseline and graduation from the study. These drops will be analyzed using a HemoCue machine to measure hemoglobin and a flurometer to measure zinc protoporphyrin.

## **2.9 Dietary pattern**

Dietary pattern will be assessed every three months through questions about infant feeding and appetite that are a part of the DIMAG Home Interview Form (DHIF).

The observers will ask the primary caretakers for frequencies of foods fed to their infants in order to determine the stages of complementary feeding. The items chosen for the food frequency were an outgrowth of the NNIPS-2 data that were gathered to describe infant feeding in Sarlahi District during the mid-1990s. The foods include milk (human and non-human), several different types of carbohydrates (rice, corn/flour porridge, bread, biscuits), vegetables, fruits, and protein sources (lentils, eggs, meat).

# **2.10 Growth**

All of the growth indices will be measured in the clinic at baseline and graduation from the study by the project anthropometrist. These include recumbent length, weight, head circumference, mid-upper arm circumference (MUAC), and upper-arm skin fold thickness. In addition, head circumference will be measured at 3-, 6-, and 9-months.

## **2.11 Morbidity**

The observers will assess morbidity at the time of the Actiwatch attachment, the day before the observation, on the day of the observation, and prior to each of the clinic visits. The questions ask the primary caretaker to report whether the target child has had the following symptoms‘ today’ (the past 24 hours) or in the past five days: fever, cough, difficult/rapid breathing, or watery stool. Watery stool is checked if the child has had 4 or more watery stools in a single day. If the child has fever, difficult or rapid breathing, and/or watery stools ‘today’, the observation and Clinic B visits will be rescheduled. The visit will proceed if the child only has a cough. Children who presented with morbidity symptoms in the past 24-hours will not be excluded from the Clinic A visit. As a part of the Clinic A protocol, the clinic staff will measure temperature of all the children who present for Clinic A.

**3. DIMAG FIELD VISITS**

Two types of field visits will occur during DIMAG. The first is part of the larger NNIPS-4 trial and involves the WDs. The second is exclusively a DIMAG activity during which the DIMAG observers will visit the DIMAG infants’ homes every three months to attach and remove the Actiwatch, conduct the observation, and interview the primary caretaker about the infant’s dietary pattern, motor and language development, temperament, and sleep patterns.

**3.1 Motor Milestones Worksheet DIMAG (MMWD)**

As a part of their bi-weekly home visit activities, the WDs will ask the primary caretakers of all of the children enrolled in NNIPS-4 in Ishwarpur if their child has achieved one of the 14 motor milestones listed on the Motor Milestones Worksheet DIMAG. Each Friday the WDs will record 00 to indicate that a milestone or the number of the appropriate motor milestone (01-14) on the Child Dosing Roster- DIMAG (CDR-D) form. They will put a dash (--) on the form once the 14^th^ milestone has been achieved and the form has been sent to Kathmandu. When the caregiver reports that a milestone has been achieved, the WD will record the appropriate milestone on to the form. Should the WD observe that the child has not completed the milestone that the mother reported, the WD will not change the code, as this is meant to be a parental report. At the end of each month, the CDR-D will be taken to Kathmandu for data analysis. The MMWDs are sent to Kathmandu once they are complete.

The data collection will begin prior to the start of DIMAG when the NNIPS-4 Batch 4 begins on December 10, 2001. The WDs will begin by asking whether the target child has achieved each of the 14 codes listed on the MMWD. For every code achieved, the WD will draw a line through the boxes on the MMWD. During the first visit to the home, they will ask about milestones that have been achieved until the caretaker tells them that the child has not achieved two milestones in a row. After two no answers, the WDs will stop reading the list of milestones and will return to the last milestone completed. They will ask the caretaker whether this milestone was achieved in the past 7 days. If it was, the WD will record the NNIPS week number on the MMWD and will mark the number of the milestone into the appropriate boxes on the CDR-D Form. If the milestone was not completed in the past 7 days, the WD will make sure that a line has been drawn through the boxes on the MMWD and will record a 00 on to the CDR-D Form.

When the WD returns to the house the following week, she will resume her questioning by asking the caretaker whether the child has achieved the milestone following the one through which she drew the line the previous week. For example, if the child achieved Pulls to Sit (code 01) the previous week, the WD will begin inquiring about Creep 1 (code 02). As she did the first week, she will stop asking about the milestones when the caretaker has indicated that the child has not completed two consecutive milestones (i.e. 2 no answers after a yes). In some cases, a child will either skip a milestone or will achieve milestones in a different order than they are listed on the questionnaire. If this happens, the WD will record the NNIPS week number on to the MMWD and the corresponding code on to the CDR-D, and will remember to ask the caretaker about the code that was skipped until the two milestones following the one in question have been completed. For example, if a child completes Pulls to Sit (code 01) and then accomplishes Sit 1 (code 03), but still hasn’t performed Creep 1 (code 02), the WD will record the week number on to the MMWD that Sit 1 was performed and the Sit 1 code (03) on to the CDR-D. When she returns to the house the next week, she will ask about Creep 1 (code 02) and Sit 2 (code 04). Should Sit 2 and All 4’s (code 05) be completed, while Creep 1 remains blank, the WD may stop asking about Creep 1. It is important that the WD always ask about the two milestones following the last milestone completed.

It is possible that two milestones will be completed during the same week. We expect this condition to be rare; however, in the event that it occurs, the WD will record the same NNIPS week on the MMWD and the greater of the two codes on to the CDR-D. For example, if both Sit 2 (code 04) and All 4’s (code 05) are completed during NNIPS week number 60, the WD will write 060 into the boxes on the MMWD that correspond with codes 04 and 05, and will record 05 into the boxes on the CDR-D. Because there is no mechanism for entering the same code into the data program, the WD will report these cases to the Ishwarpur TLI during the weekly meeting. She will give the TLI the NNIPS number of the child and will tell him which two codes were achieved during the same week. The TLI, in turn, will report these cases to Emily. The WD will also report unusual cases, such as two blank milestones being followed by two completed ones, to the TLI for him to record the information in a note at the bottom of the MMWD form.

The codes that will be entered into the boxes on the CDR-D follow: 00 = no new milestone achieved; 01 = Pulls to sit; 02 = Creep 1; 03 = Sit 1; 04 = Sit 2; 05 = All 4’s; 06 = Creep 2; 07 = Crawl; 08 = Stand 1; 09 = Walk 1; 10 = Stand 2; 11 = Walk 2; 12 = Run; 13 = Jump; 14 = Stand on 1 foot; 66 = Refused; and 99 = Don’t know.

**3.2 DIMAG Child Tracking Log (DCTL) and enrollment**

The DCTL will assist us in tracking the status of each of the DIMAG infants and will contain identifying data on each child that will make it easy for both the observers and clinic staff to complete Section A for each of the forms. The forms were printed from a merge file that was created in the data center in Kathmandu. This file contains NNIPS-4 infants that were greater than 28 days and less than 510 days on January 7, 2002, the official start date for DIMAG. Infants were included in the DIMAG sample if a) if they were >120 days and <510 days on the first day of the first NNIPS week or >120 days and <510 days on the last day of the last NNIPS week that we listed for each of the wards, b) the observers were able to find them at home between the first date listed for each ward and up to two weeks after the last date listed, and c) the child’s family did not move permanently from one location to another, within the ward, between wards, or even to another VDC during the enrollment period. The latter is a NNIPS-4 rule that was designed to prevent contamination between supplement groups. We tried to visit each child three times before we coded the child’s vital status in DIMAG as “not met” or “2”. For this first visit, a “not met” status warranted removal from the DIMAG master list.

The list of eligible DIMAG kids included 639 names. From this number we subtracted 5 kids with a vital status of “7” (permanent move) during Round 1; 1 kid who did not receive a vital status of “7” until Round 2 but our field workers knew had moved before the data was taken to and entered in Kathmandu; 1 kid who our fieldworkers were told had moved permanently but is still considered by the data center in Kathmandu to have a vital status of “2” (not met); 2 kids with a vital status of “8” (dead); 5 kids whose parents refused to allow them to participate in DIMAG; 8 kids who were considered to be too old when we reached their homes as we moved through their wards; 39 who we were not able to meet when we were working in their respective wards; and 10 who were not identified by the data center in Kathmandu as being eligible until after we moved through their wards. Five of the ten kids mentioned as being identified too late were enrolled from the NNIPS-4 Baseline New Family Enrollment Form shortly before the start of DIMAG. Their data had not been entered into the database when the list of kids eligible to be enrolled in DIMAG was created. We speculate that the other five were not considered eligible until the WDs and TLIs revisited their houses after enrollment and figured out that the people on their list had incorrect birth dates and were children instead of adults (i.e. were less than 2 instead of greater than 20). The number of kids eventually enrolled in DIMAG includes 568 kids.

The dates that we worked in each ward for Round 1 follow:

Ward 1: NNIPS Weeks 53-56 (4 Jan 2002 – 31 Jan 2002)

Ward 2: NNIPS Weeks 55-58 (18 Jan 2002 – 14 Feb 2002)

Ward 3: NNIPS Weeks 57-60 (1 Feb 2002 – 28 Feb 2002)

Ward 4: NNIPS Weeks 58-61 (8 Feb 2002 – 7 Mar 2002)

Ward 5: NNIPS Weeks 58-59 (8 Feb 2002 – 21 Feb 2002)

Ward 6: NNIPS Weeks 59-62 (15 Feb 2002 – 14 Mar 2002)

Ward 7: NNIPS Weeks 61-62 (1 Mar 2002 – 14 Mar 2002)

Ward 8: NNIPS Weeks 61-62 (1 Mar 2002 – 14 Mar 2002)

Ward 9: NNIPS Weeks 62-63 (8 Mar 2002 – 21 Mar 2002)

We allowed 2 weeks after the last date recorded for DIMAG kids to return home from maiti or otherwise change their vital status from “2” to “1”. These kids had to be between 120 and 510 days during the dates listed for each of the wards for us to continue to pursue them. For example, we would have allowed an observation done on a child in Ward 6 during NNIPS week 64 to be included in the data, as long as the child was between 120 and 510 days between NNIPS weeks 59 and 62.

Although the father’s, mother’s, and child’s names are listed on the form, it is the father’s name that is most useful for finding the child in the field. Both the father’s and child’s names are recorded on to each of the scheduling documents and are used by both the observers and clinic staff when searching for the DIMAG children in the field.

**3.3 DIMAG Daily Home Observation Schedule (DDHOS)**

Once the DCTL forms are complete, the staff will fill out the four scheduling forms. The DDHOS will be used to schedule the first home visits for DIMAG where the observers will request consent. About 12 or 13 observations will be scheduled every day, depending on how many observers are present to work. Eligible subjects will be grouped according to the ward and sector in which they live. By grouping the subjects, the observers will be able to travel together to do their observations each day and the project vehicle will be able to travel to a single location to pick up the caretakers and infants for the Clinic A visits. DIMAG will begin in Ward 1, Sector 1. Ward 2 will be the next ward and Ward 9 the last. The observers will move through each ward consecutively, visiting all the eligible children. For the first round, the observers will have two weeks to return to observe children who were at maiti, another location, or were sick when they initially tried to visit the target child before the work in the specific ward and sector is considered complete. For each of the other rounds, the child must be seen within two months of their first dose date.

The DDHOS initially will identify which subjects need to be observed. On the first day that DIMAG begins, 12 observers will go to the field to request consent from the infants’ caretakers. They will read the DIMAG Consent Form (DCF) and will ask the primary caretaker whether she agrees. If consent is given, the observers will proceed by completing Section B of the DHOS. If the child is determined to be healthy, the observer will attach an Actiwatch to the left ankle of each healthy child and will request that the Actiwatch not be removed. It is a sturdy device that can get wet. Before leaving the observer will tell the caretakers that he/she will return to the home the next day around 9:30 a.m. to conduct an observation that will last 3 hours. He/she is interested in watching the child participate in everyday activities, so it will not be necessary for the family to make any special preparations for this visit.

The observer will return to the office and record on the DDHOS the name and identifying information of the child that he/she will visit the next day. The purpose of this is to record which of the study infants are being observed, so their names can be transferred later to the DIMAG Daily Clinic Schedule (DDCS) and the DIMAG Daily Home Interview Schedule (DDHIS). Also, by recording the infant’s identifying information (address, father’s name, child’s NNIPS number, name, and age) and by putting their worker ID, Shishir and Emily will know where the observers are in the field should they need to be contacted. Once the visit has been completed, the observer will check the observation complete box and record the observation duration time. If the child that was observed was wearing an Actiwatch, the observer will also check the Actiwatch box. If there was no Actiwatch, the observer will put a dash in this box. On the comments section of this form the observer will record any problems that he/she encountered to alert the other observers to what happened, information that might be useful when another observer visits the house to re-do the observation.

On Friday afternoons the Visor and Actiwatch data are downloaded as usual; however, the observers do not return to the field to attach the Actiwatches. Instead, the person who downloads the data prepares the Actiwatches to be attached on Sunday. Before leaving on Friday, Shishir compiles a list of 20 kids that need observations and puts the Actiwatches on top of this list for the designated TLI to find when he works on Sunday. This TLI works from Sunday to Thursday instead of Monday to Friday. The TLI goes to the field and attaches Actiwatches to the children he finds at home who are determined to be healthy per our morbidity criteria. Once he places an Actiwatch on a child, he puts a check next to the child’s identifying information so Shishir knows where to send the observers on Monday morning. The TLI then returns the form to the observer room in the Pink Palace for Shishir to find the next day.

During subsequent rounds, Shishir and Emily will use the first dose date scheduling tool that was created based on the date that each child received his/her first dose of the supplement to provide assistance in scheduling observations (see Appendix). A two-month window has been created around this date for each round to indicate when the child needs to be scheduled, one month before and after the three-month target date. All children will be scheduled within the window. If they are at maiti or are unable to be observed during the particular round for another reason, their visit will be coded as missed (“2”) on the appropriate forms and the observer will not try to see them until the following round. Only under rare circumstances (children were left off the list because the data center in Kathmandu had not entered their first dose date when the list was created) will DIMAG children be seen outside the window. After returning from the field, the observers either recorded the child’s information in the DDHOS or put a note next to the child’s name on this first dose date scheduling tool to indicate whether the child was sick, at maiti, away from the house for a period of time, refused, permanently moved, or died. Shishir used these notes to help him determine which children needed to be observed during each of the rounds on any given day.

Beginning with Round 2, another scheduling tool was created to assist Shishir in determining which observers should observe which children. This tool was designed to prevent observers from observing the same children during multiple rounds. Based on Round 1 data, a list of eligible observers was recorded next to each DIMAG child’s address and NNIPS number. Shishir crossed off the observer’s worker ID after the observer conducted a new observation. This task became complicated as time passed. Prior to sending the observers to attach the Actiwatches, Shishir figured out which observers were eligible to do which observations. He grouped the eligible observers together and sent them out in a group to make contact with the children’s families. Each day the groups were different, depending on which child needed to be observed.

**3.4 DIMAG Home Observation Form (DHOF)**

On the second day of DIMAG, the observers will return to the houses that they visited the previous day to do the observation. They will have already completed most of Section A and all of Section B prior to reaching the house. Before proceeding they will need to fill in the remaining variables: the Respondent’s Name, the Respondent’s Relationship to Child, and the Form Status. They will then read the morbidity questions in Section B again to make sure that the child has not gotten sick since the visit the previous day. As with the other forms, the observer will reschedule the visit if the child has had a fever, difficult or rapid breathing, or watery stool (4 or more) in the previous 24-hours. The observers will then begin the observation

The observers will complete Section C after they have finished the observation. The first series of questions address the Actiwatch. Was one present during the observation? If so, was it removed between the time that it was placed on the child and the time that the observer came to do the interview? If so, when was it removed? Was it replaced? If so, when was it replaced? If the Actiwatch was removed for two periods, the longer of the two will be recorded on the form (i.e. If the Actiwatch is first removed for 10 minutes and then 1 hour, the hour period will be recorded.). If the Actiwatch was removed twice, but only replaced for a short period of time between taking it off the first time and attaching it again, the first removal time and the final replacement time will be recorded. Observations that were not completed (i.e. were not 180 minutes), but were greater than 120 minutes do not need to be rescheduled. Only observations that were less than 120 minutes (2 hours) will be rescheduled. Only primary caretakers and significant peers will be counted as being present during the observation. These people might include people to whom the child initiated or responded to a vocalization. The observer may need to inquire about the caretakers’ and peers’ relationship to the target child, as it might not be evident. Because being inside often restricts the child’s activity, it will be important for the observer to estimate what percentage of the observation was spent inside. Also, we are interested in knowing where the observation took place. An observation during which the caretaker and child traveled to another destination potentially could be a different observation than one that occurred exclusively in the child’s home. Weather in Sarlahi can be extreme. It is possible that it might have an effect on the child’s behavior. Children in this region often spend more time inside during rainy days and time sleeping during very hot days. For this reason, the observers will be asked to note the weather conditions on the day of the observation.

**3.5 Conducting the observation**

The observer will arrive at the house and greet the family. It most cases it will be necessary for the observer to introduce himself/herself. It is possible that this will be the first time that the observer has met the family, since another observer may have attached the Actiwatch the previous day. Before beginning the observation, the observer will talk with the family to give the family some time to ask questions and the child some time to feel comfortable with this new person’s presence. Immediately prior to and after the observation, observers observing children with Actiwatches will push the button on the Actiwatch to mark the duration of the observation.

The observation will be conducted for a period of three hours. If the observer needs to change the batteries or needs a break, he/she will push the pause button to stop the observation until he/she is ready to proceed. If the infant falls asleep during the observation, the observer will push the sleep button. When either the pause or sleep buttons are pressed, the time counter will stop until the buttons are pressed again. For example, should the observer take a five-minute break and observe an infant that sleeps for 40 minutes, the box indicating that the observation is complete will not pop up until 3 hours and 45 minutes have passed. A mandatory five-minute break has been implemented after each hour of observation to give the observers an opportunity to rest.

The observers will observe the target child and the child’s natural activity and social behavior. They will record the data that they observe into their individual Handspring Visors that have been provided for the observation, and which have been fitted with a unique software package called CAOS, that data programmer, Darrell Mast, designed specifically for this task. The observers will select a natural activity from a list of 20 activities on a screen that is separate from the social behavior screen. Once the observation begins, the observers must select an activity before proceeding with the observation. During the observation they will only code natural activities that the child has performed for the observer’s count of 3-seconds. Social behaviors and vocalizations are also recorded when they occur; however, the screen is not cleared (i.e. the data are stored) for 15 seconds. Should two instances of the same activity occur in the same 15-second interval, only one will be recorded (i.e. the child smiles twice, the data will indicate that a smile occurred during that 15-second interval). There are nine social behaviors and 6 vocalizations that are recorded. These and the list of natural activities can be found in the appendix.

The observers’ job is to record the activity that occurs within the family setting. They will go to the house, but will follow the child (and in most cases a caretaker) to another location, should the child decide to leave or be taken from the house. The observers have been instructed to remove themselves from the action occurring around them, as well as they can. Although we anticipate some reactivity, we expect that this reactivity will diminish as the observation progresses and after the first observation, once the family is familiar with the observers’ job. The observers will not initiate a conversation with the family during the observation; however, they may respond if someone tries to talk to them. If the person in question has a lot to say or many questions to ask, the observer will push the pause button and will talk to the person. During the formative research stage, we found that many family members and neighbors return from the field during the late morning hours, well after the observation has started. Those that are unfamiliar with the nature of the observation tend to have a lot of questions for the observer. After about five minutes we expect the questioner to walk away, or at least stop asking the questions, at which time the observation can continue.

Once the observation is complete, the observers will remove the Actiwatch and return to the office. They will place their Actiwatches on top of their Visors (so the person downloading the data knows that the two go together) on the table next to the computer for Shishir, Emily, or one of the observers who has been trained to use the computer to download the data into the CAOS base-station. At 3 p.m. they will assemble to collect the Actiwatches they will fasten on the child they will observe the next day. Each observer will write down several names of DIMAG kids that are due to be scheduled before they get into the office vehicle and are transported to the Ward and Sector in which they plan to do the observation the next day. They may have to visit several houses before they find one in which the child is both present and healthy. Once they make contact, they will fasten the Actiwatch, if they have one, and will return to the vehicle. Because there are only 11 Actiwatches, there will be days that some observers will not have a watch. Nevertheless, all the observers who plan on doing observations the next day will make contact with the family the afternoon prior to the visit. When they return to the office, they will record in the DHOF the name of the child they made contact with and will indicate whether they attached an Actiwatch.

In the morning, the observers will either report to the office or go directly to the house where they are scheduled to conduct the observation. The choice is the observers; however, they must synchronize the clock on their individual Visor with the one on the CAOS base-station before they depart from the office, either the afternoon before or morning of the observation. They will arrive at the child’s house around 9:30 a.m. and will have 5 hours to complete the observation. During the hot months, some observers may elect to start the observation earlier. If the child falls asleep, the observers will stay at the house until 2:30 p.m., at which time they will return to the office. If the actual time that the observers are able to observe the target child is less than 2 hours, the observation will be considered incomplete and will be redone on another day.

**3.6 Downloading data into the base station**

When the observers return to the office after conducting the observation, they will place the Visors on to the table next to the computer. So the person who is downloading data into the computer knows which Actiwatch was attached to which DIMAG child, the Actiwatch will be placed on top of the corresponding Visor. The person downloading the data into the computer will pick up the pair and will place the Visor into the cradle and the Actiwatch on to the Actiwatch reader.

The process of downloading the data begins with attaching both the Actiwatch Reader and Hot sync cradle to the computer using the USB and serial ports respectively. Of the two computers used for DIMAG (caosbase1 and caosbase2), caosbase2 is the primary computer into which the data are downloaded and from which the data are transferred. Shishir or Kesab, one of the observers trained to use the computer, will turn on the computer and logon. The logon id is the same as the computer name: caosbase2 or caosbase1. The password for both computers is “dimag.” Once the logon process is complete, the designated “downloaded” will open both the CAOSBase and Actiwatch Rhythm software by clicking twice on each of the icons placed on the desktop. As was mentioned above, he will place respectively the Visor and Actiwatch in the cradle and on the reader.

The process of placing the Visor in the cradle is relatively straightforward. Periodically, when the reader is unable to read data from the Visor, it may be necessary to clean the metal strips on the bottom of the Visor with alcohol prior to placing it in the cradle. Otherwise, the Visor will be placed vertically in the cradle with the screen facing the front and the metal strips on the bottom touching the metal strips on the reader. The Actiwatch requires a little more attention when placing it on the reader. The download person must look for the small, engraved dot located near one of the four screws, match this dot with the picture on the reader, place the Actiwatch on the reader, and move it around until the green light is illuminated. This light means the data from the Actiwatch are ready to be downloaded.

Darrell Mast, the CAOSBase software programmer, designed the CAOSBase program to create a folder in which both the Visor and Actiwatch data will be stored on a daily basis. Because the Hot sync process signals the computer to create the folder titled with the date, the Visor data must be downloaded before the Actiwatch data. To download the data from the Visor, the download person will take the following steps:

1. Click on the “Palm” icon in the CAOSBase program and select “Download Palm Data.”
2. When the “Please select a Palm Device” screen appears, select the appropriate Visor number from the list. Refer to the sticker affixed to the back of the Visor for the number of the individual Visor.
3. Press the Hot sync button on the front of the cradle.
4. When the cradle makes a noise signaling the data have been downloaded, press “Ok” in the “Download Palm Data” screen.
5. Enter the Observer ID, Observer Count Total Length min, Child ID, and Data Points recorded on the screen in the DIMAG Daily CAOS Log (DDCL).
6. Press “Ok” two more times.
7. Press the Hot sync button again to load a blank copy of the program on to the Visor.
8. Press “Complete” in the “Download Palm Data” screen.
9. Remove the Visor from the cradle.

Once the data from the Visor have been downloaded, the Actiwatch data may be downloaded taking the following steps:

1. Click on “Reader” in the Actiwatch-Rhythm program and select “Read.”
2. When the “Preparing to communicate with Actiwatch” message appears, press “Ok.”
3. “Reading Actiwatch setup” message will appear. Wait for the data to download. Record the Actiwatch serial number in the DDCL.
4. When the “Actiwatch Read complete” box appears and the program asks whether to save the data, click “Yes.”
5. When the next screen appears, click on the blank in “Save in” and select the folder with today’s date from the list. The program will automatically create a folder during the Visor download process. This folder will be named with an 8-digit number: four digits for the year followed by two for the month and two for the day.
6. From the same screen, change the file name from the Actiwatch serial number to the child’s NNIPS number followed by the date. This number will have 14 numbers: six for the NNIPS number, four for the year, two for the month, and two for the day. Refer to the DDCL for the child’s NNIPS number that was recorded during the Visor download process. Press “Save.”

After the downloading process, the person responsible for downloading the data must write to the Actiwatch as a means of preparing it to collect data once again. The steps for writing to the Actiwatch follow:

1. Click on “Reader” in the Actiwatch-Rhythm program and select “Write.”
2. When the message “Are you sure you want to write to the Actiwatch” appears, press “Yes.”
3. When the message “Preparing to communicate with the Actiwatch” appears, press “Okay.”
4. When the message “Actiwatch setup information loaded” appears, press “Ok.”
5. When the “Actiwatch Setup” screen appears, check to see that the “User ID” box has the Actiwatch serial number; the “Start Date” is today’s date, unless the Actiwatch is not being placed on another child that day; the “Start Time” is set for 16:00 hours, and the “Epoch Length” is set for 00.25. Disregard the information under “Sex” and “Age.” Data from these variables will not be viewed. If the Actiwatch is not being sent to the field until a later date, either write the appropriate date to the Actiwatch (on Fridays the download person must write the date for Sunday) or set the Actiwatch aside for Shishir or Kesab to write to it another day. Once the data is correct, press “Send.”
6. When the “No User ID was entered, do you want to continue with the old ID?” message appears, press “Yes.”
7. When the “During the following configuration process, DO NOT REMOVE Actiwatch from reader.” Appears, press “Ok.”
8. When the “Actiwatch configured and ready” message appears, press “Ok.”
9. Remove Actiwatch from reader. Clean and send to the field or set aside for use another day.

Each of the steps must be repeated for each Visor/Actiwatch pair. Because there are not enough Actiwatches for each of the observers to have one, some of the Visors will not have Actiwatches on top of them. Once all the data have been downloaded, the Visor cradle and Actiwatch reader may be unplugged from the computer and stored in the closet to avoid being exposed further to dust.

- 1. **DIMAG Daily CAOS Log (DDCL)**

As was mentioned above, identifying information from the Visor and Actiwatch data are recorded in the DDCL: which child was observed and for how long, whether the observation was less than 2 hours and needs to be rescheduled, and whether and which Actiwatch was attached to the specific child.

The form requires that the person downloading the data from the Visor and Actiwatch record the NNIPS week and day and sign his initials. The Palm ID is marked on the back of each palm with a sticker. The CAOS program was designed to display the observer ID, child ID (NNIPS Number), length of the observation, data points, and number of observations. While in theory each observer only performs one observation on a given day, it is possible that the observer will exit the program for any number of reasons (i.e. changing batteries, the observer presses the pause or sleep button with force, the observer presses the pause or sleep button twice by mistake) during the course of the observation and will have to reenter the program to proceed with the observation. When more than one observation is noted, it is a sign to the person responsible for cleaning the data (Darrell) that the multiple observations must be linked before the data are analyzed. The Actiwatch serial number only is displayed in the bottom right corner of the Actiwatch screen. It is first displayed while the data from the Actiwatch are being read. The form also has a “Notes” section for the person downloading the data to note anything unusual that happened during the observation or course of the download process. While these notes are sometimes recorded in English, they are usually written in Nepali.

There are boxes at the bottom of the form serve as reminders for the person downloading the data to save the data or compact and transfer it depending on the day of the week. Only the first three boxes are used: Daily Backup (to Floppy Disk), Bi-Weekly Backup (Base Station), and Compact Database. This form is stored in a binder in the Ishwarpur office. It is not entered.

- 1. **Saving, compacting, and transferring data**

On a daily basis DIMAG data are saved to a disk or are compacted and transferred to caosbase1. Periodically (no longer than one-month intervals), the CAOSBase database and data files are transferred to Sagarmatha, located in the Data Center in Kathmandu. Although the activities of the week can vary, depending on external events, such as the political situation or a festival, the data files are usually saved to a floppy disk on Mondays and Wednesdays and the database and data files are compacted and transferred to caosbase1 on Tuesdays, Thursdays, and Fridays.

The instructions for saving the data files follow.

1. Click twice on the “My Computer” desktop icon.
2. Click twice to open “3 ½ Floppy (A).” Delete the old data file stored on the disk. Move the window aside and repeat instruction #1.
3. Click twice to open “Local Disk (C:).”
4. Click twice to open “CAOS.”
5. Click twice to open “Data.”
6. Find the file that will be saved. It has automatically been titled with the date that the data were collected: four-digits for the year, two for the month, and two for the day. Right click on this folder. Click once on “Send To” and move the curser to 3 1/2 Floppy (A). Click.
7. Alternatively, it is possible to save the data file by dragging it from the “Data” folder to screen that was moved aside in instruction #2, Floppy disk (A). After saving using either of the two methods, check to see that the designated folder is present in the Floppy (A) window.
8. Close all of the windows and Shut Down the computer.

The data are compacted prior to transferring from caosbase2 to caosbase1. The instructions for compacting the data follow.

1. Find the “Start” button at the bottom left corner of the computer screen. Click. Proceed to “Programs” and click. Find “Microsoft Access” from the list of programs and double click.
2. Select “Open an existing file” from the list of three options. Find “C:\CAOS\CAOSBaseDB” from the list of files. Click “OK.”
3. The CAOSBaseDB: Database screen will appear. Make sure PobsInstance is highlighted. Proceed to “Tools” at the top of the screen. Click once. Find “Database Utilities” in the dropdown list. Click once. Select “Compact and Repair Database…” Click once. The timer will appear on the screen and “Ready” can be seen in the bottom left corner of the program. “Compacting” will appear next. Wait until the program indicates that the compact process is finished. When the CAOSBaseDB: Database screen reappears, exit Microsoft Access.

The instructions for transferring the data from caosbase2 to caosbase1 follow.

1. Shut down both computers. Attach an end of the networking cable to each of them. Turn on the computers and logon on to both.
2. Find the “My Network Places” icon on the caosbase2 desktop and click twice.
3. Find and open “CAOS on Caos1” by clicking twice on it. Move the box/screen to the side.
4. Open “My Computer.”
5. Follow the same directions above saving data to a floppy disk. Open “Local Disk (C:),” open “CAOS,” and open “Data” by clicking on each of these folders.
6. Copy all data files that have not been copied to caosbase2. Drag these files from the data file to the “CAOS on Caos1” screen that was opened in step #2. Exit the “Data” file.
7. Find “CAOSBaseDB.” Drag this folder to “CAOS on Caos1.”
8. Before shutting down both computers, check to see that the data were actually transferred to caosbase1 by looking for the files in the same place that they were sent from caosbase2: “My Computer,” “C:,” and “CAOS.”

The instructions for transferring the data from caosbase2 (or Emily’s computer) to Sagarmatha have changed throughout the course of DIMAG due to changes in the data center. Presently, both the data files and CAOSBaseDB are transferred into the CAOS folder in Sagarmatha using the network cable. The new data files are copied into the “Data” folder, while the CAOSBaseDB is renamed with the date that the program was saved (i.e. CAOSBaseDB_20030204), so as not to confuse it with previous copies of the program. This transfer is done in the same way that the data are transferred from caosbase2 to caosbase1.

- 1. **Viewing data for the standardization exercises**

The instructions for viewing the standardization reports follow.

1. Enter CAOSBase program.

2. Click Report button

3. Select “Observation data” pull-down option.

1. Select Child ID and date. By default the present date will appear. Because of this the date will have to be changed in the event that the person is viewing the report after the date that the work was done.
2. Click on Process Count
3. Click View
4. When a gold standard is used, select the Worker ID of the person who will be used as a gold standard. When each observer is compared against each of the others, select the Worker ID of the first person who will be compared to the others. Click on the “New Weighted Kappa” button.
5. Select the second observer’s ID whose record will be compared to the first. Again select “New Weighted Kappa” button.
6. Wait for the report to be displayed.

10. Repeat for each of the observers.

**3.10 Cleaning the Actiwatches**

Once the Actiwatch data have been downloaded into the base station, the Actiwatches must be cleaned before they are taken out to the field and attached to another child. The bands will be removed, washed separately in a soap and water solution, and laid out to dry. The watches will be scrubbed with a small nailbrush to remove the mud and excrement that collects daily around the screws and marker button on the top of the watchcase. The watches will be held under a steady stream of water to remove the soap. The bands will be threaded into the watches once both have dried. Alcohol may be used instead of soap; however, the soap and water combination appears to work better for cleaning the dirt.

### 3.11 DIMAG Daily Home Interview Schedule (DDHIS)

Beginning on the third day of DIMAG, 2 observers will visit the homes that were observed the previous day to administer the DHIF. Afterwards, interviews will be scheduled on Fridays or when there is a surplus of observers. Theoretically, the families that were observed will be interviewed in the same week; however, we expect that the primary caretaker of the household occasionally will be unavailable and the interview will have to be scheduled for a later date. At baseline and the end of the visit, when we are bringing children into the office for Clinic A, the interviews that remain after not being able to connect with the families at home can be done in the office by Shishir or one of the observers in lieu of doing an observation in the field or after he/she returns, depending on the situation.

During Round 1 the observers recorded the identifying information of the families that required observations on the DIMAG Daily Home Interview Schedule (DDHIS). This information included the following from the DCTL: the family’s address (ward, sector, and household); the father’s name, the child’s NNIPS number, name, and age; the worker ID; and whether the form has been completed. Beginning with Round 2 and the introduction of boxes in the motor module of the DIMAG Home Interview Form that asked the observers to record whether they witnessed the child performing any of the six activities (walk forwards in a straight line, stand on one foot, walk backward in a straight line, walk on tiptoes, skip, jump twenty times on one foot), the form was only used by Shishir to record completed interviews. The observers were asked to fill out the identifying information on the front of the DHIF and record whether they saw the behaviors mentioned above during the course of the observation. Afterwards, they put the forms in the designated cubicle to alert Shishir that an interview needed to be conducted.

**3.12 DIMAG Home Interview Form (DHIF)**

Depending on how fast we are able to move through the wards and sectors, periodically several observers will visit the houses where the most recent observations have been conducted to administer the DHIF. Each observer will conduct the interview with the mother of the target child, if it is possible. If the mother is not home, the observer will determine whether there is another primary caretaker that has been responsible for the child’s care (i.e. grandmother, aunt, or sometimes the father) that would be able to answer the questions. The observer will move to another house if no one is able to answer the questions at that time and another observer will return at a later date. Each interview will take about 20 minutes.

The observer will use the Child Tracking Form as a reference to fill in the identifying information in Section A before traveling to the child’s house. At the house, the observer will only have to fill in the Respondent’s Name, the Respondent’s Relationship to Child, and the Form Status to complete Section A. As was mentioned above, beginning with Round 2 the observer who conducted the observation completed this information prior to the interview. From Round 2 it was necessary for the observer to record only his or her name and worker ID in the designated place. For Section B the observer will read the question “In the past 7 days, has your child eaten ……” and will ask the respondent to respond ‘no’ or ‘yes’ to the items listed. All ‘no’ responses will be coded with a ‘0’ and all ‘yes’ responses will be coded with a ‘1’. If question number 1 or questions 1 and 3 were the only questions with ‘yes’ responses, Section C (Appetite Questions) may be skipped. Otherwise, the observers will continue by asking the respondent the questions in the appetite module. Questions 1-8 will be coded with a 0 or 1. Question 9 will receive a 1, 2, or 3, depending on whether the child’s appetite is good, so-so, or poor.

Section D contains the parental report scales, comprised of both the motor and language scales. Yes answers (1’s) will be coded when the caretaker reports that the child has achieved the particular item. The observer will begin with question number 1 when administering both the motor and language scales, despite the age of the child. If the child is older and the observer can clearly see that he or she is walking, the observer might want to tell the caretaker that he or she is supposed to ask all the questions, even if they seem funny. When 5 consecutive “no” responses (0’s) have been coded, the observer may stop asking questions about that scale before proceeding to the next one. If the caretaker does not understand a particular item, the observer may want to demonstrate. This may be easier to do with the motor items than with the language ones, but it should be possible with all of them.

The five motor items with an asteroid next to them must be demonstrated by the child before the observer marks “1”. In order to encourage the kids to perform these activities, the observers have been asked to do treat it like a game, exhibiting each of the skills before asking the kids to do the same. This is self-explanatory for the standing on one foot, skipping, and hopping items. However, for the questions that demand the child to walk forwards and backwards along a straight line, the observer will the piece of stiff rope that they were issued and will show the child that he wants him/her to walk forwards and backwards beside the rope. Beginning in Round 2 the observers will look for each of these items marked with an asterisk while they are observing in the field. When they return from the field they will complete the first part of the Home Interview Form for use at a later date and will mark “0” or “1” whether they saw the child perform these items. In the event that a “1” is recorded, it will not be necessary for the interviewer to ask the child to perform the item when he/she administers the DHIF. If a “0” is marked and the observer is still asking questions (5 consecutive “0”s have not been marked), the observer must try to elicit the behavior from the child.

Section E contains questions about the child’s temperament. Temperament in this context can also refer to the child’s level of fussiness or irritation. We are interested in knowing how the child reacts to stimuli in the environment. As with the other scales, “yes” responses will be coded with a 1; whereas, “no” responses will be coded with a 0. Question number 7 has caused some difficulty in the past during pre-testing phase. It might be easier for the observer to use an example for this particular item. For instance, does the child become very upset when the caretaker tries to put clothes on him? Putting on clothes is something that does not usually upset most children; however, it might upset this child. The ultimate example will have to come from the caretaker. Question number 12 requires the observer to mark 1, 2, or 3 to indicate whether it is easier than average, average, or more difficult than average to take care of the child.

**4. DIMAG CLINIC VISITS**

DIMAG includes two different types of clinic visits: Clinic A and Clinic B. Clinic A will occur at baseline and 12-months. This clinic will require members of the lab staff to administer a short morbidity questionnaire, take anthropometric measurements, and draw blood. Clinic B will occur independent of Clinic A. Children who are within 7-days of 39- and 52-weeks will be eligible for this visit. The clinic staff will administer a short morbidity questionnaire, the A-not-B Task, and the Fagan Test of Infant Intelligence.

**4.1 DIMAG Daily Clinic Schedule (DDCS)**

Before DIMAG begins, the clinic staff will transfer data from the DCTL to the DDCS in order to schedule the Clinic visits. Clinic A visits will occur the day after the observers do the observation at baseline and 12-months. The DIMAG kids will be scheduled by the location of their house, by the Ward and Sector in which they live. The Clinic A1 visits, the baseline ones, will occur simultaneously with the Clinic B visits. Ideally, the Clinic A and Clinic B visits will occur on separate days; however, there will be occasions when it will be necessary to schedule both on the same day. This will be possible as long as each member of the staff is present and two can work on Clinic B, while the rest devote their time to Clinic A .

Clinic B visits will be scheduled using the DBF within 7-days of the when the child turns 39- and/or 52-weeks. They will occur in the morning when the children are most likely to be alert and active. The form has space for basic identifying data. It also has boxes to indicate which clinic the child will participate in (A or B) and which visit this will be (A: 1 or 2; B: 39 or 52 weeks). There is also space for indicating whether the form has been completed and for comments.

**4.2 Clinic A**

**4.2.1 DIMAG Clinic A Form (DCAF)**

To begin Clinic A, one of the clinic staff will ride in the project vehicle with the driver each day to administer Section B, the morbidity questions, to the caretakers before the children come into the office. Children that have been sick in the 24-hours preceding the clinic visit will not be brought into the office and will be rescheduled for another date. These criteria changed for Clinic A1 once we reduced the phlebotomy protocol to collecting drops of whole blood for hemoglobin and zinc protoporphyrin. Because neither of the two measures is significantly impacted by morbidity and we needed to see the kids quickly, we decided to bring everyone into the clinic regardless of their morbidity status. It was at this time that we began taking body temperature with the digital thermometers. Because the Clinic A2 visits were scheduled closer to the observer (in most cases, the day after the observation), the clinic staff encountered fewer sick kids when they went to the field to transport the subjects to the lab. Because the kids were healthy, the staff did not take temperatures until the third week of data collection when the decision was made to collect the same data that were collected during Clinic A1.

Sections C and D are devoted to the two main components of Clinic A: anthropometry and phlebotomy.

- - 1. **Anthropometry**

In order to complete Section C, our DIMAG anthropometrist will take the following measurements: MUAC (cm), head circumference (cm), triceps skinfold thickness (cm), weight (kg), and recumbent length (cm). Each of the measurements, with the exception of weight, will be taken three times and recorded on the DCAF. The Seca Scales used to measure weight have been deemed sensitive enough to only need one measurement.

- - - 1. **Mid-upper arm circumference (MUAC)**

When measuring the DIMAG children’s MUAC, the staff member will use a plastic, calibrated arm circumference tape. The ones that we are using for DIMAG were created for JIVITA, the Johns Hopkins Vitamin A study in Bangladesh. These tapes are stored in old vitamin supplement bottles that were cut in half to accommodate the tapes. The protocol for measuring MUAC follows.

1. Remove the tape from the protective container and check to see that it is not torn or broken.

2. Free the child’s left shoulder and arm from clothing and position the child so that his left side (and left arm) is facing the anthropometrist. The mother may hold the child in her lap. Older children may prefer to stand.

1. Find the midpoint of the upper half of the left arm:
   1. Flex the child’s arm at the elbow.
   2. Mark the top of the child’s arm at the tip of his shoulder blade with your left middle finger and his flexed elbow with your right middle finger.
   3. From these positions, run your two thumbs together at the same place along the upper arm until they meet at the mid-point.
   4. Place a pen mark at the mid-point.
2. Relax the child’s arm. Let it hang loosely down by his side.
3. Once the child is calm, wrap the tape around the arm at the mid-point and slip the end through the slit.
4. Check to see that the tape is firm around the arm, but is not indenting the skin.
5. Read the number next to the arrow on the side of the “window”. Record this number to the nearest 0.1 cm on to the DCAF.
6. Loosen the tape slightly and repeat steps 5-7 two more times for a total of three measurements.
   - - 1. **Head circumference**

For the head circumference measurement, the anthropometrist and observers (the observers will take this measurement in the field beginning with the second round of data collection) will use a long, calibrated measuring tape that has been designed for the purpose of measuring a young child’s head.

1. Free the child’s head from ribbons, rubber bands, or other headwear that might impede the head circumference measurement.

1. Take the head circumference measuring tape from its container. Thread the end through the double opening where the anthropometrist or observer will eventually take the reading and place around the child’s head.
2. Rest the back of the tape on top of the bone in the middle of the back of the child’s head. Position the front of the tape so that the window where the measurement is read is between the eyes and above the eyebrows.
3. Pull the tape so that it is neither loose nor tight. Read the measurement to the nearest 0.1 cm.
4. Loosen the tape slightly and repeat steps 3 and 4 two more times for a total of three measurements.
   - - 1. **Triceps skinfold**

The anthropometrist will use Holtain skinfold calipers to measure triceps skinfold. The calipers will be stored in a protective case inside a metal box when not in use. Prior to conducting the measurements, check that the needle on the calibrated dial points to zero. Adjust if necessary.

1. Refer to steps one and two from the MUAC protocol above for positioning the child for the measurement.

1. Approach the child from his back left side. Pinch a vertical fold of skin with the left hand about 2 cm above the mid-line of the upper left arm on the back side. Roll slightly to insure that the fold does not include deeper underlying muscle.

3. Holding the caliper with the right hand at a right angle and horizontal to the fold, open the jaws and extend to the elevated fold and release the handle. Continue holding the pinched fold of skin.

1. Hold the caliper in place for approximately 2-3 seconds without looking at the pointer on the dial.
2. Read the value to the nearest 0.1 mm (the ticks are at every 0.2 mm) on the DCAF.
3. While continuing to hold the fold, remove the calipers from the child’s arm and record the value on the DCAF.
4. Repeat steps 2 to 6 two more times for a total of three readings.
   - - 1. **Weight**

To measure weight the anthropometrist will use one of the many Seca scales in the office. These scales, manufactured in Australia for UNICEF, were designed to subtract the child’s weight from that of the mother’s. Because the scales are sensitive measures, the anthropometrist will only take one measurement. The protocol for weighing the DIMAG children follows:

1. Remove excess clothing from the child’s body.
2. Remove the scale from the box in which it is kept and place it on the floor in the room designated for the anthropometry to take place.
3. Wipe a bare foot or hand across the sensor located near the window in which the weight in kilograms is displayed to turn the scale on.
4. Wait for 0.0 to appear. Ask the mother to step on the scale. Request that she be patient because she has to be measured before we are able to measure her child. When her weight appears, wipe the scale once again and hand the child to the mother for her to hold while the scale is taking the measurement.
5. When the child’s weight appears, the anthropometrist will record this reading on the DCAF and will ask the mother to step down from the scale.
   - - 1. **Recumbent length**

For measuring recumbent length the anthropometrist will use a Schorr board, a calibrated measuring board with a sliding block to adjust to the different lengths of the DIMAG infants. Typically, recumbent length is measured for children less than 2 years (24 months) and height is measured for children greater than this age; however, for the purpose of being able to compare our baseline and end-of-study data, we will only use recumbent length to measure the DIMAG children. The recumbent length protocol follows:

1. Place the Schorr board on the table in the designated Clinic A testing room. Place a small, clean towel at the fixed end of the board for the child’s head.

1. Remove shoes, sandals, or socks from the child’s feet and gently lay the child on the board with his head at the fixed end and his feet toward the sliding block.
2. Position the child’s head so that the top of the head touches the fixed end and the child’s gaze is toward the ceiling. An assistant or parent may apply gentle pressure under the right and left sides of the chin to keep the head in place.
3. Straighten the legs using gentle pressure across the knees, so that the feet point towards the ceiling. Gently push the sliding block toward the feet until they are flush against it. Check the child’s position for accuracy.
4. Read the length to the nearest 0.1 cm at the point where the sliding block rests against the feet.
5. Relax the sliding block and repeat steps 3 to 5 two more times for a total of three readings.

### 4.2.3 Phlebotomy

Section D reflects the changes in protocol that we made between the time Clinic A1 was started and completed. Question 1 asks that the staff member completing the form indicate whether a venous or capillary sample was taken. Ultimately, the collection of venous and capillary samples was dropped from the protocol once it became apparent that collecting a quantity of blood was not culturally acceptable to the majority of the mothers and paternal grandmothers in our study population. The venous blood draws proved to be especially challenging for our staff when they were presented with healthy children whose subcutaneous fat obscured their veins. Although the heel prick technique used to collect the capillary samples was acceptable to the mothers and grandmothers, these individuals, in just about every instance, pulled their child away from the lab technician once they thought the tech had collected enough blood for the capillary tubes. Their concept of “enough” and ours were very different. In the end, for Clinics A1 and A2 we resorted to extracting drops of blood via heel prick for the ZPP and hemoglobin readings.

**4.2.3.1 Blood draw**

The protocols for the venous, capillary, and heel prick blood draws follow.

Venous Blood Draw

1. Draw a 3 ml blood sample into the vacutainer.

1. Blood from the tubing will be dripped onto the cover glass for the zinc protoporphyrin machine and the HemoCue cuvette.
2. Insert the ZPP slide and HemoCue cuvette into their respective machines.
3. Record the ZPP and hemoglobin readings on the DCAF.
4. Place the vacutainer of blood into the refrigerator until all the blood draws are complete for the day.
5. When all subjects are finished, take the tube out of the refrigerator and let it sit for 10 minutes in the summer and 20 minutes in the winter before centrifuging.
6. Centrifuge the blood at 3000 rpm for 15 minutes. If the serum and red blood cells have not separated, spin for another 10 minutes.
7. Place a paper label on the front of the DCAF and put a corresponding satin label on to each of the cryovials. Put 1 ml of serum into each of 2 cryovials and the remainder of the serum into the third cryovial. Place all three cryovials with labels into a Ziploc bag and then into the liquid nitrogen tank.
8. Each day, check the liquid nitrogen level in the storage tank. When the level dips below the full line, add more liquid nitrogen from the filler container.

Capillary Blood Draw

1. Use a lancet to prick the child’s heel

1. Wipe the first drop away. Place the second on to the slide for ZPP and the third on to the HemoCue cuvette.
2. Fill two capillary tubes with 300 uL blood.
3. Insert the ZPP slide and HemoCue cuvette into their respective machines.
4. Record the ZPP and hemoglobin readings on the DCAF.
5. Wait 30 minutes and spin the two capillary tubes in the centrifuge at 4000 rpm for 15 minutes.
6. Transfer the serum from each capillary tube into a separate cryovial.
7. Place a paper label on the front of the DCAF and put a corresponding satin label on to each of the cryovials. Place both cryovials into a Ziploc bag and then into the liquid nitrogen tank.

Heel Prick Blood Draw

1. Use a lancet to prick the child’s heel.

1. Wipe away the first drop. Place the second on to the slide for ZPP and the third on to the HemoCue cuvette.
2. Insert the ZPP slide and HemoCue cuvette into their respective machines.
3. Place cotton and apply pressure to the place where the heel was pricked.
4. Give biscuits to the child and send both the mom and child on their way.
5. Record the ZPP and hemoglobin readings on the DCAF.

Questions 2, 3, and 4 of the DCAF address hemoglobin, zinc protoporphyrin, and temperature readings. Both the HemoCue machine and Flurometer will display readings within a minute or two of inserting the slide. All children with a hemoglobin reading less than 7.0 will be given an iron-rich tonic available in the local market. A lab staff member will show the mother how to give the tonic to her child daily until the contents of the bottle are gone. To obtain body temperature, one of the lab staff members will insert a digital thermometer under the arm of each child. This thermometer will beep when the reading is complete.

**4.2.3.2 Lab machine calibration**

Before the DIMAG kids are brought into the office for the Clinic A visit, the HemoCue and flurometer machines are checked for accuracy. The HemoCue is checked daily, whereas, the flurometer is checked weekly.

HemoCue calibration:

- 1. Remove the control cuvette from its box and insert in the HemoCue.
  2. Record reading, date, and staff ID in the designated logbook. The control cuvette that we used for Clinics A1 and A2 had an accuracy reading of 12.6-g/dl +/- 0.3 g/dl.
  3. When the reading is a borderline reading (i.e. 12.3 or 12.9 g/dl) or exceeds the specified range, remove and clean the black slide tray and the eye that reads the cuvette located on the roof of the opening. Once the tray has dried, insert the control cuvette again and record reading. Repeat until reading is within the specified range.
  4. Clean the machine weekly and when a borderline reading is present using an alcohol wipe and a cotton ball. First wipe the machine with the alcohol sachet. If it is especially dirty (i.e. encrusted with spots of blood), immerse with soap and water before applying the alcohol. Use the cotton to wipe the inside of the machine. Air dry. When the slide is completely dry, re-insert into the machine.

Flurometer calibration:

- 1. Place a glass cover from the small plastic box from AVIV on the extended slide tray. Push the measure button. Use the slide if the reading is between –000 and 001. Otherwise, discard into the designated receptacle and repeat process.
  2. Begin by checking the calibrator values. Check to see that they are room temperature before using. Rub between hands to warm. Tip from top to bottom to ensure that the contents are sufficiently mixed before dripping on to the glass slide. Begin by testing the red value: range = 362 +/- 18 (344-380). Follow by testing the blue value: range = 52 +/- 5 (47 – 57).
  3. If both the red and blue values are between the appropriate ranges, proceed to testing the control values. If any one of these measurements is outside the range, calibrate the machine by hand. Locate the screw marked “calibration” on the back of the machine and turn slightly, toward the + or – depending on whether the value was less or greater than the appropriate range. Place the same calibration slides into the machine and repeat step number 2. Repeat until the values are within the appropriate range.
  4. Check the control values. Determine which slides are acceptable for use. Test the blue, white, and red vials in this order. The appropriate ranges for each follow: blue = 74 +/-9 (65 – 83); white = 173 +/- 18 (155 – 191); and red = 281 +/- 28 (253 – 309).
  5. If any of the blue, white, or red values are out of range, do step number 3, except adjust the bottom screw labeled “zero.” Once the control values are within the appropriate range, retest the calibration values to make sure the adjustment didn’t change the calibration measurements. Once all the values are within range, begin Clinic A.
  6. Between weekly measurements, store the calibrators in the freezer and the controls in the refrigerator.
  7. **Clinic B**

**4.3.1 Scheduling**

The timing of Clinic B is dictated by the ages that the children are eligible to be tested for the Fagan Test of Infant Intelligence. Although the test has been designed to test children at four ages, we have chosen only two of these for DIMAG: 39- and 52-weeks. All children that attain 39- and 52-weeks of age while they are enrolled in DIMAG will be scheduled for the age-appropriate Clinic B visit. Each DIMAG child’s birth date will be entered into a computer program that Luke Mullany has designed to assist us in scheduling the Clinic B visits. The program will tell us which NNIPS weeks the DIMAG children are eligible for testing. For a child to be eligible for the 39- and 52-week testing sessions, the child must be tested within a 7-day window that falls on either side of the child’s 39- and 52-week birth date. A printed schedule will be generated from the data center at the beginning of DIMAG. It will be updated once baseline enrollment is complete and the master list of DIMAG kids has been created. This schedule has sorted the children by ward, sector, and household to make it easier for the lab staff to schedule children living in the same area to come into the office on the same day.

When the mothers and children arrive in the office, one of the testers will greet them. The person will explain that we are going to test the children’s memory and mind by administering two developmental tests. One is called the A-not-B Task and requires the child to find a toy that has been hidden in one of two wells in a table. The other, the Fagan Test of Infant Intelligence, presents the children with different sets of faces that have are mounted on a wooden board. The children will do the A-not-B Task before the Fagan Test, unless they appear to be very scared of the testing dynamic. Should they be scared, the testers will have the option to administer the FTII before the A-not-B Task. We understand that the tests and testing conditions are new and that the children might need some time to adjust. This is okay. We will give the children an assortment of toys, including rocks and balls, with which they can play while they are waiting for their turn. We do not expect the visit to take longer than 2 hours.

**4.3.2 DIMAG Clinic B Form (DCBF)**

The testers will complete Section A of the DCBF using data from the DCTL before the child arrives at the office. Only the Respondent’s Name, Relationship to Child, and Form Status questions will be left for the tester to ask the caretaker before the test begins. A member of the clinic staff, who accompanies the driver to pick up the children scheduled to be tested, will complete Section B in the child’s home to ensure that we will not be testing sick children. All children who have been sick in the 24-hours preceding the clinic visit will be rescheduled for another day when they are well.

In the office, the testers will begin by administering the A-not-B Task. Once the children have completed the A-not-B Task, or it has been decided that they are unable to complete the test, another tester will call them into the room that houses the computer and will administer the Fagan Test. For both Sections C and D, the tester will be required to complete the following information: whether the testing date is outside the testing window, which of the two tests was performed first, the time of day that the test was performed, the duration of the test, and whether the test was completed. The dates for the testing window are printed on the scheduling form. It will be the tester’s job to match the date that the test was completed with the dates on the form to determine whether the test was performed inside or outside this window. Ideally, we do not want to test kids whose testing windows have passed and who are therefore ineligible for testing. The time of day that the test is administered is determined by a.m. and p.m. The testers will have to record whether the test was administered before or after noon. The duration of the test includes any pauses that might have occurred during the test. The reasons listed for not completing the test include the following: baby closed eyes; baby turned to breast; baby looked away; baby fussy; and other. Because the A-not-B Task demands more from the child, two additional reasons are listed in Section D for not completing the test. These include: baby did not engage with tester and baby not able to pick up cloth or toy.

There are additional questions that are unique to each of the two tests. In Section C, questions 3 and 4 require that Shishir, Emily, or one of the lab staff trained to use the computer copy the raw and scaled novelty scores from the FTII program on to the form. In Section D, the tester must ask the mother whether her child found the toy fewer, about the same as, or more times than she expected and record the answer in the box provided next to question 3. There is room for comments in both sections, should the tester want to make a note about the testing conditions or anything else that might have happened while the test was in progress.

# 4.3.3 A-not-B Task

**4.3.3.1 Introduction**

The tester begins by inviting the caretaker and child to sit in a chair across the table from the tester. The child will be seated in the caretaker’s lap and positioned so the child is equidistant from the two wells in the middle of the table and far enough from the table that the child cannot remove both of the cloth covers at the same time.

The tester will explain to the caretaker that the child is about to perform a test that will test the baby’s mind and memory. The test begins when the tester hides a toy in one of the two wells and asks the baby to find it. Between the time that the tester hides the toy and the child is asked to look for it, the tester will be responsible for distracting the child from looking at or reaching towards the wells. The tester will encourage the caretaker to move the child’s arms (clapping or pumping the arms up and down seems to work well). If the caregiver is unable to restrain the baby, the tester will reach across the table and grab and play with the baby’s hands as a means of distraction.

# **4.3.3.2 Warm-up**

To begin the test, the tester puts both of the cloth well covers on the table with a selection of toys (including a small rock for this population that doesn’t usually have toys available for play). While the tester records the identifying information that is needed for the A-not-B Worksheet, he/she will suggest that the child choose a toy. Once the child has shown preference for one toy, the tester will remove the other toys from the child’s sight. The tester will then give the child a few minutes (if the child has not already been playing with the toy) to play with the toy.

Next, the tester will place the toy between the two wells, will cover it with one cloth (the other cloth will be out of sight), and will ask the child where it is. “Where is the toy?” When the child is able to successfully uncover the toy, the tester will cover the toy again; however, this time the tester will impose the delay. Once the child is able to remove the cloth to find the toy underneath at the end of the delay period, the tester may begin the test.

If the child has trouble removing the cloth or appears distracted by the cloth, the tester will present both the cloth and toy to the child for further exploration. The purpose of presenting the cloth is for the child to become familiar with it. If the child seems to be viewing the cloth as a desirable object, the tester will have to remove it and try to interest the child in the toy. Ultimately, the point of the task is for the child to want to retrieve the toy from under the cloth and within the wells. After giving the child a few minutes with the cloth and toy, the tester will begin again with the warm-up exercises.

# **4.3.3.3 The A-not-B Task**

The tester will begin this task by making sure that the child is centered between the two wells and is far enough from the table that he/she is not able to slide both covers off the wells. Instead the child will have to reach to uncover the cloth from one of the wells.

The tester will pick up the toy and will make sure the baby is looking at it before he/she places it into the first well, covers both wells with the cloths, and starts the timer. The tester will begin by hiding the toy in the left well (side A). Once the child has performed correctly on two trials in a row, the side will be switched to side B. After 2 correct performances in a row at side B, the side will be switched again to side A. The test will proceed until the stopping criteria have been achieved.

## **4.3.3.4 Setting the delay**

Delay:

Use a 3-second delay for infants 9.0-10.5 months of age.

Use a 5-second delay for infants 11.0-12.5 months of age.

Rules for setting the delay:

*Begin using the appropriate delay for the child’s age.

*If the child performs perfectly on the first 3 trials, increase the delay:

9.0-10.5 month child from 3 to 5 seconds

11.0-12.5 month child from 5 to 8 seconds

*If the child performs perfectly on the next 3 trials, increase the delay further:

9.0-10.5 month child from 5 to 8 seconds

11.0-12.5 month child from 8 to 10 seconds

*After 6 trials do not adjust the delay.

*After 2 adjustments to the delay, do not adjust further. Keep the present delay.

*If the child makes a mistake on the first or second trial, reduce the delay:

9.0-10.5 month child from 3 to 1 second

11.0-12.5 month child from 5 to 3 seconds

*If the child makes a mistake on the first or second trial after the delay, further reduce the delay:

9.0-10.5 month child from 1 to 0 seconds

11.0-12.5 month child from 3 to 1 second

*If the delay has been reduced twice and the child makes another mistake, do not reduce further.

*If the child performs correctly on the first and second trial, but makes a mistake on the reversal trial do not change the delay. This is called the A-not-B error and means that the initial delay used is appropriate. Continue using the same delay.

*If the child makes a mistake on the first or second trial, the delay is reduced and then the child performs correctly on the next 3 trials, increase the delay and do not change it further.

*If the child performs correctly on the first 3 trials, the delay is increased and the child subsequently makes a mistake on the next first or second trial, reduce the delay to the original one used and do not change it further.

## **4.3.3.5 Stopping rules**

Begin by hiding the toy on the left side. Continue to switch sides after the child has performed correctly on 2 trials in a row on a given side. The test will end after the child performs two correct trials in a row:

*After 3 reversals if total trials at testing delay after two reversals is >12.

*After 4 reversals if total trials at testing delay after 3 reversals is > 15.

*After 5 reversals if total trials after 4 reversals is < 15.

Other Points to Remember*:*

*After errors on 5 consecutive trials, hide the toy using only one cover. Once the child finds the toy under the cover, return to using two covers.

*During the delay, the tester should not clap to distract the child during the delay, as clapping is too distracting. Moving the child’s arms or using the face and/or voice should be sufficient.

*If there is any indication that the child does not understand the point of the game, play with the toy and a cover in the middle of the table until the child knows to find the toy.

*Give the child a chance to get bored with the cloths before testing begins, or during the testing if the cloth distracts the child.

*Make sue the child is looking for the toy and not the cloths. This can be done by not cheering until the child starts to reach for the toy itself.

*If there is any indication that the child is getting bored with the toy, switch toys by offering all the toys once again and allow the child to make a selection. Do not switch toys on a reversal trial if you can help it.

*It is counted as wrong if the child fails to reach to either well. Remind the child that the toy is still there by asking, “Where is the toy?” If the child still does not reach, go through the hiding procedure again or change toys.

*If the child uncovers both wells, code the well to which the child is looking the first time that this happens. The tester will discourage the child from reaching with both hands by keeping his/her hand on both covers, and make the child chose by not releasing the covers until the child is only pulling on one.

*Allow the child to reach to the correct well (“self-correct”) if the first reach was wrong. Allow the child to uncover the correct well, but do not allow the infant to retrieve the toy. The child only gets the toy if the first reach was correct. If the child does not self-correct, make sure the child is looking when you show him or her where the toy is. Do not allow the child to take the toy out. Immediately go to the next trial before the child gets too frustrated from the disappointment.

*Once the child begins to reach do not distract him or her.

*If the child is upset during the delay, it is good to have the caretaker bounce the child (but never during the hiding).

*Always keep the child centered.

*Cheer robustly when the infant is correct, unless you find that the infant is shy.

**4.3.3.6 Trial definitions**

*Reversal = side of hiding changed (these occur after each time the child has reached correctly twice in a row at the same place).

*Repeat-following-correct trial = side of hiding remains the same and subject was *correct* on the previous trial (if the child is correct on this trial, side of hiding is reversed on the next trial).

*Repeat-following-error trial = side of hiding remains the same and subject was *wrong* on the previous trial.

*AB Error:

1. No more than 1 error on a Repeat-following-correct trial, and
2. at least 1 error on a reversal trial, and
3. either: i) an error on at least one more reversal trial; ii) an error on the trial immediately following the reversal error.

*Correct Performance = > 85% correct

*Deteriorated Performance = < 75% correct; > 1 error on repeat following correct trials.

**4.3.4 The DIMAG A-not-B Test Form (DABTF)**

Before the visit, the tester will copy the identifying information from the DCTL that is needed to complete the top of the DABTF. The Western calendar will be used for the date. The variable will be written in the European format with two digits expressing the day, month, and year.

The tester will check each of the boxes when the child performs correctly and will place an “x” when the child makes an error. The columns “Starts-L” and “Starts-R” correspond with the tester hiding the toy on the tester’s left and tester’s right. As is mentioned above, the task will always begin with the tester hiding the toy on the tester’s left. Under the third column labeled “Toy” the tester will record the toy that the child selected for the task. If the toy changes, the tester will note the change in the appropriate box under this column. The fourth column, “Reached at hiding” requires the tester to record when the child does not reach (NR). In the case of NR, the tester will mark an “x” in either the first or second column and will then write “NR” in the appropriate box in this column. The fifth column “Delay Number of Seconds” is where the tester will record the duration of the delay that is imposed during the test. The sixth column “Used 1 Box” will be marked after the tester responds to a child making five consecutive errors by placing only one cloth over the well in which he/she has placed the toy. The sixth column, “Saw 2 boxes” refers to instances in which the child pulls both cloths off of the wells at the same time. The tester will mark the seventh column, “SC or PR” when the child self-corrects (SC) or demonstrates a partial reach (PR) and will code both types of trials as error trials, marked with an “x.” When the tester has to pause or stop the test, he/she will mark a “P” or “S” in column number 8 to indicate which occurred and will then record the appropriate code in column 9 to indicate why he/she had to pause or stop the test. The codes follow: 1 = baby closed eyes; 2 = baby turned to breast; 3 = baby looked away; 4 = baby fussy; 5 = baby did not engage with tester; 6 = baby not able to pick up cloth or toy; 7 = other. Once the test is complete, the tester will ask the mother whether the child found the toy fewer times than, about the same as, or more times than expected and will record the answer on to the DABTF and DCBF.

**4.3.5 The Fagan Test of Infant Intelligence**

The Fagan Test begins when the tester calls the mother and child into the room that houses the computer on the ground floor of the office. The tester will direct the mother to sit in the chair closest to the door, across the table from the tester’s chair. Prior to the mother entering the room, the tester will have already turned on the computer and clicked on the FTII icon to enter the FTII program. For the first few minutes that the mother and child are sitting in the chair getting used to the room, the tester will type the identifying information into the program. This information is found on the front page of the DCBF and is presented below. Because we have no data on length of gestation or low birth weight in this population, which can be used as a proxy for gestation, we will record into the program that all children were 40 weeks gestation. Although we are certain that this isn’t true, the fact that the children have been randomly selected to be in study allows us to make the assumption that the distribution of gestational ages will be equal in each of the four treatment groups. Post-gestational age is defined as the length of time between the child’s birth date and testing date. This number will be between 38 and 40 weeks and 51 and 53 weeks. For the purposes of this program, the tester will either enter 39 or 52 weeks to reflect the post-gestational age of the child.

Identifying information needed for the Fagan Test program:

1. Subject # = NNIPS #

2. Baby’s name = Baby’s first and last name

3. Baby’s Hospital # = Today’s date (Nepali: dd/mm/yy)

1. Tester’s name = DIMAG tester’s initials
2. Sex = Male = 1; Female = 2
3. Race: 1= Pahadi; 2 = Madeshi; 3 = Muslim; 4 = Other
4. Month of birth = Nepali month
5. Day of birth = Nepali day
6. Year of birth = Nepali year
7. -----------------
8. ------------------
9. Gestational Age = 40 weeks
10. Post-Gestational Age = 39 weeks or 52 weeks
11. ----------------------
12. ----------------------
13. ----------------------
14. ----------------------
15. ----------------------

Once the identifying information has been entered into the FTII program and the program has signaled the tester to place the first two pictures on to the stage, the test may begin. At this point, the tester will explain to the mother that he/she will be placing pictures on to the contraption in front of her, called a stage. The tester is going to be watching the child’s eyes from a hole that has been cut into the stage. The mother is not to direct the child’s gaze or attention in any way. Should the child begin to fuss, it will be okay for the child to eat a biscuit. However, if the mother thinks that the child might be hungry, the tester will ask her to please feed the baby before the test begins.

The FTII computer program tells the tester which combination of photos needs to be placed upon the stage. After the tester fastens the pictures in the appropriate place using Velcro and closes the test door, the tester will look through the hole in the stage and indicate using the right and left arrow keys which direction the child’s eyes are looking. The computer will beep to alert the tester to open the stage door and replace the pictures with the new ones before the next trial begins. Once the sequence of pictures has been displayed and the child has viewed each one for the appropriate amount of time (the computer program calculates this time automatically) a series of beeps will sound to alert the tester that the test is complete. If the tester is not able to complete the test, the tester will record the reason on to the DCBF. The codes follow: 1) baby closed eyes; 2) baby turned to breast; 3) baby looked away; 4) baby fussy; and 5) other.

The mother will be directed to the courtyard where she will be given tea and samosas for herself and biscuits for her child before being transported in the office vehicle with the other mothers back to her home.

At the end of the day before the tester places the form in the appropriate cubbyhole for editing at a later date, the tester (or Emily, Shishir, or one of the Clinic B staff) will import the FTII data into Microsoft Word and will extract the raw and scaled novelty scores for each child that was tested for that day and will record these on to the DCBF. The tester will also create folders for each day of testing that he or she will name with the appropriate Western date. These folders will be placed into larger folders that are organized by NNIPS Week. These data will be backed up on to two floppy disks, one that is kept next to the computer and another that is stored in the large cabinet in the upstairs office room.

**5. DIMAG TRAINING**

**5.1 Introduction**

Between April and December 2001 the NNIPS-4 fieldworkers who were assigned to work on DIMAG were trained to do the tasks that would be required of them once the study began. The training period was especially long due to a delay in receiving the supplements from France. The training began with preparing for the observation, since everything associated with the task, especially the material and the equipment, was new. Once the observers were familiar with the codes, many of which overlap with the Motor Milestones Checklist, they were able to assist in training the WDs to administer the Motor Milestones Checklist. In July training began for Clinic B. Four of the clinic staff members were taught how to administer both the Fagan Test of Infant Intelligence and the A-not-B Task. There was ample time during the training period for each of the workers to practice their skills prior to the beginning of the study.

**5.2 Observation**

Before the official training for the observation began, Emily worked with Shirshir to understand the codes and apply them to the field setting. Emily and Shishir used a stopwatch, paper, and a pen to record the target child’s behaviors. At the end of each practice session, they discussed the observation and any discrepancies in their coding technique. They were ready to begin training the 15 observers once they reached consensus.

The observer training began with a verbal description and visual demonstration of each of the codes. Emily personally demonstrated the 20 action, 9 social behavior, and 6 vocalization codes before calling on each of the observers to do the same. Once it was clear that they grasped the idea, they were asked to group and perform vignettes using a combination of assigned codes. The observers began observing in groups in the field and by watching videos of Shishir’s daughter. They used paper and pencil before graduating to using the Visors. After a couple of weeks of practice, a kappa program was added to the CAOS (Child Activity Observation Software). We used the kappa program to determine which codes were problem codes. We found examples of these codes in the video and discussed them as a group. When it became clear why everyone wasn’t coding the same thing, we discussed the correct methodology and developed clearer coding rules. Once the code descriptions were clear to each of the observers, they returned to the field to perform observations in small groups. During this time, it was acceptable for them to discuss questions with their respective group members as problems arose. We attempted to begin the standardization in the field; however, the observers could not stop talking between themselves. For this reason, we resorted to doing it with a video in the lab, where we could control the process and use a single person as a gold standard to which all the observers’ observations could be compared. The training was determined to be complete once everyone achieved standardization with our gold standard, Shishir after watching a one-hour video. In order to be considered standardized, each of the observers had to achieve an agreement score of 70% or greater on codes that had 15 or greater instances.

**5.3 Motor milestones**

The Motor Milestones training was conducted using similar techniques that were used to train the observers. With the assistance of the AC that was assigned to Ishwarpur and one of the observers, Emily introduced each of the motor milestones to the 23 WDs who would be collecting the motor milestones data. First, the Motor Milestones Checklist-DIMAG was circulated around the room and the WDs were asked to watch as Emily demonstrated the codes. Second, the WDs were asked to demonstrate the codes individually. The AC went around the room several times until each of the WDs had demonstrated at least 2 milestones. Then he quizzed the WDs randomly. In the case that the WDs appeared to have trouble with the codes or otherwise appeared weak, the AC asked the WD to demonstrate as many codes as she could. Once the WDs were able to demonstrate the codes with proficiency, they went to the field in small groups that were supervised by an observer or member of the clinic staff who was also trained to administer the questionnaire. By the end of the first week of training (about 8 hours), all but four WDs were deemed to be ready to collect data. The four remaining WDs needed a little more time than the others to improve their literacy training and time working with the questionnaire in the field. At the end of the second week all the WDs were ready to administer the instrument. Three months after data collection began, an additional WD was hired to assist another WD who was responsible for collecting data for a particularly large area. The same AC that was involved in the original training trained this woman in the field by going through the codes with her and accompanying her to the field until she was able to perform the task without having any problems.

**5.4 Clinic B: Fagan Test and A-not-B Task**

The challenge in training the clinic staff to do both the Fagan Test and the A-not-B Task lay in gathering proper training materials. Dr. Joseph Fagan created a video and exam to accompany his Fagan Test kit; however, the video had not been updated since he revised his software. As a result, it was very difficult to use the video for training and standardization purposes. The only way that the video would work was for the clinic staff member to code into the computer the opposite eye movements that were shown on the video. The exercise was futile, particularly if the aim was to teach the staff members how to operate the software. For the four clinic staff members, Emily acted as the child and looked in the same direction and same sequence at the Fagan Test pictures as each of the staff members performed the test. Agreement between observers was determined based on the resulting Fagan Test scores. The staff members were allowed to begin administering the Fagan Test once their scores matched. Three of the four observers received the same score the first time that they administered the test. The person who had a different score redid the test. Her Fagan Test score the second time she administered the test matched the others’.

Dr. Joseph Fagan has included a test in his Fagan Test kit. The questions are varied and range from asking what equipment is required for the test to how to proceed in the event of having to pause or stop the test. The four staff members completed the test. We sent their responses to Ohio where they were graded by Dr. Fagan’s staff. Several months later Dr. Fagan’s lab awarded our clinic staff with Fagan Test certificates that arrived in the mail.

Emily contacted Dr. Adele Diamond to ask for instruction in administering the A-not-B Task. Dr. Diamond’s lab sent both a protocol for administering the test and a video of one of the lab’s testers administering the test to a child in Massachusetts to assist with our training. The three A-not-B Testers discussed the protocol and possible problems that might arise while testing. When they were ready, we brought infants between 8 and 13 months of age for them to test. Once they felt comfortable with the test and its many rules, Emily acted as the infant for 8 different sessions with each tester. The testers were deemed standardized once they were able to administer the test correctly for each session. They were observed halfway throughout the trial to verify that they were still conducting the test correctly.

**5.5 Home interview**

Emily and Shishir explained each of the sections included in this form to the observers. While much of it was straightforward, the observers needed additional instruction for the motor and language scales. Individually and as a group they explained how to administer the questions marked by an asterisk and had the field staff demonstrate that they understood. Skipping appeared to be the most difficult item to learn, as many members of our field staff were unable to skip.

**5.6 Anthropometry**

Because the NNIPS staff have been taking anthropometric measurements since the first NNIPS in 1989, it was not necessary to do much training for the anthropometry module. Rajeswori and Kalawati showed me their technique for each of the measurements we wished to take for DIMAG: MUAC, head circumference, triceps skin fold, weight, and length. The two of them demonstrated the proper technique to the NNIPS-4 TLIs and ACs prior to the MUAC standardization and the start of NNIPS-4 and to the DIMAG observers prior to the head circumference standardization exercise at the end of Round 1.

**5.7 Phlebotomy**

Jaybar, Chandeswor, and Kalawati traveled to Kathmandu for training in infant phlebotomy. They spent 4 hours in the Outpatient Department at Kanti Hospital where a nurse taught them how to draw blood from young infants. They each practiced with 15 to 20 babies before they returned to the office. What they needed was someone to accompany them to the field to assist them when they had trouble. None of the three were able to find the vein in “fat” babies. Sometimes they stuck the child multiple times and still were not able to find it. It would have been helpful had a more experienced person been able to show them how to draw blood from babies with fat on their arms. Because of the problems that the phlebotomists had and resistance from the child’s parents, we changed the protocol and discontinued the venous blood draws.

**6. DIMAG STANDARDIZATION AND RELIABILITY**

In an effort to preserve the integrity of the data, many standardization and reliability exercises were conducted throughout the course of DIMAG. The anthropometry standardization was performed at baseline and during the course of the study when the data collection logistics were changed, causing workers to assume new tasks for which they had not been standardized previously. The observers’ observations were standardized at baseline and after data collection rounds 1 through 4. The WDs were standardized at baseline and every three months beginning at the end of Round 1, six months after they were first standardized, and ending at the end of NNIPS-4. The clinic staff was standardized in their ability to administer both the A-not-B Task and the Fagan Test of Infant Intelligence at baseline and observed performing the test approximately 4.5 months after the study started. Beginning with Round 2 the observers began a reliability exercise that tested their ability to administer the Home Interview Form and the mothers’ comprehension of this form. This reliability exercise continued through Round 5.

### 6.1 Anthropometry

Because of Rajeswori’s experience in performing anthropometric measurements during past NNIPS trials, she was chosen as a gold standard against which Kalawati, the DIMAG staff, and the rest of the NNIPS TLIs and ACs were compared for the DIMAG Clinic A assessments, head circumference in the field, and the greater NNIPS-4 MUAC standardization exercises. In order to be deemed “standardized,” each person who was compared to Rajeswori had to achieve accuracy of 95% or greater on the standardization exercise. When these strict qualifications were not achieved, as was the case for 3 (2 for the MUAC and 1 for the HC standardization) DIMAG observers and 3 NNIPS-4 TLIs and ACs, the people in question had to repeat the standardization exercise until they were able to pass. In all cases, Rajeswori and the individuals in question were sent to the field to measure ten babies approximately the same age as the DIMAG and NNIPS-4 children. During Round 1 when Clinic A1 started late and we were forced to bring dozens of children into the office as a means of completing the round as scheduled, Menuka, one of the observers, was standardized with Rajeswori, so she could help to process all of the children coming through the clinic. She worked in the clinic for a previous NNIPS trial, so she knew what she was doing and did not have any problems with the standardization exercise. See Appendix for the standardization files.

**6.2 Observation**

Standardization exercises began before the baseline round of data collection and were resumed every three months after each round, prior to the next one. For the first exercise the observers watched multiple hour videos, during which they coded Shishir’s daughter’s interactions. They were supervised during each of these sessions to make sure they did not look at anyone else’s Visor or talk with the others about what they were seeing. When their data met the standardization criteria (kappa scores >70% for observations numbering > or = to15) when they were compared to Shishir using the CAOSBase program, they were considered “standardized.” For all of the subsequent standardization exercises, except the one after Round 1, the observers watched and coded multiple 30-minute segments of videotape. For each of these rounds, the observers had to match with each other before they were considered standardized.

The criteria were the same for the post-Round 1 standardization exercise; however, the observers were sent to the field in groups to observe infants not enrolled in DIMAG. Everyone had to match with everyone else to be considered “standardized”; however, due to technical difficulties and time constraints during this post-Round 1 standardization exercise, there were a few observers who did not match with a few others. Because the cases were few in number and the observers were able to match with others who matched with everyone else, the blank spaces on the standardization grid (see Appendix) were excused and all the observers were cleared to begin Round 2.

The post-Round 3 standardization video fell short of perfection, causing the observers to have difficulty discerning who initiated the vocalizations. For this reason, the observers were excused if they were able to achieve the standardization criteria for everything except the code “initiates with caretaker.” Problems with this code were marked with an “x1” in the standardization grid (see Appendix).

Similarly, there were problems with the post-Round 4 standardization video that made it difficult for the observers to tell whether social gesture was present. For this reason, the observers were considered standardized if they were able to achieve standardization on all the codes except this one. Problems with this code were marked in the standardization grid (see Appendix) by an x and the social gesture kappa score. For this last standardization exercise, Emily attempted to standardize the observers using multiple hour-long videos; however, she changed to using 30-minute videos after a week of work and problems with many of the observations. Both the 30- and 60-minute standardization grids are included in the appendix.

**6.3 Motor milestones**

The motor milestones standardization exercises were conducted at baseline, 6-months and approximately every three months until the end of NNIPS-4. The first post-data collection standardization exercise did not occur until 6-months after initial data collection for several reasons. First, the Ishwarpur WDs started collecting data at the beginning of the NNIPS-4 Batch 4 in December 2001, prior to the start of DIMAG. Second, with the study just beginning, we were not able to begin the standardization exercises until the end of Round 1. Third, the goal was to coordinate the observation and motor milestones standardization exercises to occur consecutively after each of the rounds of data collection.

For the first motor milestones standardization exercise, 4 NNIPS-4 ACs (area coordinators) visited 10 households containing children whose motor milestone data the WDs were collecting on a weekly basis. For subsequent standardization exercises, DIMAG observers did the work for the standardization exercises. The ACs and observers shadowed the WDs by asking the mothers the same question: has your child attained any of these (and described each milestone while showing them the corresponding picture on the form) milestones? They asked the questions as if they had never been asked before, drawing lines through the boxes of the milestones that the baby had attained and leaving the rest of the boxes blank. When the mothers were finished answering the question, the ACs/observers asked whether her child had achieved any of the milestones in the past 7 days? The implication of this question was that the answer would be no in most cases. If the mother answered in the affirmative, the AC recorded the present NNIPS week in the box of the appropriate milestone. If the mother responded by saying no, the AC/observer did not record any further data on the form. Before moving to the next house, the AC/observer observed the child to verify the highest milestone that the child had achieved. We recognized that the AC/observer might not have enough time in the house to observe some of the milestones, such as number 13 (jump), that are exhibited sporadically.

At the end of the day, the ACs/observers returned to the office and organized their forms by ward, sector, and household for Emily to record in the table of results. She made a table for each of the standardization exercises (see Appendix). The tables had the following categories: ward, sector, household, child’s NNIPS number, mother’s NNIPS number, number of the milestone the AC/observer observed the child performing, number of the milestone she reported to the AC/observer, NNIPS week that the AC/observer visited the house, number of the milestone that the mother reported to the WD as seen on the WDs MMWD, NNIPS week that the WDs form was checked, and comments. The motor milestones numbers that the mothers told the AC/observer were compared to the numbers that the mothers told the WD. The WDs were considered standardized if all of her numbers were within two (one above or below) numbers of the AC’s/observer’s. If the discrepancy between the numbers was greater than our criteria, the WDs were flagged for further observation. One (or more if needed to observe multiple WDs) observer was sent on a future Friday, the day that the WDs collect their motor milestone data, to watch the WD in question work. The observer was instructed to watch the WD and correct any problems she might be having.

After the earlier rounds the WDs required more supervision than they did after the later rounds. Four WDs required further training after Round 1. Observers worked with these WDs for 4 consecutive Fridays. Twenty WDs required supervision after Round; however, all of the WDs were followed by one of the observers to make sure that they were administering the form correctly. Three of the twenty-three required a second supervisory visit**.** None of the WDs exhibited problems with the data collection after Round 3, with the exception of one who was administering the form twice a week instead of once. This WD was instructed to only administer the form on Fridays. There were a few minor problems after Round 4. In the majority of the cases there seemed to be a discrepancy between what the children were able to do and what the mother reported. Because two of the observers were absorbed into NNIPS-4, we were not able to spare any observers to supervise the WDs. All of the WDs were supervised after Round 5 standardization exercise because there were so many WDs who qualified for a supervisory visit (16 of the 24 WDs) and Emily was leaving and wanted to make sure that everyone was observed an additional time while the DIMAG staff were present to offer guidance. The supervisory visits revealed that the WDs were making one of three mistakes: they were 1) neglecting to question the mother about the two codes proceeding the one that the mother reported the child had most recently attained; 2) administering the MMWD to a family member other than the mother; and/or 3) showing the mothers the pictures on the MMWD, but were not explaining what each of the codes means. Henceforth, the WDs will be supervised once every three months as they administer the MMWD.

**6.4 Clinic B: Fagan Test and A-not-B Task**

The description of the standardization procedures are embedded in the training section. The A-not-B standardization tools are included in the Appendix.

**6.4 Reliability for the Home Interview Form**

Beginning with Round 2, the observers redid a select number of Home Interview Forms 7 days after they fist administered the form. During Round 2 this number was 12 for a total of 180 repeat forms. Our target was 150. During Rounds 3 and 4 this number was reduced to 10 each. We would have reached our target had we not lost two of our observers to the larger NNIPS-4 trial during the middle of Round 3. The total number of repeat forms for Rounds 3 and 4 approximated 130 for each round. During Round 5 we realized that the number of forms during the previous two rounds fell short of our goal, so we increased the number of forms that each observer did to 12. At a minimum the percentage of forms that were repeated was 23%, a sufficient number for determining the reliability of the work our observers conducted in administering the questionnaire and the mothers responses to it.

For this reliability study the observers were instructed to only ask the person in the household to whom the previous DHIF in that round was administered. In most cases, this was the mother. In some it was the grandmother or another older female relative in the household, such as the aunt.

**7. ETHNOGRAPHY PROJECT**

Between July and October 2002 the DIMAG staff accepted the added responsibility of collecting ethnographic data to supplement the data already being collected for DIMAG. This was per Ernesto Pollit’s suggestion, and was an outgrowth of the first between-site mid-term meeting that was held in Davis, California at the beginning of May 2002. Tricia Kariger flew from the United States to assist with the beginning stages of the project. Together she and Emily developed a working plan that was adjusted and implemented in both Nepal and Pemba.

Emily spent 2 weeks in Pemba prior to returning to Nepal to train two staff members, Shishir and Kalawati, to conduct in-depth interviews. She and Tricia developed an ethnographic field guide for the interviewers to use while conducting their interviews. Extensive training in the qualitative interviewing technique was difficult due to the limitations caused by the time needed to produce a paper copy of the interviews. By the time the interviews were transcribed and translated, the interviewers had completed their work. Emily explained the need for the interviewers to use open-ended questions and follow-up on any of the respondents’ statements that might allow the interviewer to access further information. Shishir and Kalawati practiced asking me and other staff members questions before they began their official interviews. Shishir interviewed the male respondents, while Kalawati interviewed the females. Together they conducted 13 interviews with a Purohit, two traditional birth attendants, two teachers, and male and female Pahadi, Maithali, Muslim, and Tharu community members.

In addition to the in-depth interviews, DIMAG staff members questioned some of our DIMAG families about their child-care practices; perceptions of health and illness, poor and good nutritional status of children; and characteristics of play. These questions were open-ended and conducted in the style of a free-list, where the respondent was asked to list as many responses as applied to each question. Minimal training was needed for the free-list exercise. Tricia and Emily held a meeting with Shishir and the other DIMAG staff members to explain the nature of the project. The observers were then dispatched to collect their data.

The data from this project have been compiled in another location and were not included in the Appendix.

**8. LESSONS LEARNED**

While for the most part DIMAG ran smoothly, there were a few lessons that we learned only after starting data collection.

**8.1 Standardization exercises for the observation**

There are a number of advantages to using a video to standardize the observers. The primary advantage is that the person conducting the standardization has more control over the exercise, both by ensuring that each of the observers are observing the same behaviors at the same time and that there are a range of behaviors present. A secondary advantage is that it takes less time to standardize a group of observers using video than it does sending groups of observers to the field, especially if they are being compared to one another. Despite the advantages there are some significant problems with this technique. Unlike the field, observing a video (especially one presented on a small television screen such as ours) can cause the observers to strain their eyes. It is easy for them to miss some of the quick-action codes, such as social smile or social gesture when they turn their heads towards the Visor to code other behaviors that they saw. Because the observers do not mark their codes at the exact same time, it is natural to find a discrepancy between the number of social smile and social gesture episodes that were coded. Vocalizations are often hard to discern on a video. It is not always clear from what direction or from whose mouth the vocalization was uttered. For this reason, the observers often had problems reaching agreement with the vocalization codes, particularly initiates with caretaker code.

Although we initially tried to conduct 60-minute standardization exercises, we decided to reduce the length to 30-minutes after the observers complained of eyestrain. No matter how large the television was, watching it for a sustained period of time was both a difficult task and beyond what we were asking the observers to do in the field. We anticipated being able to standardize the observers in a single sitting; however, we discovered that it took up to 10 attempts for the entire group to achieve standardization. While we had had problems creating a video of good enough quality to use in both Nepal and Pemba for inter-site standardization purposes, the fact that we were not able to achieve standardization in a single sitting prevented us from being able to conduct the inter-site standardization exercise.

The first standardization exercise proved to be the most problematic. The primary reason for this was the fact that the observers had trouble taking the exercise seriously. As a part of the training, the observers were allowed to discuss the actions they were observing. When one of the observers had a problem, he was allowed to ask the others for help in judging a code. When it was time for the standardization exercise, the observers were so used to discussing the codes with each other that they experienced a lot of difficulty refraining from speaking. As a result and for fear of contamination, we chose to redo the first standardization exercise and control the task using a video instead of sending groups into the field. For each of the subsequent standardization exercises the observers were monitored to make sure they did not talk.

**8.2 Equipment**

DIMAG brought more technology to the field than had ever been used in a NNIPS project. Most of this equipment was used for the observation. The laptop computers, Visors, Actiwatches, and video camera held up surprisingly well considering the temperature extremes, dust, and fluctuations of electricity to which they were subjected. Nevertheless, we experienced a few mechanical failures with the Visors. Two suffered broken screens. One was rammed by a water buffalo and another was dropped. One of our DIMAG kids knocked a third out of an observer’s hand, while he was doing an observation. As a result, the Visor fell to the ground, the program froze, and we were not able to use it again. Fortunately, we were able to download the data before retiring it. Because we had many spare Visors, losing a few did not retard data collection.

The most significant problem that we experienced was the loss of five of our 14 Actiwatches. One leapt into the family’s cooking fire (or so we were told). Another was smashed by a rock. We suspect a curious neighborhood kid investigated the contents of the watch. Three were lost. One family handed our observer part of a band and a piece of the mechanical apparatus from the inside of the watch: the remains. We never found out what happened to the other two. One was lost on the day of the local bazaar. We suspect that an outsider walking through the area after going to the bazaar saw the watch, picked it up, and took it home. A boy living near one of our DIMAG kid’s homes used an instrument to pry open a sixth watch. We returned this one to Mini Mitter to be repaired and received it back in mint condition. While we anticipated having problems with a few of the watches, we did not expect that we would lose a third of our Actiwatch inventory. By losing these $800 watches the project lost both money and data. By the end of Round 5 only 8 Actiwatches remained, which meant as many as 5 DIMAG kids a day lacked Actiwatch data.

The major problem with the Actiwatches is the simple fact that we left them unattended for almost an entire day on active village kids. In most of the cases in which the Actiwatches were destroyed or lost, we suspect that people outside the family removed and destroyed (or otherwise disposed of) the watches. The observers suggested that we find a way to lock the watches to the kids should we repeat this data collection method in the future. One has to question the ethics of fastening a device to a child that the parents are not able to remove; however, the idea might be worth investigating. At the very least, employing shorter data collection intervals might help. Surely, a solution to this problem of loss can be found.

The laboratory equipment had been tested in the field during previous NNIPS studies. We did not have any problems with the HemoCue machines. However, we did have problems with the more sensitive AVIV flurometer. Initially, Emily experienced some problems trying to take it with her on the plane in December 2001. She was stopped at customs because she was not able to disprove the suspicion that the machine was a bomb by plugging the 220-volt machine into a 110-volt socket at the Atlanta airport. She ended up having to check the machine. The main problem that we had with this machine was with calibration. When we tested the controls, the calibrator values were not within the designated range. When we tested the calibrators, the controls were not within range. We sent the machine back to the US to be recalibrated prior to Clinic A1. We brought another machine from the US prior to Clinic A2 after the lab staff reported that the first machine’s values were out of range. The second machine turned out to be worse than the first. Because of the different voltage, we were not able to test it in the US prior to transporting it. In the end, we sent the second machine back to the US and fixed the first by using the screws on the back and control and calibrator samples and ranges to fine-tune it. These flurometers are sensitive pieces of equipment. The first sat for eight months without being used. The second traveled quite a distance through several East-coast cities in the US and across the Pacific Ocean before arriving in South Asia.

**8.3 Phlebotomy**

Of all the components of DIMAG we seemed to have the most trouble with Clinic A1. Initially, we met much resistance when we attempted to collect blood using a venous blood draw technique. The venous blood draw was very difficult for our lab workers to perform. Often they had to stick the child several times before they were able to find a vein. It was especially difficult for them to find a vein in nourished kids who actually had sub-cutaneous fat, opposed to the malnourished ones whose veins were easy to spot under their thin skin. What the lab workers needed was someone who could teach them how to draw blood from healthy kids. The training that they received in Kathmandu was not sufficient. We discussed bringing a doctor from Kathmandu down to supervise the blood collection; however, she was unable to come due to the unstable political situation. Many parents refused to allow us to take blood from their children using this technique. Of the ones that allowed us to proceed, there were many who were incredibly patient with our workers as they searched for a vein. It was painful watching the venous blood draws being performed. Due to the lack of the lab workers’ skill in this technique and the high number of refusals, we decided to change the method to a capillary blood draw. When we had problems with collecting enough blood to perform the lab tests we wanted, we changed the method again to the heel prick method both our lab workers and NNIPS families have grown accustomed to from other NNIPS studies.

Most of the problems that we had with the first Clinic A stemmed from the DIMAG kids’ parents not wanting us to take too much blood. Many people believe that it takes months to “grow” the blood that we were drawing in a matter of seconds. Drawing it would surely make their children weak. Some families (mostly those living in the Ward 2, Sector 1 toll) thought we were earning our salary from selling their children’s blood. There were more refusals when we were doing venous blood draws than capillary blood draws and heal pricks; however, many parents withdrew their consent after once we started the process and they were able to view the blood that we were taking. Some mothers and grandmothers went so far as to pull their children away from the lab technician once they saw the blood collecting. It did not matter that the capillary tubes were small and narrow. In their eyes we had already drawn enough blood for our needs. We had to change our technique for a second time when we were not able to draw enough blood to fill the capillary tubes and run the zinc and iron tests we had proposed employing. The heel prick was the only successful method that we used. Its success could be attributed to three reasons: only a few drops of blood are needed, it is easy to perform and fast.

We had a few problems with parents after we drew blood. Among those who gave their consent, there were a few who returned within a week to blame their child’s subsequent illness on the blood draw. These parents could not be convinced that the blood draw and child’s illness were not connected. When this happened one of the lab staff spoke with the parents, gave them medicine for the child, and sent them home. Most of these worried parents felt better once they had some medicine in their hands.

**9. APPENDICES**

**A. DIMAG staff**

*Staff based in the Pink Palace in Bailbas, Ishwarpur:*

Project Manager: Shishir Var Shrestha

Clinic Staff: Jaybar Shrestha, Kalawati Giri, Rajeswori Kafle, Chandeswor Nepali

Observer Team: Kesab Dhakal, Ishwori Shrestha, Ram Narayan Chaudhary, Nir Bahadur Karki, Padam Bahadur Lama, Kiran Chaudhary, Sulochana Chaudhary, Shyam Kumar Thapa, Khadga Bahadur Budhatholki, Menuka Chalise, Ram Aseshwor Chaudhary, Rudra Paudel, Yadav Mainali, Roshan Shrestha, and Dipak Ghimire

Support Staff: Parvati Malli, Udab Pahadi, and Shyam Sundar Chaudhary

Drivers: Krishna Bahadur Shrestha, Kumar Lama, and Pushpa Maharjan

*Assistance from the larger NNIPS-4 team*:

Ishwarpur WDs: (1-1) Mathura Karin, (1-2) Phul Maya Katuwal, (1-3) Tika Kumari Balampaki, (1-4) Ramita Lama, (2-1) Rina Shrestha, (2-2) Radha Devi Upreti, (2-3A) Kamala Thing, (2-3B) Bidyapati Devi Pasman, (3-1) Kebalbati Singh, (3-2) Urmila Chaudhary, (4-1) Sun Maya Lama, (4-2) Nirmala Lama, (5-1) Kiran Chaudhary, (6-1) Babita Chaudhari, (6-2) Jaypati Devi Mahato, (6-3) Renu Devi Sah, (7-1) Urmila Singh, (8-1) Sharda Chaudhary, (8-2) Ram Kumari Chaudhary, (9-1) Parbati Mainali, (9-2) Shyama Mishra, (9-3) Jamuna Badal, and (9-4) Bina Karki

Hariaun Staff: Tirtha Raj Shakya (Field Manager); Rabindra Shrestha (Assistant Field Manager); Gokarna Subedi, Uma Shankar Shah, Arun Bhetwal, and Durba Kharka (Supervisors); Bikram Tamang (AC assigned to Ishwarpur at the start of DIMAG); Dipak Upreti and Govinda (Tika) Niraula (TLIs assigned to Ishwarpur during the course of DIMAG)

Kathmandu Staff: While the entire Kathmandu staff contributed to DIMAG in one way or another, Shakuntala Singh was instrumental in helping us with a variety of word processor tasks, the most important of which was the development of our forms.

**B. Scope of work for DIMAG staff and timetable for DIMAG activities**

# Scope of Work for DIMAG Staff

January 2002 – May 2003

Shishir:

Responsibilities:

Manage scheduling of observations and interviews

Troubleshoot problems in the office and field

Download data daily from the Palms and Actiwatches

Edit all forms

Serve as a tester for Clinic B as needed

Observer Team:

Responsibilities:

Conduct 3-hour observations

Administer DHIF on days when not doing observations

Approach the family the day before the observation to notify them that they will be coming the next day and to place the Actiwatch on the infant.

Supervise WDs in NNIPS-4 upon occasion

Clinic Staff:

Responsibilities:

Conduct Clinic A1 (January 7, 2002 – March 31, 2002)

Conduct the A-not-B Task and Fagan Test testing for Clinic B

(January 7 – November 8, 2002)

Collect blood (heel prick) for neutropenia study*

[Jaybar & Chandeswor] (July 10-August 16, 2002)

# Collect blood (venous blood draw) for zinc study^

[Jaybar, Chandeswor, Kalawati, & Rajeswori]

(November 11 – January 13, 2002)

Conduct Clinic A2 (January 13, 2003- April 30, 2003)

*While Jaybar and Chandeswor will still be needed at the Pink Palace to conduct the Clinic B testing, they will work on Wednesday and Thursdays and whenever else they are free between July 10 and August 16 to complete the neutropenia study.

^The four members of the DIMAG lab staff will not have any work in the Pink Palace between November 11 and January 13, as Clinic B will be over and Clinic A2 will not have started.

Schedule of Data Collection Rounds:

Round 2: May 1 – July 31

Round 3: August 1 – October 31

Round 4: November 1 – January 31

Round 5: February 1 – April 30

Although the last infant’s window extends to May 22, 2003, we expect DIMAG to end at the beginning of May.

C. List of Equipment

| **DIMAG Equipment List** |  |  |
| --- | --- | --- |
| **Item** | **Number** | **Model Info.; Contact Info.** |
| Dell Latitude CPi Computer | 2 | Model #: PPL; Assy PN: 1421C |
| Desktop Computer for Fagan Test | 1 | Generic, Fagan Test Software |
| Hewlett Packard Deskjet 321 | 1 |  |
| Sony Handycam Video Recorder | 1 | PAL System |
| Ultra-Isolator Topaz | 1 | Output Voltage 120/240 |
| Stavolt | 2 | Model: SVC-1000N, 220V |
| Mini Mitter Actiwatch Reader | 2 |  |
| Mini Mitter Actiwatch | 14 | 198-0101-00 |
| Visor Cradle | 3 |  |
| Visor handheld | 19 |  |
| Psion Dacom External Modem | 1 | 56K + Fax |
| Etherfast Networking Card | 2 | Integrated Card Bus PC Card Linksys |
| Networking Cable | 1 |  |
| Energizer Rechargeable Batteries | 52 | AAA, 1.2V, 700mAh |
| Battery Charger | 7 |  |
| Panasonic Lithium Batteries | 24 | CR 2025, 3V |
| Surge protector | 1 |  |
| A-not-B Tables | 2 & 4 | Table: 90x30cm; Ht: 68cm; space b/w wells centered in table: 20cm; well diameter: 10cm; well depth: 6 cm |
| Cloth covers & toys for test | 4 & 8 | Locally available push cars and squeaky toys |
| Traceable Fingertip Timer | 2 | VWR International |
| UNICEF Seca Scale | 2 | Manufactured in Australia |
| Shorr Board | 2 | Recumbant Length and Height Board |
| Caliper | 2 | Holtain Ltd. |
| Ross Head Circumference Tape | 16 |  |
| MUAC Tape | 3 | Manufactured for JIVITA |
| Thermometer | 4 | Digital therometers, 1.55V |
| Hematoflurometer | 1 | AVIV Biomedical Inc. |
| Microscope Cover Glass | 40 x 10 oz. | AVIV Biomedical Inc. |
| Hemoglobin Photometer | 2 | HemoCue AB, Angelholm, Sweden |
| HemoCue Microcuvettes | 1200 |  |
| Hemo Lancets | 1800 | Vitron |
| Powderfree Nitrile Gloves | 2000 | Safeskin |
| Sterile Alcohol Prep Pads | 4000 | Fisherbrand |
| Coverlets (Bandaids) | 1200 |  |
| Centrifuge | 1 | Remi Company (Made in India) |
| Precision Microliter Pipette | 1 | Small size |
| Pipette Tips (Trace Metal Free) | 4800 | Fisherbrand (1-200 uL) #21-197-81C |
| Cryotube | 700 | Trace Mineral Free |
| Mutifly Needle | 1000 | Sarstedt |
| Monovette | 2208 | Sarstedt |
| Paper Labels & Satin Labels | 1900 each |  |
| Versi Dry | 2 rolls |  |
| Hero Hondo CD 100 Motorcycle | 2 |  |
| Toyota Land Cruiser | 1 |  |

### D. English forms and schedules

DIMAG Forms Overview

| **DIMAG Forms** |  |  |  |
| --- | --- | --- | --- |
| **Form #** | **Title** | **Abb.** | **Contents & Purpose** |
| Form 14-D | Consent Form-DIMAG | CFD | consent form for DIMAG Study |
| Form 14-N | Consent Form-Neuropenia Assessment | CFNA | consent form for Neuropenia assessment |
| Form 23 | Motor Milestones Worksheet-DIMAG | MMWD | record of 14 Motor Milestones achieved |
| Form 24 | DIMAG Home Interview Form | DHIF | infant feeding and development scales |
| Form 25 | DIMAG Home Observation Form | DHOF | morbidity, questions about observation |
| Form 26 | DIMAG Clinic A Form | DCAF | morbidity, anthropometry, phlebotomy |
| Form 27 | DIMAG Clinic B Form | DCBF | morbidity, Fagan and A-not-B Test results |
| Form 28 | DIMAG A-not-B Test Form | DABTF | place to record raw A-not-B test data |
| Form 29 | DIMAG Child Tracking Form | DCTF | basic tracking form |
| Form 30 | DIMAG Birthday Form | DBF | scheduling form for Clinic B |
| Form 33 | DIMAG Daily Clinic Schedule | DDCS | scheduling form |
| Form 34 | DIMAG Daily Home Interview Schedule | DDHIS | scheduling form |
| Form 35 | DIMAG Daily Home Observation Schedule | DDHOS | scheduling form |
| Form 38 | DIMAG Daily CAOS Log | DDCL | log of data downloaded |

NNIPS-4 Form 14 -D

7 January, 2002

**NNIPS-4 DIMAG CONSENT FORM**

WEEK: DATE:

dd mm yy

VDC: _____________________ WARD: SECT: HH:

# Consent Statement

Namaste ! I am a NNIPS staff person. Because you agreed to enroll your child in the zinc an iron tablet research project, your child is eligible to participate in an additional part of the study. We believe that zinc an iron may improve children's health, make their blood stronger in these nutrients and make them grow and develop better. We invite you to participate in this part of the study to see if this is true.

If you decide to take part, we will visit your house five times and transport you to our study office at the bazaar three to five times depending on your child's age. At the home visits, a NNIPS staff person will come to your house five times during the next year to observe you and your child for a period of three hours, while you go about your regular business. This staff person will also ask you some questions about the health of your child, your child's development, and his/her level of fussiness. This visit will take about 3.5 hours. The day before this home visit the same staff person will come to your house to fasten a watch to your child's left ankle. This watch will measure your child's activity for a period of 22-hours. It will be taken off the next day during the home visit. In the office, a staff person will measure your child's height, weight and upper arm circumference, will collect 3 milliliters of blood from your child's arm to test the level of zinc and iron in his/her blood, and will test your child's development. This visit will take about 2 hours. We will take you home after the office visit.

There are only very small risks for your child. Collecting blood may cause a small amount of pain and some bruising, but we will do it as quickly as possible and we will be sure to clean the area on his/her arm very well. Your child's participation is completely voluntary at all times. If for any reason, you do not want your child to continue with the visits for this study, you may withdraw him/her at any time. If you choose to participate or not, it will not affect your child being offered the tablets from the main part of the project. All information you provide will remain confidential and your name and other information will not be revealed to anyone who is not helping on this study. If you have any questions of problems regarding this study, I can answer them now or you can contact Dr. Subarna Khatry, NNIPS Office, Hariaun, Sarlahi (phone no. 29404).

0=Not Done

1=Done

Consent Process Conducted at Household:

Worker Signature: _____________________________________

NNIPS-4 FORM 24

16 May, 2002

# NNIPS-4 DIMAG Home Interview Form (DHIF)

Week: Date: Visit:

dd mm yy 1,2,3,4,5

#

# **Section A: Identifiers/Addresses**

# ISHWARPUR Ward: Sector: HH:

# Worker ID: _____________

NNIPS Number First and Middle Name Family Name

# Father: ________________________ ____________________

# Mother: _______________________ _____________________

# Child: _______________________ _____________________ 1=Male

# Date of birth: Age: mo. Sex: 2=Female

# dd mm yy

# Form Status: 1=Met

# 2=Not met

6=Refused

7=Permanently Moved Stop

8=Dead

# Relationship to child: 1=Mother

2=Father

3=Grandmother, Name: ____________________________

4=Other, Relationship: ________________________

Name: _____________________________

**Section B: Infant Feeding Questions**

In the past 7 days, has your child eaten….

Food Item: 0=No; 1=Yes

1. Breastmilk (from own mother or other mother)

1. Non-human milk (animal milk, powdered milk, yogurt/curd)
2. Water

1. Bhat/ Khichari

1. Roti

1. Dhirdo

1. Litto

1. Biscuits

1. Saag sabji/takari

1. Dahl

1. Achar

1. Fruits

1. Eggs

14. Meat/fish/chicken

# If only question number 1 or questions 1 and 3 were the only questions checked of the Infant Feeding Questions, this module may be skipped.

# Section C: Appetite Questions

0=No; 1=Yes

1. Child eats a lot for his age. ……………………………………………..
2. Child is usually happy or content when he is eating. …………………..
3. Child often asks for food. ……………………………………………..
4. Child is often fussy or irritable when he is eating. ……………………..
5. Child often refuses food or spits food out of his mouth. ……………….
6. Child often takes food from other people’s plates or from other sources.
7. Child eats frequently. …………………………………………………..
8. Child wants to eat only his favorite foods. …………………………….
9. Overall, is your child’s appetite: ……………………………………….

1. Good

2. So-so

3. Poor

# Section D: Parental Report Scales

# Begin with the first question and ask the mother whether the child is able to do the following skills. Questions with an asterix require the child to demonstrate the skill. Once five consecutive “0s” (No responses) have been coded, the interviewer may proceed to the next module.

MOTOR DEVELOPMENT SCALE 0=No; 1=Yes

1. She can pull to sit. Her head is steady and straight………………….

2. Raises head and chest when lying on stomach ………………………

3. Can turn and roll over ……………………………………………….

4. Holds head steady when held sitting ………………………………..

5. Makes crawling movements ………………………………………...

6. She can sit with the support of leaning against an object or person….

7. Sits without support …………………………………………………

8. She can raise her tummy off floor and support herself on

her hands and feet or knees…………………………………………

9. When she is lying on her stomach, with her head and chest raised,

she can move across the floor by using her arms and legs.

Her stomach remains on the floor…………………………….

1. She can crawl ………………………………………………………..
2. She is able to stand if she holds on to something or someone

to help support herself……………………………………………

1. She can walk when both hands are held …………………………….
2. She can walk with only one hand held ………………………………
3. She can stand for a moment on her own ……………………………

0=No; 1=Yes; Obs. Saw

1. She can stand alone for a long time …………………………………
2. She can bend down and straighten up again without falling ………..
3. She can take at least a few steps……………………. ………………

18. She can run ………….……………………………………………..

19. She can walk up steps …………………………………………….

20. She can throw a ball overhand ……………………………………

21. She can walk up and down steps ………………………………….

22. She can kick a ball forward ………………………………………..

23. She can walk forward along a straight line (10 paces)* ……………

24. She jumps with both feet ……………………………………………

25. She can stand on one foot for several seconds* ……………………

26. She can walk backward along a straight line (10 paces)* ………….

27. She can walk on tiptoe* ……………………………………………..

28. She can skip using alternate legs* ………………………………….

29. She can hop 20 times on one leg* …………………………………..

# LANGUAGE SCALE 0=No; 1=Yes

Makes sounds in play when alone …………………………………..

Makes sounds like da, ba, ga, ka, ma ……………………………….

Makes sounds like ma-ma, da-da, ba-ba ……………………………

Imitates single sounds like da, ba, ma, or repetitions ……………….

When he’s holding something and I ask him to give it to me he does

He can say 1 word ………………………………………………….

He points and makes some sounds when he wants something ……..

He can say 3 words …………………………………………………

If I ask him he can point to a dog ……………………………………

If I ask him he can point to a person that is walking ………………..

He can say 6 words …………………………………………………

He uses the words I , me, and you ………………………………….

He is constantly asking the names of objects ……………………….

He asks a lot of questions beginning “What?”, “Where?”, and “Who?”

He can say many words (20 or more) ………………………………

0=No; 1=Yes

He uses plurals when he speaks ………………………………………

17. He can tell me what a knife is for …………………………………..

1. He can tell people his full name ……………………………………….

He can tell me the opposite of the word “big” …………………………

He can talk about things that happened in the past ……………………..

# Section E: Temperament Questions

0=No; 1=Yes

1. When your baby gets upset, does he/she cry very loudly or intensely?

1. Does your baby’s mood change often and rapidly?
2. Is it easy for you to calm or soothe your baby when he/she is upset or crying?
3. Is it difficult for you to get your child to go to sleep?
4. Does your baby get fussy or irritable many times a day?
5. Does your baby cry and fuss more than the average baby?

7. Does your baby get upset easily?

8. Does your baby get very excited when people play with or talk to him/her?

0=No; 1=Yes

9. On the average, does your baby require a lot of attention,

more than the average baby?

10. When left alone, does your baby plays well by himself/herself?

11. Is your child very persistent in trying to get your attention when you are busy?

12. Overall, how easy or difficult is it to take care of this child?

1. Easier than average
2. Average
3. More difficult than average

## **Section F: Sleep**

1. Does your child usually sleep in the day time? 0=No (Go to 2)

1=Yes (Go to 1a.)

If yes,

1a. How many naps does he take?

1b. How long does he sleep during the day in all? . hours

2. How many hours does he usually sleep at night? . hours

2a. How many times during the night does he usually

wake up and cry/call for attention?

# NNIPS-4 FORM 25

# 14 March, 2002

# NNIPS-4 DIMAG Home Observation Form (DHOF)

Week: Date: Visit:

dd mm yy 1,2,3,4,5

#

# **Section A: Identifiers/Addresses**

# ISHWARPUR Ward: Sector: HH:

# Worker ID: _____________

NNIPS Number First and Middle Name Family Name

# Father: _______________________ ____________________

# Mother: _______________________ ____________________

# Child: _______________________ ____________________ 1=Male

# Date of birth: Age: mo. Sex: 2=Female

# dd mm yy

# Form Status: 1=Met

# 2=Not met

6=Refused

7=Permanently Moved Stop

8=Dead

# Relationship to child: 1=Mother

2=Father

3=Grandmother, Name: ________________________

4=Other, Relationship: ________________________

Name: _____________________________

**Section B: Morbidity Form**

Has your child experienced any of the following symptoms<

Today In the past 5 days?

0=No; 1=Yes 0=No; 1=Yes

Fever ……====================================== *

Cough ===========================================

Difficult/Rapid Breathing ……………….=== *

Watery Stool (4 or more)...==================== *

* If child has any of these symptoms today, reschedule.

## **Section C: Comments**

1. Has the Actiwatch been removed by you or anyone 0=No (Go to 2)

else in your house since it was fastened yesterday? 1=Yes (Go to 1a.)

2=No Actiwatch (Go to 2)

1a. If so, approximately when was it removed? : AM/ PM

0=No (Go to 2)

1b. If so, was it placed back on the child? 1=1=Yes (Go to 1c.)

1c. If so, approximately what time was it placed back on the child? :

AM/ PM

2. Was the observation completed? 0=No (Go to 2a.)

1=Yes (Go to 3)

If no,

2a. Why wasn't it completed ? 1=Baby fell asleep

2=Family had to go s0mewhere else outside our observation range

3=Other (please explain)_______________

2b. How many minutes did the observation last? minutes

2c. Will the child need to be re-scheduled

to complete the observation? 0=No; 1=Yes

3. How many primary caretakers were present during the observation?

3a. What was the relationship of each of these caretakers to the focus child?

1=Mother 4=Grandparent 7=Neighbour/Friend

2=Father 5=Uncle/Aunt 8=Other: __________

3=Older Sibling 6=Cousin 9=Don't know

4. How many peers were present during the observation?

4a. What was the relationship of each of these peers to the focus child?

1=Sibling

2=Cousin

3=Neighbour/Friend

4=Other: _________________

9=Don’t know

5. Where did the observation occur? 1=Outside (Go to 6)

2=Inside (Go to 6.)

3=Both outside and inside (Go to 5a.)

5a. If any, of the observation occurred inside, what percentage

of the total observation occurred inside? %

6. Did the observation occur in/around …. 1=Focus child's house

2=Another location :_____________

(Go to 6a.)

6a. If the observation occurred in another location, did the observer 0=No

follow the child from the child's house to the other location? 1=Yes

6b. If the observation occurred in another location, how far

was this location from the target child's house? km

7. How would you describe the weather on the day of the observation?

1=Pleasant

2=Hot

3=Cold

4=Raining

5=Other: ________________

8. Do you believe that the weather affected the child's activity? 0=No

1=Yes (Go to 8a.)

8a. Why do you think the weather affected the child's activities? _____________

9. Was your child's behavior typical today? 0=No; 1=Yes

10. Comments: _______________________________________________________

_______________________________________________________________________

_______________________________________________________________________

_______________________________________________________________________

_______________________________________________________________________

11. 1. . cm 2. . cm 3. . cm

# NNIPS-4 FORM 26 Blood Sample ID Label:

15 March, 2002

# NNIPS-4 DIMAG CLINIC A FORM (DCAF)

Week: Date: Visit

dd mm yy 1,2,3,4,5

#

# **Section A: Identifiers/Addresses**

# ISHWARPUR Ward: Sector: HH:

# Worker ID: _____________

NNIPS Number First and Middle Name Family Name

# Father: ________________________ ____________________

# Mother: ________________________ ____________________

# Child: _______________________ ____________________ 1=Male

# Date of birth: Age: mo. Sex: 2=Female

# dd mm yy

# Form Status: 1=Met

# 2=Not met

6=Refused

7=Permanently Moved Stop

8=Dead

# Relationship to child: 1=Mother

2=Father

3=Grandmother, Name: _____________________

# 4=Other, Relationship: ______________________

# Name: ______________________________

**Section B: Morbidity Form**

Has your child experienced any of the following symptoms<

Today In the past 5 days?

0=No; 1=Yes 0=No; 1=Yes

Fever ……====================================== *

Cough ===========================================

Difficult/Rapid Breathing ……………….=== *

Watery Stool ( 4 or more)..===================== *

* If child has any of these symptoms today, reschedule.

# Section C: Anthropometry

# Staff ID:

# MUAC (cm) : 1. . 2. . 3. .

# Head Circumference(cm): 1. . 2. . 3. .

# Tricep Skinfold Thickness (cm) : 1. . 2. . 3. .

# Weight(kg): 1. .

# Length(cm): 1. . 2. . 3. .

# Section D: Phlebotomy

# Staff ID:

# 1. Was a venous sample taken? 0=No (Go to 1b.)

# 1=Yes (Go to 1a.)

# 1a. If so, how much? . ml.

# 0=No (Stop)

# 1b. If no, was capillary sample taken? 1=Yes

#

# 1c. If yes, how many capillary tubes were filled?

# 2. Hemoglobin (g/dL):

# .

# 2a. If Hemoglobin <7.0 g/dL, give tonic. Tonic given: 0=No, 1=Yes

# 3. Protoporphyrin (μmol/no/heme):

# 4. Temperature . degrees Fahrenheit

# NNIPS-4 FORM 27

# 29 January, 2002

# NNIPS-4 DIMAG CLINIC B FORM (DCBF)

Week: Date: Visit:

# dd mm yy

# 1=39; 2=52 weeks

# **Section A: Identifiers/Addresses**

# ISHWARPUR Ward: Sector: HH:

NNIPS Number First and Middle Name Family Name

# Father: ____________________ _________________

# Mother: ____________________ _________________

# Child: ____________________ _________________

#

# Date of birth: Age: mo. Sex: 1=Male

# dd mm yy 2=Female

#

# Form Status: 1=Met

# 2=Not met

# 6=Refused Stop

# 7=Permanently Moved

# 8=Dead

# Relationship to child: 1=Mother,

# 2=Father,

# 3=Grandmother, Name: _________________________

# 4=Other, Relationship: __________________________

#

# Name: ________________________________

# Ethnic Group: 1=Pahadi

# 2=Madeshi

# 3=Muslim

# 4=Other __________________________________________

Section B: Morbidity Form

Has your child experienced any of the following symptoms<

Today In the past 5 days?

0=No; 1=Yes 0=No; 1=Yes

Fever ……====================================== *

Cough ===========================================

Difficult/Rapid Breathing ……………….=== *

Watery Stool ( 4 or more)..===================== *

* If child has any of these symptoms today, reschedule.

# Section C: Fagan Test

# 0=No

# Date: Date outside testing window? 1=Yes

# dd mm yy 1=First 1=AM

# Tester Staff ID: Order: 2= Second Time of Test: 2=PM

# 1. Was test completed ? 0=No (Go to 1a.)

# 1=Yes (Go to 2)

# 1a. If the test was not completed, what was the reason? 1=Baby closed eyes

# 2=Baby turned to breast

# 3=Baby looked away

# 4=Baby fussy

# 5=Other

# If other, please explain: __________________________________________________

# 2. Duration of Test: . minutes

# 3. Raw novelty Score: . 4. Scaled novelty Score: .

# 5. Comments about test: __________________________________________________

# Section D: A-not-B Test

# 0=No

# Date: Date outside testing window? 1=Yes

# dd mm yy

# 1=First 1=AM

# Tester Staff ID: Order: 2=Second Time of Test: 2=PM

# 1. Was test completed? 0=No (Go to 1a.)

# 1=Yes (Go to 2.)

# 1a. If the test was not completed, what was the reason? 1=Baby closed eyes

# 2=Baby turned to breast

# 3=Baby looked away

# 4=Baby fussy

# 5=Baby did not engage with

# tester

# 6=Baby not able to pick up

# cloth or toy

# 7=Other

# If other, please explain: _________________________________________________

# _____________________________________________________________________________

# 2. Duration of Test: . minutes

# 3. Did the child find the toy you expected ? 1=Fewer times than

# 2=About the same as

# 3=More times than

# 4. Comments:____________________________________________________________

# NNIPS-4 FORM 28

15 January, 2002 **NNIPS-4 DIMAG A-not-B Test Form (DABTF)**

Week: Date: Visit: 1=39 mo. 2=52 mo.

ISHWARPUR Ward: Sector: HH: WorkerID:

NNIPS Number First and Middle Name Family Name

# Child: ________________________ _____________________

# 1=First

# Date of birth: Order: 2=Second Duration of test: . dd mm yy

Put check if correct, an X if incorrect, NR if no reach, SC if self-correct, or PR if partial reach

| Trial | Starts-L | Starts-R | Toy | Reached @ hiding | Delay # sec | Used 1 box | Saw 2 boxes | SC or PR | Pause/ Stop | Reason for Pause/Stop * |
| --- | --- | --- | --- | --- | --- | --- | --- | --- | --- | --- |
| 1 |  |  |  |  |  |  |  |  |  |  |
| 2 |  |  |  |  |  |  |  |  |  |  |
| 3 |  |  |  |  |  |  |  |  |  |  |
| 4 |  |  |  |  |  |  |  |  |  |  |
| 5 |  |  |  |  |  |  |  |  |  |  |
| 6 |  |  |  |  |  |  |  |  |  |  |
| 7 |  |  |  |  |  |  |  |  |  |  |
| 8 |  |  |  |  |  |  |  |  |  |  |
| 9 |  |  |  |  |  |  |  |  |  |  |
| 10 |  |  |  |  |  |  |  |  |  |  |
| 11 |  |  |  |  |  |  |  |  |  |  |
| **12** |  |  |  |  |  |  |  |  |  |  |
| 13 |  |  |  |  |  |  |  |  |  |  |
| 14 |  |  |  |  |  |  |  |  |  |  |
| **15** |  |  |  |  |  |  |  |  |  |  |
| 16 |  |  |  |  |  |  |  |  |  |  |
| 17 |  |  |  |  |  |  |  |  |  |  |
| 18 |  |  |  |  |  |  |  |  |  |  |
| 19 |  |  |  |  |  |  |  |  |  |  |
| 20 |  |  |  |  |  |  |  |  |  |  |
| 21 |  |  |  |  |  |  |  |  |  |  |
| 22 |  |  |  |  |  |  |  |  |  |  |
| 23 |  |  |  |  |  |  |  |  |  |  |
| 24 |  |  |  |  |  |  |  |  |  |  |
| 25 |  |  |  |  |  |  |  |  |  |  |
| 26 |  |  |  |  |  |  |  |  |  |  |
| 27 |  |  |  |  |  |  |  |  |  |  |

# 3. Did your child find the toy ____________ you expected? 1= Less often than 2= As many times as

# 3= More often than

NNIPS-4 Form 33

14 January, 2002

# **NNIPS-4 DIMAG Daily Clinic Schedule (DDCS)**

NNIPS Week: Day No.: Clinic: A/B

Form

Ward Sect HH Father's name Child's NNIPS# Name Age (mo) Visit# Completed Comments

__________________ ________________ __________

__________________ ________________ __________

__________________ ________________ ___________

_________________ ________________ ___________

_________________ ________________ ___________

_________________ ________________ ___________

_________________ ________________ __________

_________________ ________________ __________

_________________ ________________ __________

_________________ ________________ __________

_________________ ________________ ___________

_________________ ________________ __________

_________________ ________________ ___________

NNIPS-4 Form 34

14Janurary, 2001

# **NNIPS-4 DIMAG Daily Home Interview Schedule (DDHIS*)***

ISHWARPUR NNIPS Week: Day No.:

Age Form

Ward Sect HH Father's name Child's NNIPS# Name (mo) Worker Complete Comments

______________ ______________ ________________

______________ ______________ ________________

______________ ______________ ________________

______________ ______________ _______________

______________ ______________ _______________

______________ ______________ _______________

______________ ______________ _______________

______________ ______________ _______________

______________ ______________ _______________

______________ ______________ _______________

______________ ______________ _______________

______________ ______________ _______________

______________ ______________ _______________

NNIPS-4 Form 35

24Janurary, 2001

# **NNIPS-4 DIMAG Daily Home Observation Schedule (DDHOS)**

Int. Schedule:

ISHWARPUR NNIPS Week: Day No.: Clinic A Schedule:

Age

Ward Sect HH Father's name Child's NNIPS# Name (mo) Worker A.W. Obs. Duration Comments

_____________ _____________ _____

_____________ _____________ ______

____________ ____________ ______

____________ ____________ ______

____________ ____________ ______

____________ ____________ ______

____________ ____________ ______

____________ ____________ ______

____________ ____________ ______

____________ ____________ ______

____________ ____________ ______

____________ _____________ ______

____________ _____________ ______

NNIPS-4 Form 38

9 January, 2002

# **NNIPS-4 DIMAG DAILY CAOS LOG (DDCL)**

NNIPS Week: Day: Completed by**:**

Palm ID Observer ID Child ID Watch Serial Number #Obs Length Data Pts Notes

### P02 02 V63

### P02 02 V63

### P02 02 V63

### P02 02 V63

### P02 02 V63

### P02 02 V63

### P02 02 V63

### P02 02 V63

### P02 02 V63

P02 02 V63

P02 02 V63

P02 02 V63

Daily Backup (to Floppy Disk) Compact Database (Monday) Data verified with schedule

Bi-Weekly Backup (Base Station) Weekly Backup to Kathmandu

# E. Nepali forms

NNIPS-4 Form 14 -D

7 January, 2002

**NNIPS-4 DIMAG CONSENT FORM**

WEEK: DATE:

dd mm yy

VDC: _____________________ WARD: SECT: HH:

Consent Statement

gd:sf/ ! d afn kf]if)f of]hgfsf] sd{rf/L x"+ . tkfO{n] tkfO{sf] aRrf h:tf / nf]xf kf}li^s tTjsf] cWoog sfo{qmddf ;xefuL ug"{ ePsf] sf/)faf^ tkfO{sf] aRrf csf]{ yk cWoog sfo{qmddf ;xefuL x"g of]Uo ePsf] % . xfdLnfO{ ljZjf; % sL h:tf / nf]xf kf}li^s tTjx?n] aRrfx?sf] :jf:Yo ;"wf/ u%{, pgLx?sf] /utdf oL kf}li^s tTjx?sf] dfqf a(\% h;n] ubf{ pgLx? /fd|/L x"s{g / ljsl;t x"g ;Sb%. xfdL tkfO{nfO{ o;sf] tYo s"/f kQf nufpg o; sfo{qmddf ;xefuL x"g lgDTofp+%f}+ .

olb tkfO{ ;xefuL x"g] ePdf xfdL tkfO{sf] #/df 5 k^s cfpg] %f}+ / tkfO{nfO{ ahf/df cjl:yt xfd|f] clkm;df tkfO{sf] aRrfsf] pd]/ cg";f/ 3-5 k^s uf*Ldf n}hfG%f}+ . tkfO{sf] #/b}nf]df xfd|f] sfo{stf{ cfp+bf] jif{e/Ldf 5 k^s cfpg"x"G% / tkfO{ cfkm\gf] ;fljs #/sf] sfd u/L/x+bf tkfO{ / tkfO{sf] aRrfnfO{ 3 #)^f;Dd lgofn]/ x]/L a:% . pSt sfo{stf{n] tkfO{sf] aRrfsf] :jf:Yo jf/], aRrfsf] ljsf; jf/] / pgsf] lk/f]Ng] jfgL cfbL jf/] tkfO{;+u s]xL k|Zgx? ;f]Wg] % . oL ;j ug{nfO{ s/La 3 1/2 #)^f hlt nfUb% . ;f]xL sfo{stf{ of] cjnf]sg ug{ cfpg"eGbf Ps lbg cuf*L tkfO{sf] aRrfsf] jf+of uf]nL uf+&f]lg/ Pp^f #*L af+Wg tkfO{sf] #/ cfpg] %. of] #*Ln] tkfO{sf] aRrfsf] 22 #)^f;Ddsf] lqmofsnfksf] n]vfhf]vf ug]{ % . csf]{ lbg pgL #/df cfp+bf o;nfO{ lemSb% . clkm;df n}hf+bf Ps hgf sfo{stf{n] tkfO{sf] aRrfsf] prfO{, jhg, dflyNnf] kfv"/fsf] uf]nfO{ gfKb% / aRrfsf] /utdf h:tf / nf]xf slt dfqfdf % egL hf+Rg cfwf rdr -3 dL=nL=_ hlt /ut lemSb% / aRrfsf] ljsf; jf/] hf+Rb% . oL ;j} sfd k'/f ug{ s/La 2 #)^f hlt nfUb% . xfdL tkfO{x?nfO{ clkm;af^ km]/L #/ k"/\ofO{ lbG%f}+ .

o; sfo{df tkfO{sf] aRrfnfO{ Psbd} sd dfq vt/f % . /ut ln+bf clnslt b"Vg ;S%, cln cK&\of/f] x"g;S% / ;fgf] *fd a:g ;S% t/ xfdL of] ;a} ;s];Dd rf+*f] u%f}{ / xfdL pgsf] kfv"/f /fd|/L ;kmf u%f}{+ . tkfO{sf] aRrfsf] ;xefuLtf ;b}j k")f{tof :j}lR%s /xg] %. s"g} sf/)fjz tkfO{nfO{ o; cWoogdf /xL /xg dg gnfu]df s"g} klg a]nf tkfO{ lg:sg ;Sg" x"G% . o; cWoogdf tkfO{ ;xefuL eP klg gePklg tkfO{sf] aRrfn] csf]{ sfo{qmdaf^ kfpg] kf}li^s tTjx? kfO g} /xg] %. tkfO{n] lbPsf] ljj/)fx? ;j} uf]Ko /xg]% / tkfO{sf] gfd jf c? kl/rofTds ljj/)fx? o; cWoogdf g/x]sf] s;}nfO{ klg lbOg] %}g . tkfO{nfO{ c? s]xL ;f]Wg" % jf o; cWoog jf/] s]xL ;d:ofx? % eg] d clxn] g} tkfO{nfO{ k|i^ ug{ ;S%" cyjf afn kf]if)f sfof{no, xl/cf}g -kmf]= g+= 29404_df xfd|f] lgb]{zs *f= ;"j)f{ s"= vqL;+u ;Dks{ ug{ ;Sg" x"G% .

0=Not Done

1=Done

Consent Process Conducted at Household: Worker Signature:

# NNIPS-4 FORM 24

10 May, 2002

**NNIPS-4 DIMAG Home Interview Form (DHIF)**

Week: Date: Visit:

# dd mm yy 1,2, 3, 4, 5

# ***Section A: Identifiers/Addresses***

# ISHWARPUR Ward: Sector: HH:

# Worker ID: _____________

NNIPS Number First and Middle Name Family Name

# Father: _________________ ___________________

# Mother: __________________ _________________

# Child: ___________________ _________________

#

# Date of birth: Age: mo. Sex:

# dd mm yy 1=Male;2=Female

# Form Status: 1=Met

# 2=Not met

6=Refused

7=Permanently Moved Stop

8=Dead

# Relationship to child: 1=Mother

2=Father

3=Grandmother, Name: ________________________

4=Other, Relationship: _________________________

Name: ______________________________

# Section B: Infant Feeding Questions

ut 7 lbgdf tkfO{sf] aRrfn] tnsf vfg]s"/fx? vfPsf] lyof] <

vfg]s"/fM 0=lyPg/1=lyof]

1. :tgkfg -cfkm\g} cfdf jf csf]{ cfdf_ =========================

2. b'w -ufO{, e}+;L, afv|L, kfp*/ b'w jf bxL_ ==================

3. kfgL =====================================================================

4. eft/lvr*L ============================================================

5. /f]^L =====================================================================

6. l(+(f] ====================================================================

7. ln^f] ====================================================================

8. lj:s"^ =================================================================

9. ;fu ;AhL/t/sf/L ==================================================

10. bfn ====================================================================

11. crf/ ==================================================================

12. kmnkm"n ===============================================================

13. c)*f ==================================================================

14. df;"/df%F =============================================================

gf]^M olb o; v)*sf] k|Zg g+= 1 dfq} jf k|Zg g+= 1 / 3 sf] dfq} hjfkm "1" ePdf "Section C-Appetite Questions" ug"{ kb}{g .

# **Section C: Appetite Questions**

0=vf+b}g/1=vfG%

1. aRrfn] cfkm\gf] pd]/ cg";f/eGbf a(L vfG% < ====================

2. aRrf /dfO{ /dfO{ -v"zL;fy_ vfG% < ================================

3. aRrf ykL ykL/dfuL dfuL vfG% < ===================================

4. aRrf vfgf dg gnfuL gnfuL/emsf]{ dfg]/ vfG% < ==============

5. aRrf vfgf vfg dfGb}g -d"vdf /fv]sf] vfgf cf]slnlbG%_ < ==

6. aRrfn] c?sf] yfn/ef+*fs"+*faf^ jf c? &fp+af^ vfg]s"/f

cfkm}n] lems]/ vfG% < =====================================================

7. lbge/Ldf aRrfn] l%g-l%gdf vfg]s"/f dfu]/ vfG% < ===========

8. aRrfn] cfkm"nfO{ dg kg]{ vfg]s"/f dfq} vfg vf]H% < ==========

9. aRrfsf] ?rL s:tf] % < ===============================================

1=/fd|f]

2=l&sl&s} -;fdfGo_

3=;fdfGoeGbf sd

### Section D: Parental Report Scales

klxnf] k|Zgaf^ z"? ug"{xf];\ / cfdfnfO{ pgsf] aRrfn] lgDg lqmofx? ug{ ;S% egL ;f]Wg"xf];\ . tf/f lrGx (*) nufOPsf] k|Zgx?df aRrfnfO{ ug{ nufP/ x]g"{xf];\ . nuftf/ kf+rj^f k|Zgx?sf] hjfkm "0" ePdf o; v)*sf af+sL k|Zgx? gu/Lsg} csf]{ v)*df hfg"xf];\ .

## Motor Development

0=xf]Og/1=xf]

1. aRrf pQfgf] k/]/ ;"t]sf] a]nf b"a} xft ;dft]/ p&fp+bf

^fpsf] l:y/ / ;f]emf] /fVg ;S% . ========================================

2. #f]K^f] k/]sf] a]nf k]^sf] cf*df ^fpsf] / %flt p&fp+% . ==========

3. df]*\g / u"*\g -klN^+b} hfg_ ;S% . =======================================

4. a;]sf] a]nfdf ^fpsf] l:y/ /fVg ;S% . ==================================

5. jfd] ;/]sf] h:tf] rfn u%{ . ================================================

6. s"g} JolSt jf lrhsf] cf* lnP/ a:g ;S% . ========================

7. ljgf cf*df a:g ;S% . =====================================================

8. xft / v"^\^f jf #"+*fsf] cf* lnP/ aRrfn] cfkm\gf] k]^

e"O{af^ p&fpg ;S% . ==========================================================

9. #f]K^]/ klN^/x]sf] a]nfdf ^fpsf] / %ftL e"O{df g%f]Osg

xft v"^\^f rnfP/ e"Odf ;g{;S% . p;sf] k]^ e"O{df

%f]O/x]sf] x"G% . ==================================================================

10. pgL jfd] ;%{ . ………………………………………………………….

11. s"g} lrh of ;fdfg ;dft]/ -cf* lnP/_ cfkm} pleg ;S% . =====

12. p;sf] b"a} xft ;dftL lbof] eg] lx+*\g ;S% . ……………………..

13. p;sf] Pp^f xft dfq ;dftL lbof] eg] lx+*\g ;S% . …………….

14. pgL cfkm} Ps}l%g pleg ;S% . ………………………………………

15. pgL w]/} a]/;Dd ple/xg ;S% . ……………………………………..

16. pgL cuf*L em"s]/ gn*Lsg km]/L &f*f] x"g ;S% . ……………….

0=xf]Og/1=xf]

17. pgL s]xL kfOnf lx+*\g ;S% . ………………………………………

18. pgL bu"g{ ;S% . ………………………………………………………

19. pgL v"*\sLnfdf -e/\ofªdf_ r(\g ;S% . ………………………….

20. pgL xftn] an kmfNg ;S% . ……………..………………………..

21. pgL e/\ofª\df tn dfly ug{ ;S% . ………………………………

Interviewer n]

22. pgn] v"^\^fn] an cuf*L xfGg ;S% . …………..…………..….. b]v]sf]/gb]v]sf]

23. pgL bz kfOnf;Dd ;f]em} lx+*\g ;S% .* ……………………….... 0=gb]v]sf]

1=b]v]sf]

24. pgL b"j} v"^\^fn] pk|mg ;S% . …………………………………..…

25. pgL Pp^f v"^\^fn] s]xL a]/;Dd -s]xL ;]s])*;Dd_

ple/xg ;S% .* ………………………………………….……………

26. pgL bz kfOnf;Dd ;f]em} k%f*Llt/ lx+*\g ;S% .* …………..….

27. pgL k}tfnfsf] k~hfn] dfq -cf}+nfx?n] dfq ^]s]/_

klg lx+*\g ;S% .* ………………………………………..…………

28. pgL pk|mL pk|mL v"^\^f km]g{ ;S% .* …………………………….….

29. pgL Pp^} v"^\^fn] 20 k^s;Dd pk|mg ;S% .* ……………..….

*;Dej ePdf aRrfnfO{ u/fpg"xf];\ .

# Language Scale 0=xf]Og/1=xf]

1. PSn} v]Nbf cfjfh lgsfN% . =============================================

1. bf, af, uf, sf, df, h:tf] cfjfh lgsfN% . ========================

0=xf]Og/1=xf]

3. dfdf, bfbf, afaf, h:tf] zAb lgsfN% . ===============================

4. bf, af, df, h:tf Pp^f dfq cIf/ ePsf

zAbx?sf] gSsn u%{ jf bf]x/\ofp+% . ================================

5. pgn] s"g} lrh lnO{ /fv]sf] a]nfdf c?n] Tof] lrh

dfUof] eg] lbG% . ===========================================================

6. pgL s"g} Pp^f zAb eGg ;S% . ……………………………...

7. pgn] s"g} lrh rflxPsf] a]nfdf To;nfO{ b]vfP/

cfjfh lgsfNg ;S% .…………………………….………………...

8. pgL tLg j^f zAbx? eGg ;S% . ………..…..………………..

9. tkfO{n] ;f]Wg" eof] eg] p;n] s"s"/ b]vfpg ;S% . ………….

10. tkfO{n] pgL;+u ;f]Wg"eof] eg] pgn] lx+l*/x]sf]

dflg;nfO{ b]vfpg ;S% . …………………………………………

11. pgn] s"g} % j^f zAbx? eGg ;S% . ……………………….

12. pgL d, dnfO{ / ltdL zAbx? k|of]u ub{% . …………………

13. pgL nuftf/ lrhaLhx?sf] gfd ;f]lw/xG% . ………………….

14. pgL s], sxf+ / sf] egL w]/} k|Zgx? ;f]lw/xG% . …………..

15. pgL 20 jf Tof] eGbf a(L zAbx? eGg ;S% . ….…………

16. pgL af]Nbf ax" arg (plurals) k|of]u ub{% . …………………..

17. pgn] rSs" s] sf] nflu xf] eGg ;S% . ……………………..

18. pgn] c?nfO{ cfkm\gf] k'/f gfd eGg ;S% . ======================

19. pgn] "&'nf]" eGg] zAbsf] pN^f] zAb eGg ;S% . …………..

20. pgL ljlt;s]sf] #^gfx? jf/] s"/f ug{ ;S% . ………………

# Section E: Temperament Questions

0=xf]Og/1=xf]

1. tkfO{sf] aRrf lvGg ePsf] a]nfdf, w]/} &'nf]

cfjfh u/L jf rsf]{ :j/df ?g] ub{% < ==============================

2. tkfO{sf] aRrfsf] dgsf] efjgf #/L #/L

/ l%^f] l%^f] ablnG% < ===================================================

3. tkfO{sf] aRrf lvGg ePsf] jf /f]O/x]sf]

a]nfdf zfGt jf v"zL kfg{ ;lhnf] % < ===========================

4. tkfO{sf] aRrfnfO{ ;"tfpg ufx|f] % < ================================

5. tkfO{sf] aRrf lbgdf w]/} k^s srsr ug]{

jf l/;fO/xg] ub{% < ====================================================

6. tkfO{sf] aRrf ;fwf/)f aRrfeGbf a(L

?g] jf lk/f]Ng] u%{ < ====================================================

7. tkfO{sf] aRrf l%^} -;lhn}_ lvGg x"G% < ==========================

8. tkfO{sf] aRrf;+u c? dflg;x?n] v]Nbf jf af]Nbf

w]/} pQ]lht -/dfpg]_ x"G% < ===========================================

9. ;/b/df tkfO{sf] aRrfnfO{ c? ;fdfGo aRrfnfO{

eGbf a(L Wofg lbg" cfjZos kb{% < =============================

10. PSn} %f*L /fVbf tkfO{sf] aRrf cfkm} dhf;+u v]N% < ========

11. tkfO{ Jo:t /x+bf tkfO{sf] aRrf tkfO{sf] Wofg

kfpg w]/} lh$L u%{ < ==================================================

12. ;a}s"/fsf] ljrf/ ubf{ of] aRrfsf] :ofxf/ ug{

;lhnf] % sL ufx|f] % < ================================================

1. ;fwf/)feGbf ;lhnf] % .

2. cf};t % . &Ls&Ls} % .

3. ;fwf/)feGbf ufx|f] % .

## **Section F: Sleep**

1. tkfO{sf] aRrf ;+w}h;f] lbp+;f] ;"T% < 0=;"Tb}g -k|=2 df hfg"xf];\_

1=;"T% -k|=1a. df hfg"xf];\_

olb ;"T% eg],

1a. Ps lbgdf slt k^s ;"T% (naps)?

1b. Ps lbgdf hDdf slt #)^f ;"T% < . #)^f

2. /ftdf w]/}h;f] hDdf slt #)^f ;"T% < . #)^f

2a. /ftdf w]/}h;f] slt k^s p&]/ ?G% cyjf

tkfO{sf] Wofg cfsif{)f ub{% <

# NNIPS-4 FORM 25

# 16 May, 2002

# NNIPS-4 DIMAG Home Observation Form (DHOF)

Week: Date: Visit:

# dd mm yy 1,2,3,4,5

# **Section A: Identifiers/Addresses**

# ISHWARPUR Ward: Sector: HH:

# Worker ID: _____________

NNIPS Number First and Middle Name Family Name

# Father: _______________ ___________________

# Mother: ________________ ___________________

# Child: __________________ _________________

#

# Date of birth: Age: mo. Sex:

# dd mm yy 1=Male;2=Female

# Form Status: 1=Met

# 2=Not met

6=Refused

7=Permanently Moved Stop

8=Dead

# Relationship to child: 1=Mother

2=Father

3=Grandmother, Name: _________________________

4=Other, Relationship: __________________________

Name: _______________________________

**Section B: Morbidity Form**

1. tkfO{sf] aRrfnfO{ tnsf nIf)fx?dWo] s"g} b]lvPsf] lyof] <

cfh ut 5 lbgdf

0=%}g; 1=% 0=lyPg; 1=lyof]

Hj/f] ========================================================== *

vf]sL =========================================================

:jf; km]g{ ufx|f]/l%^fl%^f] :jf; kmg]{ ================= *

kfgL h:tf] kftnf] lb;f -4 jf a(L k^s_=========== *

* olb aRrfnfO{ cfh of] nIf)f b]lvPdf km]/L e]^\g] sfo{qmd agfpg"xf];\ .

**Section C: Comments**

1. lxhf] af+w]b]lv of] #*L (actiwatch) tkfO{ jf 0=lyPg -k|= 2 df hfg"xf];\_

#/sf] c? s;}n] km"sfNg" ePsf] lyof] < 1=lyof] -k|= 1a. df hfg"xf];\_

2=(actiwatch) gaf+lwPsf]

(k|= 2 df hfg"xf];\)

olb lyof] eg],

1a. cGbfhL slt ah]lt/ km"sflnPsf] lyof] < M -;do_ AM/PM

1b. #*LnfO{ km]/L af+lwPsf] lyof] < 0=lyPg -k|= 2 df hfg"xf];\_

1=lyof] -k|= 1c. df hfg"xf];\_

olb lyof] eg],

1c. cGbfhL slt ah]lt/ #*L km]/L M -;do_ AM/PM

af+lwPsf] lyof] <

2. cjnf]sg k'/f ePsf] lyof] < 0=lyPg -k|= 2a. df hfg"xf];\_

1=lyof] -k|= 3 df hfg"xf];\_

olb lyPg eg],

2a. lsg k'/f gePsf] < 1=aRrf ;"t]sf]n]

2=#/kl/jf/nfO{ cjnf]sg If]q

eGbf aflx/ hfg" k/]sf]n]

3=cGo, :ki^ ug"{xf];\ _________

______________________________________________________________

2b. hDdf slt ldg]^ cjnf]sg ug{" eof] < ldg]^

0=kb}{g;

2c. cjnf]sg k'/f ug{sf] nflu km]/L sfo{qmd agfpg" kb{%< 1=k%{

3. cjnf]sgsf] a]nfdf slt hgf aRrfnfO{ x]/rfx ug]{ JolStx?

(caretakers) pkl:yt lyP <

3a. tL pkl:yt JolStx?sf] aRrf;+usf] ;DaGw -gftf_ s] xf] <

1=cfdf 5=sfsf, dfdf, &'nf]a"af/sfsL, dfOh', &'nLcfdf

2=afa" 6=km"km", sfsf, &'nf] a"af, dfdfsf] %f]/f%f]/L

3=cfkm\g} bfh"efO{/lbbL alxgL 7=l%d]sL /;fyL

4=afh]ah} 8=cGoM ___________________

9=yfxf %}g

4. cjnf]sgsf] ;dodf slthgf pxL pd]/sf aRrfx? (peers) pkl:yt lyP <

4a. oL aRrfx?sf] cjnf]sg ul/Psf] aRrf;+usf] ;DaGw -gftf_ s] xf] <

1=cfkm\g} bfh"efO{/lbbL alxgL 4=cGo: ______________

2=km"km", sfsf, &'nf] a"af, dfdfsf] %f]/f%f]/L 9=yfxf %}g .

3=l%d]sL /;fyL

5. cjnf]sg sxf+ ul/Psf] lyof] < 1=#/aflx/ -k|= 6. df hfg"xf];\_

2=#/leq -k|= 6. df hfg"xf];\_

3=b"a} -k|= 5a. df hfg"xf];\_

5a. cjnf]sgsf] s"g} efu #/leq klg ul/Psf] lyof] eg],

slt k|ltzt cjnf]sg #/leq ul/Psf] lyof] < %

6. cjnf]sg ePsf] &fp+ …….. 1=cjnf]sg ul/Psf] aRrfsf] #/

2=cs}{ &fp+ : ________________

-k|= 6a. df hfg"xf];\_

6a. olb cjnf]sg cs}{ &fp+df ePsf] eP, cjnf]sg stf{n] aRrfnfO{ 0=xf]Og

p;sf] #/b]lv To; &fp+;Dd k%\ofp+b} uPsf] xf] < 1=xf]

6b. olb cjnf]sg cs}{ &fp+df ePsf] eP, To; &fp+

cjnf]sg ul/Psf] aRrfsf] #/eGbf slt ^f(f %< = ls=dL=

7. cjnf]sg ul/Psf] lbgsf] df};d s:tf] lyof] <

1=/fd|f] df};d 4=kfgL k/]sf]

2=udL{ 5=cGo, k|i^ ug"{xf];\; ___________________

3=hf*f]

8. tkfO{sf] ljrf/df aRrfsf] lqmofsnfknfO{ 0=lyPg

df};dn] c;/ kf/]sf] lyof] < 1=lyof] -k|= 8a. df hfg"xf];\_

8a. lsg tkfO{nfO{ aRrfsf] lqmofsnfknfO{ df};dn] c;/ kf/]sf] h:tf] nfUb%<

_____________________________________________________________________

_____________________________________________________________________

9. tkfO{sf] aRrfn] cfh ;+w}sf] h:tf] 0=%}g

;fdfGo Jojxf/ u/L/x]sf] % < 1=%

10. l^Kk)fLM ____________________________________________________________

___________________________________________________________________

11. ^fpsf]sf] uf]nfO{M 1. . cm 2. . cm 3.

# NNIPS-4 FORM 26 Blood Sample ID Label:

15 March 2002

# NNIPS-4 DIMAG CLINIC A FORM (DCAF)

Week: Date: Visit#: 1 or 2

# dd mm yy

# ISHWARPUR Ward: Sector: HH:

# Section A: Identifiers/Addresses

NNIPS Number First and Middle Name Family Name

# Father: _________________ _____________________

# Mother: __________________ ____________________

#

# Child: __________________ ____________________

# 1 =Male

# Date of birth: Age: mo. Sex: 2=Female

# dd mm yy

# Form Status: 1=Met

# 2=Not met

6=Refused

7=Permanently Moved Stop

8=Dead

# Relationship to child: 1=Mother

2=Father

3=Grandmother, Name: __________________________

4=Other, Relationship: __________________________

Name: _______________________________

### Section B: Morbidity Form

1. tkfO{sf] aRrfnfO{ tnsf nIf)fx?dWo] s"g} b]lvPsf] lyof] <

cfh ut 5 lbgdf

0=%}g; 1=% 0=lyPg; 1=lyof]

Hj/f] ========================================================== *

vf]sL =========================================================

:jf; km]g{ ufx|f]/l%^fl%^f] :jf; kmg]{ ================= *

kfgL h:tf] kftnf] lb;f -4 jf a(L k^s_=========== *

* olb aRrfnfO{ cfh of] nIf)f b]lvPdf km]/L e]^\g] sfo{qmd agfpg"xf];\ .

# Section C: Anthropometry

# Staff ID:

# MUAC (cm) : 1. . 2. . 3. .

# Head Circumference(cm): 1. . 2. . 3. .

# Tricep Skinfold Thickness (cm) : 1. . 2. . 3. .

# Weight(kg): 1. .

# Length(cm): 1. . 2. . 3. .

# Section D: Phlebotomy

# Staff ID:

# 1. Was a venous sample taken? 0=No (Go to 1b.)

# 1=Yes (Go to 1a.)

# 1a. If so, how much? . ml.

# 0=No (Stop)

# 1b. If no, was capillary sample taken? 1=Yes

# 1c. If yes, how many capillary tubes were filled?

# 2. Hemoglobin (g/dL):

# .

# 2a. If Hemoglobin <7.0 g/dL, give tonic. Tonic given: 0=No, 1=Yes

# 3. Protoporphyrin (μmol/no/heme): .

# 4. Temperature: . ° F

# NNIPS-4 FORM 27

# 29 January, 2002

# NNIPS-4 DIMAG CLINIC B FORM (DCBF)

Week: Date: Visit:

# dd mm yy 1=39 weeks

# 2=52 weeks

# Section A: Identifiers/Addresses

# ISHWARPUR Ward: Sector: HH:

NNIPS Number First and Middle Name Family Name

# Father: ___________________ __________________

# Mother: ___________________ __________________

#

# Child: ___________________ _________________

#

# Date of birth: Age: mo. Sex:

# dd mm yy 1=Male;

# 2=Female

# Form Status: 1=Met

# 2=Not met

# 6=Refused Stop

# 7=Permanently Moved

# 8=Dead

# Relationship to child: 1=Mother,

# 2=Father,

# 3=Grandmother, Name: _________________________

# 4=Other, Relationship: __________________________

#

# Name: ________________________________

# Ethnic Group: 1=Pahadi

# 2=Madeshi

# 3=Muslim

# 4=Other

#

**Section B: Morbidity Form**

1. tkfO{sf] aRrfnfO{ tnsf nIf)fx?dWo] s"g} b]lvPsf] lyof] <

cfh ut 5 lbgdf

0=%}g; 1=% 0=lyPg; 1=lyof]

Hj/f] ========================================================== *

vf]sL =========================================================

:jf; km]g{ ufx|f]/l%^fl%^f] :jf; kmg]{ ================= *

kfgL h:tf] kftnf] lb;f -4 jf a(L k^s_=========== *

* olb aRrfnfO{ cfh of] nIf)f b]lvPdf km]/L e]^\g] sfo{qmd agfpg"xf];\ .

# Section C: Fagan Test

# 0=No

# Date: Date outside testing window? 1=Yes

# dd mm yy

# 1=First 1=AM

# Tester Staff ID: Order: 2=Second Time of Test: 2=PM

#

# 1. Was test completed ? 0=No (Go to 1a.)

# 1=Yes (Go to 2)

# 1a. If the test was not completed, what was the reason? 1=Baby closed eyes

# 2=Baby turned to breast

# 3=Baby looked away

# 4=Baby fussy

# 5=Other

# If other, please explain:________________________________________

# 2. Duration of Test: . minutes

# 3. Raw novelty Score: . 4. Scaled novelty Score: .

# 5. Comments about test: _____________________________________________________

# Section D: A-not-B Test

# 0=No

# Date: Date outside testing window? 1=Yes

# dd mm yy

# 1=First 1=AM

# Tester Staff ID: Order: 2=Second Time of Test: 2=PM

# 1. Was test completed? 0=No (Go to 1a.)

# 1=Yes (Go to 2.)

# 1a. If the test was not completed, what was the reason? 1=Baby closed eyes

# 2=Baby turned to breast

# 3=Baby looked away

# 4=Baby fussy

# 5=Baby did not engage

# with tester

# 6=Baby not able to pick up cloth or toy

# 7=Other

# If other, please explain: _____________________________________________________

# _______________________________________________________________________

# 2. Duration of Test: . minutes

# 3. tkfO{sf] aRrfn] tkfO{n] ;f]r]sf] h:t} v]nf}gfx? e]^\^fof]< 1=;f]r]sf]eGbf sd

# 2=;f]r]sf] hlQs}

# 3=;f]r]sf]eGbf a(L

# 4. Comments: ________________________________________________________________________

# NNIPS-4 FORM 28

15 January, 2002 **NNIPS-4 DIMAG A-not-B Test Form (DABTF)**

1=39 mo.

Week: Date: Visit: 2=52 mo.

dd mm yy

ISHWARPUR Ward: Sector: HH: Worker ID:

# NNIPS Number First and Middle Name Family Name

# Child: ________________________ _________________________

# 1=First

# Date of birth: Order: 2=Second Duration of test: .

# dd mm yy min.

Put check if correct, an X if incorrect, NR if no reach, SC if self-correct, or PR if partial reach

| Trial | Starts-L | Starts-R | Toy | Reached @ hiding | Delay # sec | Used 1 box | Saw 2 boxes | SC or PR | Pause/ Stop | Reason for Pause/Stop * |
| --- | --- | --- | --- | --- | --- | --- | --- | --- | --- | --- |
| 1 |  |  |  |  |  |  |  |  |  |  |
| 2 |  |  |  |  |  |  |  |  |  |  |
| 3 |  |  |  |  |  |  |  |  |  |  |
| 4 |  |  |  |  |  |  |  |  |  |  |
| 5 |  |  |  |  |  |  |  |  |  |  |
| 6 |  |  |  |  |  |  |  |  |  |  |
| 7 |  |  |  |  |  |  |  |  |  |  |
| 8 |  |  |  |  |  |  |  |  |  |  |
| 9 |  |  |  |  |  |  |  |  |  |  |
| 10 |  |  |  |  |  |  |  |  |  |  |
| 11 |  |  |  |  |  |  |  |  |  |  |
| **122** |  |  |  |  |  |  |  |  |  |  |
| 13 |  |  |  |  |  |  |  |  |  |  |
| 14 |  |  |  |  |  |  |  |  |  |  |
| **15** |  |  |  |  |  |  |  |  |  |  |
| 16 |  |  |  |  |  |  |  |  |  |  |
| 17 |  |  |  |  |  |  |  |  |  |  |
| 18 |  |  |  |  |  |  |  |  |  |  |
| 19 |  |  |  |  |  |  |  |  |  |  |
| 20 |  |  |  |  |  |  |  |  |  |  |
| 21 |  |  |  |  |  |  |  |  |  |  |
| 22 |  |  |  |  |  |  |  |  |  |  |
| 23 |  |  |  |  |  |  |  |  |  |  |
| 24 |  |  |  |  |  |  |  |  |  |  |
| 25 |  |  |  |  |  |  |  |  |  |  |
| 26 |  |  |  |  |  |  |  |  |  |  |
| 27 |  |  |  |  |  |  |  |  |  |  |

# 3. tkfO{sf] aRrfn] tkfO{n] ;f]r]sf] h:t} v]nf}gfx? e]^\^fof] < 1=;f]r]sf]eGbf sd

# 2=;f]r]sf] hlQs}

# 3=;F]r]sf]eGbf a(L

**F. Observation codes**

Protocol for Observation Codes

# NATURAL ACTIVIES

CARRIED

The child is carried by an adult or another child in the other person’s arms, on their back, or on their shoulders. Child may be strapped to the adult using fabric. Adult can be standing or sitting, moving or not moving. If adult is sitting and child is in front, the weight of the child is predominately resting on the adult’s arms. If the weight of the child is predominately on the lap, the code becomes SIT 1 or LIE.

LIE

In a supine position on the back, face down, or on side. This includes baby positioned in a lap, on top of another person, or while breastfeeding. Baby can be on back, moving arms and legs.

SIT 1

In a position in which the body is supported more or less upright by the buttocks resting on the ground, in a seat, in someone's lap, or on any other surface. By supported we mean that if the support were taken away, the child would tumble.

SIT 2

As in SIT 1, but child sits on own without support from a person or object.

ALL 4’s

The child’s body weight rests on hands and knees or hands and feet without the tummy touching the supporting surface and without moving forward. All 4’s movement will be coded as crawling. If only three of the four points mentioned above are touching the ground, this is still coded as ALL 4’s.

SQUAT

Rests on ground or other surface supported by feet with knees drawn up and heels close to or touching thighs. Only the feet may touch the supporting surface.

CREEP 1

Child’s tummy is in contact with the supporting surface. The chest and head are raised from the surface with or without the help of the arms. This will create an arch in the back. Limbs and head may move to one side or the other but body may not move forward or backward. This includes rotating, but does not include forward or backward movement.

CREEP 2

Moves on ground or other surface with the movement of the limbs while the tummy remains in contact with the supporting surface. The difference between CREEP 1 and CREEP 2 is that CREEP 2 requires forward or backward movement.

CRAWL

The child moves forwards or backwards on its four limbs. As in ALL 4’s with movement.

SCOOT

Forward, backward, or sideways movement, with or without the aid of the arms, with buttocks or at least one thigh remaining in contact with the ground.

KNEEL

In a stationary position with one or both knees resting on the supporting surface. The weight of the child is predominately on one or both knees.

STAND 1

Having an upright position on one foot or both feet while holding on to a person or a support. May move hands, feet, or head, or may rest head on a surface or someone’s lap.

STAND 2

As in STAND 1, but stands on own without support from a person or object.

WALK 1

Progresses in an upright position with support from a person or an object by advancing each foot alternately, never having both feet off the ground at once. May move forward, backward, or sideways, or stepping in the same spot. This may include pushing an object, such as a walking aid.

WALK 2

As in WALK 1, but the child walks on own without support from person or object. This code may include the activities of kneeling or squatting with movement.

CLIMB

The child moves up or on to an object or down or off of an object using his/her hands and/or feet.

RUN 1

The child moves on foot, such that both feet are off the ground at once, usually at a faster pace than walking, but slower than RUN 2.

RUN 2

As in RUN 1, but faster.

JUMP 1

The child is in an upright position and uses the assistance of an object or person to raise both feet off the ground. This includes a child being helped to jump by an adult (bouncing) or jumping while holding on to a person or object for support.

JUMP 2

The child uses the muscles of the legs and feet to lift both feet off the ground with no assistance from an object or person.

# SOCIAL BEHAVIORS

## OBJECT MANIPULATION

Child touches, mouths, waves, grabs, rubs, scratches, or handles any of the toys or other objects available to him or her. The child must be attending to the object. Usually the child’s own body parts are not considered object manipulation; however, if a baby is clearly playing with its own fingers or toes this is considered object manipulation. By the same criteria, baby playing with someone else’s finger, toes, hair, etc. might also be considered object manipulation. Swinging is coded as object manipulation.

EAT/DRINK

Child eats or drinks or is fed. If food is kept in the mouth for a period of time and the child is not chewing, this activity is not coded.

SOCIAL PLAY

Child plays with another person, with or without an object.

SOCIAL GESTURE

Child gestures; he/she points or applauds in the direction of another person (adult or peer) or in the direction of an effect from what another person did. This may include pointing, expressing Namaste, waving, beckoning, and holding hands.

AFFECTIVE DISPLAY

Child shows physical affection toward an adult, another child, or an animal. The affective display must involve touch.

NEGATIVE AFFECTIVE DISPLAY

Child shows physical aggression toward an adult, another child, or an animal. This includes hitting, slapping, kicking, pushing, pulling hair, and biting, This may also include spitting at, throwing an object at, or kicking something at another person. Hit with force while laughing is included in this category.

SOCIAL SMILE

Child smiles; his/her mouth is turned upward. This behavior is overtly directed to another person.

BREASTFEEDING

Child is breastfeeding. Nipple must be in the child’s mouth. If the nipple is in the child’s mouth, but the child is not sucking or is breastfeeding under a cover and the activity cannot be viewed completely, breastfeeding is still coded.

FUSSING

Child fusses; he/she cries or shows other signs of displeasure.

### VOCALIZATIONS

INITIATES WITH CARETAKER

Child vocalizes; he/she voices a sound; social; child initiates vocalization with caretaker.

RESPONDS TO CARETAKER

Child vocalizes; he/she voices a sound; social; child responds to vocalization with caregiver.

INITIATES WITH PEER

Child vocalizes; he/she voices s sound; social; child initiates vocalization with peer or sibling.

RESPONDS TO PEER

Child vocalizes; he/she voices a sound; social; child responds to vocalization with peer or sibling.

NON-SOCIAL WITH OBJECT

Child vocalizes; he/she voices a sound; social; child vocalizes, but not toward another person, while an object is present.

NON-SOCIAL NO OBJECT

Child vocalizes; he/she voices a sound; social; child vocalizes, but not toward another person, no object is present.

**G. DIMAG timetable of observer and clinic visits**

| DIMAG TIMETABLE | | |  |  |  |  |  |  |  |  |  |  |  |  |  |  |  |
| --- | --- | --- | --- | --- | --- | --- | --- | --- | --- | --- | --- | --- | --- | --- | --- | --- | --- |
|  |  | **Jan '02** | **Feb '02** | **Mar '02** | **Apr '02** | **May '02** | **Jun '02** | **Jul '02** | **Aug '02** | **Sep '02** | **Oct '02** | **Nov '02** | **Dec '02** | **Jan '03** | **Feb '03** | **Mar '03** | **Apr '03** |
| **Group 1** |  |  |  |  |  |  |  |  |  |  |  |  |  |  |  |  |  |
|  | *Observation* | 1 | 1 |  | 2 | 2 |  | 3 | 3 |  | 4 | 4 |  | 5 | 5 |  |  |
|  | *Interview* | 1 | 1 |  | 2 | 2 |  | 3 | 3 |  | 4 | 4 |  | 5 | 5 |  |  |
|  | *Clinic A* | 1 | 1 |  |  |  |  |  |  |  |  |  |  | 2 | 2 |  |  |
|  | *Clinic B* | 1,2 | 1,2 | 1,2 | 1,2 | 1,2 | 1,2 | 1,2 | 1,2 | 1,2 | 2 | 2 | 2 |  |  |  |  |
|  |  |  |  |  |  |  |  |  |  |  |  |  |  |  |  |  |  |
| **Group 2** |  |  |  |  |  |  |  |  |  |  |  |  |  |  |  |  |  |
|  | *Observation* |  |  | 1 | 1 |  | 2 | 2 |  | 3 | 3 |  | 4 | 4 |  | 5 | 5 |
|  | *Interview* |  |  | 1 | 1 |  | 2 | 2 |  | 3 | 3 |  | 4 | 4 |  | 5 | 5 |
|  | *Clinic A* |  |  | 1 | 1 |  |  |  |  |  |  |  |  |  |  | 2 | 2 |
|  |  |  |  |  |  |  |  |  |  |  |  |  |  |  |  |  |  |
| **The numbers above refer to the visit number. There will be five rounds of observations & interviews and two clinic visits for both Clinic A & Clinic B. Note that not every child will be seen for both Clinic B1 and B2, as the scheduling of Clinic B depends on the child's age.* | | | | | | | | | | | | | | | | |  |
|  |  |  |  |  |  |  |  |  |  |  |  |  |  |  |  |  |  |
| **With the +/- 1-month rule in place for the 3-month home visits, we should finish our work at the beginning of the second month recorded (April, July, October, January, and April).* | | | | | | | | | | | | | | | | | |

**H. First and last dates for conducting observations in each ward**

| Ward | MIN3 | MAX3 | MIN2 | MAX2 | MIN4 | MAX4 | MIN6 | MAX6 | MIN5 | MAX5 | MIN7 | MAX7 |
| --- | --- | --- | --- | --- | --- | --- | --- | --- | --- | --- | --- | --- |
| 1 | 4/28/2002 | 7/1/2002 | 3/28/2002 | 6/1/2002 | 5/28/2002 | 8/1/2002 | 7/28/2002 | 10/1/2002 | 6/28/2002 | 9/1/2002 | 8/28/2002 | 11/1/2002 |
| 2 | 4/28/2002 | 7/22/2002 | 3/28/2002 | 6/22/2002 | 5/28/2002 | 8/22/2002 | 7/28/2002 | 10/22/2002 | 6/28/2002 | 9/22/2002 | 8/28/2002 | 11/22/2002 |
| 3 | 6/4/2002 | 7/22/2002 | 5/4/2002 | 6/22/2002 | 7/4/2002 | 8/22/2002 | 9/4/2002 | 10/22/2002 | 8/4/2002 | 9/22/2002 | 10/4/2002 | 11/22/2002 |
| 4 | 6/18/2002 | 7/1/2002 | 5/18/2002 | 6/1/2002 | 7/18/2002 | 8/1/2002 | 9/18/2002 | 10/1/2002 | 8/18/2002 | 9/1/2002 | 10/18/2002 | 11/1/2002 |
| 5 | 6/18/2002 | 7/22/2002 | 5/18/2002 | 6/22/2002 | 7/18/2002 | 8/22/2002 | 9/18/2002 | 10/22/2002 | 8/18/2002 | 9/22/2002 | 10/18/2002 | 11/22/2002 |
| 6 | 6/18/2002 | 7/22/2002 | 5/18/2002 | 6/22/2002 | 7/18/2002 | 8/22/2002 | 9/18/2002 | 10/22/2002 | 8/18/2002 | 9/22/2002 | 10/18/2002 | 11/22/2002 |
| 7 | 6/25/2002 | 7/1/2002 | 5/25/2002 | 6/1/2002 | 7/25/2002 | 8/1/2002 | 9/25/2002 | 10/1/2002 | 8/25/2002 | 9/1/2002 | 10/25/2002 | 11/1/2002 |
| 8 | 6/25/2002 | 7/22/2002 | 5/25/2002 | 6/22/2002 | 7/25/2002 | 8/22/2002 | 9/25/2002 | 10/22/2002 | 8/25/2002 | 9/22/2002 | 10/25/2002 | 11/22/2002 |
| 9 | 7/1/2002 | 7/15/2002 | 6/1/2002 | 6/15/2002 | 8/1/2002 | 8/15/2002 | 10/1/2002 | 10/15/2002 | 9/1/2002 | 9/15/2002 | 11/1/2002 | 11/15/2002 |
|  |  |  |  |  |  |  |  |  |  |  |  |  |
|  |  |  |  |  |  |  |  |  |  |  |  |  |
| Ward | MIN9 | MAX9 | MIN8 | MAX8 | MIN10 | MAX10 | MIN12 | MAX12 | MIN11 | MAX11 | MIN13 | MAX13 |
| 1 | 1/1/2003 | 12/18/2002 | 9/28/2002 | 12/1/2002 | 1/11/2003 | 12/18/2002 | 1/28/2003 | 4/1/2003 | 1/4/2003 | 12/28/2002 | 2/28/2003 | 5/1/2003 |
| 2 | 1/1/2003 | 12/25/2002 | 9/28/2002 | 12/22/2002 | 1/11/2003 | 12/4/2002 | 1/28/2003 | 4/22/2003 | 1/4/2003 | 12/28/2002 | 2/28/2003 | 5/22/2003 |
| 3 | 1/15/2003 | 12/25/2002 | 11/4/2002 | 12/22/2002 | 1/4/2003 | 2/22/2003 | 3/4/2003 | 4/22/2003 | 2/4/2003 | 3/22/2003 | 4/4/2003 | 5/22/2003 |
| 4 | 1/1/2003 | 12/25/2002 | 11/18/2002 | 12/1/2002 | 1/18/2003 | 2/1/2003 | 3/18/2003 | 4/1/2003 | 2/18/2003 | 3/1/2003 | 4/18/2003 | 5/1/2003 |
| 5 | 1/22/2003 | 12/25/2002 | 11/18/2002 | 12/22/2002 | 1/18/2003 | 2/22/2003 | 3/18/2003 | 4/22/2003 | 2/18/2003 | 3/22/2003 | 4/18/2003 | 5/22/2003 |
| 6 | 1/22/2003 | 12/25/2002 | 11/18/2002 | 12/22/2002 | 1/18/2003 | 2/22/2003 | 3/18/2003 | 4/22/2003 | 2/18/2003 | 3/22/2003 | 4/18/2003 | 5/22/2003 |
| 7 | 1/1/2003 | 12/25/2002 | 11/25/2002 | 12/1/2002 | 1/25/2003 | 2/1/2003 | 3/25/2003 | 4/1/2003 | 2/25/2003 | 3/1/2003 | 4/25/2003 | 5/1/2003 |
| 8 | 1/1/2003 | 12/25/2002 | 11/25/2002 | 12/22/2002 | 1/25/2003 | 2/22/2003 | 3/25/2003 | 4/22/2003 | 2/25/2003 | 3/22/2003 | 4/25/2003 | 5/22/2003 |
| 9 | 1/1/2003 | 1/15/2003 | 12/1/2002 | 12/15/2002 | 2/1/2003 | 2/15/2003 | 4/1/2003 | 4/15/2003 | 3/1/2003 | 3/15/2003 | 5/1/2003 | 5/15/2003 |

#### I. Standardization reports

#### Observer Standardization Exercises

| **Table 1. Baseline Standardization of Observers using a Gold Standard and Kappa** | | | | | | |  |
| --- | --- | --- | --- | --- | --- | --- | --- |
| Oct-01 | **202** | **203** | **204** | **205** | **206** | **207** | **208** |
|  | **Dipak** | **Roshan** | **Ishwori** | **Yadav** | **Rudra** | **Ram Aseshor** | **Menuka** |
|  | **1 hour** | **1 hour** | **1 hour** | **1 hour** | **1 hour** | **1 hour** | **1 hour** |
| **CODES** | **Oct. 18, 01** | **Oct. 18 01** | **Oct. 18 01** | **Oct. 18 01** | **Oct. 18 01** | **Oct. 16 01** | **Oct. 18 01** |
| **Motor** | kappa (n) | kappa (n) | kappa (n) | kappa (n) | kappa (n) | kappa (n) | kappa (n) |
| Carried | 1.0 (6) | 1.0 (6) | .981 (7) | .981 (7) | .967(8) | .967 (8) | 0.981(7) |
| Lie | .939 (9) | .939 (9) | .939 (9) | .986 (10) | .986 (10) | .986 (10) | 0.939 (9) |
| Sit 1 | .793 (31) | .894 (30) | .923 (29) | .849 (29) | .84 (36) | .904 (37) | .876 (35) |
| Sit 2 | .960 (74) | .821 (77) | .918 (72) | .945 (73) | .924 (72) | .872 (57) | .892 (73) |
| All 4s | .592 (7) | .393 (4) | 1.0 (3) | .661 (6) | .661 ((6) | .706 (9) | .770 (6) |
| Squat | .985 (18) | .959 (20) | .977 (18) | .977 (18) | .997 (18) | .965 (16) | .970 (18) |
| Creep 1 | 1.0 (0) | 0 (0) | 1.0 (0) | 1.0 (0) | 1 (0) | 1.0 (0) | 1.0 (0) |
| Creep 2 | 1.0 (0) | 0 (0) | 1.0 (0) | 1.0 (0) | 1 (0) | 1.0 (0) | 1.0 (0) |
| Crawl | 1.0 (0) | 0 (0) | 1.0 (0) | 1.0 (0) | 1 (0) | 1.0 (0) | 1.0 (0) |
| Scoot | 1.0 (0) | 0 (0) | 1.0 (0) | 1.0 (0) | 1 (0) | 1.0 (0) | 1.0 (0) |
| Kneel | 1.0 (0) | 0 (0) | 1.0 (0) | 1.0 (0) | 1 (0) | 1.0 (0) | 1.0 (0) |
| Stand 1 | .89 (52) | .871 (49) | .859 (52) | .957 (49) | .813 (51) | .859 (49) | .875 (54) |
| Stand 2 | .95 (120) | .958 (122) | .979 (118) | .958 (119) | .94 (124) | .946 (119) | .936 (121) |
| Walk 1 | .937 (2) | 1.0 (1) | 1.0 (1) | .937 (2) | .747 (3) | 1.0 (0) | 0 (1) |
| Walk 2 | .836 (64) | .883 (53) | .891 (58) | .889 (53) | .858 (58) | .916 (56) | .830 (60) |
| Climb | .894 (13) | .913 (11) | .879 (14) | .853 (15) | .888 (14) | .989 (12) | .851 (13) |
| Run 1 | 1.0 (0) | 1.0 (0) | 1.0 (0) | 1.0 (0) | 1 (0) | 1.0 (0) | 1.0 (0) |
| Run 2 | .937 (2) | 1.0 (1) | .937 (2) | 1.0 (1) | 1 (0) | 1.0 (1) | 1.0 (1) |
| Jump 1 | 1.0 (0) | 0 (0) | 1.0 (0) | 1.0 (0) | 1 (0) | 1.0 (0) | 1.0(0) |
| Jump 2 | 1.0 (0) | 0 (0) | 1.0 (0) | 1.0 (0) | 1 (0) | 1.0 (0) | 1.0 (0) |
| **Social Behaviors** |  |  |  |  |  |  |  |
| Object Manipulation | .919 (133) | .848 (126) | .903 (147) | .930 (139) | .893 (138) | .893 (131) | .923 (136) |
| Eat/Drink | .974 (48) | .917 (48) | .971 (47) | .974(49) | .972 (49) | .926 (48) | .985 (49) |
| Social Play | .725 (9) | .842 (3) | .725 (9) | .771 (7) | .622 (8) | .963 (5) | .725 (9) |
| Social Gesture | .776 (24) | .759 (25) | .864 (25) | .754 (29) | .706 (35) | .824 (25) | .787 (29) |
| Affective Display | .870 (9) | .842 (11) | .895 (11) | .762 (9) | .881 (10) | .785 (11) | .879 (10) |
| Negative Aff. Display | 0 (1) | 1.0 (0) | 1.0 (0) | 1.0 (0) | 1.0 (0) | 0 (1) | 1.0 (0) |
| Social Smile | .741 (20) | .791 (11) | .823 (16) | .771 (15) | .764 (19) | .781 (10) | .746 (16) |
| Breastfeeding | 1.0 (0) | 1.0 (0) | 1.0 (0) | 1.0 (0) | 1.0 (0) | 1.0 (0) | 1.0 (0) |
| Carried in Arms | 1.0 (0) | 1.0 (0) | .981 (6) | .981 (6) | .967 (6) | .983 (7) | .981 (6) |
| Fussing | .968 (3) | .795 (6) | .795 (6) | .886 (5) | .714 (4) | .770 (5) | .795 (6) |
| Int. w/ Caregiver | .772 (55) | .770 (53) | .834 (56) | .866 (51) | .786 (57) | .764 (46) | .798 (55) |
| Resp. to Caregiver | .756 (34) | .710 (32) | .855 (37) | .882 (33) | .747 (32) | .755 (31) | .838 (38) |
| Int. with Peer | .962 (22) | .887 (19) | .947 (21) | .930 (22) | .940 (21) | .867 ( 22) | .923 (18) |
| Resp. to Peer | 1.0 (0) | 1.0 (0) | 1.0 (0) | 1.0 (0) | 1.0 (0) | 1.0 (0) | 1.0 (0) |
| Non-social w/ Object | .596 (10) | .639 (9) | .604 (12) | .534 (8) | .534 (8) | .655 (15) | .487 (9) |
| Non-social w/ Object | .925 (9) | .913 (8) | .956 (6) | .917 (8) | 1.0 (0) | .905 (8) | .501 (10) |
| **Rules: Kappa Scores > 70%; if the number of observations is <15, i okay to have a score <70%;** | | | | | | | |
| **A gold standard was used to generate these scores** | | | | |  |  |  |

|  | **209** | **210** | **211** | **212** | **213** | **214** | **215** | **216** |
| --- | --- | --- | --- | --- | --- | --- | --- | --- |
|  | **Kharga** | **Shyam** | **Sulochana** | **Kesab** | **Kiran** | **Padam** | **Nir BDR** | **Ram NR** |
|  | **1 hour** | **1 hour** | **1 hour** | **1 hour** | **1 hour** | **1 hour** | **1 hour** | **1 hour** |
| **CODES** | **Oct. 18 01** | **Oct. 18 01** | **Oct. 18 01** | **Oct. 18 01** | **Oct. 18 01** | **Oct. 18 01** | **Oct. 18 01** | **Oct. 18 01** |
| **Motor** | kappa (n) | kappa (n) | kappa (n) | kappa (n) | kappa (n) | kappa (n) | kappa (n) | kappa (n) |
| Carried | .981 (7) | .981 (7) | .981 (7) | 1.0 (6) | .981 (7) | .967 (8) | .981 (7) | .981 (7) |
| Lie | .986 (10) | .939 (9) | .939 (9) | .939 (9) | .973 (10) | .354 (9) | .986 (10) | .939 (9) |
| Sit 1 | .915 (28) | .787 (28) | .831 (31) | .836 (33) | .953 (27) | .915 (28) | .924 (29) | .905 (30) |
| Sit 2 | .892 (70) | .930 (75) | .849 (76) | .975 (71) | .961 (70) | .900 (75) | .953 (73) | .845 (74) |
| All 4s | .968 (4) | .592 (7) | .746 (5) | .542 (10) | .968 (4) | .324 (5) | .537 (8) | ,858 (5) |
| Squat | .952 (20) | .930 (20) | .957 (19) | .976 (17) | .925 (21) | .937 (20) | .959 (20) | ,925 (15) |
| Creep 1 | 1.0 (0) | 1.0 (0) | 1.0 (0) | 1.0 (0) | 1.0 (0) | 1.0 (0) | 1.0 (0) | 1,0 (0) |
| Creep 2 | 1.0 (0) | 1.0 (0) | 1.0 (0) | 1.0 (0) | 1.0 (0) | 1.0 (0) | 1.0 (0) | 1.0 (0) |
| Crawl | 1.0 (0) | 1.0 (0) | 1.0 (0) | 1.0 (0) | 1.0 (0) | 1.0 (0) | 1.0 (0) | 1.0 (0) |
| Scoot | 1.0 (0) | 1.0 (0) | 1.0 (0) | 1.0 (0) | 1.0 (0) | 1.0 (0) | 1.0 (0) | 1.0 (0) |
| Kneel | 1.0 (0) | 1.0 (0) | 1.0 (0) | 1.0 (0) | 1.0 (0) | 1.0 (0) | 1.0 (0) | 1.0 (0) |
| Stand 1 | .853 (52) | .851 (51) | .962 (52) | .919 (50) | .867 (49) | .924 (49) | .824 (52) | .931 (52) |
| Stand 2 | .933 (121) | .918 (125) | .983 (116) | .942 (123) | .940 (122) | .869 (127) | .914 (124) | .955 (122) |
| Walk 1 | .937 (2) | .937 (2) | 0 (1) | 1.0 (1) | .747 (3) | .937 (2) | .937 (2) | .937 (2) |
| Walk 2 | .850 (61) | .880 (59) | .804 (60) | .848 (58) | .855 (61) | .851 (54) | .845 (64) | .854 (56) |
| Climb | .813 (14) | .878 (14) | .810 (16) | .920 (12) | .851 (13) | .890 (13) | .704 (15) | .867 (12) |
| Run 1 | 1.0 (0) | 1.0 (0) | 1.0 (0) | 1.0 (0) | 1.0 (0) | 1.0 (0) | 1.0 (0) | 1.0 (0) |
| Run 2 | 1.0 (1) | 1.0 (1) | .937 (2) | 1.0 (1) | .937 (2) | .937 (2) | 1.0 (0) | 1.0 (1) |
| Jump 1 | 1.0 (0) | 1.0 (0) | 1.0 (0) | 1.0 (0) | 1.0 (0) | 1.0 (0) | 1.0 (0) | 1.0 (0) |
| Jump 2 | 1.0 (0) | 1.0 (0) | 1.0 (0) | 1.0 (0) | 1.0 (0) | 1.0 (0) | 1.0 (0) | 1.0 (0) |
| **Social Behaviors** |  |  |  |  |  |  |  |  |
| Object Manipulation | .918 (129) | .904 (128) | .900 (143) | .901 (143) | .929 (136) | .858 (123) | .900 (135) | .875 (144) |
| Eat/Drink | .957 (43) | .956 (46) | .955 (44) | .970 (45) | .956 (45) | .951 (45) | .965 (45) | .956 (50) |
| Social Play | .765 (4) | .687 (8) | .669 (7) | .725 (9) | .743 (7) | .770 (5) | .742 (6) | .725 (5) |
| Social Gesture | .823 (24) | .824 (29) | .795 (32) | .843 (24) | .763 (31) | .713 (23) | .712 (28) | .877 ( 27) |
| Affective Display | .747 (11) | .822 (10) | .825 (10) | .802 (12) | .888 (11) | .792 (10) | .869 (9) | .840 (11) |
| Negative Aff. Display | 1.0 (0) | 1.0 (0) | 1.0 (0) | 1.0 (0) | 0 (1) | 1.0 (0) | 1.0 (0) | 1.0 (0) |
| Social Smile | .545 (13) | .783 (18) | .625 (14) | .762 (23) | .786 (15) | .584 (13) | .616 (13) | .769 (20) |
| Breastfeeding | 1.0 (0) | 1.0 (0) | 1.0 (0) | 1.0 (0) | 1.0 (0) | 1.0 (0) | 1.0 (0) | 1.0 (0) |
| Carried in Arms | .981 (6) | .981 (6) | .981 (6) | 1.0 (6) | .981 (6) | .967 (6) | .981 (6) | .981 (6) |
| Fussing | .745 (5) | .660 (6) | .886 (5) | .886 (5) | ..795 (6) | .714 (4) | .745 (5) | .886 (5) |
| Int. w/ Caregiver | .810 (53) | .779 (57) | .812 (51) | .797 (50) | .830 (53) | .772 (55) | .784 (50) | .834 (52) |
| Resp. to Caregiver | .849 (27) | .792 (33) | .820 (37) | .858 (34) | .854 (32) | .800 (28) | .817 (36) | .779 (32) |
| Int. with Peer | .900 (18) | .940 (21) | .907 (19) | .950 (23) | .896 (16) | .878 (17) | .872 (16) | .790 (19) |
| Resp. to Peer | 1.0 (0) | 1.0 (0) | 1.0 (0) | 1.0 (0) | 1.0 (0) | 1.0 (0) | 1.0 (0) | 0 (1) |
| Non-social w/ Object | .559 (10) | .559 (11) | .559 (10) | .525 (12) | .658 (8) | .658 (8) | .639 (9) | .661 (11) |
| Non-social w/ Object | .971 (7) | .622 (8) | .596 (10) | .890 (6) | .688 (8) | .869 (9) | .841 (7) | .985 (8) |

| **Post-Baseline Observer Standardization: After Round 1** | | | | | | | | | | | | | | | |
| --- | --- | --- | --- | --- | --- | --- | --- | --- | --- | --- | --- | --- | --- | --- | --- |
| **Apr-02** | | | | | | | | | | | | | | | |
|  | **Dipak** | **Roshan** | **Ishwori** | **Yadav** | **Rudra** | **Ram As.** | **Menuka** | **Khadga** | **Shyam** | **Sulochana** | **Kesab** | **Kiran** | **Padam** | **Nir BDR** | **Ram NR** |
| **Dipak** |  | **x** | **x** |  | **x** | **x** | **x** | **x** | **x** | **x** | **x** | **x** | **x** | **x** | **x** |
| **Roshan** | **x** |  | **x** | **x** | **x** | **x** | **x** | **x** | **x** | **x** | **x** | **x** | **x** | **x** | **x** |
| **Ishwori** | **x** | **x** |  | **x** | **x** | **x** | **x** | **x** | **x** | **x** | **x** | **x** | **x** | **x** | **x** |
| **Yadav** |  | **x** | **x** |  | **x** | **x** | **x** | **x** |  | **x** |  | **x** | **x** | **x** | **x** |
| **Rudra** | **x** | **x** | **x** | **x** |  | **x** | **x** | **x** |  | **x** |  | **x** | **x** |  |  |
| **Ram Asesh** | **x** | **x** | **x** | **x** | **x** |  | **x** | **x** | **x** | **x** | **x** | **x** | **x** | **x** | **x** |
| **Menuka** | **x** | **x** | **x** | **x** | **x** | **x** |  | **x** | **x** | **x** | **x** | **x** | **x** | **x** | **x** |
| **Khadga** | **x** | **x** | **x** | **x** | **x** | **x** |  |  | **x** | **x** | **x** | **x** | **x** | **x** | **x** |
| **Shyam** | **x** | **x** | **x** |  |  | **x** | **x** | **x** |  | **x** | **x** | **x** | **x** | **x** |  |
| **Sulochana** | **x** | **x** | **x** | **x** | **x** | **x** | **x** | **x** | **x** |  | **x** | **x** | **x** | **x** | **x** |
| **Kesab** | **x** | **x** | **x** |  |  | **x** | **x** | **x** | **x** | **x** |  | **x** | **x** | **x** |  |
| **Kiran** | **x** | **x** | **x** | **x** | **x** | **x** | **x** | **x** | **x** | **x** | **x** |  | **x** | **x** | **x** |
| **Padam** | **x** | **x** | **x** | **x** | **x** | **x** | **x** | **x** | **x** | **x** | **x** | **x** |  | **x** | **x** |
| **Nir BDR** | **x** | **x** | **x** | **x** | **x** | **x** | **x** | **x** | **x** | **x** | **x** | **x** | **x** |  | **x** |
| **Ram NR** | **x** | **x** | **x** | **x** | **x** | **x** | **x** | **x** |  | **x** |  | **x** | **x** | **x** |  |
|  |  |  |  |  |  |  |  |  |  |  |  |  |  |  |  |
| **For these standardization exercises the observers conducted 30-minute observations while in small groups in the field.** | | | | | | | | | | | | | | |  |
|  |  |  |  |  |  |  |  |  |  |  |  |  |  |  |  |
| **Observation** | **Date** | **Child IDs** | | | |  |  |  |  |  |  |  |  |  |  |
| 1 | 4/8/2002 | 298814 | 298877 | 298797 | 298933 |  |  |  |  |  |  |  |  |  |  |
| 2 | 4/9/2002 | 298814 | 298877 | 298797 | 298933 |  |  |  |  |  |  |  |  |  |  |
| 3 | 4/10/2002 | 298814 | 298877 | 298797 | 298933 |  |  |  |  |  |  |  |  |  |  |
| 4 | 4/11/2002 | 298814 | 298877 | 298797 | 298933 |  |  |  |  |  |  |  |  |  |  |
| 5 | 4/12/2002 | 298814 | 298877 | 298797 | 298933 |  |  |  |  |  |  |  |  |  |  |
| 6 | 4/29/2002 | 298814 | 298877 | 298797 | 298933 |  |  |  |  |  |  |  |  |  |  |
| 7 | 4/30/2002 | 298814 | 298877 | 298797 | 298933 |  |  |  |  |  |  |  |  |  |  |
| 8 | 5/1/2002 | 187469 | 298933 | 298814 | 298877 |  |  |  |  |  |  |  |  |  |  |
| 9 | 5/2/2002 | 298877 |  |  |  |  |  |  |  |  |  |  |  |  |  |

| **Observer Standardization** | | | | | | | | | | | | | | | |
| --- | --- | --- | --- | --- | --- | --- | --- | --- | --- | --- | --- | --- | --- | --- | --- |
| **Jul-02** | | | | | | | | | | | | | | | |
|  | **Dipak** | **Roshan** | **Ishwori** | **Yadav** | **Rudra** | **Ram As.** | **Menuka** | **Khadga** | **Shyam** | **Sulochana** | **Kesab** | **Kiran** | **Padam** | **Nir BDR** | **Ram NR** |
| **Dipak** |  | **x** | **x4** | **x** | **x** | **x** | **x** | **x** | **x** | **x9** | **x** | **x** | **x** | **x** | **x7** |
| **Roshan** | **x** |  | **x** | **x4** | **x** | **x** | **x** | **x** | **x** | **x7** | **x** | **x7** | **x5** | **x** | **x** |
| **Ishwori** | **x4** | **x** |  | **x** | **x** | **x** | **x** | **x4** | **x** | **x2** | **x** | **x9** | **x** | **x** | **x3** |
| **Yadav** | **x** | **x4** | **x** |  | **x** | **x** | **x** | **x** | **x** | **x** | **x** | **x** | **x** | **x** | **x4** |
| **Rudra** | **x** | **x** | **x** | **x** |  | **x** | **x5** | **x** | **x** | **x6** | **x** | **x** | **x** | **x5** | **x6** |
| **Ram Asesh** | **x** | **x** | **x** | **x** | **x** |  |  | **x4** | **x** | **x4** | **x** | **x7** | **x** | **x** | **x3** |
| **Menuka** | **x** | **x** | **x** | **x** | **x5** |  |  | **x** | **x** | **x** | **x** |  | **x** | **x2** | **x6** |
| **Khadga** | **x** | **x** | **x4** | **x** | **x** | **x4** | **x** |  | **x** | **x** | **x** | **x** | **x** | **x** | **x** |
| **Shyam** | **x** | **x** | **x** | **x** | **x** | **x** | **x** | **x** |  | **x2** | **x** | **x** | **x** | **x** | **x** |
| **Sulochana** | **x9** | **x7** | **x2** | **x** | **x6** | **x4** | **x** | **x** | **x** |  | **x** | **x** | **x3** | **x2** | **x3** |
| **Kesab** | **x** | **x** | **x** | **x** | **x** | **x** | **x** | **x** | **x** | **x** |  | **x2** | **x5** | **x** | **x6** |
| **Kiran** | **x** | **x7** | **x9** | **x** | **x** | **x7** |  | **x** | **x** | **x** | **x2** |  | **x8** | **x** | **x** |
| **Padam** | **x** | **x5** | **x** | **x** | **x** | **x** | **x** | **x** | **x** | **x3** | **x5** | **x8** |  | **x** | **x** |
| **Nir BDR** | **x** | **x** | **x** | **x** | **x5** | **x** | **x2** | **x** | **x** | **x2** | **x** | **x** | **x** |  | **x3** |
| **Ram NR** | **x7** | **x** | **x3** | **x4** | **x6** | **x3** | **x6** | **x** | **x** | **x3** | **x6** | **x** | **x** | **x3** |  |
|  |  |  |  |  |  |  |  |  |  |  |  |  |  |  |  |
| **For these standardization exercises the observers watched 30-minute videos** | | | | | | | | |  |  |  |  |  |  |  |
|  |  |  |  |  |  |  |  |  |  |  |  |  |  |  |  |
| **Observation** | **Date** | **Child ID** |  |  |  |  |  |  |  |  |  |  |  |  |  |
| 1 | 7/15/2002 | 165146 |  |  |  |  |  |  |  |  |  |  |  |  |  |
| 2 | 7/16/2002 | 183627 |  |  |  |  |  |  |  |  |  |  |  |  |  |
| 3 | 7/17/2002 | 184006 |  |  |  |  |  |  |  |  |  |  |  |  |  |
| 4 | 7/17/2002 | 165095 |  |  |  |  |  |  |  |  |  |  |  |  |  |
| 5 | 7/22/2002 | 165095 |  |  |  |  |  |  |  |  |  |  |  |  |  |
| 6 | 7/22/2002 | 165256 |  |  |  |  |  |  |  |  |  |  |  |  |  |
| 7 | 7/22/2002 | 183858 |  |  |  |  |  |  |  |  |  |  |  |  |  |
| 8 | 7/22/2002 | 183984 |  |  |  |  |  |  |  |  |  |  |  |  |  |
| 9 | 7/30/2002 | 165095 |  |  |  |  |  |  |  |  |  |  |  |  |  |
| 10 | 7/30/2002 | 165227 |  |  |  |  |  |  |  |  |  |  |  |  |  |

| **Observer Standardization** | | | | | | | | | | | | | | | |
| --- | --- | --- | --- | --- | --- | --- | --- | --- | --- | --- | --- | --- | --- | --- | --- |
| **Oct-02** | | | | | | | | | | | | | | | |
|  | **Dipak** | **Roshan** | **Ishwori** | **Y.** | **Rudra** | **R.A.** | **Menuka** | **Khadga** | **Shyam** | **Sulochana** | **Kesab** | **Kiran** | **Padam** | **Nir BDR** | **Ram NR** |
| **Dipak** |  | **x1** | **x1** |  | **x1** |  | **x** | **x1** | **x1** | **x1** | **x1** | **x** | **x1** | **x1** | **x1** |
| **Roshan** | **x1** |  | **x1** |  | **x1** |  | **x1** | **x1** | **x1** | **x** | **x1** | **x1** | **x1** | **x1** | **x1** |
| **Ishwori** | **x1** | **x1** |  |  | **x** |  | **x1** | **x1** | **x1** | **x1** | **x1** | **x1** | **x1** | **x1** | **x1** |
| **Yadav** |  |  |  |  |  |  |  |  |  |  |  |  |  |  |  |
| **Rudra** | **x1** | **x1** | **x** |  |  |  | **x1** | **x1** | **x1** | **x** | **x1** | **x** | **x1** | **x1** | **x** |
| **Ram Asesh** |  |  |  |  |  |  |  |  |  |  |  |  |  |  |  |
| **Menuka** | **x** | **x1** | **x1** |  | **x1** |  |  | **x** | **x1** | **x** | **x1** | **x1** | **x1** | **x1** | **x1** |
| **Khadga** | **x1** | **x1** | **x1** |  | **x1** |  | **x** |  | **x1** | **x1** | **x1** | **x1** | **x1** | **x1** | **x** |
| **Shyam** | **x1** | **x1** | **x1** |  | **x1** |  | **x1** | **x1** |  | **x** | **x1** | **x1** | **x** | **x1** | **x1** |
| **Sulochana** | **x1** | **x** | **x1** |  | **x1** |  | **x** | **x1** | **x** |  | **x** | **x1** | **x** | **x1** | **x1** |
| **Kesab** | **x1** | **x1** | **x1** |  | **x1** |  | **x1** | **x1** | **x1** | **x** |  | **x1** | **x1** | **x1** | **x1** |
| **Kiran** | **x** | **x1** | **x1** |  | **x** |  | **x1** | **x1** | **x1** | **x1** | **x1** |  | **x1** | **x** | **x1** |
| **Padam** | **x1** | **x1** | **x1** |  | **x1** |  | **x1** | **x1** | **x** | **x** | **x1** | **x1** |  | **x** | **x1** |
| **Nir BDR** | **x1** | **x1** | **x1** |  | **x1** |  | **x1** | **x1** | **x1** | **x1** | **x1** | **x** | **x** |  | **x1** |
| **Ram NR** | **x1** | **x1** | **x1** |  | **x** |  | **x1** | **x** | **x1** | **x1** | **x1** | **x1** | **x1** | **x1** |  |
|  |  |  |  |  |  |  |  |  |  |  |  |  |  |  |  |
| **For these standardization exercises the observers watched 30-minute video segments. "1" indicates observers who had trouble with the initiates with caretaker code. It was difficult for the observers to discriminate between vocalizations uttered by the child and caretaker versus those uttered by others in the video.** | | | | | | | | | | | | | | | |
|
|
|  |  |  |  |  |  |  |  |  |  |  |  |  |  |  |  |
| **Observation** | **Date** | **Child ID** |  |  |  |  |  |  |  |  |  |  |  |  |  |
| 1 | 10/24/2002 | 165146 |  |  |  |  |  |  |  |  |  |  |  |  |  |
| 2 | 10/24/2002 | 165256 |  |  |  |  |  |  |  |  |  |  |  |  |  |
| 3 | 10/24/2002 | 183627 |  |  |  |  |  |  |  |  |  |  |  |  |  |
| 4 | 10/25/2002 | 165146 |  |  |  |  |  |  |  |  |  |  |  |  |  |
| 5 | 10/25/2002 | 165227 |  |  |  |  |  |  |  |  |  |  |  |  |  |
| 6 | 10/25/2002 | 183627 |  |  |  |  |  |  |  |  |  |  |  |  |  |
| 7 | 10/29/2002 | 165095 |  |  |  |  |  |  |  |  |  |  |  |  |  |
| 8 | 10/29/2002 | 165227 |  |  |  |  |  |  |  |  |  |  |  |  |  |
| 9 | 10/29/2002 | 165256 |  |  |  |  |  |  |  |  |  |  |  |  |  |

| **Observer Standardization** | | | | | | | | | | | | | | | |
| --- | --- | --- | --- | --- | --- | --- | --- | --- | --- | --- | --- | --- | --- | --- | --- |
| **Jan-03** | | | | | | | | | | | | | | | |
|  | **Dipak** | **Roshan** | **Ishwori** | **Y.** | **Rudra** | **R.A.** | **Menuka** | **Khadga** | **Shyam** | **Sulochana** | **Kesab** | **Kiran** | **Padam** | **Nir BDR** | **Ram NR** |
| **Dipak** |  | **x.63** | **x** |  | **x** |  | **x** | **x** | **x** | **x** | **x** | **x** | **x** | **x** | **x** |
| **Roshan** | **x.63** |  | **x** |  | **x** |  | **x** | **x.68** | **x** | **x.65** | **x** | **x** | **x** | **x.68** | **x.65** |
| **Ishwori** | **x** | **x** |  |  | **x** |  | **x** | **x** | **x** | **x** | **x** | **x** | **x** | **x.62** | **x** |
| **Yadav** |  |  |  |  |  |  |  |  |  |  |  |  |  |  |  |
| **Rudra** | **x** | **x** | **x** |  |  |  | **x** | **x** | **x** | **x** | **x** | **x** | **x** | **x.68** | **x** |
| **Ram Asesh** |  |  |  |  |  |  |  |  |  |  |  |  |  |  |  |
| **Menuka** | **x** | **x** | **x** |  | **x** |  |  | **x** | **x** | **x** | **x** | **x** | **x** | **x** | **x** |
| **Khadga** | **x** | **x.68** | **x** |  | **x** |  | **x** |  | **x** | **x** | **x** | **x** | **x** | **x** | **x** |
| **Shyam** | **x** | **x** | **x** |  | **x** |  | **x** | **x** |  | **x** | **x** | **x.66** | **x** | **x.67** | **x.66** |
| **Sulochana** | **x** | **x.65** | **x** |  | **x** |  | **x** | **x** | **x** |  | **x** | **x** | **x** | **x** | **x** |
| **Kesab** | **x** | **x** | **x** |  | **x** |  | **x** | **x** | **x** | **x** |  | **x** | **x** | **x** | **x** |
| **Kiran** | **x** | **x** | **x** |  | **x** |  | **x** | **x** | **x.66** | **x** | **x** |  | **x** | **x** | **x** |
| **Padam** | **x** | **x** | **x** |  | **x** |  | **x** | **x** | **x** | **x** | **x** | **x** |  | **x** | **x** |
| **Nir BDR** | **x** | **x.68** | **x.62** |  | **x.68** |  | **x** | **x** | **x.67** | **x** | **x** | **x** | **x** |  | **x** |
| **Ram NR** | **x** | **x.65** | **x** |  | **x** |  | **x** | **x** | **x.66** | **x** | **x** | **x** | **x** | **x** |  |
|  |  |  |  |  |  |  |  |  |  |  |  |  |  |  |  |
| **For these standardization exercises the observers watched 30-minute video segments. The numbers are kappa scores of observers who had trouble with the social gesture code.** | | | | | | | | | | | | | | | |
|
|  |  |  |  |  |  |  |  |  |  |  |  |  |  |  |  |
| **Observation** | **Date** | **Child ID** |  |  |  |  |  |  |  |  |  |  |  |  |  |
| 1 | 1/23/2003 | 165146 |  |  |  |  |  |  |  |  |  |  |  |  |  |
| 2 | 1/28/2003 | 165022 |  |  |  |  |  |  |  |  |  |  |  |  |  |
| 3 | 1/28/2003 | 165146 |  |  |  |  |  |  |  |  |  |  |  |  |  |
| 4 | 1/28/2003 | 165227 |  |  |  |  |  |  |  |  |  |  |  |  |  |
| 5 | 1/28/2003 | 165256 |  |  |  |  |  |  |  |  |  |  |  |  |  |
| 6 | 1/29/2003 | 165022 |  |  |  |  |  |  |  |  |  |  |  |  |  |

| **Observer Standardization** | | | | | | | | | | | | | | | |
| --- | --- | --- | --- | --- | --- | --- | --- | --- | --- | --- | --- | --- | --- | --- | --- |
| **Jan-03** | | | | | | | | | | | | | | | |
|  | **Dipak** | **Roshan** | **Ishwori** | **Y.** | **Rudra** | **R.A.** | **Menuka** | **Khadga** | **Shyam** | **Sulochana** | **Kesab** | **Kiran** | **Padam** | **Nir BDR** | **Ram NR** |
| **Dipak** |  | **x.548** | **x** |  | **x.518** |  | **x** | **x** | **x.55** | **x** | **x** | **x** | **x** | **x** | **x** |
| **Roshan** | **x.548** |  | **x** |  | **x** |  | **x** | **x.56** | **x.548** | **x** | **x** | **x** | **x** | **x.61** | **x** |
| **Ishwori** | **x** | **x** |  |  | **x.58** |  | **x.668** | **x.574** | **x.690** | **x** | **x** | **x.676** | **x.60** | **x** | **x** |
| **Yadav** |  |  |  |  |  |  |  |  |  |  |  |  |  |  |  |
| **Rudra** | **x.518** | **x** | **x.58** |  |  |  | **x.582** | **x** | **A** | **x.635** | **x** | **x.666** | **x.582** | **B** | **x** |
| **Ram Asesh** |  |  |  |  |  |  |  |  |  |  |  |  |  |  |  |
| **Menuka** | **x** | **x** | **x.668** |  | **x.582** |  |  | **x** | **x.562** | **x** | **x** | **x** | **x.575** | **x** | **x** |
| **Khadga** | **x** | **x.56** | **x.574** |  | **x** |  | **x** |  | **x.537** | **x** | **x** | **x** | **x** | **x.472** | **x** |
| **Shyam** | **x.550** | **x.548** | **x.690** |  | **A** |  | **x.562** | **x.537** |  | **x.58** | **x** | **x** | **x.565** | **x.672** | **x.602** |
| **Sulochana** | **x** | **x** | **x** |  | **x.635** |  | **x** | **x** | **x.58** |  | **x** | **x** | **x** | **x** | **x** |
| **Kesab** | **x** | **x** | **x** |  | **x** |  | **x** | **x** | **x** | **x** |  | **x** | **x** | **x** | **x** |
| **Kiran** | **x** | **x** | **x.676** |  | **x.666** |  | **x** | **x** | **x** | **x** | **x** |  | **x.65** | **x** | **x** |
| **Padam** | **x** | **x** | **x.60** |  | **x.582** |  | **x.575** | **x** | **x.565** | **x** | **x** | **x.65** |  | **x.638** | **x** |
| **Nir BDR** | **x** | **x.61** | **x** |  | **B** |  | **x** | **x.472** | **x.672** | **x** | **x** | **x** | **x.638** |  | **x** |
| **Ram NR** | **x** | **x** | **x** |  | **x** |  | **x** | **x** | **x.602** | **x** | **x** | **x** | **x** | **x** |  |
| **For these standardization exercises the observers watched 1-hour video segments. Because there were problems with achieving standardization, the 30-minute exercise was counted as the primary standardization exercise. The numbers are the kappa scores for the observers who had trouble with the social gesture code. "A" and "B" mark observers who also had problems with the social play code.** | | | | | | | | | | | | | | | |
|
|
|
|  |  |  |  |  |  |  |  |  |  |  |  |  |  |  |  |
| **Observation** | **Date** | **Child ID** |  |  |  |  |  |  |  |  |  |  |  |  |  |
| 1 | 1/17/2003 | 165256 |  |  |  |  |  |  |  |  |  |  |  |  |  |
| 2 | 1/21/2003 | 165256 |  |  |  |  |  |  |  |  |  |  |  |  |  |
| 3 | 1/21/2003 | 183469 |  |  |  |  |  |  |  |  |  |  |  |  |  |
| 4 | 1/23/2003 | 165095 |  |  |  |  |  |  |  |  |  |  |  |  |  |
| 5 | 1/24/2003 | 165095 |  |  |  |  |  |  |  |  |  |  |  |  |  |
| 6 | 1/27/2003 | 165095 |  |  |  |  |  |  |  |  |  |  |  |  |  |
| 7 | 1/27/2003 | 165227 |  |  |  |  |  |  |  |  |  |  |  |  |  |
| 8 | 1/27/2003 | 165256 |  |  |  |  |  |  |  |  |  |  |  |  |  |

Motor Milestones Validation Studies

| Motor Milestones Validation Study October 2002 | | | | | | | |
| --- | --- | --- | --- | --- | --- | --- | --- |
| W | S | TLI Name | MM# TLI Saw | MM# Mom Told TLI | NNIPS Week | MM# Mom Told WD | NNIPS Wk # Checked |
| 1 | 1 | Dipak | 12 | 13 | 95 | 12 | 97 |
| 1 | 1 | Dipak | 12 | 13 | 95 | 14 | 97 |
| 1 | 1 | Dipak | 14 | 13 | 95 | 14 | 97 |
| 1 | 1 | Dipak | 13 | 12 | 95 | 14 | 97 |
| 1 | 1 | Dipak | 11 | 12 | 95 | 14 | 97 |
| 1 | 1 | Dipak | 13 | 14 | 95 | 14 | 97 |
| 1 | 1 | Dipak | 12 | 13 | 95 | 14 | 97 |
| 1 | 1 | Dipak | 12 | 12 | 95 | 14 | 97 |
| 1 | 1 | Dipak | 13 | 13 | 95 | 14 | 97 |
| 1 | 1 | Dipak | 12 | 12 | 95 | 13 (79) | 97 |
|  |  |  |  |  |  |  |  |
| 1 | 2 | Dipak | 13 | 12 | 95 | 14 | 97 |
| 1 | 2 | Dipak | 12 | 12 | 95 | 14 | 97 |
| 1 | 2 | Dipak | 11 | 12 | 95 | 14 | 97 |
| 1 | 2 | Dipak | 12 | 13 | 95 | 14 | 97 |
| 1 | 2 | Dipak | 11 | 12 | 95 | 14 | 97 |
| 1 | 2 | Dipak | 12 | 13 | 95 | 14 | 97 |
| 1 | 2 | Dipak | 12 | 13 | 95 | 14 | 97 |
| 1 | 2 | Dipak | 11 | 11 | 95 | 11 (76) | 97 |
| 1 | 2 | Dipak | 14 | 14 | 95 | 14 | 97 |
| 1 | 2 | Dipak | 13 | 14 | 95 | 14 | 97 |
| 1 | 2 | Dipak | 11 | 12 | 95 | 14 | 97 |
|  |  |  |  |  |  |  |  |
| 1 | 3 | Nir BDR | 12 | 12 | 95 | 12 (77) | 97 |
| 1 | 3 | Nir BDR | 13 | 14 | 95 | 14 | 97 |
| 1 | 3 | Nir BDR | 12 | 12 | 95 | 12 (55) | 97 |
| 1 | 3 | Nir BDR | 14 | 14 | 95 | 14 | 97 |
| 1 | 3 | Nir BDR | 14 | 14 | 95 | 14 | 97 |
| 1 | 3 | Nir BDR | 14 | 13 | 95 | 12 (54) | 97 |
| 1 | 3 | Nir BDR | 13 | 14 | 95 | 14 | 97 |
| 1 | 3 | Nir BDR | 14 | 14 | 95 | 14 | 97 |
| 1 | 3 | Nir BDR | 14 | 14 | 95 | 14 | 97 |
| 1 | 3 | Nir BDR | 13 | 13 | 95 | 14 | 97 |
| 1 | 3 | Nir BDR | 14 | 14 | 95 | 14 | 97 |
|  |  |  |  |  |  |  |  |
| 1 | 4 | Nir BDR | 14 | 13 | 95 | 14 | 97 |
| 1 | 4 | Nir BDR | 14 | 13 | 95 | 14 | 97 |
| 1 | 4 | Nir BDR | 14 | 14 | 95 | 14 | 97 |
| 1 | 4 | Nir BDR | 14 | 13 | 95 | 14 | 97 |
| 1 | 4 | Nir BDR | 14 | 14 | 95 | 14 | 97 |
| 1 | 4 | Nir BDR | 13 | 13 | 95 | 14 | 97 |
| 1 | 4 | Nir BDR | 14 | 13 | 95 | 14 | 97 |
| 1 | 4 | Nir BDR | 14 | 14 | 95 | 14 | 97 |
| 1 | 4 | Nir BDR | 14 | 14 | 95 | 14 | 97 |
| 1 | 4 | Nir BDR | 14 | 14 | 95 | 14 | 97 |
|  |  |  |  |  |  |  |  |
| 2 | 1 | Kesab | 14 | 14 | 95 | 14 | 97 |
| 2 | 1 | Kesab | 12 | 13 | 95 | 14 | 97 |
| 2 | 1 | Kesab | 13 | 14 | 95 | 14 | 97 |
| 2 | 1 | Kesab | 14 | 14 | 95 | 14 | 97 |
| 2 | 1 | Kesab | 12 | 13 | 95 | 14 | 97 |
| 2 | 1 | Kesab | 13 | 14 | 95 | 14 | 97 |
| 2 | 1 | Kesab | 12 | 13 | 95 | 13 (87) | 97 |
| 2 | 1 | Kesab | 14 | 14 | 95 | 14 | 97 |
| 2 | 1 | Kesab | 14 | 14 | 95 | 14 | 97 |
| 2 | 1 | Kesab | 12 | 14 | 95 | 14 | 97 |
|  |  |  |  |  |  |  |  |
| 2 | 2 | Kesab | 12 | 13 | 95 | 14 | 97 |
| 2 | 2 | Kesab | 12 | 13 | 95 | 14 | 97 |
| 2 | 2 | Kesab | 14 | 12 | 95 | 14 | 97 |
| 2 | 2 | Kesab | 12 | 13 | 95 | 14 | 97 |
| 2 | 2 | Kesab | 13 | 13 | 95 | 14 | 97 |
| 2 | 2 | Kesab | 12 | 12 | 95 | 14 | 97 |
| 2 | 2 | Kesab | 14 | 14 | 95 | 14 | 97 |
| 2 | 2 | Kesab | 12 | 14 | 95 | 14 | 97 |
| 2 | 2 | Kesab | 12 | 12 | 95 | 14 | 97 |
| 2 | 2 | Kesab | 14 | 14 | 95 | 14 | 97 |
|  |  |  |  |  |  |  |  |
| 2 | 3A | Kesab | 12 | 12 | 95 | 12 (83) | 97 |
| 2 | 3A | Kesab | 14 | 13 | 95 | 14 | 97 |
| 2 | 3A | Kesab | 12 | 12 | 95 | 14 | 97 |
| 2 | 3A | Kesab | 14 | 14 | 95 | 14 | 97 |
| 2 | 3A | Kesab | 11 | 12 | 95 | 14 | 97 |
| 2 | 3A | Kesab | 14 | 14 | 95 | 14 | 97 |
| 2 | 3A | Kesab | 14 | 14 | 95 | 14 | 97 |
| 2 | 3A | Kesab | 14 | 14 | 95 | 14 | 97 |
| 2 | 3A | Kesab | 14 | 14 | 95 | 14 | 97 |
| 2 | 3A | Kesab | 11 | 12 | 95 | 14 (96) | 97 |
|  |  |  |  |  |  |  |  |
| 2 | 3B | Kesab | 11 | 11 | 95 | 14 | 97 |
| 2 | 3B | Kesab | 12 | 13 | 95 | 14 | 97 |
| 2 | 3B | Kesab | 11 | 11 | 95 | 11 (85) | 97 |
| 2 | 3B | Kesab | 11 | 12 | 95 | 13 (90) | 97 |
| 2 | 3B | Kesab | 13 | 13 | 95 | 14 | 97 |
| 2 | 3B | Kesab | 12 | 13 | 95 | 14 | 97 |
| 2 | 3B | Kesab | 11 | 12 | 95 | 11 (57) | 97 |
| 2 | 3B | Kesab | 12 | 12 | 95 | 14 | 97 |
| 2 | 3B | Kesab | 12 | 13 | 95 | 14 | 97 |
| 2 | 3B | Kesab | 11 | 12 | 95 | 12 (62) | 97 |
|  |  |  |  |  |  |  |  |
| 3 | 1 | Nir BDR | 14 | 13 | 95 | 14 | 97 |
| 3 | 1 | Nir BDR | 14 | 13 | 95 | 14 | 97 |
| 3 | 1 | Nir BDR | 14 | 14 | 95 | 14 | 97 |
| 3 | 1 | Nir BDR | 14 | 14 | 95 | 14 | 97 |
| 3 | 1 | Nir BDR | 14 | 14 | 95 | 14 | 97 |
| 3 | 1 | Nir BDR | 9 | 9 | 95 |  | 97 |
| 3 | 1 | Nir BDR | 14 | 14 | 95 | 14 | 97 |
| 3 | 1 | Nir BDR | 13 | 13 | 95 | 14 | 97 |
| 3 | 1 | Nir BDR | 14 | 14 | 95 | 12 (92) | 97 |
| 3 | 1 | Nir BDR | 14 | 14 | 95 | 14 | 97 |
| 3 | 1 | Nir BDR | 14 | 14 | 95 | 14 | 97 |
|  |  |  |  |  |  |  |  |
| 3 | 2 | Nir BDR | 14 | 14 | 95 | 14 | 97 |
| 3 | 2 | Nir BDR | 14 | 14 | 95 | 14 | 97 |
| 3 | 2 | Nir BDR | 14 | 14 | 95 | 14 | 97 |
| 3 | 2 | Nir BDR | 14 | 14 | 95 | 14 | 97 |
| 3 | 2 | Nir BDR | 14 | 14 | 95 | 13 (-) | 97 |
| 3 | 2 | Nir BDR | 12 | 12 | 95 | 13 (56) | 97 |
| 3 | 2 | Nir BDR | 12 | 12 | 95 | 14 | 97 |
| 3 | 2 | Nir BDR | 12 | 12 | 95 | 13 (94) | 97 |
| 3 | 2 | Nir BDR | 14 | 13 | 95 | 14 | 97 |
| 3 | 2 | Nir BDR | 12 | 13 | 95 | 14 (93) | 97 |
|  |  |  |  |  |  |  |  |
| 4 | 1 | Kesab | 12 | 14 | 95 | 14 | 97 |
| 4 | 1 | Kesab | 14 | 14 | 95 | 14 | 97 |
| 4 | 1 | Kesab | 14 | 13 | 95 | 14 | 97 |
| 4 | 1 | Kesab | 14 | 13 | 95 | 14 | 97 |
| 4 | 1 | Kesab | 14 | 14 | 95 | 14 | 97 |
| 4 | 1 | Kesab | 14 | 14 | 95 | 14 | 97 |
| 4 | 1 | Kesab | 12 | 14 | 95 | 14 | 97 |
| 4 | 1 | Kesab | 14 | 14 | 95 | 14 | 97 |
| 4 | 1 | Kesab | 14 | 14 | 95 | 14 | 97 |
| 4 | 1 | Kesab | 14 | 14 | 95 | 14 | 97 |
|  |  |  |  |  |  |  |  |
| 4 | 2 | Kesab | 14 | 14 | 95 | 14 | 97 |
| 4 | 2 | Kesab | 14 | 13 | 95 | 14 | 97 |
| 4 | 2 | Kesab | 14 | 14 | 95 | 14 | 97 |
| 4 | 2 | Kesab | 14 | 14 | 95 | 14 | 97 |
| 4 | 2 | Kesab | 12 | 13 | 95 | 12 (63) | 97 |
| 4 | 2 | Kesab | 11 | 12 | 95 | 12 (63) | 97 |
| 4 | 2 | Kesab | 12 | 14 | 95 | 12 (64) | 97 |
| 4 | 2 | Kesab | 14 | 14 | 95 | 14 | 97 |
| 4 | 2 | Kesab | 14 | 13 | 95 | 14 | 97 |
| 4 | 2 | Kesab | 13 | 14 | 95 | 14 | 97 |
|  |  |  |  |  |  |  |  |
| 5 | 1 | Ram NR | 12 | 14 | 96 | 14 | 97 |
| 5 | 1 | Ram NR | 12 | 14 | 96 | 14 | 97 |
| 5 | 1 | Ram NR | 12 | 12 | 96 | 13 (51) | 97 |
| 5 | 1 | Ram NR | 12 | 13 | 96 | 14 | 97 |
| 5 | 1 | Ram NR | 12 | 12 | 96 | 14 | 97 |
| 5 | 1 | Ram NR | 12 | 13 | 96 | 14 | 97 |
| 5 | 1 | Ram NR | 12 | 13 | 96 | 14 | 97 |
| 5 | 1 | Ram NR | 12 | 13 | 96 | 14 (97) | 97 |
| 5 | 1 | Ram NR | 12 | 14 | 96 | 14 | 97 |
| 5 | 1 | Ram NR | 14 | 13 | 96 | 14 | 97 |
|  |  |  |  |  |  |  |  |
| 6 | 1 | Kesab | 12 | 12 | 96 | 14 | 97 |
| 6 | 1 | Kesab | 14 | 13 | 96 | 14 | 97 |
| 6 | 1 | Kesab | 14 | 12 | 96 | 14 | 97 |
| 6 | 1 | Kesab | 14 | 14 | 96 | 14 | 97 |
| 6 | 1 | Kesab | 14 | 14 | 96 | 14 | 97 |
| 6 | 1 | Kesab | 14 | 14 | 96 | 14 | 97 |
| 6 | 1 | Kesab | 12 | 14 | 96 | 14 | 97 |
| 6 | 1 | Kesab | 12 | 14 | 96 | 14 (93) | 97 |
| 6 | 1 | Kesab | 14 | 14 | 96 | 14 | 97 |
| 6 | 1 | Kesab | 14 | 14 | 96 | 14 | 97 |
|  |  |  |  |  |  |  |  |
| 6 | 2 | Ram NR | 11 | 11 | 95 | 11 (90) | 97 |
| 6 | 2 | Ram NR | 14 | 14 | 95 | 14 | 97 |
| 6 | 2 | Ram NR | 14 | 14 | 95 | 14 | 97 |
| 6 | 2 | Ram NR | 11 | 12 | 95 | 12 (64) | 97 |
| 6 | 2 | Ram NR | 14 | 14 | 95 | 14 | 97 |
| 6 | 2 | Ram NR | 12 | 12 | 95 | 14 | 97 |
| 6 | 2 | Ram NR | 14 | 14 | 95 | 14 | 97 |
| 6 | 2 | Ram NR | 12 | 12 | 95 | 14 | 97 |
| 6 | 2 | Ram NR | 12 | 12 | 95 | 13 (81) | 97 |
| 6 | 2 | Ram NR | 11 | 12 | 95 | 12 (59) | 97 |
|  |  |  |  |  |  |  |  |
| 6 | 3 | Ram NR | 14 | 14 | 95 | 14 | 97 |
| 6 | 3 | Ram NR | 12 | 14 | 95 | 14 | 97 |
| 6 | 3 | Ram NR | 9 | 9 | 95 | 14 | 97 |
| 6 | 3 | Ram NR | 14 | 14 | 95 | 14 | 97 |
| 6 | 3 | Ram NR | 11 | 12 | 95 | 14 | 97 |
| 6 | 3 | Ram NR | 12 | 14 | 95 | 14 | 97 |
| 6 | 3 | Ram NR | 12 | 12 | 95 | 14 | 97 |
| 6 | 3 | Ram NR | 11 | 12 | 95 | 14 | 97 |
| 6 | 3 | Ram NR | 12 | 13 | 95 | 14 | 97 |
| 6 | 3 | Ram NR | 14 | 14 | 95 | 14 | 97 |
|  |  |  |  |  |  |  |  |
| 7 | 1 | Ram NR | 11 | 12 | 95 | 14 | 97 |
| 7 | 1 | Ram NR | 11 | 12 | 95 | 14 | 97 |
| 7 | 1 | Ram NR | 12 | 13 | 95 | 14 | 97 |
| 7 | 1 | Ram NR | 12 | 13 | 95 | 14 | 97 |
| 7 | 1 | Ram NR | 12 | 13 | 95 | 14 | 97 |
| 7 | 1 | Ram NR | 11 | 12 | 95 | 14 | 97 |
| 7 | 1 | Ram NR | 12 | 14 | 95 | 14 | 97 |
| 7 | 1 | Ram NR | 12 | 13 | 95 | 14 | 97 |
| 7 | 1 | Ram NR | 10 | 10 | 95 | 10 (73) | 97 |
| 7 | 1 | Ram NR | 12 | 13 | 95 | 14 | 97 |
|  |  |  |  |  |  |  |  |
| 8 | 1 | Ram NR | 11 | 12 | 95 | 14 | 97 |
| 8 | 1 | Ram NR | 14 | 13 | 95 | 14 | 97 |
| 8 | 1 | Ram NR | 10 | 10 | 95 | 14 | 97 |
| 8 | 1 | Ram NR | 11 | 12 | 95 | 14 | 97 |
| 8 | 1 | Ram NR | 10 | 11 | 95 | 14 | 97 |
| 8 | 1 | Ram NR | 12 | 13 | 95 | 14 | 97 |
| 8 | 1 | Ram NR | 11 | 13 | 95 | 14 | 97 |
| 8 | 1 | Ram NR | 14 | 14 | 95 | 14 | 97 |
| 8 | 1 | Ram NR | 12 | 12 | 95 | 14 | 97 |
| 8 | 1 | Ram NR | 12 | 13 | 95 | 14 | 97 |
|  |  |  |  |  |  |  |  |
|  |  |  |  |  |  |  |  |
| 8 | 2 | Ram NR | 11 | 12 | 95 | 14 | 97 |
| 8 | 2 | Ram NR | 11 | 12 | 95 | 12 (85) | 97 |
| 8 | 2 | Ram NR | 9 | 9 | 95 | 9 (86) | 97 |
| 8 | 2 | Ram NR | 14 | 14 | 95 | 14 | 97 |
| 8 | 2 | Ram NR | 12 | 14 | 95 | 14 | 97 |
| 8 | 2 | Ram NR | 12 | 14 | 95 | 14 | 97 |
| 8 | 2 | Ram NR | 11 | 12 | 95 | 14 | 97 |
| 8 | 2 | Ram NR | 14 | 14 | 95 | 14 | 97 |
| 8 | 2 | Ram NR | 11 | 12 | 95 | 12 (86) | 97 |
| 8 | 2 | Ram NR | 11 | 12 | 95 | 12 (-) | 97 |
|  |  |  |  |  |  |  |  |
|  |  |  |  |  |  |  |  |
| 9 | 1 | Kesab | 12 | 14 | 95 | 14 (91) | 97 |
| 9 | 1 | Kesab | 12 | 14 | 95 | 14 | 97 |
| 9 | 1 | Kesab | 14 | 14 | 95 | 14 | 97 |
| 9 | 1 | Kesab | 14 | 14 | 95 | 14 | 97 |
| 9 | 1 | Kesab | 14 | 14 | 95 | 14 | 97 |
| 9 | 1 | Kesab | 14 | 14 | 95 | 14 | 97 |
| 9 | 1 | Kesab | 14 | 14 | 95 | 14 | 97 |
| 9 | 1 | Kesab | 12 | 14 | 95 | 14 | 97 |
| 9 | 1 | Kesab | 12 | 14 | 95 | 14 | 97 |
| 9 | 1 | Kesab | 14 | 13 | 95 | 14 | 97 |
|  |  |  |  |  |  |  |  |
|  |  |  |  |  |  |  |  |
| 9 | 2 | Kesab | 14 | 14 | 95 | 14 | 97 |
| 9 | 2 | Kesab | 14 | 14 | 95 | 14 | 97 |
| 9 | 2 | Kesab | 14 | 14 | 95 | 14 | 97 |
| 9 | 2 | Kesab | 14 | 14 | 95 | 14 | 97 |
| 9 | 2 | Kesab | 14 | 14 | 95 | 14 | 97 |
| 9 | 2 | Kesab | 14 | 14 | 95 | 14 | 97 |
| 9 | 2 | Kesab | 12 | 14 | 95 | 14 | 97 |
| 9 | 2 | Kesab | 14 | 14 | 95 | 14 | 97 |
| 9 | 2 | Kesab | 11 | 12 | 95 | 14 | 95 |
| 9 | 2 | Kesab | 14 | 14 | 95 | 14 | 97 |
|  |  |  |  |  |  |  |  |
|  |  |  |  |  |  |  |  |
| 9 | 3 | Nir BDR | 14 | 14 | 95 | 14 | 97 |
| 9 | 3 | Nir BDR | 14 | 14 | 95 | 14 | 97 |
| 9 | 3 | Nir BDR | 14 | 14 | 95 | 14 | 97 |
| 9 | 3 | Nir BDR | 14 | 14 | 95 | 14 | 97 |
| 9 | 3 | Nir BDR | 14 | 14 | 95 | 14 | 97 |
| 9 | 3 | Nir BDR | 12 | 12 | 95 | 14 | 97 |
| 9 | 3 | Nir BDR | 14 | 14 | 95 | 14 | 97 |
| 9 | 3 | Nir BDR | 14 | 14 | 95 | 14 | 97 |
| 9 | 3 | Nir BDR | 14 | 14 | 95 | 14 | 97 |
| 9 | 3 | Nir BDR | 12 | 12 | 95 | 14 | 97 |
|  |  | Nir BDR | 14 | 14 | 95 | 14 | 97 |
|  |  |  |  |  |  |  |  |
| 9 | 4 | Nir BDR | 14 | 14 | 95 | 14 | 97 |
| 9 | 4 | Nir BDR | 14 | 14 | 95 | 14 | 97 |
| 9 | 4 | Nir BDR | 14 | 14 | 95 | 14 | 97 |
| 9 | 4 | Nir BDR | 14 | 13 | 95 | 14 | 97 |
| 9 | 4 | Nir BDR | 14 | 14 | 95 | 14 | 97 |
| 9 | 4 | Nir BDR | 14 | 14 | 95 | 14 | 97 |
| 9 | 4 | Nir BDR | 14 | 13 | 95 | 14 | 97 |
| 9 | 4 | Nir BDR | 14 | 14 | 95 | 14 | 97 |
| 9 | 4 | Nir BDR | 14 | 14 | 95 | 13 (95) | 97 |
| 9 | 4 | Nir BDR | 14 | 14 | 95 | 14 | 97 |
| 9 | 4 | Nir BDR | 14 | 14 | 95 | 14 | 97 |
| 9 | 4 | Nir BDR | 14 | 14 | 95 | 14 | 97 |
|  |  |  |  |  |  |  |  |

| Motor Milestones Validation Study July 2002 | | | | | | | | |
| --- | --- | --- | --- | --- | --- | --- | --- | --- |
| W | S | TLI Name | MM# TLI Saw | MM# Mom Told TLI | NNIPS Week | MM# Mom Told WD | NNIPS Wk # Checked | Comments |
| 1 | 1 | Yadav | 8 | 8 | 81 | 7 & 8 | 82 |  |
| 1 | 1 | Yadav | 12 | 14 | 81 | 14 (81) | 82 |  |
| 1 | 1 | Yadav | 12 | 12 | 81 | 12 (51) | 82 |  |
| 1 | 1 | Yadav | 11 | 11 | 81 | 11 (68) | 82 |  |
| 1 | 1 | Yadav | 5 | 5 | 81 | 3 (81) | 82 |  |
| 1 | 1 | Yadav | 11 | 11 | 81 | 11 (82) | 82 |  |
| 1 | 1 | Yadav | 14 | 14 | 81 | 13 (79) | 82 |  |
| 1 | 1 | Yadav | 12 | 12 | 81 | 11 (69) | 82 |  |
| 1 | 1 | Yadav | 9 | 10 | 81 | 10 (79) | 82 |  |
| 1 | 1 | Yadav | 4 | 4 | 81 | 9 &10 (81) | 82 |  |
|  |  |  |  |  |  |  |  |  |
| 1 | 2 | Ram NR | 9 | 9 | 81 | 9 (78) | 82 |  |
| 1 | 2 | Ram NR | 3 | 3 | 81 | 2 (79) | 82 |  |
| 1 | 2 | Ram NR | 9 | 10 | 81 | 11 (96) | 82 |  |
| 1 | 2 | Ram NR | 10 | 10 | 81 | 10 (64) | 82 |  |
| 1 | 2 | Ram NR | 11 | 12 | 81 | 12 (72) | 82 |  |
| 1 | 2 | Ram NR | 9 | 9 | 81 | 9 (--) | 82 |  |
| 1 | 2 | Ram NR | 2 | 2 | 81 | 3 (82) | 82 |  |
| 1 | 2 | Ram NR | 11 | 11 | 81 | 11 (73) | 82 |  |
| 1 | 2 | Ram NR | 12 | 12 | 81 | 12 (72) | 82 |  |
| 1 | 2 | Ram NR | 9 | 10 | 81 | 10 (76) | 82 |  |
|  |  |  |  |  |  |  |  |  |
| 1 | 3 | Ram NR | 11 | 12 | 81 | 12 (76) | 82 |  |
| 1 | 3 | Ram NR | 11 | 12 | 81 | 12 (79) | 82 |  |
| 1 | 3 | Ram NR | 9 | 9 | 81 | 8 (75) | 82 |  |
| 1 | 3 | Ram NR | 14 | 14 | 81 | 14 | 82 | Sent to KTM |
| 1 | 3 | Ram NR | 9 | 9 | 81 | 8 (75) | 82 |  |
| 1 | 3 | Ram NR | 12 | 13 | 81 | 13 (51) | 82 |  |
| 1 | 3 | Ram NR | 9 | 9 | 81 | 9 (51) | 82 |  |
| 1 | 3 | Ram Asesh | 9 | 9 | 82 | 9 (81) | 82 |  |
| 1 | 3 | Ram NR | 9 | 10 | 81 | 10 (72) | 82 |  |
| 1 | 3 | Ram NR | 2 | 2 | 81 | 2 (81) | 82 |  |
| 1 | 3 | Ram NR | 11 | 12 | 81 | 12 (72) | 82 |  |
|  |  |  |  |  |  |  |  |  |
| 1 | 4 | Ram Asesh | 11 | 11 | 82 | 12 (80) | 82 |  |
| 1 | 4 | Yadav | 12 | 12 | 81 | 12 (64) | 82 |  |
| 1 | 4 | Yadav | 10 | 10 | 81 | 9 (69) | 82 |  |
| 1 | 4 | Yadav | 10 | 10 | 81 | 10 (80) | 82 |  |
| 1 | 4 | Yadav | 10 | 10 | 81 | 10 (74) | 82 |  |
| 1 | 4 | Yadav | 11 | 12 | 81 | 14 | 82 |  |
| 1 | 4 | Yadav | 3 | 3 | 81 | 3 (81) | 82 |  |
| 1 | 4 | Yadav | 12 | 12 | 81 | 12 (81) | 82 |  |
| 1 | 4 | Yadav | 10 | 11 | 81 | 11 (78) | 82 |  |
| 1 | 4 | Yadav | 0 | 0 | 81 | 0 | 82 |  |
| 1 | 4 | Yadav | 3 | 3 | 81 | 4 (82) | 82 |  |
|  |  |  |  |  |  |  |  |  |
| 2 | 1 | Kesab | 5 | 5 | 81 | 3 (81) | 82 |  |
| 2 | 1 | Kesab | 12 | 12 | 91 | 12 (74) | 82 |  |
| 2 | 1 | Kesab | 12 | 12 | 81 | 13 (74) | 82 |  |
| 2 | 1 | Kesab | 13 | 13 | 81 | 13 (55) | 82 |  |
| 2 | 1 | Kesab | 9 | 9 | 81 | 8 (80) | 82 |  |
| 2 | 1 | Kesab | 11 | 11 | 81 | 11 (68) | 82 |  |
| 2 | 1 | Kesab | 9 | 10 | 81 | 9 (81) | 82 |  |
| 2 | 1 | Kesab | 11 | 11 | 81 | 11 (80) | 82 |  |
| 2 | 1 | Kesab | 12 | 13 | 81 | 13 & 14 (81) | 82 |  |
| 2 | 1 | Kesab | 12 | 12 | 81 | 13 & 14 (81) | 82 |  |
| 2 | 1 | Ram NR | 11 | 12 | 82 | 13 (75) | 82 |  |
|  |  |  |  |  |  |  |  |  |
| 2 | 2 | Dipak | 9 | 10 | 81 | 10 (81) | 82 |  |
| 2 | 2 | Dipak | 9 | 9 | 81 | 9 (76) | 82 |  |
| 2 | 2 | Dipak | 10 | 11 | 81 | 11 (76) | 82 |  |
| 2 | 2 | Dipak | 9 | 9 | 81 | 9 (73) | 82 |  |
| 2 | 2 | Dipak | 2 | 2 | 81 | 1 (77) | 82 |  |
| 2 | 2 | Dipak | 12 | 12 | 81 | 14 (76) | 82 |  |
| 2 | 2 | Dipak | 11 | 11 | 81 | 13 (75) | 82 |  |
| 2 | 2 | Ram NR | 9 | 10 | 82 | 10 (76) | 82 |  |
| 2 | 2 | Dipak | 10 | 10 | 81 | 11 (82) | 82 |  |
| 2 | 2 | Dipak | Didn't see | 11 | 81 | 11 (80) | 82 |  |
| 2 | 2 | Dipak | 9 | 9 | 81 | 8 (80) | 82 |  |
|  |  |  |  |  |  |  |  |  |
| 2 | 3 | Ram Asesh | 11 | 11 | 81 | 11 (67) | 82 |  |
| 2 | 3 | Ram Asesh | 10 | 10 | 81 | 9 (61) | 82 |  |
| 2 | 3 | Ram NR | 9 | 9 | 81 | 9 (80) | 82 |  |
| 2 | 3 | Ram NR | 3 | 3 | 81 | 4 (79) | 82 |  |
| 2 | 3 | Ram NR | 11 | 12 | 81 | 13 (80) | 82 |  |
| 2 | 3 | Ram NR | 9 | 9 | 81 | 8 (79) | 82 |  |
| 2 | 3 | Ram NR | 2 | 2 | 81 | 2 (77) | 82 |  |
| 2 | 3 | Ram NR | 11 | 11 | 81 | 12 (71) | 82 |  |
| 2 | 3 | Ram NR | 9 | 11 | 81 | 9 (80) | 82 |  |
| 2 | 3 | Ram NR | 1 | 2 | 81 | 0 | 82 |  |
| 2 | 3 | Yadav | 8 | 9 | 81 | 9 (74) | 82 |  |
| 2 | 3 | Yadav | 4 | 5 | 81 | 5 (60) | 82 |  |
| 2 | 3 | Kesab | 12 | 11 | 81 | 12 (66) | 82 |  |
| 2 | 3 | Yadav | 8 | 8 | 81 | 7 & 8 (81) | 82 |  |
| 2 | 3 | Yadav | 11 | 11 | 81 | 12 (60) | 82 |  |
| 2 | 3 | Yadav | 9 | 8 | 81 | 7 & 8 (81) | 82 |  |
| 2 | 3 | Yadav | 9 | 10 | 81 | 10 (79) | 82 |  |
| 2 | 3 | Kesab | 11 | 12 | 81 | 12 (64) | 82 |  |
| 2 | 3 | Yadav | 11 | 11 | 81 | 11 (55) | 82 |  |
| 2 | 3 | Kesab | 11 | 12 | 81 | 12 (79) | 82 |  |
|  |  |  |  |  |  |  |  |  |
| 3 | 1 | Ram NR | 9 | 9 | 82 | 5 (82) | 82 |  |
| 3 | 1 | Ram NR | 11 | 12 | 82 | 12 (66) | 82 |  |
| 3 | 1 | Ram NR | 9 | 9 | 82 | 9 (61) | 82 |  |
| 3 | 1 | Ram NR | 3 | 4 | 82 | 2 (79) | 82 |  |
| 3 | 1 | Ram NR | 9 | 9 | 82 | 9 (75) | 82 |  |
| 3 | 1 | Dipak | 12 | 12 | 82 | 12 (66) | 82 |  |
| 3 | 1 | Dipak | 2 | 2 | 82 | 1 (75) | 82 |  |
| 3 | 1 | Dipak | 12 | 12 | 82 | 12 (60) | 82 |  |
| 3 | 1 | Dipak | 2 | 2 | 82 | 1 (76) | 82 |  |
| 3 | 1 | Dipak | 8 | 8 | 82 | 3 (77) | 82 |  |
| 3 | 1 | Dipak | 3 | 3 | 82 | 3 (82) | 82 |  |
|  |  |  |  |  |  |  |  |  |
| 3 | 2 | Dipak | 9 | 9 | 81 | 9 (82) | 82 |  |
| 3 | 2 | Dipak | 12 | 13 | 81 | 12 (--) | 82 |  |
| 3 | 2 | Dipak | 9 | 9 | 81 | 3 (79) | 82 |  |
| 3 | 2 | Dipak | 11 | 12 | 81 | 12 (81) | 82 |  |
| 3 | 2 | Dipak | 9 | 9 | 81 | 9 (74) | 82 |  |
| 3 | 2 | Dipak | 11 | 12 | 81 | 12 (56) | 82 |  |
| 3 | 2 | Dipak | 12 | 12 | 81 | 12 (58) | 82 |  |
| 3 | 2 | Dipak | 6 | 6 | 81 | 4 (74) | 82 |  |
| 3 | 2 | Dipak | 11 | 12 | 81 | 11 (57) | 82 |  |
| 3 | 2 | Dipak | 12 | 12 | 81 | 11 (65) | 82 |  |
| 3 | 2 | Dipak | 9 | 9 | 81 | 10 (81) | 82 |  |
|  |  |  |  |  |  |  |  |  |
| 4 | 1 | Dipak | 8 | 8 | 81 | 8 (82) | 82 |  |
| 4 | 1 | Dipak | 10 | 10 | 81 | 9 (--) | 82 |  |
| 4 | 1 | Dipak | Didn't see | 11 | 81 | 11 (49) | 82 |  |
| 4 | 1 | Dipak | 9 | 9 | 81 | 7 (76) | 82 |  |
| 4 | 1 | Dipak | 9 | 9 | 81 | 12 (70) | 82 |  |
| 4 | 1 | Dipak | Didn't see | 12 | 81 | 9 (75) | 82 |  |
| 4 | 1 | Dipak | 11 | 12 | 81 | 12 (75) | 82 |  |
| 4 | 1 | Dipak | 12 | 12 | 81 | 11 (56) | 82 |  |
| 4 | 1 | Dipak | 8 | 8 | 81 | 9 (79) | 82 |  |
| 4 | 1 | Yadav | 11 | 12 | 82 | 12 (73) | 82 |  |
|  |  |  |  |  |  |  |  |  |
| 4 | 2 | Dipak | 8 | 8 | 81 | 8 (74) | 82 |  |
| 4 | 2 | Dipak | 8 | 8 | 81 | 7 (74) | 82 |  |
| 4 | 2 | Dipak | 11 | 11 | 81 | 11 (74) | 82 |  |
| 4 | 2 | Dipak | 11 | 12 | 81 | 12 (75) | 82 |  |
| 4 | 2 | Dipak | 11 | 11 | 81 | 9 (64) | 82 |  |
| 4 | 2 | Dipak | 8 | 8 | 81 | 7 (78) | 82 |  |
| 4 | 2 | Dipak | 12 | 12 | 81 | 13 (82) | 82 |  |
| 4 | 2 | Dipak | Didn't see | 12 | 81 | 13 (59) | 82 |  |
| 4 | 2 | Dipak | 7 | 7 | 81 | 6 (59) | 82 |  |
| 4 | 2 | Yadav | Didn't see | 10 | 82 | 10 (82) | 82 |  |
|  |  |  |  |  |  |  |  |  |
| 5 | 1 | Dipak | 11 | 11 | 81 | 11 (74) | 82 |  |
| 5 | 1 | Dipak | 12 | 12 | 81 | 12 (57) | 82 |  |
| 5 | 1 | Dipak | 8 | 8 | 81 | 10 (54) | 82 |  |
| 5 | 1 | Dipak | 12 | 12 | 81 | 12 (51) | 82 |  |
| 5 | 1 | Dipak | 12 | 12 | 81 | 12 (57) | 82 |  |
| 5 | 1 | Dipak | 12 | 12 | 81 | 12 (71) | 82 |  |
| 5 | 1 | Dipak | 12 | 12 | 81 | 11 (79) | 82 |  |
| 5 | 1 | Dipak | 10 | 10 | 81 | 9 (51) | 82 |  |
| 5 | 1 | Dipak | 11 | 11 | 81 | 11 (49) | 82 |  |
| 5 | 1 | Dipak | 2 | 2 | 81 | 2 (77) | 82 |  |
| 5 | 1 | Dipak | 12 | 12 | 81 | 12 (80) | 82 |  |
| 5 | 1 | Dipak | 11 | 11 | 81 | 11 (72) | 82 |  |
|  |  |  |  |  |  |  |  |  |
| 6 | 1 | Ram Asesh | 9 | 9 | 82 | 11 (81) | 82 |  |
| 6 | 1 | Yadav | 11 | 12 | 82 | 12 (79) | 82 |  |
| 6 | 1 | Yadav | 11 | 12 | 82 | 14 | 82 | sent in KTM |
| 6 | 1 | Yadav | 11 | 12 | 82 | 12 (71) | 82 |  |
| 6 | 1 | Ram Asesh | 9 | 8 | 82 | 6 (80) | 82 | week 81 = 05 code |
| 6 | 1 | Ram Asesh | 12 | 13 | 82 | 13 (55) | 82 |  |
| 6 | 1 | Ram Asesh | 3 | 11 | 82 | 2 (75) | 82 |  |
| 6 | 1 | Ram Asesh | 11 | 11 | 82 | 11 (62) | 82 |  |
| 6 | 1 | Yadav | 8 | 8 | 82 | 8 (82) | 82 |  |
| 6 | 1 | Yadav | 9 | 9 | 82 | 9 (78) | 82 |  |
|  |  |  |  |  |  |  |  |  |
| 6 | 2 | Ram Asesh | 10 | 10 | 82 | 9 (65) | 82 |  |
| 6 | 2 | Ram Asesh | 12 | 11 | 82 | 12 (79) | 82 |  |
| 6 | 2 | Ram Asesh | 12 | 12 | 82 | 14 | 82 |  |
| 6 | 2 | Yadav | 4 | 9 | 82 | 6 (77) | 82 | week 82 = 05 code |
| 6 | 2 | Ram Asesh | 11 | 11 | 82 | 11 (69) | 82 |  |
| 6 | 2 | Ram Asesh | 2 | 2 | 82 | 1 (77) | 82 |  |
| 6 | 2 | Yadav | 3 | 6 | 82 | 3 (75) | 82 |  |
| 6 | 2 | Ram Asesh | 10 | 10 | 82 | 11 (62) | 82 |  |
| 6 | 2 | Yadav | 11 | 11 | 82 | 11 (71) | 82 |  |
| 6 | 2 | Yadav | 10 | 10 | 82 | 9 (78) | 82 |  |
|  |  |  |  |  |  |  |  |  |
| 6 | 3 | Ram NR | 11 | 11 | 81 | 11 (77) | 82 |  |
| 6 | 3 | Ram NR | 11 | 12 | 81 | 12 (76) | 82 |  |
| 6 | 3 | Ram NR | 6 | 6 | 81 | 3 (77) | 82 | Mom said 6, WD didn't believe |
| 6 | 3 | Ram NR | 9 | 9 | 81 | 8 (74) | 82 |  |
| 6 | 3 | Ram NR | 7 | 7 | 81 | 7 (81) | 82 |  |
| 6 | 3 | Ram NR | 7 | 7 | 81 | 9 (58) | 82 |  |
| 6 | 3 | Ram NR | 12 | 12 | 81 | 12 (82) | 82 |  |
| 6 | 3 | Ram NR | 11 | 12 | 81 | 12 (74) | 82 |  |
| 6 | 3 | Ram NR | 6 | 6 | 81 | 3 (73) | 82 |  |
| 6 | 3 | Ram NR | 11 | 12 | 81 | 12 (51) | 82 |  |
|  |  |  |  |  |  |  |  |  |
| 7 | 1 | Ram Asesh | 12 | 12 | 81 | 13 (75) | 82 |  |
| 7 | 1 | Ram Asesh | 10 | 10 | 81 | 11 (82) | 82 |  |
| 7 | 1 | Ram Asesh | 9 | 9 | 81 | 10 (82) | 82 |  |
| 7 | 1 | Ram Asesh | 9 | 9 | 81 | 9 (65) | 82 |  |
| 7 | 1 | Ram Asesh | 9 | 9 | 81 | 13 (80) | 82 |  |
| 7 | 1 | Ram Asesh | 11 | 11 | 81 | 12 (78) | 82 |  |
| 7 | 1 | Ram Asesh | 11 | 11 | 81 | 4 (79) | 82 | WD: asked 13 yr. old brother |
| 7 | 1 | Ram Asesh | 8 | 9 | 81 | 10 (73) | 82 |  |
| 7 | 1 | Ram Asesh | 9 | 10 | 81 | 6 (75) | 82 |  |
| 7 | 1 | Ram Asesh | 6 | 6 | 81 | 5 (80) | 82 |  |
|  |  |  |  |  |  |  |  |  |
| 8 | 1 | Yadav | 10 | 10 | 81 | 11 (81) | 82 |  |
| 8 | 1 | Yadav | 8 | 8 | 81 | 8 (75) | 82 |  |
| 8 | 1 | Yadav | 11 | 11 | 81 | 12 (77) | 82 |  |
| 8 | 1 | Yadav | 8 | 8 | 81 | 8 (82) | 82 |  |
| 8 | 1 | Yadav | 11 | 11 | 81 | 12 (75) | 82 |  |
| 8 | 1 | Yadav | 10 | 10 | 81 | 10 (75) | 82 |  |
| 8 | 1 | Yadav | 7 | 8 | 81 | 8 (82) | 82 |  |
| 8 | 1 | Yadav | 10 | 10 | 81 | 10 (63) | 82 |  |
| 8 | 1 | Yadav | 12 | 13 | 81 | 12 (51) | 82 |  |
| 8 | 1 | Yadav | 1 | 2 | 81 | 2 (77) | 82 |  |
|  |  |  |  |  |  |  |  |  |
| 8 | 2 | Yadav | 8 | 9 | 81 | 8 (78) | 82 |  |
| 8 | 2 | Yadav | 12 | 14 | 81 | 12 (--) | 82 |  |
| 8 | 2 | Yadav | 10 | 10 | 81 | 9 (81) | 82 |  |
| 8 | 2 | Yadav | 5 | 5 | 81 | 8 (71) | 82 | Can't do 6 & 7 |
| 8 | 2 | Yadav | 12 | 14 | 81 | 12 (--) | 82 |  |
| 8 | 2 | Yadav | 10 | 10 | 81 | 9 (77) | 82 |  |
| 8 | 2 | Yadav | 9 | 9 | 81 | 9 (77) | 82 |  |
| 8 | 2 | Yadav | 11 | 11 | 81 | 11 (50) | 82 |  |
| 8 | 2 | Yadav | 9 | 9 | 81 | 6 (56) | 82 |  |
| 8 | 2 | Yadav | 3 | 5 | 81 | 3 (77) | 82 |  |
|  |  |  |  |  |  |  |  |  |
| 9 | 1 | Ram Asesh | 12 | 12 | 81 | 12 (--) | 82 |  |
| 9 | 1 | Yadav | 11 | 12 | 82 | 12 (--) | 82 |  |
| 9 | 1 | Ram Asesh | 9 | 9 | 81 | 9 (79) | 82 |  |
| 9 | 1 | Ram Asesh | 9 | 9 | 81 | 9 (80) | 82 |  |
| 9 | 1 | Ram Asesh | 12 | 11 | 81 | 13 (76) | 82 |  |
| 9 | 1 | Ram Asesh | 12 | 13 | 81 | 14 (81) | 82 |  |
| 9 | 1 | Ram Asesh | 13 | 13 | 81 | 14 (81) | 82 |  |
| 9 | 1 | Ram Asesh | 2 | 2 | 81 | 2 (78) | 82 |  |
| 9 | 1 | Ram Asesh | 2 | 2 | 81 | 2 (82) | 82 |  |
| 9 | 1 | Ram Asesh | 12 | 12 | 81 | 12 (76) | 82 |  |
| 9 | 1 | Ram Asesh | 10 | 10 | 81 | 10 (78) | 82 |  |
|  |  |  |  |  |  |  |  |  |
| 9 | 2 | Ram Asesh | 9 | 9 | 81 | 10 (73) | 82 |  |
| 9 | 2 | Ram Asesh | 13 | 14 | 81 | 14 | 82 | Sent to KTM |
| 9 | 2 | Ram Asesh | 9 | 9 | 81 | 9 (72) | 82 |  |
| 9 | 2 | Ram Asesh | 12 | 12 | 81 | 11 (--) | 82 |  |
| 9 | 2 | Ram Asesh | 3 | 3 | 81 | 7 (82) | 82 | can't do 04 and 06 |
| 9 | 2 | Ram Asesh | 9 | 10 | 81 | 10 (76) | 82 |  |
| 9 | 2 | Ram Asesh | 9 | 9 | 81 | 10 (76) | 82 |  |
| 9 | 2 | Ram Asesh | 12 | 12 | 81 | 10 (--) | 82 |  |
| 9 | 2 | Ram Asesh | 12 | 13 | 81 | 13 (82) | 82 | 14 completed in week 80 |
| 9 | 2 | Ram Asesh | 9 | 9 | 81 | 8 (82) | 82 |  |
|  |  |  |  |  |  |  |  |  |
| 9 | 3 | Ram NR | 3 | 3 | 81 | 3 (73) | 82 |  |
| 9 | 3 | Ram NR | 11 | 11 | 81 | 12 (77) | 82 |  |
| 9 | 3 | Ram NR | 5 | 5 | 81 | 4 (81) | 82 |  |
| 9 | 3 | Ram NR | 9 | 9 | 81 | 9 (72) | 82 |  |
| 9 | 3 | Ram NR | 10 | 10 | 81 | 10 (76) | 82 |  |
| 9 | 3 | Ram NR | 10 | 10 | 81 | 10 (76) | 82 |  |
| 9 | 3 | Ram NR | 9 | 9 | 81 | 9 (73) | 82 |  |
| 9 | 3 | Ram NR | 1 | 1 | 81 | 3 (74) | 82 |  |
| 9 | 3 | Ram NR | 9 | 9 | 81 | 9 (73) | 82 |  |
| 9 | 3 | Ram NR | 9 | 9 | 81 | 9 (73) | 82 |  |
| 9 | 3 |  |  |  |  |  |  |  |
|  |  |  |  |  |  |  |  |  |
| 9 | 4 | Ram NR | 11 | 11 | 81 | 12 (77) | 82 |  |
| 9 | 4 | Ram NR | 3 | 3 | 81 | 6 (79) | 82 |  |
| 9 | 4 | Ram NR | 9 | 9 | 81 | 7 (78) | 82 |  |
| 9 | 4 | Ram NR | 8 | 8 | 81 | 8 (76) | 82 |  |
| 9 | 4 | Ram NR | 9 | 9 | 81 | 9 (77) | 82 |  |
| 9 | 4 | Ram NR | 12 | 12 | 81 | 14 | 82 | Sent to KTM |
| 9 | 4 | Ram NR | 12 | 12 | 81 | 12 (--) | 82 |  |
| 9 | 4 | Ram NR | 5 | 5 | 81 | 5 (82) | 82 |  |
| 9 | 4 | Ram NR | 12 | 12 | 81 | 13 (82) | 82 |  |
| 9 | 4 | Ram NR | 12 | 12 | 81 | 12 (70) | 82 |  |

| Motor Milestones Validation Study October 2002 | | | | | | | |
| --- | --- | --- | --- | --- | --- | --- | --- |
| W | S | TLI Name | MM# TLI Saw | MM# Mom Told TLI | NNIPS Week | MM# Mom Told WD | NNIPS Wk # Checked |
| 1 | 1 | Dipak | 12 | 13 | 95 | 12 | 97 |
| 1 | 1 | Dipak | 12 | 13 | 95 | 14 | 97 |
| 1 | 1 | Dipak | 14 | 13 | 95 | 14 | 97 |
| 1 | 1 | Dipak | 13 | 12 | 95 | 14 | 97 |
| 1 | 1 | Dipak | 11 | 12 | 95 | 14 | 97 |
| 1 | 1 | Dipak | 13 | 14 | 95 | 14 | 97 |
| 1 | 1 | Dipak | 12 | 13 | 95 | 14 | 97 |
| 1 | 1 | Dipak | 12 | 12 | 95 | 14 | 97 |
| 1 | 1 | Dipak | 13 | 13 | 95 | 14 | 97 |
| 1 | 1 | Dipak | 12 | 12 | 95 | 13 (79) | 97 |
|  |  |  |  |  |  |  |  |
| 1 | 2 | Dipak | 13 | 12 | 95 | 14 | 97 |
| 1 | 2 | Dipak | 12 | 12 | 95 | 14 | 97 |
| 1 | 2 | Dipak | 11 | 12 | 95 | 14 | 97 |
| 1 | 2 | Dipak | 12 | 13 | 95 | 14 | 97 |
| 1 | 2 | Dipak | 11 | 12 | 95 | 14 | 97 |
| 1 | 2 | Dipak | 12 | 13 | 95 | 14 | 97 |
| 1 | 2 | Dipak | 12 | 13 | 95 | 14 | 97 |
| 1 | 2 | Dipak | 11 | 11 | 95 | 11 (76) | 97 |
| 1 | 2 | Dipak | 14 | 14 | 95 | 14 | 97 |
| 1 | 2 | Dipak | 13 | 14 | 95 | 14 | 97 |
| 1 | 2 | Dipak | 11 | 12 | 95 | 14 | 97 |
|  |  |  |  |  |  |  |  |
| 1 | 3 | Nir BDR | 12 | 12 | 95 | 12 (77) | 97 |
| 1 | 3 | Nir BDR | 13 | 14 | 95 | 14 | 97 |
| 1 | 3 | Nir BDR | 12 | 12 | 95 | 12 (55) | 97 |
| 1 | 3 | Nir BDR | 14 | 14 | 95 | 14 | 97 |
| 1 | 3 | Nir BDR | 14 | 14 | 95 | 14 | 97 |
| 1 | 3 | Nir BDR | 14 | 13 | 95 | 12 (54) | 97 |
| 1 | 3 | Nir BDR | 13 | 14 | 95 | 14 | 97 |
| 1 | 3 | Nir BDR | 14 | 14 | 95 | 14 | 97 |
| 1 | 3 | Nir BDR | 14 | 14 | 95 | 14 | 97 |
| 1 | 3 | Nir BDR | 13 | 13 | 95 | 14 | 97 |
| 1 | 3 | Nir BDR | 14 | 14 | 95 | 14 | 97 |
|  |  |  |  |  |  |  |  |
| 1 | 4 | Nir BDR | 14 | 13 | 95 | 14 | 97 |
| 1 | 4 | Nir BDR | 14 | 13 | 95 | 14 | 97 |
| 1 | 4 | Nir BDR | 14 | 14 | 95 | 14 | 97 |
| 1 | 4 | Nir BDR | 14 | 13 | 95 | 14 | 97 |
| 1 | 4 | Nir BDR | 14 | 14 | 95 | 14 | 97 |
| 1 | 4 | Nir BDR | 13 | 13 | 95 | 14 | 97 |
| 1 | 4 | Nir BDR | 14 | 13 | 95 | 14 | 97 |
| 1 | 4 | Nir BDR | 14 | 14 | 95 | 14 | 97 |
| 1 | 4 | Nir BDR | 14 | 14 | 95 | 14 | 97 |
| 1 | 4 | Nir BDR | 14 | 14 | 95 | 14 | 97 |
|  |  |  |  |  |  |  |  |
| 2 | 1 | Kesab | 14 | 14 | 95 | 14 | 97 |
| 2 | 1 | Kesab | 12 | 13 | 95 | 14 | 97 |
| 2 | 1 | Kesab | 13 | 14 | 95 | 14 | 97 |
| 2 | 1 | Kesab | 14 | 14 | 95 | 14 | 97 |
| 2 | 1 | Kesab | 12 | 13 | 95 | 14 | 97 |
| 2 | 1 | Kesab | 13 | 14 | 95 | 14 | 97 |
| 2 | 1 | Kesab | 12 | 13 | 95 | 13 (87) | 97 |
| 2 | 1 | Kesab | 14 | 14 | 95 | 14 | 97 |
| 2 | 1 | Kesab | 14 | 14 | 95 | 14 | 97 |
| 2 | 1 | Kesab | 12 | 14 | 95 | 14 | 97 |
|  |  |  |  |  |  |  |  |
| 2 | 2 | Kesab | 12 | 13 | 95 | 14 | 97 |
| 2 | 2 | Kesab | 12 | 13 | 95 | 14 | 97 |
| 2 | 2 | Kesab | 14 | 12 | 95 | 14 | 97 |
| 2 | 2 | Kesab | 12 | 13 | 95 | 14 | 97 |
| 2 | 2 | Kesab | 13 | 13 | 95 | 14 | 97 |
| 2 | 2 | Kesab | 12 | 12 | 95 | 14 | 97 |
| 2 | 2 | Kesab | 14 | 14 | 95 | 14 | 97 |
| 2 | 2 | Kesab | 12 | 14 | 95 | 14 | 97 |
| 2 | 2 | Kesab | 12 | 12 | 95 | 14 | 97 |
| 2 | 2 | Kesab | 14 | 14 | 95 | 14 | 97 |
|  |  |  |  |  |  |  |  |
| 2 | 3A | Kesab | 12 | 12 | 95 | 12 (83) | 97 |
| 2 | 3A | Kesab | 14 | 13 | 95 | 14 | 97 |
| 2 | 3A | Kesab | 12 | 12 | 95 | 14 | 97 |
| 2 | 3A | Kesab | 14 | 14 | 95 | 14 | 97 |
| 2 | 3A | Kesab | 11 | 12 | 95 | 14 | 97 |
| 2 | 3A | Kesab | 14 | 14 | 95 | 14 | 97 |
| 2 | 3A | Kesab | 14 | 14 | 95 | 14 | 97 |
| 2 | 3A | Kesab | 14 | 14 | 95 | 14 | 97 |
| 2 | 3A | Kesab | 14 | 14 | 95 | 14 | 97 |
| 2 | 3A | Kesab | 11 | 12 | 95 | 14 (96) | 97 |
|  |  |  |  |  |  |  |  |
| 2 | 3B | Kesab | 11 | 11 | 95 | 14 | 97 |
| 2 | 3B | Kesab | 12 | 13 | 95 | 14 | 97 |
| 2 | 3B | Kesab | 11 | 11 | 95 | 11 (85) | 97 |
| 2 | 3B | Kesab | 11 | 12 | 95 | 13 (90) | 97 |
| 2 | 3B | Kesab | 13 | 13 | 95 | 14 | 97 |
| 2 | 3B | Kesab | 12 | 13 | 95 | 14 | 97 |
| 2 | 3B | Kesab | 11 | 12 | 95 | 11 (57) | 97 |
| 2 | 3B | Kesab | 12 | 12 | 95 | 14 | 97 |
| 2 | 3B | Kesab | 12 | 13 | 95 | 14 | 97 |
| 2 | 3B | Kesab | 11 | 12 | 95 | 12 (62) | 97 |
|  |  |  |  |  |  |  |  |
| 3 | 1 | Nir BDR | 14 | 13 | 95 | 14 | 97 |
| 3 | 1 | Nir BDR | 14 | 13 | 95 | 14 | 97 |
| 3 | 1 | Nir BDR | 14 | 14 | 95 | 14 | 97 |
| 3 | 1 | Nir BDR | 14 | 14 | 95 | 14 | 97 |
| 3 | 1 | Nir BDR | 14 | 14 | 95 | 14 | 97 |
| 3 | 1 | Nir BDR | 9 | 9 | 95 |  | 97 |
| 3 | 1 | Nir BDR | 14 | 14 | 95 | 14 | 97 |
| 3 | 1 | Nir BDR | 13 | 13 | 95 | 14 | 97 |
| 3 | 1 | Nir BDR | 14 | 14 | 95 | 12 (92) | 97 |
| 3 | 1 | Nir BDR | 14 | 14 | 95 | 14 | 97 |
| 3 | 1 | Nir BDR | 14 | 14 | 95 | 14 | 97 |
|  |  |  |  |  |  |  |  |
| 3 | 2 | Nir BDR | 14 | 14 | 95 | 14 | 97 |
| 3 | 2 | Nir BDR | 14 | 14 | 95 | 14 | 97 |
| 3 | 2 | Nir BDR | 14 | 14 | 95 | 14 | 97 |
| 3 | 2 | Nir BDR | 14 | 14 | 95 | 14 | 97 |
| 3 | 2 | Nir BDR | 14 | 14 | 95 | 13 (-) | 97 |
| 3 | 2 | Nir BDR | 12 | 12 | 95 | 13 (56) | 97 |
| 3 | 2 | Nir BDR | 12 | 12 | 95 | 14 | 97 |
| 3 | 2 | Nir BDR | 12 | 12 | 95 | 13 (94) | 97 |
| 3 | 2 | Nir BDR | 14 | 13 | 95 | 14 | 97 |
| 3 | 2 | Nir BDR | 12 | 13 | 95 | 14 (93) | 97 |
|  |  |  |  |  |  |  |  |
| 4 | 1 | Kesab | 12 | 14 | 95 | 14 | 97 |
| 4 | 1 | Kesab | 14 | 14 | 95 | 14 | 97 |
| 4 | 1 | Kesab | 14 | 13 | 95 | 14 | 97 |
| 4 | 1 | Kesab | 14 | 13 | 95 | 14 | 97 |
| 4 | 1 | Kesab | 14 | 14 | 95 | 14 | 97 |
| 4 | 1 | Kesab | 14 | 14 | 95 | 14 | 97 |
| 4 | 1 | Kesab | 12 | 14 | 95 | 14 | 97 |
| 4 | 1 | Kesab | 14 | 14 | 95 | 14 | 97 |
| 4 | 1 | Kesab | 14 | 14 | 95 | 14 | 97 |
| 4 | 1 | Kesab | 14 | 14 | 95 | 14 | 97 |
|  |  |  |  |  |  |  |  |
| 4 | 2 | Kesab | 14 | 14 | 95 | 14 | 97 |
| 4 | 2 | Kesab | 14 | 13 | 95 | 14 | 97 |
| 4 | 2 | Kesab | 14 | 14 | 95 | 14 | 97 |
| 4 | 2 | Kesab | 14 | 14 | 95 | 14 | 97 |
| 4 | 2 | Kesab | 12 | 13 | 95 | 12 (63) | 97 |
| 4 | 2 | Kesab | 11 | 12 | 95 | 12 (63) | 97 |
| 4 | 2 | Kesab | 12 | 14 | 95 | 12 (64) | 97 |
| 4 | 2 | Kesab | 14 | 14 | 95 | 14 | 97 |
| 4 | 2 | Kesab | 14 | 13 | 95 | 14 | 97 |
| 4 | 2 | Kesab | 13 | 14 | 95 | 14 | 97 |
|  |  |  |  |  |  |  |  |
| 5 | 1 | Ram NR | 12 | 14 | 96 | 14 | 97 |
| 5 | 1 | Ram NR | 12 | 14 | 96 | 14 | 97 |
| 5 | 1 | Ram NR | 12 | 12 | 96 | 13 (51) | 97 |
| 5 | 1 | Ram NR | 12 | 13 | 96 | 14 | 97 |
| 5 | 1 | Ram NR | 12 | 12 | 96 | 14 | 97 |
| 5 | 1 | Ram NR | 12 | 13 | 96 | 14 | 97 |
| 5 | 1 | Ram NR | 12 | 13 | 96 | 14 | 97 |
| 5 | 1 | Ram NR | 12 | 13 | 96 | 14 (97) | 97 |
| 5 | 1 | Ram NR | 12 | 14 | 96 | 14 | 97 |
| 5 | 1 | Ram NR | 14 | 13 | 96 | 14 | 97 |
|  |  |  |  |  |  |  |  |
| 6 | 1 | Kesab | 12 | 12 | 96 | 14 | 97 |
| 6 | 1 | Kesab | 14 | 13 | 96 | 14 | 97 |
| 6 | 1 | Kesab | 14 | 12 | 96 | 14 | 97 |
| 6 | 1 | Kesab | 14 | 14 | 96 | 14 | 97 |
| 6 | 1 | Kesab | 14 | 14 | 96 | 14 | 97 |
| 6 | 1 | Kesab | 14 | 14 | 96 | 14 | 97 |
| 6 | 1 | Kesab | 12 | 14 | 96 | 14 | 97 |
| 6 | 1 | Kesab | 12 | 14 | 96 | 14 (93) | 97 |
| 6 | 1 | Kesab | 14 | 14 | 96 | 14 | 97 |
| 6 | 1 | Kesab | 14 | 14 | 96 | 14 | 97 |
|  |  |  |  |  |  |  |  |
| 6 | 2 | Ram NR | 11 | 11 | 95 | 11 (90) | 97 |
| 6 | 2 | Ram NR | 14 | 14 | 95 | 14 | 97 |
| 6 | 2 | Ram NR | 14 | 14 | 95 | 14 | 97 |
| 6 | 2 | Ram NR | 11 | 12 | 95 | 12 (64) | 97 |
| 6 | 2 | Ram NR | 14 | 14 | 95 | 14 | 97 |
| 6 | 2 | Ram NR | 12 | 12 | 95 | 14 | 97 |
| 6 | 2 | Ram NR | 14 | 14 | 95 | 14 | 97 |
| 6 | 2 | Ram NR | 12 | 12 | 95 | 14 | 97 |
| 6 | 2 | Ram NR | 12 | 12 | 95 | 13 (81) | 97 |
| 6 | 2 | Ram NR | 11 | 12 | 95 | 12 (59) | 97 |
|  |  |  |  |  |  |  |  |
| 6 | 3 | Ram NR | 14 | 14 | 95 | 14 | 97 |
| 6 | 3 | Ram NR | 12 | 14 | 95 | 14 | 97 |
| 6 | 3 | Ram NR | 9 | 9 | 95 | 14 | 97 |
| 6 | 3 | Ram NR | 14 | 14 | 95 | 14 | 97 |
| 6 | 3 | Ram NR | 11 | 12 | 95 | 14 | 97 |
| 6 | 3 | Ram NR | 12 | 14 | 95 | 14 | 97 |
| 6 | 3 | Ram NR | 12 | 12 | 95 | 14 | 97 |
| 6 | 3 | Ram NR | 11 | 12 | 95 | 14 | 97 |
| 6 | 3 | Ram NR | 12 | 13 | 95 | 14 | 97 |
| 6 | 3 | Ram NR | 14 | 14 | 95 | 14 | 97 |
|  |  |  |  |  |  |  |  |
| 7 | 1 | Ram NR | 11 | 12 | 95 | 14 | 97 |
| 7 | 1 | Ram NR | 11 | 12 | 95 | 14 | 97 |
| 7 | 1 | Ram NR | 12 | 13 | 95 | 14 | 97 |
| 7 | 1 | Ram NR | 12 | 13 | 95 | 14 | 97 |
| 7 | 1 | Ram NR | 12 | 13 | 95 | 14 | 97 |
| 7 | 1 | Ram NR | 11 | 12 | 95 | 14 | 97 |
| 7 | 1 | Ram NR | 12 | 14 | 95 | 14 | 97 |
| 7 | 1 | Ram NR | 12 | 13 | 95 | 14 | 97 |
| 7 | 1 | Ram NR | 10 | 10 | 95 | 10 (73) | 97 |
| 7 | 1 | Ram NR | 12 | 13 | 95 | 14 | 97 |
|  |  |  |  |  |  |  |  |
| 8 | 1 | Ram NR | 11 | 12 | 95 | 14 | 97 |
| 8 | 1 | Ram NR | 14 | 13 | 95 | 14 | 97 |
| 8 | 1 | Ram NR | 10 | 10 | 95 | 14 | 97 |
| 8 | 1 | Ram NR | 11 | 12 | 95 | 14 | 97 |
| 8 | 1 | Ram NR | 10 | 11 | 95 | 14 | 97 |
| 8 | 1 | Ram NR | 12 | 13 | 95 | 14 | 97 |
| 8 | 1 | Ram NR | 11 | 13 | 95 | 14 | 97 |
| 8 | 1 | Ram NR | 14 | 14 | 95 | 14 | 97 |
| 8 | 1 | Ram NR | 12 | 12 | 95 | 14 | 97 |
| 8 | 1 | Ram NR | 12 | 13 | 95 | 14 | 97 |
|  |  |  |  |  |  |  |  |
|  |  |  |  |  |  |  |  |
| 8 | 2 | Ram NR | 11 | 12 | 95 | 14 | 97 |
| 8 | 2 | Ram NR | 11 | 12 | 95 | 12 (85) | 97 |
| 8 | 2 | Ram NR | 9 | 9 | 95 | 9 (86) | 97 |
| 8 | 2 | Ram NR | 14 | 14 | 95 | 14 | 97 |
| 8 | 2 | Ram NR | 12 | 14 | 95 | 14 | 97 |
| 8 | 2 | Ram NR | 12 | 14 | 95 | 14 | 97 |
| 8 | 2 | Ram NR | 11 | 12 | 95 | 14 | 97 |
| 8 | 2 | Ram NR | 14 | 14 | 95 | 14 | 97 |
| 8 | 2 | Ram NR | 11 | 12 | 95 | 12 (86) | 97 |
| 8 | 2 | Ram NR | 11 | 12 | 95 | 12 (-) | 97 |
|  |  |  |  |  |  |  |  |
|  |  |  |  |  |  |  |  |
| 9 | 1 | Kesab | 12 | 14 | 95 | 14 (91) | 97 |
| 9 | 1 | Kesab | 12 | 14 | 95 | 14 | 97 |
| 9 | 1 | Kesab | 14 | 14 | 95 | 14 | 97 |
| 9 | 1 | Kesab | 14 | 14 | 95 | 14 | 97 |
| 9 | 1 | Kesab | 14 | 14 | 95 | 14 | 97 |
| 9 | 1 | Kesab | 14 | 14 | 95 | 14 | 97 |
| 9 | 1 | Kesab | 14 | 14 | 95 | 14 | 97 |
| 9 | 1 | Kesab | 12 | 14 | 95 | 14 | 97 |
| 9 | 1 | Kesab | 12 | 14 | 95 | 14 | 97 |
| 9 | 1 | Kesab | 14 | 13 | 95 | 14 | 97 |
|  |  |  |  |  |  |  |  |
|  |  |  |  |  |  |  |  |
| 9 | 2 | Kesab | 14 | 14 | 95 | 14 | 97 |
| 9 | 2 | Kesab | 14 | 14 | 95 | 14 | 97 |
| 9 | 2 | Kesab | 14 | 14 | 95 | 14 | 97 |
| 9 | 2 | Kesab | 14 | 14 | 95 | 14 | 97 |
| 9 | 2 | Kesab | 14 | 14 | 95 | 14 | 97 |
| 9 | 2 | Kesab | 14 | 14 | 95 | 14 | 97 |
| 9 | 2 | Kesab | 12 | 14 | 95 | 14 | 97 |
| 9 | 2 | Kesab | 14 | 14 | 95 | 14 | 97 |
| 9 | 2 | Kesab | 11 | 12 | 95 | 14 | 95 |
| 9 | 2 | Kesab | 14 | 14 | 95 | 14 | 97 |
|  |  |  |  |  |  |  |  |
|  |  |  |  |  |  |  |  |
| 9 | 3 | Nir BDR | 14 | 14 | 95 | 14 | 97 |
| 9 | 3 | Nir BDR | 14 | 14 | 95 | 14 | 97 |
| 9 | 3 | Nir BDR | 14 | 14 | 95 | 14 | 97 |
| 9 | 3 | Nir BDR | 14 | 14 | 95 | 14 | 97 |
| 9 | 3 | Nir BDR | 14 | 14 | 95 | 14 | 97 |
| 9 | 3 | Nir BDR | 12 | 12 | 95 | 14 | 97 |
| 9 | 3 | Nir BDR | 14 | 14 | 95 | 14 | 97 |
| 9 | 3 | Nir BDR | 14 | 14 | 95 | 14 | 97 |
| 9 | 3 | Nir BDR | 14 | 14 | 95 | 14 | 97 |
| 9 | 3 | Nir BDR | 12 | 12 | 95 | 14 | 97 |
|  |  | Nir BDR | 14 | 14 | 95 | 14 | 97 |
|  |  |  |  |  |  |  |  |
| 9 | 4 | Nir BDR | 14 | 14 | 95 | 14 | 97 |
| 9 | 4 | Nir BDR | 14 | 14 | 95 | 14 | 97 |
| 9 | 4 | Nir BDR | 14 | 14 | 95 | 14 | 97 |
| 9 | 4 | Nir BDR | 14 | 13 | 95 | 14 | 97 |
| 9 | 4 | Nir BDR | 14 | 14 | 95 | 14 | 97 |
| 9 | 4 | Nir BDR | 14 | 14 | 95 | 14 | 97 |
| 9 | 4 | Nir BDR | 14 | 13 | 95 | 14 | 97 |
| 9 | 4 | Nir BDR | 14 | 14 | 95 | 14 | 97 |
| 9 | 4 | Nir BDR | 14 | 14 | 95 | 13 (95) | 97 |
| 9 | 4 | Nir BDR | 14 | 14 | 95 | 14 | 97 |
| 9 | 4 | Nir BDR | 14 | 14 | 95 | 14 | 97 |
| 9 | 4 | Nir BDR | 14 | 14 | 95 | 14 | 97 |
|  |  |  |  |  |  |  |  |

| Motor Milestones Validation Study January 2003 | | | | | | | | |  |
| --- | --- | --- | --- | --- | --- | --- | --- | --- | --- |
| W | S | TLI Name | MM# TLI Saw | MM# Mom Told TLI | NNIPS Week | MM# Mom Told WD | NNIPS Wk # Checked | Comments |  |
| 1 | 1 | Rudra | 11 | 11 | 110 | 12 (98) | 110 |  |  |
| 1 | 1 | Rudra | 11 | 11 | 110 | 12 (90) | 110 |  |  |
| 1 | 1 | Rudra | 10 | 10 | 110 | 10 (90) | 110 |  |  |
| 1 | 1 | Rudra | 11 | 11 | 110 | 12 (104) | 110 |  |  |
| 1 | 1 | Rudra | 9 | 9 | 110 | 8 (79) | 110 |  |  |
| 1 | 1 | Rudra | 11 | 11 | 110 | 11 (102) | 110 |  |  |
| 1 | 1 | Rudra | 11 | 11 | 110 | 12 (106) | 110 |  |  |
| 1 | 1 | Rudra | 11 | 11 | 110 | 11 (110) | 110 |  |  |
| 1 | 1 | Rudra | 9 | 9 | 110 | 10 (110) | 110 |  |  |
| 1 | 1 | Rudra | 9 | 9 | 110 | 8 (102) | 110 |  |  |
|  |  |  |  |  |  |  |  |  |  |
| 1 | 2 | Rudra | 7 | 7 | 110 | 9 (100) | 110 | WD says saw | ***** |
| 1 | 2 | Rudra | 9 | 9 | 110 | 9 (98) | 110 |  |  |
| 1 | 2 | Rudra | 4 | 6 | 110 | 5 (110) | 110 |  |  |
| 1 | 2 | Rudra | 11 | 11 | 110 | 12 (98) | 110 |  |  |
| 1 | 2 | Rudra | 11 | 11 | 110 | 12 (99) | 110 |  |  |
| 1 | 2 | Rudra | 11 | 11 | 110 | 11 (99) | 110 |  |  |
| 1 | 2 | Rudra | 11 | 11 | 110 | 12 (110) | 110 |  |  |
| 1 | 2 | Rudra | 11 | 11 | 110 | 12 (110) | 110 |  |  |
| 1 | 2 | Rudra | 11 | 11 | 110 | 12 (100) | 110 |  |  |
| 1 | 2 | Rudra | 11 | 11 | 110 | 12 (110) | 110 |  |  |
|  |  |  |  |  |  |  |  |  |  |
| 1 | 3 | Kesab | 11 | 12 | 110 | 11 (104) | 110 |  |  |
| 1 | 3 | Kesab | 12 | 14 | 110 | 11 (57) | 110 |  | * |
| 1 | 3 | Kesab | 11 | 12 | 110 | 12 (55) | 110 |  |  |
| 1 | 3 | Kesab | 11 | 12 | 110 | 12 (110) | 110 |  |  |
| 1 | 3 | Kesab | 12 | 13 | 110 | 12 (55) | 110 |  |  |
| 1 | 3 | Kesab | 11 | 11 | 110 | 11 (108) | 110 |  |  |
| 1 | 3 | Kesab | 10 | 10 | 110 | 10 (108) | 110 |  |  |
| 1 | 3 | Kesab | 12 | 12 | 110 | 12 (102) | 110 |  |  |
| 1 | 3 | Kesab | 10 | 11 | 110 | 11 (108) | 110 |  |  |
| 1 | 3 | Kesab | 9 | 8 | 110 | 8 (103) | 110 |  |  |
|  |  |  |  |  |  |  |  |  |  |
| 1 | 4 | Kesab | 9 | 9 | 110 | 7(90) | 110 |  | * |
| 1 | 4 | Kesab | 11 | 11 | 110 | 12 (105) | 110 |  |  |
| 1 | 4 | Kesab | 9 | 9 | 110 | 9 (103) | 110 |  |  |
| 1 | 4 | Kesab | 10 | 10 | 110 | 10 (103) | 110 |  |  |
| 1 | 4 | Kesab | 9 | 9 | 110 | 9 (97) | 110 |  |  |
| 1 | 4 | Kesab | 6 | 6 | 110 | 6 (109) | 110 |  |  |
| 1 | 4 | Kesab | 2 | 2 | 110 | 1 (102) | 110 |  |  |
| 1 | 4 | Kesab | 11 | 11 | 110 | 12 (107) | 110 |  |  |
| 1 | 4 | Kesab | 6 | 6 | 110 | 8 (107) | 110 |  | * |
| 1 | 4 | Roshan | 13 | 13 | 110 | 14 (-) | 110 |  |  |
|  |  |  |  |  |  |  |  |  |  |
| 2 | 1 | Ram NR | 11 | 11 | 110 | 12 (110) | 110 |  |  |
| 2 | 1 | Ram NR | 11 | 11 | 110 | 11 (93) | 110 |  |  |
| 2 | 1 | Ram NR | 12 | 12 | 110 | 12 (71) | 110 |  |  |
| 2 | 1 | Ram NR | 10 | 10 | 110 | 10 (104) | 110 |  |  |
| 2 | 1 | Ram NR | 10 | 10 | 110 | 10 (55) | 110 |  |  |
| 2 | 1 | Ram NR | 12 | 13 | 110 | 12 (65) | 110 |  |  |
| 2 | 1 | Ram NR | 11 | 11 | 110 | 11 (81) | 110 |  |  |
| 2 | 1 | Ram NR | 10 | 10 | 110 | 10 (110) | 110 |  |  |
| 2 | 1 | Ram NR | 11 | 11 | 110 | 9 (89) | 110 | WD says can't do;sick | * |
| 2 | 1 | Ram NR | 12 | 12 | 110 | 12 (76) | 110 |  |  |
|  |  |  |  |  |  |  |  |  |  |
| 2 | 2 | Dipak | 12 | 12 | 110 | 12 (109) | 110 |  |  |
| 2 | 2 | Dipak | 12 | 13 | 110 | 12 (109) | 110 |  |  |
| 2 | 2 | Kesab | 11 | 11 | 111 | 12 (109) | 111 |  |  |
| 2 | 2 | Kesab | 1 | 1 | 111 | 1 (107) | 111 |  |  |
| 2 | 2 | Kesab | 11 | 11 | 111 | 11 (105) | 111 |  |  |
| 2 | 2 | Kesab | 11 | 12 | 111 | 12 (107) | 111 |  |  |
| 2 | 2 | Kiran | 11 | 11 | 111 | 11 (98) | 111 |  |  |
| 2 | 2 | Kesab | 11 | 11 | 111 | 12 (109) | 111 |  |  |
| 2 | 2 | Kiran | 10 | 10 | 111 | 10 (105) | 111 |  |  |
| 2 | 2 | Kesab | 12 | 12 | 111 | 14 (109) | 111 | 13 blank | * |
|  |  |  |  |  |  |  |  |  |  |
| 2 | 3A | Ram NR | 11 | 11 | 110 | 10 (101) | 110 |  |  |
| 2 | 3A | Ram NR | 9 | 9 | 110 | 10 (95) | 110 |  |  |
| 2 | 3A | Ram NR | 9 | 8 | 110 | 9 (56) | 110 |  |  |
| 2 | 3A | Ram NR | 9 | 10 | 110 | 10 (106) | 110 |  |  |
| 2 | 3A | Ram NR | 10 | 10 | 110 | 10 (110) | 110 |  |  |
| 2 | 3A | Ram NR | 12 | 12 | 110 | 14 (-) | 110 |  | * |
| 2 | 3A | Ram NR | 12 | 12 | 110 | 14 (-) | 110 |  | * |
| 2 | 3A | Ram NR | 12 | 12 | 110 | 12 (92) | 110 |  |  |
| 2 | 3A | Ram NR | 12 | 12 | 110 | 12 (59) | 110 |  |  |
| 2 | 3A | Ram NR | 12 | 12 | 110 | 12 (82) | 110 |  |  |
|  |  |  |  |  |  |  |  |  |  |
| 2 | 3B | Dipak | 12 | 12 | 110 | 12 (60) | 110 |  |  |
| 2 | 3B | Dipak | 12 | 12 | 110 | 9 (101) | 110 |  | * |
| 2 | 3B | Dipak | 12 | 12 | 110 | 12 (88) | 110 |  |  |
| 2 | 3B | Sulochana | 11 | 11 | 110 | 11 (105) | 110 |  |  |
| 2 | 3B | Sulochana | 11 | 11 | 110 | 12 (110) | 110 |  |  |
| 2 | 3B | Sulochana | 12 | 12 | 110 | 12 (92) | 110 |  |  |
| 2 | 3B | Sulochana | 11 | 11 | 110 | 11 (103) | 110 |  |  |
| 2 | 3B | Sulochana | 11 | 11 | 110 | 12 (98) | 110 |  |  |
| 2 | 3B | Sulochana | 11 | 12 | 110 | 12 (108) | 110 |  |  |
| 2 | 3B | Sulochana | 10 | 10 | 110 | 11 (110) | 110 |  |  |
|  |  |  |  |  |  |  |  |  |  |
| 3 | 1 | Nir BDR | 9 | 9 | 110 | 11 (73) | 110 |  | * |
| 3 | 1 | Nir BDR | 10 | 11 | 110 | 11 (101) | 110 |  |  |
| 3 | 1 | Nir BDR | 12 | 12 | 110 | 12 (106) | 110 |  |  |
| 3 | 1 | Nir BDR | 1 | 1 | 110 | 1 (98) | 110 |  |  |
| 3 | 1 | Nir BDR | 12 | 12 | 110 | 12 (89) | 110 |  |  |
| 3 | 1 | Nir BDR | 11 | 10 | 110 | 9 (101) | 110 |  |  |
| 3 | 1 | Nir BDR | 11 | 12 | 110 | 12 (106) | 110 |  |  |
| 3 | 1 | Nir BDR | 9 | 10 | 110 | 9 (109) | 110 |  |  |
| 3 | 1 | Nir BDR | 9 | 10 | 110 | 11 (102) | 110 |  |  |
| 3 | 1 | Kesab | 12 | 13 | 110 |  |  |  |  |
|  |  |  |  |  |  |  |  |  |  |
| 3 | 2 | Kiran | 12 | 12 | 111 | 12 (90) | 111 |  |  |
| 3 | 2 | Kiran | 11 | 12 | 111 | 12 (101) | 111 |  |  |
| 3 | 2 | Kiran | 12 | 12 | 111 | 12 (99) | 111 |  |  |
| 3 | 2 | Kiran | 3 | 3 | 111 | 1 (108) | 111 |  | * |
| 3 | 2 | Kiran | 9 | 9 | 111 | 8 (101) | 111 |  |  |
| 3 | 2 | Kesab | 11 | 11 | 111 | 11 (102) | 111 |  |  |
| 3 | 2 | Nir BDR | 11 | 11 | 110 | 9 (97) | 110 |  | * |
| 3 | 2 | Nir BDR | 8 | 8 | 110 | 9 (102) | 110 |  |  |
| 3 | 2 | Nir BDR | 12 | 12 | 110 | 12 (81) | 110 |  |  |
| 3 | 2 | Nir BDR | 11 | 11 | 110 | 11 (107) | 110 |  |  |
|  |  |  |  |  |  |  |  |  |  |
| 4 | 1 | Kesab | 11 | 11 | 110 | 11 (104) | 110 |  |  |
| 4 | 1 | Kesab | 12 | 12 | 110 | 12 (94) | 110 |  |  |
| 4 | 1 | Kesab | 11 | 13 | 110 | 12 (104) | 110 |  |  |
| 4 | 1 | Kesab | 3 | 6 | 110 | 3 (110) | 110 |  | * |
| 4 | 1 | Kesab | 11 | 11 | 110 | 11 (104) | 110 |  |  |
| 4 | 1 | Kesab | 9 | 9 | 110 | 9 (97) | 110 |  |  |
| 4 | 1 | Nir BDR | 12 | 12 | 110 | 13 (101) | 110 |  |  |
| 4 | 1 | Nir BDR | 14 | 13 | 110 | 14 (-) | 110 |  |  |
| 4 | 1 | Nir BDR | 14 | 13 | 110 | 14 (-) | 110 |  |  |
| 4 | 1 | Nir BDR | 3 | 3 | 110 | 6 (107) | 110 |  | * |
| 4 | 1 | Nir BDR | 14 | 13 | 110 | 14 (-) | 110 |  |  |
| 4 | 1 | Nir BDR | 14 | 13 | 110 | 14 (107) | 110 |  |  |
|  |  |  |  |  |  |  |  |  |  |
| 4 | 2 | Kesab | 12 | 12 | 110 | 12 (85) | 110 |  |  |
| 4 | 2 | Kesab | 12 | 13 | 110 | 12 (75) | 110 |  |  |
| 4 | 2 | Kesab | 12 | 12 | 110 | 12 (107) | 110 |  |  |
| 4 | 2 | Kesab | 11 | 11 | 110 | 11 (109) | 110 |  |  |
| 4 | 2 | Kesab | 9 | 9 | 110 | 9 (100) | 110 |  |  |
| 4 | 2 | Kesab | 12 | 12 | 110 | 12 (109) | 110 |  |  |
| 4 | 2 | Kesab | 9 | 9 | 110 | 9 (99) | 110 |  |  |
| 4 | 2 | Kesab | 10 | 10 | 110 | 10 (108) | 110 |  |  |
| 4 | 2 | Nir BDR | 13 | 13 | 110 | 14 (-) | 110 |  |  |
| 4 | 2 | Nir BDR | 13 | 13 | 110 | 14 (-) | 110 |  |  |
|  |  |  |  |  |  |  |  |  |  |
| 5 | 1 | Ram NR | 11 | 12 | 110 | 12 (92) | 110 |  |  |
| 5 | 1 | Ram NR | 11 | 12 | 110 | 12 (107) | 110 |  |  |
| 5 | 1 | Ram NR | 12 | 14 | 110 | 12 (85) | 110 |  | * |
| 5 | 1 | Ram NR | 12 | 12 | 110 | 12 (80) | 110 |  |  |
| 5 | 1 | Ram NR | 5 | 6 | 110 | 2 (104) | 110 | WD also saw 5 | * |
| 5 | 1 | Ram NR | 9 | 10 | 110 | 10 (110) | 110 |  |  |
| 5 | 1 | Ram NR | 4 | 4 | 110 | 9 (92) | 110 |  | * |
| 5 | 1 | Ram NR | 12 | 12 | 110 | 12 (92) | 110 |  |  |
| 5 | 1 | Ram NR | 6 | 6 | 110 | 6 (100) | 110 |  |  |
| 5 | 1 | Ram NR | 2 | 2 | 110 | 3 (103) | 110 |  |  |
|  |  |  |  |  |  |  |  |  |  |
| 6 | 1 | Kiran | 12 | 12 | 110 | 12 (106) | 110 |  |  |
| 6 | 1 | Kiran | 9 | 9 | 110 | 6 (105) | 110 |  | * |
| 6 | 1 | Kiran | 12 | 12 | 110 | 12 (102) | 110 |  |  |
| 6 | 1 | Kiran | 9 | 9 | 110 | 10 (108) | 110 |  |  |
| 6 | 1 | Kiran | 1 | 1 | 110 | 1 (108) | 110 |  |  |
| 6 | 1 | Kiran | 11 | 12 | 110 | 12 (96) | 110 |  |  |
| 6 | 1 | Kiran | 9 | 9 | 110 | 8 (106) | 110 |  |  |
| 6 | 1 | Kiran | 14 | 14 | 110 | 12 (92) | 110 |  | * |
| 6 | 1 | Kiran | 9 | 10 | 110 | 10 (103) | 110 |  |  |
| 6 | 1 | Kiran | 11 | 12 | 110 | 12 (106) | 110 |  |  |
|  |  |  |  |  |  |  |  |  |  |
| 6 | 2 | Ram NR | 12 | 12 | 110 | 12 (91) | 110 |  |  |
| 6 | 2 | Ram NR | 11 | 12 | 110 | 11 (98) | 110 |  |  |
| 6 | 2 | Ram NR | 12 | 12 | 110 | 12 (92) | 110 |  |  |
| 6 | 2 | Ram NR | 10 | 11 | 110 | 11 (96) | 110 |  |  |
| 6 | 2 | Ram NR | 1 | 1 | 110 | 1 (103) | 110 |  |  |
| 6 | 2 | Ram NR | 10 | 10 | 110 | 10 (79) | 110 |  |  |
| 6 | 2 | Ram NR | 11 | 11 | 110 | 11 (96) | 110 |  |  |
| 6 | 2 | Ram NR | 2 | 2 | 110 | 2 (110) | 110 |  |  |
| 6 | 2 | Ram NR | 9 | 9 | 110 | 9 (92) | 110 |  |  |
| 6 | 2 | Ram NR | 10 | 10 | 110 | 11 (109) | 110 |  |  |
|  |  |  |  |  |  |  |  |  |  |
| 6 | 3 | Shyam | 11 | 11 | 110 |  | 110 |  | * |
| 6 | 3 | Ram NR | 1 | 1 | 110 | 1 (94) | 110 |  |  |
| 6 | 3 | Ram NR | 1 | 1 | 110 | 1 (105) | 110 |  |  |
| 6 | 3 | Shyam | 12 | 13 | 110 | 14 (-) | 110 |  |  |
| 6 | 3 | Shyam | 13 | 13 | 110 | 14 (-) | 110 |  |  |
| 6 | 3 | Ram NR | 9 | 10 | 110 | 9 (96) | 110 |  |  |
| 6 | 3 | Ram NR | 9 | 10 | 110 | 10 (108) | 110 |  |  |
| 6 | 3 | Ram NR | 11 | 12 | 110 | 12 (83) | 110 |  |  |
| 6 | 3 | Ram NR | 2 | 2 | 110 | 2 (110) | 110 |  |  |
| 6 | 3 | Ram NR | 12 | 13 | 110 | 11 (91) | 110 |  | * |
|  |  |  |  |  |  |  |  |  |  |
| 7 | 1 | Kiran | 12 | 12 | 110 | 10 (103) | 110 |  | * |
| 7 | 1 | Kiran | 11 | 12 | 110 | 11 (100) | 110 |  |  |
| 7 | 1 | Kiran | 12 | 12 | 110 | 11 (106) | 110 |  |  |
| 7 | 1 | Kiran | 9 | 9 | 110 | 9 (89) | 110 |  |  |
| 7 | 1 | Kiran | 2 | 2 | 110 | 3 (102) | 110 |  |  |
| 7 | 1 | Kiran | 12 | 12 | 110 | 12 (105) | 110 |  |  |
| 7 | 1 | Kiran | 12 | 12 | 110 | 12 (76) | 110 |  |  |
| 7 | 1 | Kiran | 1 | 1 | 110 | 1 (107) | 110 |  |  |
| 7 | 1 | Kiran | 10 | 10 | 110 | 10 (109) | 110 |  |  |
| 7 | 1 | Kiran | 9 | 9 | 110 | 9 (93) | 110 |  |  |
|  |  |  |  |  |  |  |  |  |  |
| 8 | 1 | Kiran | 3 | 3 | 110 | 2 (101) | 110 |  |  |
| 8 | 1 | Kiran | 10 | 11 | 110 | 11 (102) | 110 |  |  |
| 8 | 1 | Kiran | 11 | 11 | 110 | 12 (108) | 110 |  |  |
| 8 | 1 | Kiran | 10 | 10 | 110 | 9 (80) | 110 |  |  |
| 8 | 1 | Kiran | 9 | 9 | 110 | 10 (103) | 110 |  |  |
| 8 | 1 | Kiran | 11 | 11 | 110 | 11 (107) | 110 |  |  |
| 8 | 1 | Ram NR | 12 | 13 | 110 | 14 (-) | 110 |  |  |
| 8 | 1 | Ram NR | 11 | 12 | 110 | 12 (64) | 110 |  |  |
| 8 | 1 | Kiran | 11 | 11 | 110 | 11 (108) | 110 |  |  |
| 8 | 1 | Kiran | 10 | 10 | 110 | 10 (108) | 110 |  |  |
|  |  |  |  |  |  |  |  |  |  |
| 8 | 2 | Kiran | 3 | 3 | 110 | 2 (96) | 110 |  |  |
| 8 | 2 | Kiran | 4 | 4 | 110 | 4 (97) | 110 |  |  |
| 8 | 2 | Kiran | 9 | 9 | 110 | 8 (110) | 110 |  |  |
| 8 | 2 | Kiran | 4 | 5 | 110 | 3 (102) | 110 |  | * |
| 8 | 2 | Kiran | 12 | 12 | 110 | 12 (96) | 110 |  |  |
| 8 | 2 | Kiran | 1 | 1 | 110 | 1 (104) | 110 |  |  |
| 8 | 2 | Kiran | 12 | 12 | 110 | 12 (79) | 110 |  |  |
| 8 | 2 | Ram NR | 12 | 13 | 110 | 12 (85) | 110 |  |  |
| 8 | 2 | Ram NR | 12 | 12 | 110 | 12 (86) | 110 |  |  |
| 8 | 2 | Ram NR | 12 | 12 | 110 | 13 (99) | 110 |  |  |
|  |  |  |  |  |  |  |  |  |  |
| 9 | 1 | Rudra | 12 | 14 | 110 | 14 (91) | 110 |  |  |
| 9 | 1 | Khadga | 9 | 9 | 110 | 9 (104) | 110 |  |  |
| 9 | 1 | Khadga | 11 | 11 | 110 | 11 (102) | 110 |  |  |
| 9 | 1 | Khadga | 10 | 10 | 110 | 9 (99) | 110 |  |  |
| 9 | 1 | Khadga | 12 | 12 | 110 | 12 (76) | 110 |  |  |
| 9 | 1 | Khadga | 11 | 11 | 110 | 11 (98) | 110 |  |  |
| 9 | 1 | Khadga | 11 | 12 | 110 | 12 (104) | 110 |  |  |
| 9 | 1 | Khadga | 12 | 12 | 110 | 12 (93) | 110 |  |  |
| 9 | 1 | Khadga | 11 | 11 | 110 | 12 (92) | 110 |  |  |
| 9 | 1 | Rudra | 12 | 12 | 110 | 14 (-) | 110 |  | * |
|  |  |  |  |  |  |  |  |  |  |
| 9 | 2 | Khadga | 9 | 9 | 110 | 9 (105) | 110 |  |  |
| 9 | 2 | Khadga | 9 | 9 | 110 | 8 (110) | 110 |  |  |
| 9 | 2 | Khadga | 11 | 12 | 110 | 12 (106) | 110 |  |  |
| 9 | 2 | Khadga | 11 | 11 | 110 | 11 (106) | 110 |  |  |
| 9 | 2 | Khadga | 12 | 12 | 110 | 12 (102) | 110 |  |  |
| 9 | 2 | Rudra | 11 | 12 | 110 | 12 (-) | 110 |  |  |
| 9 | 2 | Rudra | 13 | 13 | 110 | 14 (-) | 110 |  |  |
| 9 | 2 | Rudra | 13 | 13 | 110 | 14 (-) | 110 |  |  |
| 9 | 2 | Khadga | 9 | 9 | 110 | 9 (102) | 110 |  |  |
| 9 | 2 | Rudra | 12 | 13 | 110 | 14 (-) | 110 |  |  |
|  |  |  |  |  |  |  |  |  |  |
| 9 | 3 | Khadga | 3 | 3 | 110 | 3 (107) | 110 |  |  |
| 9 | 3 | Khadga | 9 | 9 | 110 | 8 (110) | 110 |  |  |
| 9 | 3 | Khadga | 12 | 12 | 110 | 12 (104) | 110 |  |  |
| 9 | 3 | Khadga | 10 | 11 | 110 | 10 (104) | 110 |  |  |
| 9 | 3 | Khadga | 12 | 12 | 110 | 12 (102) | 110 |  |  |
| 9 | 3 | Khadga | 9 | 9 | 110 | 6 (103) | 110 |  | * |
| 9 | 3 | Khadga | 3 | 3 | 110 | 3 (106) | 110 |  |  |
| 9 | 3 | Khadga | 9 | 10 | 110 | 9 (102) | 110 |  |  |
| 9 | 3 | Khadga | 12 | 12 | 110 | 12 (100) | 110 |  |  |
| 9 | 3 | Khadga | 11 | 12 | 110 | 12 (91) | 110 |  |  |
|  |  |  |  |  |  |  |  |  |  |
| 9 | 4 | Khadga | 11 | 11 | 110 | 11 (107) | 110 |  |  |
| 9 | 4 | Khadga | 11 | 12 | 110 | 12 (105) | 110 |  |  |
| 9 | 4 | Khadga | 10 | 11 | 110 | 11 (109) | 110 |  |  |
| 9 | 4 | Khadga | 10 | 10 | 110 | 10 (105) | 110 |  |  |
| 9 | 4 | Khadga | 9 | 9 | 110 | 9 (107) | 110 |  |  |
| 9 | 4 | Khadga | 11 | 11 | 110 | 11 (103) | 110 |  |  |
| 9 | 4 | Khadga | 11 | 12 | 110 | 12 (103) | 110 |  |  |
| 9 | 4 | Khadga | 12 | 12 | 110 | 12 (90) | 110 |  |  |
| 9 | 4 | Khadga | 4 | 4 | 110 | 3 (102) | 110 |  |  |
| 9 | 4 | Khadga | 11 | 12 | 110 | 12 (107) | 110 |  |  |
|  |  |  |  |  |  |  |  |  |  |

| Motor Milestones Validation Study April 2003 | | | | | | | | |  |
| --- | --- | --- | --- | --- | --- | --- | --- | --- | --- |
| W | S | TLI Name | MM# TLI Saw | MM# Mom Told TLI | NNIPS Week | MM# Mom Told WD | NNIPS Wk # Checked | Comments |  |
| 1 | 1 | Sulochana | 11 | 12 | 119 | 12 (98) | 119 |  |  |
| 1 | 1 | Sulochana | 12 | 12 | 119 | 12 (90) | 119 |  |  |
| 1 | 1 | Sulochana | 12 | 12 | 119 | 14 (sent) | 119 |  | ***** |
| 1 | 1 | Sulochana | 11 | 12 | 119 | 12 (99) | 119 |  |  |
| 1 | 1 | Sulochana | 11 | 12 | 119 | 13 (118) | 119 |  |  |
| 1 | 1 | Sulochana | 11 | 12 | 119 | 11 (111) | 119 |  |  |
| 1 | 1 | Sulochana | 11 | 12 | 119 | 12 (95) | 119 |  |  |
| 1 | 1 | Sulochana | 11 | 13 | 119 | 13 (111) | 119 |  |  |
| 1 | 1 | Sulochana | 13 | 13 | 119 | 12 (104) | 119 |  |  |
| 1 | 1 | Sulochana | 11 | 11 | 119 | 12 (106) | 119 |  |  |
|  |  |  |  |  |  |  |  |  |  |
| 1 | 2 | Dipak | 11 | 12 | 119 | 14 (sent) | 119 |  | ***** |
| 1 | 2 | Dipak | 12 | 13 | 119 | 14 (sent) | 119 |  |  |
| 1 | 2 | Dipak | 9 | 9 | 119 | 10 (116) | 119 |  |  |
| 1 | 2 | Dipak | 11 | 11 | 119 | 11 (112) | 119 |  |  |
| 1 | 2 | Dipak | 10 | 10 | 119 | 10 (100) | 119 |  |  |
| 1 | 2 | Dipak | 9 | 9 | 119 | 9 (100) | 119 |  |  |
| 1 | 2 | Dipak | 12 | 12 | 119 | 12 (109) | 119 |  |  |
| 1 | 2 | Dipak | 3 | 3 | 119 | 3 (113) | 119 |  |  |
| 1 | 2 | Dipak | 9 | 9 | 119 | 7 (116) | 119 |  | ***** |
| 1 | 2 | Dipak | 12 | 13 | 119 | 12 (99) | 119 |  |  |
| 1 | 2 | Dipak | 12 | 12 | 119 | 14 (119) | 119 |  | ***** |
|  |  |  |  |  |  |  |  |  |  |
| 1 | 3 | Dipak | 14 | 14 | 119 | 12 (76) | 119 |  |  |
| 1 | 3 | Dipak | 12 | 12 | 119 | 12 (79) | 119 |  |  |
| 1 | 3 | Dipak | 11 | 11 | 119 | 10 (106) | 119 |  |  |
| 1 | 3 | Dipak | 9 | 9 | 119 | 8 (119) | 119 |  |  |
| 1 | 3 | Dipak | 12 | 13 | 119 | 12 (98) | 119 |  |  |
| 1 | 3 | Dipak | 12 | 13 | 119 | 12 (82) | 119 |  |  |
| 1 | 3 | Dipak | 9 | 9 | 119 | 4 (113) | 119 |  | ***** |
| 1 | 3 | Dipak | 9 | 9 | 119 | 7 (118) | 119 |  | ***** |
| 1 | 3 | Dipak | 12 | 12 | 119 | 12 (108) | 119 |  |  |
| 1 | 3 | Dipak | 12 | 12 | 119 | 11 (92) | 119 |  |  |
|  |  |  |  |  |  |  |  |  |  |
| 1 | 4 | Khadga | 4 | 6 | 119 | 4 (114) | 119 |  |  |
| 1 | 4 | Khadga | 9 | 9 | 119 | 2 (100) | 119 |  | ***** |
| 1 | 4 | Khadga | 9 | 10 | 119 | 9 (103) | 119 |  |  |
| 1 | 4 | Khadga | 9 | 10 | 119 | 9 (116) | 119 |  |  |
| 1 | 4 | Khadga | 9 | 9 | 119 | 9 (97) | 119 |  |  |
| 1 | 4 | Khadga | 11 | 11 | 119 | 11 (118) | 119 |  |  |
| 1 | 4 | Khadga | 4 | 5 | 119 | 2 (112) | 119 |  |  |
| 1 | 4 | Khadga | 11 | 11 | 119 | 12 (107) | 119 |  | ***** |
| 1 | 4 | Khadga | 5 | 7 | 119 | 8 (107) | 119 |  |  |
| 1 | 4 | Khadga | 10 | 11 | 119 | 10 (112) | 119 |  |  |
|  |  |  |  |  |  |  |  |  |  |
| 2 | 1 | Khadga | 10 | 11 | 119 | 10 (110) | 119 |  |  |
| 2 | 1 | Khadga | 12 | 12 | 119 | 12 (110) | 119 |  |  |
| 2 | 1 | Khadga | 5 | 6 | 119 | 2 (115) | 119 |  | ***** |
| 2 | 1 | Khadga | 10 | 10 | 119 | 10 (116) | 119 |  |  |
| 2 | 1 | Khadga | 11 | 11 | 119 | 12 (105) | 119 |  |  |
| 2 | 1 | Khadga | 7 | 7 | 119 | 5 (119) | 119 |  |  |
| 2 | 1 | Khadga | 11 | 12 | 119 | 12 (65) | 119 |  | ***** |
| 2 | 1 | Khadga | 10 | 10 | 119 | 9 (107) | 119 |  |  |
| 2 | 1 | Khadga | 11 | 11 | 119 | 11 (081) | 119 |  |  |
| 2 | 1 | Khadga | 11 | 11 | 119 | 10 (110) | 119 |  |  |
|  |  |  |  |  |  |  |  |  |  |
| 2 | 2 | Roshan | 6 | 6 | 119 | 3 (112) | 119 |  | ***** |
| 2 | 2 | Roshan | 11 | 12 | 119 | 12 (114) | 119 |  |  |
| 2 | 2 | Roshan | 12 | 12 | 119 | 12 (109) | 119 |  |  |
| 2 | 2 | Roshan | 11 | 12 | 119 | 12 (94)/14 (119) | 119 |  |  |
| 2 | 2 | Roshan | 12 | 12 | 119 | 12 (109)/14 (119) | 119 |  |  |
| 2 | 2 | Roshan | 10 | 10 | 119 | 9 (94) | 119 |  |  |
| 2 | 2 | Roshan | 12 | 12 | 119 | 14 (-)/13 (119) | 119 |  |  |
| 2 | 2 | Rudra | 9 | 9 | 119 | 9 (103) | 119 |  |  |
| 2 | 2 | Rudra | 9 | 9 | 119 | 6 (117) | 119 |  | ***** |
| 2 | 2 | Rudra | 11 | 11 | 119 | 12 (114) | 119 |  |  |
|  |  |  |  |  |  |  |  |  |  |
| 2 | 3A | Sulochana | 9 | 9 | 119 | 9 (114) | 119 |  |  |
| 2 | 3A | Sulochana | 11 | 12 | 119 | 12 (112) | 119 |  |  |
| 2 | 3A | Sulochana | 12 | 12 | 119 | 12 (111) | 119 |  |  |
| 2 | 3A | Sulochana | 11 | 12 | 119 | 13 (119) | 119 |  |  |
| 2 | 3A | Sulochana | 10 | 10 | 119 | 10 (112) | 119 |  |  |
| 2 | 3A | Sulochana | 11 | 11 | 119 | 11 (116) | 119 |  |  |
| 2 | 3A | Sulochana | 12 | 14 | 119 | 13 (111) | 119 |  |  |
| 2 | 3A | Sulochana | 11 | 12 | 119 | 14 (sent) | 119 |  | ***** |
| 2 | 3A | Sulochana | 12 | 11 | 119 | 14 (sent) | 119 |  | ***** |
| 2 | 3A | Sulochana | 11 | 11 | 119 | 11 (115) | 119 |  |  |
| 2 | 3A | Sulochana | 11 | 12 | 119 | 12 (92) | 119 |  |  |
|  |  |  |  |  |  |  |  |  |  |
| 2 | 3B | Khadga | 11 | 12 | 119 | 12 (104) | 119 |  |  |
| 2 | 3B | Khadga | 11 | 12 | 119 | 12 (116) | 119 |  |  |
| 2 | 3B | Khadga | 10 | 10 | 119 | 10 (118) | 119 |  |  |
| 2 | 3B | Khadga | 11 | 11 | 119 | 12 (118) | 119 |  |  |
| 2 | 3B | Khadga | 11 | 11 | 119 | 11 (111) | 119 |  |  |
| 2 | 3B | Khadga | 9 | 9 | 119 | 9 (113) | 119 |  |  |
| 2 | 3B | Khadga | 11 | 11 | 119 | 12 (118) | 119 |  |  |
| 2 | 3B | Khadga | 11 | 11 | 119 | 11 (107) | 119 |  |  |
| 2 | 3B | Khadga | 5 | 6 | 119 | 5 (118) | 119 |  |  |
| 2 | 3B | Khadga | 2 | 2 | 119 | 2 (118) | 119 |  |  |
|  |  |  |  |  |  |  |  |  |  |
| 3 | 1 | Kiran | 8 | 8 | 119 | 11 (73) | 119 |  | ***** |
| 3 | 1 | Kiran | 11 | 11 | 119 | 11 (101) | 119 |  |  |
| 3 | 1 | Kiran | 9 | 9 | 119 | 6 (118) | 119 |  | ***** |
| 3 | 1 | Kiran | 12 | 13 | 119 | 12 (106) | 119 |  |  |
| 3 | 1 | Kiran | 6 | 6 | 119 | 2 (113) | 119 |  | ***** |
| 3 | 1 | Kiran | 12 | 12 | 119 | 12 (89) | 119 |  |  |
| 3 | 1 | Kiran | 11 | 12 | 119 | 11 (112) | 119 |  |  |
| 3 | 1 | Kiran | 11 | 12 | 119 | 12 (82) | 119 |  |  |
| 3 | 1 | Kiran | 10 | 11 | 119 | 11 (115) | 119 |  |  |
| 3 | 1 | Kiran | 11 | 11 | 119 | 11 (102) | 119 |  |  |
|  |  |  |  |  |  |  |  |  |  |
| 3 | 2 | Kiran | 11 | 11 | 119 | 12 (118) | 119 |  |  |
| 3 | 2 | Kiran | 12 | 14 | 119 | 12 (81) | 119 |  |  |
| 3 | 2 | Kiran | 10 | 11 | 119 | 10 (112) | 119 |  |  |
| 3 | 2 | Kiran | 9 | 9 | 119 | 3 (115) | 119 |  | ***** |
| 3 | 2 | Kiran | 9 | 10 | 119 | 10 (113) | 119 |  |  |
| 3 | 2 | Kiran | 11 | 11 | 119 | 10 (108) | 119 |  |  |
| 3 | 2 | Kiran | 12 | 12 | 119 | 14 (119) | 119 |  | ***** |
| 3 | 2 | Kiran | 1 | 2 | 119 | 3 (119) | 119 |  |  |
| 3 | 2 | Kiran | 11 | 14 | 119 | 11 (99) | 119 |  | ***** |
| 3 | 2 | Kiran | 3 | 3 | 119 | 1 (106) | 119 |  | ***** |
|  |  |  |  |  |  |  |  |  |  |
| 4 | 1 | Menuka | 12 | 13 | 119 | 12 (104) | 119 |  |  |
| 4 | 1 | Menuka | 9 | 9 | 119 | 8 (118) | 119 |  |  |
| 4 | 1 | Menuka | 9 | 9 | 119 | 7 (118) | 119 |  | ***** |
| 4 | 1 | Menuka | 11 | 12 | 119 | 11 (102) | 119 |  |  |
| 4 | 1 | Menuka | 9 | 9 | 119 | 8 (116) | 119 |  |  |
| 4 | 1 | Menuka | 12 | 12 | 119 | 11 (93) | 119 |  |  |
| 4 | 1 | Menuka | 10 | 11 | 119 | 10 (98) | 119 |  |  |
| 4 | 1 | Menuka | 14 | 14 | 119 | 12 (71) | 119 |  | ***** |
| 4 | 1 | Menuka | 5 | 5 | 119 | 3 (117) | 119 |  | ***** |
| 4 | 1 | Menuka | 12 | 13 | 119 | 11 (99) | 119 |  | ***** |
|  |  |  |  |  |  |  |  |  |  |
| 4 | 2 | Menuka | 12 | 12 | 119 | 12 (85) | 119 |  |  |
| 4 | 2 | Menuka | 9 | 9 | 119 | 7 (117) | 119 |  | ***** |
| 4 | 2 | Menuka | 12 | 12 | 119 | 12 (117) | 119 |  |  |
| 4 | 2 | Menuka | 9 | 9 | 119 | 5 (118) | 119 |  | ***** |
| 4 | 2 | Menuka | 4 | 6 | 119 | 4 (112) | 119 |  | ***** |
| 4 | 2 | Menuka | 12 | 12 | 119 | 12 (109) | 119 |  |  |
| 4 | 2 | Menuka | 12 | 12 | 119 | 12 (114) | 119 |  |  |
| 4 | 2 | Menuka | 10 | 10 | 119 | 10 (114) | 119 |  |  |
| 4 | 2 | Menuka | 11 | 12 | 119 | 12 (87) | 119 |  |  |
| 4 | 2 | Menuka | 10 | 10 | 119 | 8 (117) | 119 |  | ***** |
|  |  |  |  |  |  |  |  |  |  |
| 5 | 1 | Padam | 10 | 10 | 119 | 10 (119) | 119 |  |  |
| 5 | 1 | Padam | 12 | 12 | 119 | 12 (51) | 119 |  |  |
| 5 | 1 | Padam | 12 | 11 | 119 | 12 (92) | 119 |  |  |
| 5 | 1 | Padam | 11 | 12 | 119 | 12 (100) | 119 |  |  |
| 5 | 1 | Padam | 12 | 11 | 119 | 14 (119) | 119 |  | ***** |
| 5 | 1 | Padam | 11 | 12 | 119 | 12 (81) | 119 |  |  |
| 5 | 1 | Padam | 3 | 3 | 119 | 2 (111) | 119 |  |  |
| 5 | 1 | Padam | 11 | 12 | 119 | 12 (116) | 119 |  |  |
| 5 | 1 | Padam | 11 | 11 | 119 | 12 (92) | 119 |  |  |
| 5 | 1 | Padam | 10 | 10 | 119 | 10 (111) | 119 |  |  |
|  |  |  |  |  |  |  |  |  |  |
| 6 | 1 | Shyam | 5 | 6 | 119 | 6 (105) | 119 |  |  |
| 6 | 1 | Shyam | 11 | 12 | 119 | 13 (117) | 119 |  |  |
| 6 | 1 | Shyam | 9 | 10 | 119 | 10 (119) | 119 |  |  |
| 6 | 1 | Shyam | 1 | 1 | 119 | 1 (108) | 119 |  |  |
| 6 | 1 | Shyam | 12 | 14 | 119 | 14 (109) | 119 |  |  |
| 6 | 1 | Shyam | 10 | 10 | 119 | 10 (103) | 119 |  |  |
| 6 | 1 | Shyam | 8 | 9 | 119 | 9 (113) | 119 |  |  |
| 6 | 1 | Shyam | 2 | 2 | 119 | 6 (118) | 119 |  | ***** |
| 6 | 1 | Shyam | 8 | 8 | 119 | 8 (111) | 119 |  |  |
| 6 | 1 | Shyam | 11 | 11 | 119 | 12 (117) | 119 |  |  |
|  |  |  |  |  |  |  |  |  |  |
| 6 | 2 | Shyam | 11 | 12 | 119 | 13 (112) | 119 |  |  |
| 6 | 2 | Shyam | 11 | 11 | 119 | 12 (95) | 119 |  |  |
| 6 | 2 | Shyam | 11 | 12 | 119 | 13 (112) | 119 |  |  |
| 6 | 2 | Shyam | 12 | 12 | 119 | 13 (111) | 119 |  |  |
| 6 | 2 | Shyam | 11 | 12 | 119 | 12 (89) | 119 |  |  |
| 6 | 2 | Shyam | 10 | 11 | 119 | 11 (96) | 119 |  |  |
| 6 | 2 | Shyam | 12 | 12 | 119 | 11 (111) | 119 |  |  |
| 6 | 2 | Shyam | 11 | 11 | 119 | 11 (116) | 119 |  |  |
| 6 | 2 | Shyam | 11 | 12 | 119 | 12 (59) | 119 |  |  |
| 6 | 2 | Shyam | 2 | 2 | 119 | 2 (117) | 119 |  |  |
|  |  |  |  |  |  |  |  |  |  |
| 6 | 3 | Rudra | 11 | 12 | 119 | 14 (117) | 119 |  | ***** |
| 6 | 3 | Rudra | 1 | 2 | 119 | 2 (119) | 119 |  |  |
| 6 | 3 | Rudra | 12 | 12 | 119 | 11 (64) | 119 |  |  |
| 6 | 3 | Rudra | 10 | 11 | 119 | 11 (118) | 119 |  |  |
| 6 | 3 | Rudra | 11 | 11 | 119 | 11 (117) | 119 |  |  |
| 6 | 3 | Rudra | 12 | 12 | 119 | 12 (84) | 119 |  |  |
| 6 | 3 | Rudra | 2 | 2 | 119 | 5 (119) | 119 |  | ***** |
| 6 | 3 | Rudra | 11 | 12 | 119 | 12 (90) | 119 |  |  |
| 6 | 3 | Rudra | 11 | 11 | 119 | 14 (sent) | 119 |  | ***** |
| 6 | 3 | Rudra | 11 | 11 | 119 | 11 (90) | 119 |  |  |
| 6 | 3 | Rudra | 12 | 12 | 119 | 12 (73) | 119 |  |  |
|  |  |  |  |  |  |  |  |  |  |
| 7 | 1 | Ishwori | 11 | 12 | 119 | 12 (86) | 119 |  |  |
| 7 | 1 | Ishwori | 3 | 6 | 119 | 3 (117) | 119 | 4 & 5 not complete |  |
| 7 | 1 | Ishwori | 7 | 8 | 119 | 8 (118) | 119 |  |  |
| 7 | 1 | Ishwori | 11 | 12 | 119 | 12 (102) | 119 |  |  |
| 7 | 1 | Ishwori | 3 | 3 | 119 | 3 (113) | 119 |  |  |
| 7 | 1 | Ishwori | 10 | 11 | 119 | 12 (119) | 119 |  |  |
| 7 | 1 | Ishwori | 10 | 11 | 119 | 11 (106) | 119 |  |  |
| 7 | 1 | Ishwori | 11 | 12 | 119 | 12 (94) | 119 |  |  |
| 7 | 1 | Ishwori | 3 | 3 | 119 | 3 (116) | 119 |  |  |
| 7 | 1 | Ishwori | 11 | 12 | 119 | 12 (105) | 119 |  |  |
|  |  |  |  |  |  |  |  |  |  |
| 8 | 1 | Ram NR | 11 | 12 | 119 | 12 (100) | 119 |  |  |
| 8 | 1 | Ram NR | 11 | 12 | 119 | 12 (116) | 119 |  |  |
| 8 | 1 | Ram NR | 10 | 10 | 119 | 10 (111) | 119 |  |  |
| 8 | 1 | Ram NR | 11 | 11 | 119 | 11 (117) | 119 |  |  |
| 8 | 1 | Ram NR | 14 | 14 | 119 | 12 (100) | 119 |  |  |
| 8 | 1 | Ram NR | 11 | 12 | 119 | 12 (112) | 119 |  |  |
| 8 | 1 | Ram NR | 11 | 11 | 119 | 11 (108) | 119 |  |  |
| 8 | 1 | Ram NR | 11 | 12 | 119 | 12 (100) | 119 |  |  |
| 8 | 1 | Ram NR | 9 | 10 | 119 | 11 (113) | 119 |  |  |
| 8 | 1 | Ram NR | 11 | 11 | 119 | 12 (116) | 119 |  |  |
|  |  |  |  |  |  |  |  |  |  |
| 8 | 2 | Ram NR | 12 | 13 | 119 | 12 (96) | 119 |  |  |
| 8 | 2 | Ram NR | 3 | 3 | 119 | 2 (116) | 119 |  |  |
| 8 | 2 | Ram NR | 14 | 14 | 119 | 13 (117) | 119 |  |  |
| 8 | 2 | Ram NR | 10 | 10 | 119 | 10 (115) | 119 |  |  |
| 8 | 2 | Ram NR | 12 | 12 | 119 | 12 (119) | 119 |  |  |
| 8 | 2 | Ram NR | 11 | 12 | 119 | 11 (98) | 119 |  |  |
| 8 | 2 | Ram NR | 11 | 12 | 119 | 11 (87) | 119 |  |  |
| 8 | 2 | Ram NR | 10 | 10 | 119 | 9 (106) | 119 |  |  |
| 8 | 2 | Ram NR | 4 | 6 | 119 | 2 (112) | 119 |  | ***** |
| 8 | 2 | Ram NR | 9 | 9 | 119 | 8 (118) | 119 |  |  |
|  |  |  |  |  |  |  |  |  |  |
| 9 | 1 | Nir BDR | 6 | 6 | 119 | 6 (114) | 119 |  |  |
| 9 | 1 | Nir BDR | 12 | 13 | 119 | 12 (113) | 119 |  |  |
| 9 | 1 | Nir BDR | 11 | 12 | 119 | 12 (114) | 119 |  |  |
| 9 | 1 | Nir BDR | 11 | 12 | 119 | 12 (119) | 119 |  |  |
| 9 | 1 | Nir BDR | 11 | 11 | 119 | 10 (111) | 119 |  |  |
| 9 | 1 | Nir BDR | 12 | 13 | 119 | 12 (076) | 119 |  |  |
| 9 | 1 | Nir BDR | 11 | 12 | 119 | 11 (114) | 119 | see note |  |
| 9 | 1 | Nir BDR | 11 | 11 | 119 | 11 (98) | 119 |  |  |
| 9 | 1 | Nir BDR | 11 | 12 | 119 | 11 (91) | 119 |  |  |
| 9 | 1 | Nir BDR | 12 | 12 | 119 | 12 (104) | 119 |  |  |
|  |  |  |  |  |  |  |  |  |  |
| 9 | 2 | Nir BDR | 12 | 12 | 119 | 11 (85) | 119 |  |  |
| 9 | 2 | Nir BDR | 11 | 12 | 119 | 12 (102) | 119 |  |  |
| 9 | 2 | Nir BDR | 12 | 13 | 119 | 12 (86) | 119 |  |  |
| 9 | 2 | Nir BDR | 11 | 11 | 119 | 10 (116) | 119 |  |  |
| 9 | 2 | Nir BDR | 9 | 10 | 119 | 10 (117) | 119 |  |  |
| 9 | 2 | Nir BDR | 8 | 9 | 119 | 4 (107) | 119 | WD data from aunt, not mom | ***** |
| 9 | 2 | Nir BDR | 11 | 12 | 119 | 12 (118) | 119 |  |  |
| 9 | 2 | Nir BDR | 9 | 9 | 119 | 10 (104) | 119 |  |  |
| 9 | 2 | Nir BDR | 10 | 11 | 119 | 10 (112) | 119 |  |  |
| 9 | 2 | Nir BDR | 11 | 12 | 119 | 12 (106)/14 (112) | 119 |  |  |
|  |  |  |  |  |  |  |  |  |  |
| 9 | 3 | Kesab | 4 | 5 | 119 | 6 (112) | 119 |  |  |
| 9 | 3 | Kesab | 11 | 12 | 119 | 13 (106) | 119 |  |  |
| 9 | 3 | Kesab | 9 | 9 | 119 | 9 (111) | 119 |  |  |
| 9 | 3 | Kesab | 11 | 11 | 119 | 12 (114) | 119 |  |  |
| 9 | 3 | Kesab | 11 | 12 | 119 | 12 (104) | 119 |  |  |
| 9 | 3 | Kesab | 11 | 12 | 119 | 12 (102) | 119 |  |  |
| 9 | 3 | Kesab | 9 | 9 | 119 | 10 (119) | 119 |  |  |
| 9 | 3 | Kesab | 12 | 12 | 119 | 13 (116) | 119 |  |  |
| 9 | 3 | Kesab | 11 | 11 | 119 | 11 (117) | 119 |  |  |
| 9 | 3 | Kesab | 11 | 12 | 119 | 11 (113) | 119 |  |  |
|  |  |  |  |  |  |  |  |  |  |
| 9 | 4 | Kesab | 11 | 14 | 119 | 12 (104) | 119 |  |  |
| 9 | 4 | Kesab | 10 | 10 | 119 | 10 (117) | 119 |  |  |
| 9 | 4 | Kesab | 11 | 12 | 119 | 12 (115) | 119 |  |  |
| 9 | 4 | Kesab | 11 | 11 | 119 | 11 (113) | 119 |  |  |
| 9 | 4 | Kesab | 11 | 11 | 119 | 11 (116) | 119 |  |  |
| 9 | 4 | Kesab | 11 | 12 | 119 | 12 (109) | 119 |  |  |
| 9 | 4 | Kesab | 12 | 14 | 119 | 12 (115) | 119 |  |  |
| 9 | 4 | Kesab | 9 | 9 | 119 | 10 (118) | 119 |  |  |
| 9 | 4 | Kesab | 11 | 12 | 119 | 13 (116) | 119 |  |  |
| 9 | 4 | Kesab | 12 | 14 | 119 | 12 (90) | 119 |  |  |
|  |  |  |  |  |  |  |  |  |  |

Anthropometry Standardization

| **Anthropometry Standardization Exercise** | | | | | |  |  |  |
| --- | --- | --- | --- | --- | --- | --- | --- | --- |
| **Clinic Staff: Summer 2001/ Spring 2002** | | | | | |  |  |  |
| **MUAC** |  |  |  |  |  |  |  |  |
| **Child** | **Rajeswori** | **Kalawati** | **Difference** | **Diff/Supp.** | **Sign** |  |  |  |
| 1 | 13.4 | 13.7 | 0.3 | 2.23 | pos |  |  |  |
| 2 | 13.8 | 13.8 | 0 | 0 |  |  |  |  |
| 3 | 13.3 | 13.5 | 0.2 | 1.5 | pos |  |  |  |
| 4 | 13.5 | 13.6 | 0.1 | 0.74 | pos |  |  |  |
| 5 | 14.1 | 13.4 | 0.7 | 4.96 | neg |  |  |  |
| 6 | 13.4 | 13.6 | 0.2 | 1.49 | pos |  |  |  |
| 7 | 15.1 | 15.5 | 0.4 | 2.65 | pos |  |  |  |
| 8 | 12.4 | 12.8 | 0.4 | 3.22 | pos |  |  |  |
| 9 | 7.4 | 7.3 | 0.1 | 1.35 | neg |  |  |  |
| 10 | 14.4 | 14.5 | 0.1 | 0.69 | pos |  |  |  |
| **HC** |  |  |  |  |  |  |  |  |
| **Child** | **Rajeswori** | **Kalawati** | **Difference** | **Diff/Supp.** | **Sign** |  |  |  |
| 1 | 41.2 | 41.4 | 0.2 | 0.49 | pos |  |  |  |
| 2 | 42.1 | 42 | 0.1 | 0.24 | neg |  |  |  |
| 3 | 42.6 | 42.7 | 0.1 | 0.23 | pos |  |  |  |
| 4 | 43.2 | 42.8 | 0.4 | 0.93 | neg |  |  |  |
| 5 | 46 | 45.7 | 0.3 | 0.65 | neg |  |  |  |
| 6 | 46.4 | 46.7 | 0.3 | 0.65 | pos |  |  |  |
| 7 | 46 | 46.6 | 0.6 | 1.3 | pos |  |  |  |
| 8 | 42.6 | 42 | 0.6 | 1.41 | neg |  |  |  |
| 9 | 35.6 | 35.5 | 0.1 | 0.28 | neg |  |  |  |
| 10 | 40.9 | 41 | 0.1 | 0.24 | pos |  |  |  |
| **Tricep Skinfold Thickness** | | |  |  |  |  |  |  |
| **Child** | **Rajeswori** | **Kalawati** | **Difference** | **Diff/Supp.** | **Sign** |  |  |  |
| 1 | 7.9 | 8 | 0.1 | 1.27 | pos |  |  |  |
| 2 | 8.3 | 9.3 | 1 | 12.05 | pos |  |  |  |
| 3 | 7.1 | 7.5 | 0.4 | 5.63 | pos |  |  |  |
| 4 | 8.7 | 9.5 | 0.8 | 9.2 | pos |  |  |  |
| 5 | 6.1 | 5.9 | 0.2 | 3.28 | neg |  |  |  |
| 6 | 7.5 | 7.5 | 0 | 0 |  |  |  |  |
| 7 | 9.5 | 9.9 | 0.4 | 4.21 | pos |  |  |  |
| 8 | 6.4 | 6.3 | 0.1 | 1.56 | neg |  |  |  |
| 9 | 3.7 | 3.3 | 0.4 | 10.81 | neg |  |  |  |
| 10 | 9.7 | 9.9 | 0.2 | 2.06 | pos |  |  |  |
|  |  |  |  |  |  |  |  |  |
|  |  |  |  |  |  |  |  |  |
| **Weight** |  |  |  |  |  |  |  |  |
| **Child** | **Rajeswori** | **Kalawati** | **Difference** | **Diff/Supp.** | **Sign** |  |  |  |
| 1 | 7.1 | 7.1 | 0 | 0 |  |  |  |  |
| 2 | 7.6 | 7.6 | 0 | 0 |  |  |  |  |
| 3 | 7.1 | 7.1 | 0 | 0 |  |  |  |  |
| 4 | 7.3 | 7.2 | 0.1 | 1.37 | neg |  |  |  |
| 5 | 9.8 | 9.8 | 0 | 0 |  |  |  |  |
| 6 | 8.6 | 8.5 | 0.1 | 1.16 | neg |  |  |  |
| 7 | 11 | 11 | 0 | 0 |  |  |  |  |
| 8 | 7.2 | 7.3 | 0.1 | 1.39 | pos |  |  |  |
| 9 | 2.6 | 2.6 | 0 | 0 |  |  |  |  |
| 10 | 7 | 6.9 | 0.1 | 1.43 | neg |  |  |  |
| **Recumbent Length** | |  |  |  |  |  |  |  |
| **Child** | **Rajeswori** | **Kalawati** | **Difference** | **Diff/Supp.** | **Sign** |  |  |  |
| 1 | 64.8 | 64 | 0.2 | 0.31 | pos |  |  |  |
| 2 | 67.2 | 65.4 | 1.8 | 2.68 | neg |  |  |  |
| 3 | 66.8 | 66.5 | 0.3 | 0.45 | neg |  |  |  |
| 4 | 67 | 66.4 | 0.6 | 0.9 | neg |  |  |  |
| 5 | 82.1 | 82.2 | 0.1 | 0.12 | pos |  |  |  |
| 6 | 75 | 74.2 | 0.8 | 1.07 | neg |  |  |  |
| 7 | 78.2 | 78.2 | 0 | 0 |  |  |  |  |
| 8 | 70 | 70 | 0 | 0 |  |  |  |  |
| 9 | 53.2 | 53.1 | 0.1 | 0.19 | neg |  |  |  |
| 10 | 61.7 | 62.1 | 0.4 | 0.65 | pos |  |  |  |
| **Head Circumference** | |  |  |  | **Weight** |  |  |  |
| **Child** | **Rajeswori** | **Menuka** | **% Agreement** |  | **Child** | **Rajeswori** | **Menuka** | **% Agree.** |
| 1 | 44.8 | 44.8 | 100 |  | 1 | 8.1 | 8.2 | 98.8 |
| 2 | 41.5 | 41.5 | 100 |  | 2 | 6.6 | 6.7 | 98.5 |
| 3 | 43.5 | 43.5 | 100 |  | 3 | 6.9 | 6.8 | 101.5 |
| 4 | 41.3 | 41.1 | 100.5 |  | 4 | 5.9 | 6 | 98.3 |
| 5 | 40.1 | 40.1 | 100 |  | 5 | 4.6 | 4.6 | 100 |
| 6 | 40.7 | 40.7 | 100 |  | 6 | 5.6 | 5.6 | 100 |
| 7 | 42.3 | 42 | 100.7 |  | 7 | 6.3 | 6.4 | 98.4 |
| 8 | 40.5 | 40.7 | 99.5 |  | 8 | 6.6 | 6.7 | 98.5 |
| 9 | 44.4 | 44.4 | 100 |  | 9 | 7.6 | 7.6 | 100 |
| 10 | 44.9 | 44.6 | 100.7 |  | 10 | 7.2 | 7.2 | 100 |
| **Triceps Skinfold** | |  |  |  | **Recumbent Length** | |  |  |
| **Child** | **Rajeswori** | **Menuka** | **% Agreement** |  | **Child** | **Rajeswori** | **Menuka** | **% Agree.** |
| 1 | 9.4 | 9.4 | 100 |  | 1 | 73.4 | 71.8 | 102.2 |
| 2 | 7.4 | 7.6 | 103 |  | 2 | 65.1 | 65 | 100.2 |
| 3 | 5.2 | 5.2 | 100 |  | 3 | 69.8 | 69.9 | 99.9 |
| 4 | 4.2 | 4.3 | 97.7 |  | 4 | 68.2 | 67.7 | 100.7 |
| 5 | 5.6 | 5.7 | 98.2 |  | 5 | 58.8 | 58.9 | 99.8 |
| 6 | 5.1 | 5.3 | 96.2 |  | 6 | 61.9 | 61.9 | 100 |
| 7 | 5.6 | 5.6 | 100 |  | 7 | 65.5 | 65.6 | 99.8 |
| 8 | 8 | 8 | 100 |  | 8 | 64.5 | 64.6 | 99.8 |
| 9 | 4.8 | 4.8 | 100 |  | 9 | 72.8 | 72.8 | 100 |
| 10 | 6.8 | 6.8 | 100 |  | 10 | 72.2 | 72.2 | 100 |

| **DIMAG MUAC Standardization (December 2001)** | | |  |
| --- | --- | --- | --- |
| **Worker ID** | **Worker's Measurement** | **Rajeswori's Measurement (GS)** | **% Agreement** |
| 213 (Kiran) | 12.6 | 13 | 96.9 |
|  | 12.2 | 12.5 | 97.6 |
|  | 13.1 | 13.1 | 100 |
|  | 12.4 | 12.8 | 96.9 |
|  | 14.1 | 14.3 | 98.6 |
|  | 14 | 14.6 | 95.9 |
|  | 13.5 | 13.7 | 98.5 |
|  | 11.5 | 11.9 | 96.6 |
|  | 13 | 12.5 | 104 |
|  | 13.5 | 13.5 | 100 |
|  |  |  |  |
| **Worker ID** | **Worker's Measurement** | **Rajeswori's Measurement (GS)** | **% Agreement** |
| 210 (Shyam) | 13 | 13 | 100 |
|  | 12 | 12.5 | 96 |
|  | 14 | 13.1 | 106.9 |
|  | 12.4 | 12.8 | 96.9 |
|  | 14.2 | 14.3 | 99.3 |
|  | 14 | 14.6 | 95.9 |
|  | 13.5 | 13.7 | 98.5 |
|  | 11.6 | 11.9 | 97.5 |
|  | 12.5 | 12.5 | 100 |
|  | 13.1 | 13.5 | 97 |
|  |  |  |  |
| **Worker ID** | **Worker's Measurement** | **Rajeswori's Measurement (GS)** | **% Agreement** |
| 210 (Shyam) | 9.2 | 9.2 | 100 |
| REDO | 13.6 | 13.5 | 100.7 |
|  | 12.2 | 12.1 | 100.8 |
|  | 13.9 | 14 | 99.3 |
|  | 15.3 | 15.4 | 99.4 |
|  | 14.6 | 14.5 | 100.7 |
|  | 14.1 | 14.1 | 100 |
|  | 13 | 13 | 100 |
|  | 13.5 | 13.5 | 100 |
|  | 13.9 | 13.8 | 100.7 |
|  |  |  |  |
| **Worker ID** | **Worker's Measurement** | **Rajeswori's Measurement (GS)** | **% Agreement** |
| 211 (Sulochana) | 13.3 | 13 | 102.3 |
|  | 12.4 | 12.5 | 99.2 |
|  | 13.7 | 13.1 | 104.6 |
|  | 13.1 | 12.8 | 102.3 |
|  | 14.9 | 14.3 | 104.2 |
|  | 14.6 | 14.6 | 100 |
|  | 13.5 | 13.7 | 98.5 |
|  | 12.5 | 11.9 | 105 |
|  | 13.2 | 12.5 | 105.6 |
|  | 13.7 | 13.5 | 101.5 |
|  |  |  |  |
| **Worker ID** | **Worker's Measurement** | **Rajeswori's Measurement (GS)** | **% Agreement** |
| 211 (Sulochana) | 9.2 | 9.2 | 100 |
| REDO | 13.5 | 13.5 | 100 |
|  | 12.2 | 12.1 | 100.8 |
|  | 14 | 14 | 100 |
|  | 15.3 | 15.4 | 99.4 |
|  | 14.5 | 14.5 | 100 |
|  | 14 | 14.1 | 99.3 |
|  | 13 | 13 | 100 |
|  | 13.7 | 13.5 | 101.5 |
|  | 13.9 | 13.8 | 100.7 |
|  |  |  |  |
| **Worker ID** | **Worker's Measurement** | **Rajeswori's Measurement (GS)** | **% Agreement** |
| 208 (Menuka) | 13.2 | 13 | 101.5 |
|  | 12.2 | 12.5 | 97.6 |
|  | 13.4 | 13.1 | 102.3 |
|  | 13 | 12.8 | 101.6 |
|  | 14 | 14.3 | 97.9 |
|  | 14.4 | 14.6 | 98.6 |
|  | 13.8 | 13.7 | 100.7 |
|  | 11.8 | 11.9 | 99.2 |
|  | 13 | 12.5 | 104 |
|  | 13.6 | 13.5 | 100.7 |
|  |  |  |  |
| **Worker ID** | **Worker's Measurement** | **Rajeswori's Measurement (GS)** | **% Agreement** |
| 216 (Ram NR) | 13 | 13 | 100 |
|  | 12.1 | 12.5 | 96.8 |
|  | 13.3 | 13.1 | 101.5 |
|  | 12.3 | 12.8 | 96.1 |
|  | 13.9 | 14.3 | 97.2 |
|  | 14.5 | 14.6 | 99.3 |
|  | 13.4 | 13.7 | 97.8 |
|  | 11.6 | 11.9 | 97.5 |
|  | 12.5 | 12.5 | 100 |
|  | 13.3 | 13.5 | 98.5 |
|  |  |  |  |
| **Worker ID** | **Worker's Measurement** | **Rajeswori's Measurement (GS)** | **% Agreement** |
| 202 (Dipak) | 13 | 13 | 100 |
|  | 12.3 | 12.5 | 98.4 |
|  | 13.4 | 13.1 | 102.3 |
|  | 12.1 | 12.8 | 94.5 |
|  | 13.9 | 14.3 | 97.2 |
|  | 13.8 | 14.6 | 94.5 |
|  | 13 | 13.7 | 94.8 |
|  | 11.7 | 11.9 | 98.3 |
|  | 12.6 | 12.5 | 100.8 |
|  | 12.9 | 13.5 | 95.6 |
|  |  |  |  |
| **Worker ID** | **Worker's Measurement** | **Rajeswori's Measurement (GS)** | **% Agreement** |
| 201 (Shishir) | 12.9 | 13 | 99.2 |
|  | 12.2 | 12.5 | 97.6 |
|  | 13.4 | 13.1 | 97.8 |
|  | 12.1 | 12.8 | 94.5 |
|  | 14.1 | 14.3 | 98.6 |
|  | 14.5 | 14.6 | 99.3 |
|  | 13.4 | 13.7 | 97.8 |
|  | 11.7 | 11.9 | 98.3 |
|  | 12.3 | 12.5 | 98.4 |
|  | 13 | 13.5 | 96.3 |
|  |  |  |  |
| **Worker ID** | **Worker's Measurement** | **Rajeswori's Measurement (GS)** | **% Agreement** |
| 215 (Nir BDR) | 12.9 | 13 | 99.2 |
|  | 12.4 | 12.5 | 99.2 |
|  | 13.4 | 13.1 | 102.3 |
|  | 12.8 | 12.8 | 100 |
|  | 14 | 14.3 | 97.9 |
|  | 13.8 | 14.6 | 94.5 |
|  | 13.6 | 13.7 | 99.3 |
|  | 11.4 | 11.9 | 95.8 |
|  | 12.9 | 12.5 | 103.2 |
|  | 13.2 | 13.5 | 97.8 |
|  |  |  |  |
| **Worker ID** | **Worker's Measurement** | **Rajeswori's Measurement (GS)** | **% Agreement** |
| 204 (Ishwori) | 13.1 | 13 | 100.8 |
|  | 12.5 | 12.5 | 100 |
|  | 13.6 | 13.1 | 103.8 |
|  | 12.5 | 12.8 | 97.7 |
|  | 14.2 | 14.3 | 99.3 |
|  | 14.4 | 14.6 | 98.6 |
|  | 13.8 | 13.7 | 100.7 |
|  | 12 | 11.9 | 100.8 |
|  | 12.6 | 12.5 | 100.8 |
|  | 13.5 | 13.5 | 100 |
|  |  |  |  |
| **Worker ID** | **Worker's Measurement** | **Rajeswori's Measurement (GS)** | **% Agreement** |
| 215 (Padam) | 12.9 | 13 | 99.2 |
|  | 12.3 | 12.5 | 98.4 |
|  | 13.7 | 13.1 | 104.6 |
|  | 12.4 | 12.8 | 96.9 |
|  | 14.1 | 14.3 | 98.6 |
|  | 14.4 | 14.6 | 98.6 |
|  | 13.9 | 13.7 | 101.5 |
|  | 12.2 | 11.9 | 97.5 |
|  | 12.8 | 12.5 | 102.4 |
|  | 13.3 | 13.5 | 98.5 |
|  |  |  |  |
| **Worker ID** | **Worker's Measurement** | **Rajeswori's Measurement (GS)** | **% Agreement** |
| 203 (Roshan) | 12.7 | 13 | 97.7 |
|  | 12.4 | 12.5 | 99.2 |
|  | 13.6 | 13.1 | 103.8 |
|  | 12.2 | 12.8 | 95.3 |
|  | 14.1 | 14.3 | 98.6 |
|  | 14.2 | 14.6 | 97.3 |
|  | 13.2 | 13.7 | 96.4 |
|  | 11.6 | 11.9 | 97.5 |
|  | 12.6 | 12.5 | 100.8 |
|  | 13.2 | 13.5 | 97.8 |
|  |  |  |  |
| **Worker ID** | **Worker's Measurement** | **Rajeswori's Measurement (GS)** | **% Agreement** |
| 207 (Ram Asesh) | 13 | 13 | 100 |
|  | 12.3 | 12.5 | 98.4 |
|  | 13.3 | 13.1 | 101.5 |
|  | 12.2 | 12.8 | 95.3 |
|  | 13.9 | 14.3 | 97.2 |
|  | 13.9 | 14.6 | 95.2 |
|  | 12.7 | 13.7 | 93.7 |
|  | 11.6 | 11.9 | 97.5 |
|  | 12.2 | 12.5 | 97.6 |
|  | 12.9 | 13.5 | 95.6 |
|  |  |  |  |
| **Worker ID** | **Worker's Measurement** | **Rajeswori's Measurement (GS)** | **% Agreement** |
| 206 (Rudra) | 12.8 | 13 | 98.5 |
|  | 12.5 | 12.5 | 100 |
|  | 13.6 | 13.1 | 103.8 |
|  | 12.4 | 12.8 | 96.9 |
|  | 14.1 | 14.3 | 98.6 |
|  | 14.3 | 14.6 | 97.9 |
|  | 13.3 | 13.7 | 97.1 |
|  | 12 | 11.9 | 100.8 |
|  | 13 | 12.5 | 104 |
|  | 13.5 | 13.5 | 100 |
|  |  |  |  |
| **Worker ID** | **Worker's Measurement** | **Rajeswori's Measurement (GS)** | **% Agreement** |
| 205 (Yadav) | 13.1 | 13 | 100.8 |
|  | 12.5 | 12.5 | 100 |
|  | 13.6 | 13.1 | 103.8 |
|  | 12.8 | 12.8 | 100 |
|  | 14.3 | 14.3 | 100 |
|  | 14.5 | 14.6 | 99.3 |
|  | 13.5 | 13.7 | 98.5 |
|  | 11.8 | 11.9 | 99.2 |
|  | 12.9 | 12.5 | 103.2 |
|  | 13.4 | 13.5 | 99.3 |
|  |  |  |  |
| **Worker ID** | **Worker's Measurement** | **Rajeswori's Measurement (GS)** | **% Agreement** |
| 209 (Khadga) | 13.2 | 13 | 101.5 |
|  | 12.3 | 12.5 | 98.4 |
|  | 13.7 | 13.1 | 104.6 |
|  | 12.8 | 12.8 | 100 |
|  | 14.5 | 14.3 | 101.4 |
|  | 14.4 | 14.6 | 98.6 |
|  | 13.7 | 13.7 | 100 |
|  | 12.2 | 11.9 | 102.5 |
|  | 12.6 | 12.5 | 100.8 |
|  | 13.6 | 13.5 | 100.7 |
|  |  |  |  |
| **Worker ID** | **Worker's Measurement** | **Rajeswori's Measurement (GS)** | **% Agreement** |
| 212 (Kesab) | 12.9 | 13 | 99.2 |
|  | 12.3 | 12.5 | 98.4 |
|  | 13.5 | 13.1 | 103.1 |
|  | 12.4 | 12.8 | 96.9 |
|  | 14.5 | 14.3 | 98.6 |
|  | 14.1 | 14.6 | 96.6 |
|  | 13.3 | 13.7 | 97.1 |
|  | 12 | 11.9 | 100.8 |
|  | 12.8 | 12.5 | 102.4 |
|  | 13.5 | 13.5 | 100 |

| **DIMAG Head Circumference Standardization (May 2002)** | | |  |
| --- | --- | --- | --- |
| **Worker ID** | **Worker's Measurement** | **Giri's Measurement (GS)** | **% Agreement** |
| 208 (Menuka) | 43.7 | 43.7 | 100 |
|  | 43.3 | 43.3 | 100 |
|  | 44.6 | 44.5 | 100.2 |
|  | 43.7 | 43.8 | 99.8 |
|  | 43.6 | 43.6 | 100 |
|  | 43 | 43.1 | 99.8 |
|  | 44.6 | 44.6 | 100 |
|  | 42.7 | 42.7 | 100 |
|  | 44.6 | 44.3 | 100.7 |
|  | 40.7 | 40.8 | 99.8 |
|  |  |  |  |
| **Worker ID** | **Worker's Measurement** | **Giri's Measurement (GS)** | **% Agreement** |
| 213 (Kiran) | 43.8 | 43.7 | 100.2 |
|  | 43.3 | 43.3 | 100 |
|  | 44.6 | 44.5 | 100.2 |
|  | 43.9 | 43.8 | 98.2 |
|  | 43.8 | 43.6 | 100.5 |
|  | 43.3 | 43.1 | 100.5 |
|  | 44.4 | 44.6 | 99.6 |
|  | 43 | 42.7 | 100.7 |
|  | 44.6 | 44.3 | 100.7 |
|  | 40.6 | 40.8 | 99.5 |
|  |  |  |  |
| **Worker ID** | **Worker's Measurement** | **Giri's Measurement (GS)** | **% Agreement** |
| 211 (Sulochana) | 43.7 | 43.7 | 100 |
|  | 43.3 | 43.3 | 100 |
|  | 44.6 | 44.5 | 100.2 |
|  | 43.8 | 43.8 | 100 |
|  | 43.6 | 43.6 | 100 |
|  | 43 | 43.1 | 99.8 |
|  | 44.4 | 44.6 | 99.6 |
|  | 42.8 | 42.7 | 100.2 |
|  | 44.4 | 44.3 | 100.2 |
|  | 40.7 | 40.8 | 99.8 |
|  |  |  |  |
| **Worker ID** | **Worker's Measurement** | **Giri's Measurement (GS)** | **% Agreement** |
| 204 (Ishwori) | 43.8 | 43.7 | 100.2 |
|  | 43.3 | 43.3 | 100 |
|  | 44.6 | 44.5 | 100.2 |
|  | 43.8 | 43.8 | 100 |
|  | 43.6 | 43.6 | 100 |
|  | 43 | 43.1 | 99.8 |
|  | 44.4 | 44.6 | 99.6 |
|  | 42.7 | 42.7 | 100 |
|  | 44.5 | 44.3 | 100.5 |
|  | 40.7 | 40.8 | 99.8 |
|  |  |  |  |
| **Worker ID** | **Worker's Measurement** | **Giri's Measurement (GS)** | **% Agreement** |
| 212 (Kesab) | 43.8 | 43.7 | 100.2 |
|  | 43.3 | 43.3 | 100 |
|  | 44.6 | 44.5 | 100.2 |
|  | 43.8 | 43.8 | 100 |
|  | 43.7 | 43.6 | 100.2 |
|  | 43.1 | 43.1 | 100 |
|  | 44.4 | 44.6 | 99.6 |
|  | 42.8 | 42.7 | 100.2 |
|  | 44.5 | 44.3 | 100.5 |
|  | 40.8 | 40.8 | 100 |
|  |  |  |  |
| **Worker ID** | **Worker's Measurement** | **Giri's Measurement (GS)** | **% Agreement** |
| 216 (Ram NR) | 43.8 | 43.7 | 100.2 |
|  | 43.3 | 43.3 | 100 |
|  | 44.5 | 44.5 | 100 |
|  | 43.6 | 43.8 | 99.5 |
|  | 43.7 | 43.6 | 100.2 |
|  | 43.2 | 43.1 | 100.2 |
|  | 44.4 | 44.6 | 99.6 |
|  | 42.7 | 42.7 | 100 |
|  | 44.6 | 44.3 | 100.7 |
|  | 40.5 | 40.8 | 99.3 |
|  |  |  |  |
| **Worker ID** | **Worker's Measurement** | **Giri's Measurement (GS)** | **% Agreement** |
| 215 (Nir BDR) | 43.8 | 43.7 | 100.2 |
|  | 43.2 | 43.3 | 99.8 |
|  | 44.5 | 44.5 | 100 |
|  | 43.6 | 43.8 | 99.5 |
|  | 43.7 | 43.6 | 100.2 |
|  | 43.1 | 43.1 | 100 |
|  | 44.4 | 44.6 | 99.6 |
|  | 42.9 | 42.7 | 100.5 |
|  | 44.5 | 44.3 | 100.5 |
|  | 40.5 | 40.8 | 99.3 |
|  |  |  |  |
| **Worker ID** | **Worker's Measurement** | **Giri's Measurement (GS)** | **% Agreement** |
| 207 (Ram Asesh) | 43.8 | 43.7 | 100.2 |
|  | 43.4 | 43.3 | 100.2 |
|  | 44.5 | 44.5 | 100 |
|  | 43.6 | 43.8 | 99.5 |
|  | 43.6 | 43.6 | 100 |
|  | 43.1 | 43.1 | 100 |
|  | 44.4 | 44.6 | 99.6 |
|  | 43 | 42.7 | 100.7 |
|  | 44.7 | 44.3 | 100.9 |
|  | 40.5 | 40.8 | 99.3 |
|  |  |  |  |
| **Worker ID** | **Worker's Measurement** | **Giri's Measurement (GS)** | **% Agreement** |
| 214 (Padam BDR) | 43.9 | 43.7 | 100.5 |
|  | 43.2 | 43.3 | 99.8 |
|  | 45.2 | 44.5 | 101.6 |
|  | 49.1` | 43.8 | 112.1 |
|  | 43.8 | 43.6 | 100.5 |
|  | 44.6 | 43.1 | 103.5 |
|  | 45.3 | 44.6 | 101.6 |
|  | 47.7 | 42.7 | 11.7 |
|  | 44.6 | 44.3 | 100.7 |
|  | 45.7 | 40.8 | 112 |
|  |  |  |  |
| **Worker ID** | **Worker's Measurement** | **Giri's Measurement (GS)** | **% Agreement** |
| 214 (Padam) | 44.8 | 44.7 | 100.2 |
| REDO | 46.1 | 46.1 | 100 |
|  | 45.2 | 45.1 | 100.2 |
|  | 44.1 | 44.2 | 99.8 |
|  | 42.4 | 42.5 | 99.8 |
|  | 45.6 | 45.7 | 99.8 |
|  | 43.3 | 43.2 | 100.2 |
|  | 44 | 44 | 100 |
|  | 44.3 | 44.4 | 99.8 |
|  | 44.1 | 44.1 | 100 |
|  |  |  |  |
| **Worker ID** | **Worker's Measurement** | **Giri's Measurement (GS)** | **% Agreement** |
| 202 (Dipak) | 43.8 | 43.9 | 99.8 |
|  | 43.2 | 43.3 | 99.8 |
|  | 45.4 | 45.3 | 100.2 |
|  | 49.3 | 49.1 | 100.4 |
|  | 43.7 | 43.8 | 99.8 |
|  | 44.5 | 44.6 | 99.8 |
|  | 45.3 | 45.2 | 100.2 |
|  | 43.5 | 43.7 | 99.5 |
|  | 44.6 | 44.5 | 100.2 |
|  | 45.7 | 45.8 | 99.8 |
|  |  |  |  |
| **Worker ID** | **Worker's Measurement** | **Giri's Measurement (GS)** | **% Agreement** |
| 209 (Khadga) | 44 | 43.9 | 100.2 |
|  | 43.2 | 43.3 | 99.8 |
|  | 45.2 | 45.3 | 99.8 |
|  | 49 | 49.1 | 99.8 |
|  | 43.7 | 43.8 | 99.8 |
|  | 44.7 | 44.6 | 100.2 |
|  | 45.2 | 45.2 | 100 |
|  | 43.7 | 43.7 | 100 |
|  | 44.5 | 44.5 | 100 |
|  | 45.7 | 45.8 | 99.8 |
|  |  |  |  |
| **Worker ID** | **Worker's Measurement** | **Giri's Measurement (GS)** | **% Agreement** |
| 210 (Shyam) | 43.9 | 43.9 | 100 |
|  | 43.2 | 43.3 | 99.8 |
|  | 45.2 | 45.3 | 99.8 |
|  | 49.2 | 49.1 | 100.2 |
|  | 43.8 | 43.8 | 100 |
|  | 44.6 | 44.6 | 100 |
|  | 45.2 | 45.2 | 100 |
|  | 43.7 | 43.7 | 100 |
|  | 44.6 | 44.5 | 100.2 |
|  | 45.8 | 45.8 | 100 |
|  |  |  |  |
| **Worker ID** | **Worker's Measurement** | **Giri's Measurement (GS)** | **% Agreement** |
| 205 (Yadav) | 43.9 | 43.9 | 100 |
|  | 43.3 | 43.3 | 100 |
|  | 45.4 | 45.3 | 100.2 |
|  | 49.1 | 49.1 | 100 |
|  | 43.9 | 43.8 | 100.2 |
|  | 44.5 | 44.6 | 99.8 |
|  | 45.2 | 45.2 | 100 |
|  | 43.8 | 43.7 | 100.2 |
|  | 44.6 | 44.5 | 100.2 |
|  | 45.7 | 45.8 | 99.8 |
|  |  |  |  |
| **Worker ID** | **Worker's Measurement** | **Giri's Measurement (GS)** | **% Agreement** |
| 203 (Roshan) | 43.8 | 43.9 | 99.8 |
|  | 43.4 | 43.3 | 100.2 |
|  | 45.2 | 45.3 | 99.8 |
|  | 49.2 | 49.1 | 100.2 |
|  | 43.8 | 43.8 | 100 |
|  | 44.6 | 44.6 | 100 |
|  | 45.3 | 45.2 | 100.2 |
|  | 43.6 | 43.7 | 99.8 |
|  | 44.6 | 44.5 | 100.2 |
|  | 45.8 | 45.8 | 100 |
|  |  |  |  |
| **Worker ID** | **Worker's Measurement** | **Giri's Measurement (GS)** | **% Agreement** |
| 206 (Rudra) | 43.9 | 43.9 | 100 |
|  | 43.2 | 43.3 | 99.8 |
|  | 44.9 | 45.3 | 99.1 |
|  | 49.1 | 49.1 | 100 |
|  | 43.7 | 43.8 | 99.8 |
|  | 44.6 | 44.6 | 100 |
|  | 45.4 | 45.2 | 100.4 |
|  | 43.7 | 43.7 | 100 |
|  | 44.6 | 44.5 | 100.2 |
|  | 45.8 | 45.8 | 100 |

J. A-not-B Task training sheets

|  | **12 Month Child:** | |  |  |  |  |  | **10 Month Child:** | |  |  |
| --- | --- | --- | --- | --- | --- | --- | --- | --- | --- | --- | --- |
|  | **Left Well** | **Right Well** |  | **Delay** | **Notes** |  |  | **Left Well** | **Right Well** |  | **Delay** |
| 1 | **x** |  |  | **5 sec** |  |  | 1 | **1** |  |  | **3 sec.** |
| 2 | **1** |  |  | **3 sec** |  |  | 2 | **1** |  |  |  |
| 3 | **x SC** |  |  |  | **SC Noted** |  | 3 |  | **x** |  |  |
| 4 | **1** |  |  | **1 sec** |  |  | 4 |  | **1** |  |  |
| 5 | **1** |  |  |  |  |  | 5 |  | **x** |  |  |
| 6 |  | **1** |  |  |  |  | 6 |  | **1** |  |  |
| 7 |  | **1** |  |  |  |  | 7 |  | **1** |  |  |
| 8 |  | **1** |  |  |  |  | 8 | **1** |  |  |  |
| 9 | **1** |  |  |  |  |  | 9 | **1** |  |  |  |
| 10 | **1** |  |  |  |  |  | 10 | **1** |  |  |  |
| 11 | **x NR** |  |  |  |  |  | 11 |  | **1** |  |  |
| 12 | **1** |  |  |  |  |  | 12 |  | **x** |  |  |
| 13 | **1** |  |  |  |  |  | 13 |  | **1** |  |  |
| 14 |  | **1** |  |  |  |  | 14 |  | **1** |  |  |
| 15 |  | **1** |  |  |  |  | 15 | **1** |  |  |  |
| 16 |  | **1** |  |  |  |  | 16 | **1** |  |  |  |
|  |  |  |  |  |  |  | 17 | **1** |  |  |  |
|  |  |  |  |  |  |  | 18 |  | **1** |  |  |
|  |  |  |  |  |  |  | 19 |  | **1** |  |  |
|  |  |  |  |  |  |  | 20 |  | **1** |  |  |
|  |  |  |  |  |  |  |  |  |  |  |  |
|  | **9.5 Month Child:** | |  |  |  |  |  | **10 Month Child:** | |  |  |
|  | **Left Well** |  |  | **Delay** | **Notes** |  |  | **Left Well** | **Right Well** |  | **Delay** |
| 1 | **1** |  |  | **3 sec** |  |  | 1 | **x** |  |  | **3 sec** |
| 2 | **1** |  |  |  |  |  | 2 | **1** |  |  | **1 sec** |
| 3 |  | **1** |  |  |  |  | 3 | **1** |  |  |  |
| 4 |  | **x SC** |  | **5 sec** | **SC Noted** |  | 4 |  | **1** |  |  |
| 5 |  | **1** |  | **3sec** |  |  | 5 |  | **1** |  | **3 sec** |
| 6 |  | **1** |  |  |  |  | 6 |  | **1** |  |  |
| 7 | **1** |  |  |  |  |  | 7 | **x** |  |  |  |
| 8 | **1** |  |  |  |  |  | 8 | **1** |  |  |  |
| 9 | **x PR** |  |  |  | **PR Noted** |  | 9 | **1** |  |  |  |
| 10 | **1** |  |  |  |  |  | 10 | **1** |  |  |  |
| 11 | **1** |  |  |  |  |  | 11 |  | **1** |  |  |
| 12 |  | **1** |  |  |  |  | 12 |  | **1** |  |  |
| 13 |  | **1** |  |  |  |  | 13 |  | **1** |  |  |
| 14 |  | **x NR** |  |  | **NR Noted** |  | 14 | **x** |  |  |  |
| 15 |  | **1** |  |  |  |  | 15 | **1** |  |  |  |
| 16 |  | **1** |  |  |  |  | 16 | **1** |  |  |  |
| 17 | **1** |  |  |  |  |  | 17 | **1** |  |  |  |
| 18 | **1** |  |  |  |  |  | 18 |  | **1** |  |  |
| 19 | **1** |  |  |  |  |  | 19 |  | **1** |  |  |
|  |  |  |  |  |  |  | 20 |  | **1** |  |  |

|  | **12 Month Child:** | |  |  |  |  |  | **9 Month Child:** | |  |  |
| --- | --- | --- | --- | --- | --- | --- | --- | --- | --- | --- | --- |
|  | **Left Well** | **Right Well** |  | **Delay** | **Notes** |  |  | **Left Well** | **Right Well** |  | **Delay** |
| 1 | **1** |  |  | **3 sec** |  |  | 1 | **1** |  |  | **3 sec** |
| 2 | **x** |  |  |  |  |  | 2 | **x** |  |  |  |
| 3 | **1** |  |  | **1 sec** |  |  | 3 | **1** |  |  | **1 sec** |
| 4 | **x SC** |  |  |  | **SC Noted** |  | 4 | **1** |  |  |  |
| 5 | **1** |  |  | **0 sec** |  |  | 5 |  | **x** |  |  |
| 6 | **1** |  |  |  |  |  | 6 |  | **1** |  |  |
| 7 |  | **x** |  |  |  |  | 7 |  | **1** |  |  |
| 8 |  | **x** |  |  |  |  | 8 |  | **1** |  |  |
| 9 |  | **x** |  |  |  |  | 9 | **1** |  |  |  |
| 10 |  | **x** |  |  |  |  | 10 | **1** |  |  |  |
| 11 |  | **x** |  |  |  |  | 11 | **1** |  |  |  |
| 12 |  | **1** |  |  | **Used 1 box Noted** |  | 12 |  | **1** |  |  |
| 13 |  | **1** |  |  |  |  | 13 |  | **x** |  |  |
| 14 |  | **1** |  |  |  |  | 14 |  | **1** |  |  |
| 15 | **1** |  |  |  |  |  | 15 |  | **1** |  |  |
| 16 | **1** |  |  |  |  |  | 16 | **1** |  |  |  |
| 17 | **1** |  |  |  |  |  | 17 | **1** |  |  |  |
| 18 |  | **1** |  |  |  |  | 18 | **1** |  |  |  |
| 19 |  | **1** |  |  |  |  |  |  |  |  |  |
| 20 |  | **1** |  |  |  |  |  |  |  |  |  |
|  |  |  |  |  |  |  |  |  |  |  |  |
|  | **10 Month Child:** | |  |  |  |  |  | **11 Month Child:** | |  |  |
|  | **Left Well** | **Right Well** |  | **Delay** |  |  |  | **Left Well** | **Right Well** |  | **Delay** |
| 1 | **1** |  |  | **3 sec** |  |  | 1 | **1** |  |  | **5 sec** |
| 2 | **1** |  |  |  |  |  | 2 | **1** |  |  |  |
| 3 |  | **1** |  |  |  |  | 3 |  | **1** |  |  |
| 4 |  | **1** |  | **5 sec** |  |  | 4 |  | **1** |  | **8 sec** |
| 5 |  | **1** |  |  |  |  | 5 |  | **1** |  |  |
| 6 | **x** |  |  |  |  |  | 6 | **1** |  |  |  |
| 7 | **x** |  |  |  |  |  | 7 | **1** |  |  | **10 sec** |
| 8 | **1** |  |  |  |  |  | 8 | **1** |  |  |  |
| 9 | **1** |  |  |  |  |  | 9 |  | **1** |  |  |
| 10 | **1** |  |  |  |  |  | 10 |  | **1** |  |  |
| 11 |  | **1** |  |  |  |  | 11 |  | **1** |  |  |
| 12 |  | **1** |  |  |  |  | 12 | **1** |  |  |  |
| 13 |  | **1** |  |  |  |  | 13 | **1** |  |  |  |
| 14 | **1** |  |  |  |  |  | 14 | **1** |  |  |  |
| 15 | **1** |  |  |  |  |  | 15 |  | **1** |  |  |
| 16 | **1** |  |  |  |  |  | 16 |  | **1** |  |  |
| 17 |  | **1** |  |  |  |  | 17 |  | **1** |  |  |
| 18 |  | **1** |  |  |  |  |  |  |  |  |  |
| 19 |  | **1** |  |  |  |  |  |  |  |  |  |

**K. Formative research documents**

**Parental Report Scales**

This scale appears to have performed well. We could place question 15 before 14 and 16. This would mean throwing a ball overhand would come before walking up stairs. Questions 20 and 21 could also be switched, meaning walking backwards would come before standing on one foot. For the most part though, the data look good.

This scale might perform better if we were to change the order of some of the questions. Question number 1 appears out of place. Maybe it (babbles) would be better after question 2 (whines or whimpers). Also, question numbers 8-22 might be better if their present order were altered: 10,11,9,13,14,8,12,15,17,16,22,18… It appears that in Nepal children learn how to respond to vocalizations (gives me something when I ask for it, point to a dog, etc.) before they begin to initiate them through vocalization (says 1 word, 3 words) or gesture (does Namaste gesture).

**Temperament Scale**

| **Temperament Scale Formative Research: Summer 2001** | | | | | |
| --- | --- | --- | --- | --- | --- |
| **Overall (n=46)** | | | | | |
| **Questions** |  | **1(Easy)** | **2(Average)** | **3(Difficult)** | **Chi-square** |
| 1 | cry loudly | 6 (18.8) | 12 (37.5) | 14 (43.8) | 0.331 |
| 2 | mood | 5 (27.8) | 5 (27.5) | 8 (44.4) | 0.208 |
| 3 | calm | 5 (17.2) | 17 (58.6) | 7 (24.1) | 0.02* |
| 4 | sleep | 2 (14.3) | 8 (57.1) | 4 (28.6) | 0.464 |
| 5 | fussy | 2 (13.3) | 5 (33.3) | 8 (53.3) | 0.275 |
| 6 | cry more | 2 (22.2) | 1 (11.1) | 6 (66.7) | 0.067* |
| 7 | upset | 1 (16.7) | 1 (16.7) | 4 (66.7) | 0.244 |
| 8 | excited | 7 (20.6) | 16 (47.1) | 11 (32.4) | 0.548 |
| 9 | more attention | 3 (12.0) | 9 (36.0) | 13 (52.0) | 0.059* |
| 10 | play by self | 6 (20.0) | 16 (53.3) | 8 (26.7) | 0.11 |
| 11 | persistent | 3 (11.5) | 8 (30.8) | 15 (57.7) | 0.004* |
|  |  |  |  |  |  |
| Overall (N=20) - Revised Question #'s 1 and 2 | | | | | |
| **Questions** |  | **1 (Easy)** | **2 (Average)** | **3 (Difficult)** | **Chi-square** |
| 1 | cry loudly | 0 | 11 (78.6) | 3 (21.4) | 0.012 |
| 2 | mood | 0 | 3 (100) | 0 | 0.469 |
| 3 | calm | 3 (18.8) | 11 (68.8) | 2 (12.5) | 0.577 |
| 4 | sleep | 1 (33.3) | 2 (66.7) | 0 | 0.515 |
| 5 | fussy | 0 | 2 (50) | 2 (50) | 0.077 |
| 6 | cry more | 0 | 3 (50) | 3 (50) | 0.012 |
| 7 | upset | 0 | 0 | 0 |  |
| 8 | excited | 3 (17.6) | 11 (64.7) | 3 (17.6) | 0.469 |
| 9 | more attention | 0 | 4 (57.1) | 3 (42.9) | 0.024 |
| 10 | play by self | 3 (16.7) | 13 (72.2) | 2 (11.1) | 0.321 |
| 11 | persistent | 0 | 5 (62.5) | 3 (37.5) | 0.037 |

Appetite Scale

| Appetite Scale Formative Research Spring 2001 | | | | | |
| --- | --- | --- | --- | --- | --- |
| Overall (N =36) | | | | | |
|  |  | Overall Appetite | | |  |
| Questions | Code | 1(good) | 2 (so-so) | 3 (poor) | Chi-square |
| 1 | eatalot | 8 (80.0) | 2 (20.0) | 0 | 0.063 |
| 2 | happy | 17 (63.0) | 9 (33.3) | 1 (3.7) | 0 |
| 3 | asksfood | 12 (63.2) | 6 (31.6) | 1 (5.3) | 0.102 |
| 4 | fusses | 4 (33.3) | 3 (25.0) | 5 (41.7) | 0.017 |
| 5 | refuses | 7 (46.7) | 3 (20.0) | 5 (33.3) | 0.079 |
| 6 | snitches | 10 (58.8) | 6 (35.3) | 1 (5.9) | 0.205 |
| 7 | eatsfreq | 11 (78.6) | 3 (21.4) | 0 | 0.014 |
| 8 | picky | 5 (38.5) | 5 (38.5) | 3 (23.1) | 0.548 |
| 9 | sick | 7 (50.0) | 5 (35.7) | 2 (14.3) | 0.943 |

**L. Letters submitted to the Nepal NHRC and the Johns Hopkins CHR**

December 15, 2000

The Chairman

Nepal Health Research Council

Kathmandu

Nepal

RE: Addendum to research proposal: Impact of Zinc and Iron/Folate Supplementation on Child Mortality (NNIPS-4).

Dear Sir:

We are submitting for review to the NHRC the following addendum to the above protocol, which has already been approved by the NHRC. The title of the addendum is: Impact of Zinc and Iron/Folate Supplementation on the Development of Young Children.

# Background

In recent years, iron and zinc have received increased attention due to their important role in the cognitive and motor development of children. Iron deficiency anemia is associated with comparatively low performance in mental and motor development scales among infants and toddlers. Zinc deficiency has been implicated in decreased motor development, activity, and cognitive functioning: Neurobiological evidence suggests that these are causal associations. However, evidence from randomized trials is insufficient to determine whether iron or zinc supplementation of young children at risk for iron and zinc deficiency and developmental delays can prevent and/or correct these abnormalities.

Studies have also shown that iron and zinc supplementation increase the motor activity of previously deficient toddlers. Motor milestones such as crawling, creeping and walking and early motor activity play critical roles in the acquisition and organization of basic cognitive functions such as intersensory organization and visual spatial perception. Language acquisition during the first 24 months of life includes several interrelated functional changes. These range from the first signs of speech and word comprehension to early grammatical development, key cognitive functions that lie at the roots of interpersonal communication.

Early child development is integral to a child’s well-being. Poverty puts children at developmental risk for a variety of reasons, including poor health and nutrition. We will test whether 2 relatively low-cost health interventions can improve the development of children in rural Nepal.

# Overview and Specific Aims

This study will be implemented as a sub-study of the larger randomized 2x2 factorial trial of zinc and/or iron-folate supplementation with child mortality as the outcome. A sub-sample consisting of 750 children 6-18 months of age will be enrolled in the proposed sub-study. The overall goal of this project is to describe and document the role of zinc and iron supplementation in the development of infants and toddlers living in southern Nepal.

Primary Aim: To measure whether zinc or iron-folate supplementation provided over a 12-month period to Nepali children 6-18 months of age improves language development, motor development, and motor activity, or affects irritability.

Secondary Aim: To describe the development of rural Nepali children from 6-18 months using both scientific and local socio-cultural perspectives.

# Study Population

The study population for this project will consist of 750 children 6-18 months of age living in 3 of the 30 Village Development Committees (VDCs) households in the NNIPS catchment area in Sarlahi District of southern Nepal. At the beginning of the mortality trial, a baseline census of the study area will be conducted to enumerate the population and determine which households have children eligible to participate in the study. Households in the 3 subsample VDCs with age-eligible children will be invited to participate in the child development sub-study. The purpose of the study will be explained, a disclosure statement will be read and verbal consent will be obtained.

Data Collection

Study children will be assessed at baseline and every 3 months over the 12 months of follow-up. At each time-point, an interview with the parents of the child will be conducted. Caregivers will be asked to answer questions about their child’s motor and language development and temperament (irritability). Activity over a 24-hour period will be measured using a small watch-like device that records movement. Additionally, children will be assessed in the clinic with the Bayley Scales of Infant Development II, a standard developmental test, at baseline, 6 months, and 12 months following enrollment.

I would be happy to answer any questions about this addendum. Please feel free to contact me via the NNIPS office or at Johns Hopkins University in Baltimore.

Sincerely,

James M. Tielsch, Ph.D.

Project Scientist

Memorandum

To: Committee on Human Research

School of Hygiene and Public Health

From: James Tielsch, Ph.D.

Re: Amend CHR # H.22.99.06.01.A

“Impact of Zinc Supplementation on Child Mortality”

Date: February 9, 2001

We would like to amend “Impact of Zinc Supplementation on Child Mortality” to include an additional research question, methods to address this question, and a student researcher, Emily Siegel, who will be using the data for her dissertation.

Research Question

This sub-study is designed to examine the impact of zinc and iron/folate supplementation on the cognitive development of young children living in Sarlahi District, Nepal.

Rationale

The first few years of life are critical for physical growth and development. During this period, infants achieve major milestones, such as crawling and walking, and begin the process of socialization. They use gestures and vocalizations to interact with their primary caregivers and to satisfy their basic needs. Their burgeoning ability to sense and perceive helps them to process the stimuli present in their surrounding environment, which facilitates exploration and learning. It is during these first few years that the infants’ temperament or personality begins to emerge, punctuating their individual differences. When the environment is not optimal and detrimental influences, such as malnutrition, or more specifically micronutrient deficiencies, occur, infants have been found to experience decreased cognitive functioning, motor development, and activity.

Methods

The sub-study population will consist of 700 children 6- to 18-months of age that will be selected from sectors in close proximity to the field office in the study area in southern Nepal. These children will be the same children selected to be a part of the growth and morbidity sub-sample outlined in the original CHR proposal. The children will be followed for a year. Their development will be assessed both in the home and in a central site located near their homes. The majority of the measures will be administered every 6-months (baseline, 6-, and 12-months); however, the parental report and the temperament scale will be given every three months (baseline, 3-, 6-, 9-, and 12-months).

In the home, fieldworkers will administer two questionnaires to the primary caregivers. The first is a parental report scale that asks questions about the motor, social, and emotional development of the children. The second is a temperament scale that focuses on the fussiness and irritability of the child. Fieldworkers will use a watch-like device called an Actiwatch to measure each child’s activity. The Actiwatch will be strapped on to the left ankle of the target child for a period of 22-hours. At the end of the 22-hour period, fieldworkers will observe for a period of 4 hours the interaction of the primary caregiver and the target child. Specifically, they will record events that include motor actions, social behavior, non-direct social behavior, and directions; such as to whom the child is looking or traveling.

In the central site, fieldworkers will administer The Bayley Scales of Infant Development II, a developmental test used to measure motor and mental development and behavior in young children. For this test, the infants will be seated at a table (the younger ones will sit on their caregiver’s lap) and will be asked to perform tasks, such as stacking and placing blocks into a cup. Once the table items have been administered, the tester will direct the child to the floor where he will assess the infants’ ability to perform motor actions. At the end of the test, the tester will discuss with the caregiver whether the infant’s behavior during the test was typical of behavior observed at home.

# Table 1. Summary of data to be collected

| *Primary Outcomes* | *Measures* | *Timepoints* |
| --- | --- | --- |
| Motor Development | Parental Report  Bayley Scales of Infant Dev’t II  Observation | 0, 3, 6, 9, 12  0, 6, 12  0, 6, 12 |
| Language Development | Parental Report  Bayley Scales of Infant Dev’t II  Observation | 0, 3, 6, 9, 12  0, 6, 12  0, 6, 12 |
| Temperament | Parental Report  Temperament Scale | 0, 3, 6, 9, 12  0, 3, 6, 9, 12 |
| Activity | Actiwatch  Observation | 0, 6, 12  0, 6, 12 |
| Social Behavioral Interaction | Observation | 0, 6, 12 |

Risks

Other than the additional time involved on the part of the participants, which we estimate to be a total of 6 hours in the clinic (2 hours x 3 visits) and 17 hours in the home (1 hour x 5 visits for the questionnaires and 4 hours x 3 visits for the observations), there will be no additional risks associated with participation in this sub-study. To protect the confidentiality of these data, we will use techniques described in the original application.

The Johns Hopkins University

School of Hygiene and Public Health

Committee on Human Research

Consent Form

Nepal Nutrition Intervention Project-Sarlahi

Namaste! I am a NNIPS staff person. You agreed to enroll your child in the zinc and iron tablet research project. We believe these tablets may improve children’s health, make their blood stronger in these nutrients and make them grow and develop better. Your child is invited to participate in an additional part of the study we are conducting to see if this is true.

If you decide to take part, we will visit your house five times and transport you to our study office at the bazaar three times. At the home visits, a NNIPS staff person will ask you a few questions about the health of your child, your child’s development, and his/her level of fussiness. This visit will take approximately 1 hour. A different staff person will come to your house three times during the next year to observe you and your child for a period of four hours, while you go about your regular business. In the office, a staff person will measure your child’s height, weight, and upper arm circumference, will collect 3 milliliters of blood from your child’s arm to test the level of zinc and iron in his/her blood, and will test your child’s development. This visit will take about 2 hours. We will take you home after the visit.

There are only very small risks for your child. Collecting blood may cause a small amount of pain and discomfort, but we will do it as quickly as possible and we will be sure to clean the area on his/her arm very well. Your child’s participation is completely voluntary at all times. If for any reason, you do not want your child to continue with the visits for this study, you may withdraw them at any time. Whether you choose to participate will not affect them being offered the tonic from the other study. All information you provide will remain confidential and your name or other identifying information will not be revealed to anyone who is not helping on this study. If you have any questions or problems regarding this study, I can answer them now or you can contact Dr. Subarna Khatry, NNIPS office, Hariaun, Sarlahi (phone no. 29404).

Memorandum

To: Committee on Human Research

School of Hygiene and Public Health

From: James Tielsch, Ph.D.

Re: Amend CHR # H.22.99.06.01.A

“Impact of Zinc Supplementation on Child Mortality”

Date: September 9, 2001

We would like to amend “Impact of Zinc Supplementation on Child Mortality” to include an additional clinic visit that will occur within two weeks of the sub-study infants’ 9-month birthday.

Research Question

This sub-study is designed to examine the impact of zinc and iron/folate supplementation on the cognitive development of young children living in Sarlahi District, Nepal.

Rationale

We propose to include this additional visit for two reasons:

1. Both of the developmental tests we propose to use recommend taking repeated measures at different time-points.
2. Having an additional data point will make the data stronger and will possibly yield more information on the effect of length of supplementation, since the children who participate in Clinic B will have received varying periods of supplementation prior to their clinic visit. Because this visit is dependent on the children’s birthdays, the period of supplementation will range from 0 to 9-months.

Methods

The methods that we propose to use for this visit will be the same those we have previously outlined for the 12-month Clinic B visit. These include The Fagan Test of Infant Intelligence and the A-not-B Test of Executive Processing. Combined the tests should not take more than 50 minutes. Of the 700 young children we anticipate will participate in the child development sub-study (called DIMAG: Development of Infant Motor skills, Articulation, and Growth), 324 will be eligible for the 9-month Clinic B visit. This means we will test 14 additional 9-month children each week for 6 months. During this period a total of 28 young children 9- and 12-months of age will be brought to the office. For the following 3 months, 14 young children 12-months of age will be tested weekly. The Clinic B visits will end after 9 months, at which time training for the Clinic C visit will begin.

In sum, we plan to visit each of our study participants in their homes five times throughout the course of the study. These visits will occur at baseline, 3-, 6-, 9-, and 12- months. We will bring everyone into the office twice for the Clinic A visit at baseline and 12-months and once for the Clinic C visit at 12-months. Children who are eligible for Clinic B (a total of 486 young children) will be brought into the office one or two additional times, depending on their age at enrollment. The maximum number of time-points that we could possibly see a single child is 10 over the course of 15-months.

October 2001 amendment to the CHR embedded within another NNIPS-4 amendment:

DIMAG Sub-study

We have named our developmental sub-study, DIMAG (Development of Infant Motor skills, Articulations, and Growth) which means, “mind” in Nepali.

DIMAG infants will be 4- to 16-months instead of 6- to 18-months at enrollment. We reduced the age of enrollment to allow us to administer the cognitive tests to a greater number of infants. Both the Fagan and A-not-B Tests cannot be administered to infants greater than 12-months.

The home visits including the Actiwatch attachment and retrieval, the observation, and the Home Interview will occur every 3-months instead of every 6-months.

The Home Interview will include scales that address the following topics: morbidity, infant feeding, appetite, motor and language development, and temperament. We have added the morbidity, infant feeding and appetite sections and have removed the emotional development section.

The observation has been reduced from 4 to 3 hours. It will focus on the target child’s activity, social behavior, and vocalizations.

To distinguish between our office visits, we have named them Clinic A, B, and C. For each of these clinic visits the same morbidity questions that will be included in the Home Interview will be asked to assess the child’s health on the day of the clinic visit. Clinic A will occur at baseline and after 12-months of supplementation. During this visit our lab team will assess anthropometry (recumbent length, weight, mid-upper arm circumference (MUAC), head circumference, and tricep skinfold thickness) and will do phlebotomy. Three milliliters of blood will be drawn from each child through venipuncture and will be used to assess the following: serum zinc, serum ferritin, hemoglobin, erythrocyte protoporphrin, and transferrin receptor. The Clinic A visit is not expected to take longer than 45 minutes.

For Clinic B our lab personnel will administer two measures of cognitive development. These include the Fagan Test of Infant Intelligence and the A-not-B Task. The Fagan Test measures visual recognition memory through the presentation of pictures of faces on a wooden stage. The child will not be able to observer the tester behind the stage as the observer records the child’s eye movements into a computer. The A-not-B Task, a measure of pre-frontal cortex functioning, requires the child to search for a toy that was hidden by the tester in one of two wells in a table directly in front of the child. Infants will be eligible for Clinic B when they turn 39 and 52 weeks of age. Only infants less than or equal to 39 weeks at the beginning of the trial will be eligible for both Clinic B visits. Infants greater than 52 weeks at the start of the trial will not be eligible for either Clinic B visit. Based on our assumption that DIMAG will enroll about 700 infants 4- to 16-months of age, we expect that about 486 infants will be eligible for the 52-week visit. Of these infants, we expect that about 378 will also be eligible for the 39-week visit. Each of the Clinic B visits is expected to take no more than 1 hour.

Clinic C will occur after the infants have received 12-months of supplementation. During this visit the Bayley Test of Infant Development will be administered. Since the Bayley Test is described in the February 9, 2001 amendment, it will not be described here. This visit is expected to take from one-and-a-half to two hours.

In sum, we plan to visit each of the infants enrolled in DIMAG in their homes five times. We will do the following while we are there: attach and remove the Actiwatch, observe the infants’ activity, social behavior, and vocalizations for a period of three hours, and administer the Home Interview, which asks questions about morbidity, infant feeding, appetite, motor and language development, and temperament.

We will bring each infant to the office between three and five times, depending on the age of the child. We will bring each infant to the office at baseline and after 12-months of supplementation to be measured and have blood drawn for Clinic A. After 12-month of supplementation each of the infants will participate in Clinic C where they will perform the Bayley Scales of Infant Development. All infants who turn 39- and 52-weeks during the trial will be brought into the lab on these “birthdays” for Clinic B, where both the Fagan Test of Infant Development and the A-not-B Task will be administered. Every infant will be transported to the office for these clinic visits.

# Activities in Ishwarpur

In addition to DIMAG, there will be two activities that will also occur in Ishwarpur VDC. The first is the administration of the Motor Milestones Checklist to all children enrolled in NNIPS 4 who are living in Ishwarpur. This is a prospective tool that is designed to capture the exact week in which young children master each of the 14 motor milestones from pulling to a seated position to standing on one foot. The ward distributors will record the dates when they visit weekly each of the families enrolled in the larger mortality trial. The checklist will be administered to every child throughout the duration of NNIPS 4.

The second activity will be a heal prick of all 1-month old infants enrolled in NNIPS 4 that will occur at baseline and a month after the study begins. The objective of this sub-sample is to measure the levels of zinc and iron/folate in our youngest children’s blood a month after supplementation begins. Only children from Ishwarpur will be eligible for this procedure. All of these children will be transported to and from our clinic with their caregivers at the time of baseline and 1-month post-supplementation.

**M. CAOS User Guide**

# CAOS

Child Activity Observation Software

# Base Module User’s Guide (DIMAG: Nepal)

Darrell Mast

Department of International Health

Johns Hopkins University

Draft – 01/08/2002

Chapter 1: Software Overview

###### General Functions

CAOSBase software provides the focal point for configuring CAOS palm devices for use in observations. It serves as the repository for data about the children being observed, the observers and the codes being utilized. It facilitates the distribution of CAOS software and data to the palm devices as wells the download, storage and access to observation data collected through the CAOS software on the Palm devices. In addition, it provides the capability to incorporate Actiwatch data with a Palm observation.

###### Getting Started

CAOSBase software requires the following:

1. A computer capable of running Windows 98 or higher.
2. A USB port.
3. A USB cradle for Hotsyncing palm devices.
4. A serial reader (for Actiwatch data only)
5. Microsoft Office (for additional database functionality)
6. Palm Desktop Software

Installation routines are provided separately. Once software is installed, each palm should be identified to the system, using the palm desktop software. Then use CAOSBase to update all palm devices with the full suite of CAOS functionality. This can be found under File, Palm, Update Palm Installation from the main menu. As each palm device is Hotsynced, the CAOS software will be installed and you are ready to begin observations.

###### Data Organization

In CAOS data is staged as it passes from the Palm devices to the Database. When a Palm is hotsynced, data is passed from the Palm to the Palm Backup directory for that device. In the download process, the data is copied from the Palm Backup directory to the CAOS\Data directory for storage and later utilization. Additionally the data is loaded into an Access database for viewing and analytical purposes.

Actiwatch data is downloaded using a serial reader and the Actiware Rhythm software (outside the scope of CAOSBase). Data files from this process, can be loaded into the CAOS database structure for use in comparing ActiWatch data with Palm Observations.

**Chapter 2: Main Menu Functions**


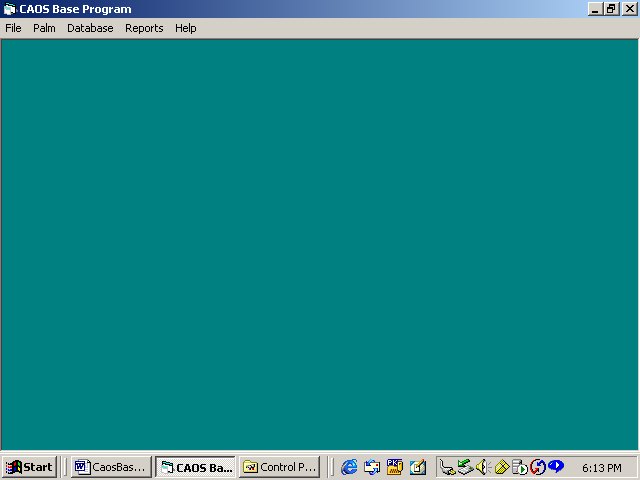


Menu Selections:

File: Functions for Importing data in and out of the CAOSBase database structure

Import ActiWatch Data:

Utility to load data from an ActiWatch file into the database.

Import Palm Data

Utility to load data from a palm file into the database. This will not normally be necessary as this automatically occurs during the download process.

Export:

Not implemented but future functionality to select data from the database for use in other applications such as excel for further analysis.

Exit:

Exit the CAOSBase software.

Palm Functions for interacting with the Palm devices.

Download Palm Data Steps you through the process of downloading observation data from the Palm to the Base Station. First the palm is hotsynced to retrieve the data, then the data is stored in C:\CAOS\Data, and the database. The observation files for the palm are refreshed for the next observation and the palm device is hotsynced again to receive the refreshed files.

Update Palm Installation Provides functions for updating the CAOS software or files on a particular palm device or all palm devices defined to the Base Station. Individual files may be selected or all files may be chosen if a palm device needs to be reinstalled.

View Palm Files Provides a utility to view information on the palm as of the last Hotsync operation. It is not envisioned this utility will be used on a routine basis.

Database Functions for interacting with the CAOSBase Database structure

View Allows you to view the information stored on the database.

Edit Provides functions for editing the Base Tables. You may add or update, child records, observer records, and code records and install them to the palm devices after updates are complete.

Reports Functions for viewing observations. Observations may be viewed in Detail or Summary format. You may also compare two observations by different observers watching the same child at the same time and view watch data along side a palm observation.

Help Provides information about the CAOSBase software. Future versions may include more detailed help information.

###### Chapter 3: Importing Watch Data

**Utilizing Actiware Rhythm**

**Uploading Actiwatch data to CAOSBase**

Chapter 4: Palm Functions

**Downloading Palm Data**


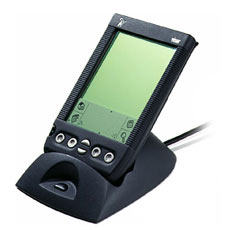


Hotsync Cradle

###### Updating a Palm Device

###### Viewing Palm Data Utility

Chapter 5: Database Functions

###### Database Overview

###### Viewing Database Information

###### Updating Database Information

Chapter 6: Reports

###### Selecting a Report

######

###### Viewing a Report

Chapter 7: Other Operations

**Loading the Child Data Table**

The following steps are needed to update the CAOS child table from the main database.

1. In SQLServer Enterprise manager, select the NNIPS4 database. Then select tools, Data Transformation Services, Export Data.
2. Export to a file named childload.txt (important).
3. Use a query (select * from dimagload) and a fixed field format. Fields in the following order (should be the default) Location, childid, firstname, lastname, dob, sexcd, motherfirstname, motherlastname, fatherfirstname, fatherlastname.
4. Copy the file to the CAOS Base Station it must be placed in the C: drive.
5. Use Access to load the CAOSBaseDB.mdb file. Select the pChild table. Under Edit, select all records. Press delete. Confirm the delete operation Close Access.
6. Start CAOSBase. Under file, select Update Child Table from NNIPS.
7. In CAOSBASE, under Database, select Edit. Select Table Children, then Create Palm File.
8. On next hotsync, the palm devices will load the new child information.
9. Repeat steps 4-6 on backup base station.

###### Configuring a New Palm

###### Rebuilding a Palm Installation of CAOS

**Export**

The Export Function is not yet available.

Chapter 8: CAOSBase File Structures

**Overview**

This chapter highlights the location of program and data files utilized by the CAOSBase program. The user should take special note of locations of data files, which is significant in backup procedures.

**CAOSBase Program Files**

Files are located in the Following Directory:

C:\PROGRAM FILES\CAOSBASE\

CAOSBASE.exe The CAOSBase Application. This file will be updated as modifications to the application occur.

### Palm Program Files

Files are located in the following Directory:

C:\PROGRAM FILES\PALM

Each Palm that has been defined to the Base Station will have associated subdirectory under the main Palm directory with the name of the Palm as the subdirectory name.

Under the subdirectory for each palm are 2 significant directories:

\Install Holds files to be installed on the Palm Device during the next HotSync. This directory is used by CAOSBase to put updated files to be installed on the Palm such as new child data tables, clean observation files, or a new copy of the CAOS application.

\Backup Stores all the data files on the Palm downloaded during the last Hotsync for backup purposes. These files are used by CAOSBase for retrieving Observation Data.

Example for Palm P0001

C:\Program Files\Palm\P0001\Install Files to install to Palm P0001 at next Hotsync.

C:\Program Files\Palm\P0001\Backup Copy of Data Files on Palm P0001 downloaded during the last Hotsync

#### ActiWatch Program Files

Files are located in the Following Directory:

C:\PROGRAM FILES\Actiware

No changes should be required related to ActiWatch Program Files.

#### CAOS Data Files

Files are stored in the following Directory

C:\CAOS

The following Sub-Directories are included:

C:\CAOS\Palm Installs Contains the most recent version of the CAOS application and the CAOS database files, including clean observation files. Updates to the CAOS application should be placed here for use in installing to the Palms through CAOSBase.

C:\CAOS\Data Contains Sub-directories by download date for storing the palm databases from the date specified. Palm Files are stored by CAOSBase in the following format:

Palm File Naming Conventions

<PALMID><FILEID><DATE><FileType>

Palm ID The Palm the data was collected from

File ID The number of the file downloaded from the Palm. Only significant if multiple downloads occur from the same palm in the same day.

Date Download Date in CCYYMMDD format.

File Type Pobs.pdb contains observation information.

Pobsinstance.pdb contains observation data points.

Watch Naming Conventions

<CHILD ID><DATE>

ChildID The Child ID for the child associated with the watch data.

Date Download Date in CCYYMMDD format.

Examples:

C:\CAOS\DATA\20010527\P0202120010527pObs.pdb

Palm P0202 first download for 27/05/2001 observation information

C:\CAOS\DATA\20010527\P0202120010527pObsinstance.pdb

Palm P0202 first download for 27/05/2001 observation data

C:\CAOS\DATA\20010527\02121220010527.awd

Watch data for child id 021212 on 27/05/2001

**Chapter 9: Backup / Maintenance Processes:**

##### Daily

CAOSBase makes a new directory under C:\CAOS\Data each day for the current date in CCYYMMDD format. This directory should be copied to disk and then transferred to the backup base station on a daily basis.

Procedures for transferring to the backup base station

1. Connect the network between the 2 systems, and log on using the appropriate user name and password.
2. Using my Network Places, locate the backup station and then select the CAOS directory and the Data Sub-directory. Drag the current day’s data files to the backup system.

##### Weekly

1. Compact and Repair COASBaseDB.mdb file in C:\CAOS.
2. Copy CAOSBaseDB.mdb to c:\CAOS\Backup

Steps 3-5 if using CAOSBase for analyzing Watch Data

1. Compact and Repair AWBaseDB.mdb file in C:\CAOS.
2. Copy AWBaseDB.mdb to c:\CAOS\Backup
3. Copy Blank copy of AWBaseDB.mdb from

c:\Program Files\CAOSBase\WatchRefresh

to c:\program files\CAOSBase

1. Copy the database backup copy to the backup base station using the same procedures as the daily backup, except copy to the CAOS\backup directory instead of the CAOS\data directory.

*To Compact and Repair a Database*.

Locate the file on your system and double-click it to start Access. Once in Access, go to tools, database utilities, compact and repair database. You should always perform this step before archiving or copying the database file.

##### Bi-Weekly or Monthly (as Available)

1. Transport backup base station to Kathmandu with network card installed.
2. Connect to an open port in the NNIPS network.
3. Go to the Network Server and my Network Places. You will need an authorized person to log on to the Server if it is not accessible.
4. Locate the backup system on the network, under workgroup.
5. Under CAOS, copy the new data file directories (since last upload) to the C:\CAOS\data archives directory. You can use copy and paste, or drag and drop techniques.
6. Under CAOS, copy the latest database copy from CAOS\backup to the C:\CAOS\CAOSBase archives, using same techniques as step 5.
7. Rename the CAOSBaseDB.mdb file to add a date of upload designation.

Example: CAOSBaseDB-27-08-2001.mdb

*As Available or Requested*:

1. Copy C:\CAOS directory on the Kathmandu Network Server to CD or floppy for transportation to Baltimore.

Additional Notes about Network Operations:

Network Operations should facilitate ease in moving data from Base Station to Backup Station, from CAOS Base Stations to personal computers for further analysis, and for upload to Kathmandu systems.

Network cards have been installed in both base stations and ESiegel and should be transferable between devices as needed. When connecting 2 computers, both the 2 network cables (one from each machine) and the coupler connecting them are needed. The network will not function with only one cable.

Security comes in play. To access information on another computer you must have appropriate rights. If you do not have inherent rights, you must “log on” to the other machine before you are allowed access to the machine’s data. This impacts how data is transported between devices.

For access between ESiegel and the base stations: ESiegel does not have privlidges to access the base stations, however base stations should have access to the C:\transfer directory on ESiegel. (This can also be utilized to download data /files from the Kathmandu network to ESiegel. As such to move data to / from ESiegel, utilize the base station, my network places to find ESiegel on the network. Then access the transfer directory and move or retrieve files as desired.

For access between Kathmandu Network Server and Base Stations. Base stations do not have access to the network. After making the connection, go to the server and locate the Base Station system in the workgroup area. Find the Base station by name and then the appropriate directory. Enter the appropriate name and password for the base station if required.

For access between the base stations: Either base station may be used and the other accessed by using the appropriate user name and password.

Security Information:

System 1: Caos1

UserID: caosbase1

Password: xxxxx

System 2: Caos2

UserID: caosbase2

Password: xxxxx

**N. Darrell Mast’s system support documentation**

For Darrell Mast

January 8, 2002

**Table of Contents**

1. Introduction
2. Hardware Specifications/ Software Configurations
   1. Base Stations
   2. Palm Devices
   3. ActiWatches
3. Data Flows
   1. Physical Flow Charts
   2. Data Table Specifications
   3. Data Sizing Estimates

Appendix A: Purchasing – Supplies

Appendix B: Nepal Configuration Information

Appendix C: Nepal Observers

Appendix D: CAOS User Guide

Appendix E: CAOSBase User Guide

**I.** **Introduction**

Overview of Manual and Procedures

This document describes data collection instruments for sub-study activities involving the monitoring of activity levels and social behavior of children. The sub-study will take place in Nepal in association with NNIPS (Nepal Nutrition Intervention Project – Sarlahi). A related sub-study will occur in Zanzibar on the island of Pemba.

The scope of this document deals specifically with data collection from direct observations of the child and from data recorded by ActiWatch devices placed on the child. Identification data about the children will be furnished from the larger study.

In Nepal, Observations will be as follows:

1. The period of the study is 1.5 years.
2. Approximately 700 children will be observed in the study.
3. Children will reside in Ishwapur (27) and will be between 4 and 16 months at Baseline.
4. Physical Observations will occur 5 times at 3 month intervals during the course of the study (Baseline,3M, 6M, 9M, 12M.)
5. Physical Observations will be 3 hours in length and will occur between the hours of 8 AM and 12 PM. Observations will record data for 17 activity codes and 15 social behaviors at 15 second intervals.
6. ActiWatch devices will be placed on the children the day before the Physical Observation at approximately 2 PM but before 4 PM and will be removed after the Physical Observation at approximately 12 PM. Total duration of watch data will be approximately 20 hours per session.
7. There will be a total of 15 observers trained to perform Physical Observations. 13 Observers are planned to perform observations each day for a total of 65 observations per normal week.

Data from the Physical Observations will be collected using CAOS software deployed on a Palm Device. Between each observation, data from CAOS and from the ActiWatch devices will be offloaded to a “Base” collection station and the devices will be configured for the next observation.

Once the data is on the “Base” station, CAOSBase software will be utilized to backup the data, to synchronize ActiWatch and CAOS data and to prepare the data for initial analysis for quality control purposes.

Depending on the actual size of the data and the processing power of the base station, further analysis may occur directly on the base station or after the data is offloaded to another device.

### II. Hardware Specifications / Software Configurations

**A. Base Stations**

The Base Stations selected for the study are Pentium II Laptops, running Windows 2000 Professional. The stations should be equipped with a USB Port for connecting a Cradle, used for connection to the Palm devices, and a serial port for connecting the ActiWatch serial reader.

2 Base Stations are provided for the study location: A primary station, and a backup station. Under normal operations, only the primary station should be utilized; however, both stations should be configured identically.

The following software should be installed when the Base Stations are configured.

1. Windows 2000 Professional
2. Office 2000 + SR 1
3. Norton AntiVirus Software (NAV)
4. Updated Virus File
5. Visual Basic Package for CAOSBase Software
6. CAOSBase Icon Files
7. PalmCntl Software (for CAOSBase integration with Palm Software)
8. Configure Access Database for ODBC – “BASECAOS”
9. CAOS software for Palm Installs
10. Actiware Rhythm Software
11. Acrobat Reader 4.0

**B. Palm Devices**

**C. ActiWatches**

1 watch sample per day per observer

- 22 hours starting at 2pm to 12 noon following day
- Activity Levels captured for 15 Second Intervals

## **III. Data Flows**

A. Physical Flow Charts

**B. Data Table Specifications**

**Palm Data Tables**:

Format: Palm Database Structure: PDB

PDefault: Stores information about Palm Settings, Control Information

PChild: Stores Child information, imported from supporting study

PObserver: Stores Information about Observers

PObs: Stores General Information about the Observation

PObsInstance: Stores Each Specific Observation Point

**Backup Palm Tables**

Format: Same as Associated Palm Data Tables <PDB>

Naming: <PalmNumber><Date – MMDDYYYY><ID><Table Name>

Example: P0001033120001Pobs

**Actiwatch Data Files**:

Format: Sequential File

Headers: Line 1: Child Name (Use ChildID)

Line 2: Observation Date

Line 3: Start Time

Line 4: Interval Setting

Valid Values: 1: 15 Seconds

2: 30 Seconds

3: 1 Minute

4: 2 Minutes

5: 5 Minutes

6: 10 Minutes

7: 15 Minutes

8: 30 Minutes

9: 1 Hour

Line 5: Child Age

Line 6: Watch ID

Line 7: Child Sex (M/F)

Data Points: String, 1 per line

**CAOSBase Data Tables**

Format: Access Database Structure: MDB

FileName CAOSBASEDB.MDB

# Child

| **Name** | **Type** | **Size** | **Description** | **Valid Values** |
| --- | --- | --- | --- | --- |
| ChildID | char | 6 | Primary Key | From NNIPS4 |
| FirstName | char | 30 | First Name |  |
| LastName | char | 30 | Last Name |  |
| SexCD | char | 1 | Sex | 1 = Male  2 = Female |
| DOB | char | 8 | Date of Birth - Roman (CCYYMMDD) | Valid Date |
| MotherFirstName | char | 30 | First Name of Mother |  |
| MotherLastName | char | 30 | Last Name of Mother |  |
| FatherFirstName | char | 30 | First Name of Father |  |
| FatherLastName | char | 30 | Last Name of Father |  |
| LocationAdr | char | 7 | Location of Home Address |  |

# Observer

| **Name** | **Type** | **Size** | **Description** | **Valid Values** |
| --- | --- | --- | --- | --- |
| ObserverID | char | 6 | Primary Key |  |
| ObserverName | char | 20 | Observer Name |  |

# Codes (Palm Code Lookup Table)

| **Name** | **Type** | **Size** | **Description** | **Valid Values** |
| --- | --- | --- | --- | --- |
| ObsTypeCD | char | 1 | Primary Key  Type of Observation Code | 1 = Activity Code  2 = Behavior Code |
| ObsValueCD | char | 1 | Primary Key  Observation Code Value | Valid Activity or Behavior Code |
| ObsTypeName | char | 20 | Type of Observation | Activity  Behavior |
| ObsValueName | char | 20 | Name of Observation Value |  |
| ObsValueDesc | char | 255 | Description of Observation Value |  |

# Obs (Palm Observation)

| **Name** | **Type** | **Size** | **Description** | **Valid Values** |
| --- | --- | --- | --- | --- |
| PalmID | Char | 5 | Palm ID | P0001-P9999 |
| FileID | Char | 2 | File ID – (based on file number for current day) |  |
| ObsID | Char | 2 | Observation ID – Number of Observation within File |  |
| ObserverID | Char | 4 | Observer ID (Foreign Key) |  |
| ChildID | Char | 6 | Child ID (Foreign Key) |  |
| ObsLocationAdr | Char | 7 | Location for Observation |  |
| ObsDate | Char | 8 | Observation Date – Roman (CCYYMMDD) | Valid Date |
| ObsStartTime | Char | 6 | Observation Start Time – Roman HHMMSS | HH 00-23;  MM 00-59;  SS 00-59 |
| ObsIntervalSec | Integer |  | Interval for Observation sampling in Seconds | 0-60 |
| ObsLengthMin | Integer |  | Length of Observation in Minutes | 1-480 |

Primary Key Consists of – PalmID, FileID, ObsID, and ObsDate

# ObsInstance (Palm Observation Instance)

| **Name** | **Type** | **Size** | **Description** | **Valid Values** |
| --- | --- | --- | --- | --- |
| PalmID | Char | 5 | Palm ID | P0001-P9999 |
| FileID | Char | 2 | File ID – (based on file number for current day) |  |
| ObsID | Char | 2 | Observation ID – Number of Observation within File |  |
| ObsDate | Char | 8 | Observation Date – Roman (CCYYMMDD) | Valid Date |
| ObsTime | Char | 6 | Time Elapsed from start in seconds |  |
| ObsTypeCD | Char | 1 | Type of Observation Recorded | 1 = Activity Code  2 = Behavior Code |
| ObsValue | Char | 1 | Value of Observation |  |

# PDefault (Palm Defaults – Access Table used solely for Creation of Palm File)

| **Name** | **Type** | **Size** | **Description** | **Valid Values** |
| --- | --- | --- | --- | --- |
| ObserverID | Char | 6 | Default Observer ID |  |
| ObsIntervalSec | Integer |  | Default Interval for Observation sampling in Seconds | 0-60 |
| ObsLengthMin | Integer |  | Default Length of Observation in Minutes | 1-480 |
| TonePitchVal | Integer |  | Default Pitch for Observation Beep |  |
| ToneDurationVal | Integer |  | Default Length of Observation Beep |  |

# AWObs (ActiWatch Observation)

| **Name** | **Type** | **Size** | **Description** | **Valid Values** |
| --- | --- | --- | --- | --- |
| WatchObsID | Integer |  | Primary Key |  |
| WatchID | Char | 7 | Observer ID (Foreign Key) |  |
| ChildID | Char | 6 | Child ID (Foreign Key) |  |
| ObsDate | Char | 8 | Observation Date – Roman (CCYYMMDD) | Valid Date |
| ObsStartTime | Char | 6 | Observation Start Time – Roman HHMMSS | HH 00-23;  MM 00-59;  SS 00-59 |
| ObsIntervalSec | Integer |  | Interval for Observation sampling in Seconds | 0-60 |

# AWObsInstance (ActiWatch Observation Instance)

| **Name** | **Type** | **Size** | **Description** | **Valid Values** |
| --- | --- | --- | --- | --- |
| WatchObsID | Integer |  | Primary Key |  |
| ObsTime | Char | 6 | Time Elapsed from start in seconds |  |
| ObsValue | Char | 1 | Value of Observation |  |

**C. Data Sizing Estimates**

**Palm Observations**:

700 Kids * 5 (Baseline, 3M, 6M, 9M, 12M) = 3500 Observations

Estimated File Size per Observation:

Estimated Database Table Size per Observation:

Daily: 12 Observations: File Size
 DB Addition

Weekly:

By Observations Cycle:

Total:

**Watch Observations**:

700 Kids * 5 (Baseline, 3M, 6M, 9M, 12M) = 3500 Observations

Estimated File Size per Observation:

Estimated Database Table Size per Observation:

Daily: 12 Observations: File Size
 DB Addition

Weekly:

By Observations Cycle:

Total:

### Appendix A: Purchasing / Supplies

Desc Amt Status

Initial: 1 Palm (Developer) $150 est. Purchased

CDW

Nepal: 6 Palms with cradles @$166 ea $996 Purchased

PC Connections

7 Innogear Memory/Vibrator Pack @$44.95 ea $314.65 Purchased

[www.innogear.com](http://www.innogear.com/)

Palms: 29 Palms / Solo @$150 ea $4321.29 Not Ordered

[www.handspring.com](http://www.handspring.com/)

Innogear Memory / Vibrator Pack @ 44.95 ea $1303.55 Not Ordered

Total: Est: $7085.49

Additional Needs:

- Batteries, life est.
- Transport Media – CDRW or Other
- Software??

**Appendix B: Nepal Configuration Information**

HandSpring Visors:

Initial Deployment

| **Palm ID** | **Serial Number** |
| --- | --- |
|  |  |
| P0202 | AAAEDO5106389 |
| P0203 | AAAEDO5106396 |
| P0204 | AAAEDO5106380 |
| P0205 | AAAEDO3111219 |
| P0206 | AAAEDO5106362 |
| P0207 | AAAEDO5106480 |
| P0208 | AAAEDO3111265 |
| P0209 | AAAEDO5106363 |
| P0210 | AAAEDO5106356 |
| P0211 | AAAEDO3111318 |
| P0212 | AAAEDO5106431 |
| P0213 | AAAEDO5106447 |
| P0214 | AAAEDO3111033 |
| P0215 | AAAEDO5106446 |
| P0216 | AAAEDO3111537 |

Defective Palms:

P0206

P0209

P0212

P0213

Damaged Palms

P0207 – Screen Damaged by Collision with Water Buffalo

Replacement / Spare Palms

Innogear – InnoPak/ 2V Memory Modules:

T02201-0401469, T02201-0401426, T02201-0401459, T02201-0400968, T02201-0401401

**Appendix C: Nepal Observers**

| **Observer ID** | **Observer Name** |
| --- | --- |
| 0201 | SHISHIRVAR SHRESTHA |
| 0202 | DIPAK GHIMIRE |
| 0203 | ROSHAN SHRESTHA |
| 0204 | ISHWORI SHRESTHA |
| 0205 | YADAV MAIWALI |
| 0206 | RUDRA PAUDEL |
| 0207 | RAM ASESHWOR CHAU |
| 0208 | MENUKA CHALISE |
| 0209 | KHADGA BDR BUDHA |
| 0210 | SHYAM THAPA |
| 0211 | SULOCHANA CHAUDHARI |
| 0212 | KESHAB DHAKAL |
| 0213 | KIRAN CHAUDHARI |
| 0214 | PADAM BDR LAMA |
| 0215 | NIR BDR KARKI |
| 0216 | RAM NR CHAUDHARI |

**Functional List for all systems (Reference)**

For Development and Verification – Functional List for CAOS application suite:

CAOS

Select Child

Select Observer

View Codes on File

Select Observation Defaults

Make Observations

Review Observation

Backup Data on Module

Datebook for Scheduling – Future

CAOS BASE

Upload Data

Backup of Palm Files

View Palm Files

Load of Database from Palm Files ( Palm to DB conversion)

View ActiWatch Files (?)

Load of Database from ActiWatch Files

Load Child Data from Main system

Load Observer Data – manual or from main system (same as update)

Configure Palm

Setup Palm Defaults – user, etc

Set up Palm User account

Install CAOS on PALM

View/Update Palm Files

Refresh Palm

Reinstall CAOS on PALM

Establish new Palm Files for new observation

Query

View Data Tables – From DB

Allow SQL Access

Reports

Statistical Frequency Reports

ActiWatch vs. Palm Synchronization

Update

Data Table Updates (as needed)

Export

Allow Data exports to Text, Excel?
